# Clinical indicators for common paediatric conditions: Processes, provenance and products of the *CareTrack Kids* study

**DOI:** 10.1371/journal.pone.0209637

**Published:** 2019-01-09

**Authors:** Louise K. Wiles, Tamara D. Hooper, Peter D. Hibbert, Charlotte Molloy, Les White, Adam Jaffe, Christopher T. Cowell, Mark F. Harris, William B. Runciman, Annette Schmiede, Chris Dalton, Andrew R. Hallahan, Sarah Dalton, Helena Williams, Gavin Wheaton, Elisabeth Murphy, Jeffrey Braithwaite

**Affiliations:** 1 Australian Centre for Precision Health, School of Health Sciences, Cancer Research Institute, University of South Australia, Adelaide, South Australia, Australia; 2 Centre for Healthcare Resilience and Implementation Science, Australian Institute of Health Innovation, Faculty of Medicine and Health Sciences, Macquarie University, Sydney, New South Wales, Australia; 3 South Australian Health and Medical Research Institute (SAHMRI), Adelaide, South Australia, Australia; 4 Centre for Health Systems and Safety Research, Australian Institute of Health Innovation, Faculty of Medicine and Health Sciences, Macquarie University, Sydney, New South Wales, Australia; 5 Australian Patient Safety Foundation, Adelaide, South Australia, Australia; 6 Centre for Health Informatics, Australian Institute of Health Innovation, Faculty of Medicine and Health Sciences, Macquarie University, Sydney, New South Wales, Australia; 7 Discipline of Paediatrics, School of Women’s and Children’s Health, University of New South Wales, Sydney, New South Wales, Australia; 8 Sydney Children’s Hospital, Sydney Children’s Hospitals Network, Randwick, Sydney, New South Wales, Australia; 9 New South Wales Ministry of Health, North Sydney, Sydney, New South Wales, Australia; 10 Department of Respiratory Medicine, Sydney Children’s Hospital, Sydney Children’s Hospitals Network, Randwick, Sydney, New South Wales, Australia; 11 Sydney Medical School, University of Sydney, Sydney, New South Wales, Australia; 12 Institute of Endocrinology and Diabetes, Children’s Hospital at Westmead, Sydney Children’s Hospitals Network, Westmead, Sydney, New South Wales, Australia; 13 Centre for Primary Health Care and Equity, Faculty of Medicine, University of New South Wales, Sydney, New South Wales, Australia; 14 BUPA Health Foundation Australia, Sydney, New South Wales, Australia; 15 Children’s Health Queensland Hospital and Health Service, South Brisbane, Brisbane, Queensland, Australia; 16 New South Wales (NSW) Agency for Clinical Innovation (ACI), Chatswood, Sydney, New South Wales, Australia; 17 Russell Clinic, Blackwood, Adelaide, South Australia, Australia; 18 Australian Commission on Safety and Quality in Health Care, Sydney, New South Wales, Australia; 19 Southern Adelaide Local Health Network, Bedford Park, Adelaide, South Australia, Australia; 20 Cancer Australia, Surry Hills, Sydney, New South Wales, Australia; 21 Adelaide Primary Health Network, Mile End, Adelaide, South Australia, Australia; 22 Country SA Primary Health Network, Nuriootpa, Adelaide, South Australia, Australia; 23 Division of Paediatric Medicine, Women’s and Children’s Health Network, Adelaide, South Australia, Australia; Beatrix Children's Hospital, University Medical Center Groningen, NETHERLANDS

## Abstract

**Background:**

In order to determine the extent to which care delivered to children is appropriate (in line with evidence-based care and/or clinical practice guidelines (CPGs)) in Australia, we developed a set of clinical indicators for 21 common paediatric medical conditions for use across a range of primary, secondary and tertiary healthcare practice facilities.

**Methods:**

Clinical indicators were extracted from recommendations found through systematic searches of national and international guidelines, and formatted with explicit criteria for inclusion, exclusion, time frame and setting. Experts reviewed the indicators using a multi-round modified Delphi process and collaborative online wiki to develop consensus on what constituted appropriate care.

**Results:**

From 121 clinical practice guidelines, 1098 recommendations were used to draft 451 proposed appropriateness indicators. In total, 61 experts (n = 24 internal reviewers, n = 37 external reviewers) reviewed these indicators over 40 weeks. A final set of 234 indicators resulted, from which 597 indicator items were derived suitable for medical record audit. Most indicator items were geared towards capturing information about under-use in healthcare (n = 551, 92%) across emergency department (n = 457, 77%), hospital (n = 450, 75%) and general practice (n = 434, 73%) healthcare facilities, and based on consensus level recommendations (n = 451, 76%). The main reason for rejecting indicators was ‘feasibility’ (likely to be able to be used for determining compliance with ‘appropriate care’ from medical record audit).

**Conclusion:**

A set of indicators was developed for the appropriateness of care for 21 paediatric conditions. We describe the processes (methods), provenance (origins and evolution of indicators) and products (indicator characteristics) of creating clinical indicators within the context of Australian healthcare settings. Developing consensus on clinical appropriateness indicators using a Delphi approach and collaborative online wiki has methodological utility. The final indicator set can be used by clinicians and organisations to measure and reflect on their own practice.

## Introduction

Despite efforts aimed at achieving quality, equity, and sustainability of healthcare systems[1–5], gaps remain between the care that is recommended (appropriate care, in line with evidence and/or clinical practice guidelines (CPGs)) and that which is delivered[6, 7]. To prioritise resources and develop strategies to address the inappropriateness of and variations in care[8], national measurement and monitoring activities are needed to capture what population-level care is given, and by whom[9–12]. Internationally, clinical standards and indicators are increasingly being used to identify gaps and areas for improvement, and understand and measure the quality of healthcare provided[13–22]. Data evaluating appropriateness of healthcare for children is limited, especially at population level[23, 24].

Interventions aimed at delivering care in line with CPGs mostly report limited or variable success[25–31]. However, there is some evidence that both compliance with accepted care processes and favourable clinical outcomes are possible[26, 27, 32–35], and may be facilitated by multi-faceted nationally-based initiatives using clinical indicator-based adherence approaches coupled with audit and feedback[36–41].

Clinical indicators can be developed using one of three main systematic approaches[42–44]: evidence, such as using scientific data from clinical trials[45–47]; combining evidence with consensus, such as a Delphi technique[17, 48] or RAND appropriateness method[23, 49, 50]; and CPG-driven derivation from recommendations in current CPGs[19, 51, 52]. While the merits and demerits of different clinical indicator development methods have been explored[53–56], the trend for contemporary indicator development centres on employing hybrid approaches[22, 57, 58].

As interpretation of performance measured by clinical indicators can have far-reaching consequences (e.g. public reporting, pay-for-performance), it is important to ensure that they are developed in a way that reflects what is reasonably expected of clinicians[57]. For example, for the purposes of a medical record audit, this may be best achieved through combining consensus-based (i.e. stakeholder perspectives / expert opinion) and CPG-driven (i.e. recommendations to clinicians to guide care). The Delphi technique, a structured process comprised of several rounds of review to gather stakeholder perspectives until consensus is reached[17], can be used to vet recommendations derived from CPGs to develop healthcare quality indicators[17]. While a number of studies have used combined methods to create clinical indicators, of these most focus on a single condition[58, 59], clinical area or care process[60, 61] or healthcare setting[62, 63].

Built on the findings and experience of the *CareTrack Australia* study[11, 51], the objective of the *CareTrack Kids* study was to determine the appropriateness of healthcare delivered to children in Australia for common conditions[64]. This paper describes a core component of the *CareTrack Kids* study; the development of a set of clinical appropriateness indicators for common paediatric conditions for use across a range of healthcare practice facilities, including primary care provided by general practitioners, secondary care provided by outpatient paediatricians and tertiary care in hospitals[65]. Our method married recognised Delphi processes[17] with a collaborative online wiki[66] to achieve consensus on what constitutes appropriate care, using CPGs as a primary information source. We report on this indicator development; detailing the processes (panel recruitment; methods of indicator development and criteria for selection), provenance (origin and evolution of original recommendations and indicators throughout the study including reasons for exclusion) and products (characteristics of the final set of indicators including linking these to evidence levels and grades of recommendations).

## Methods

The three components of our indicator development work[65] were to (1) identify and select common candidate paediatric conditions (presented in our study protocol[65]), (2) develop clinical indicators representative of “appropriate care” for these conditions, and (3) refine them for feasibility, applicability and utility. Our approach has been described in our study protocol[65]. Terms used in *CTK* and their definitions are presented in Box 1. This study was approved by the Macquarie University Human Research Ethics Committee (protocol 5201401120).[65]

**Box 1. Definitions of terms used in the *CareTrack Kids* study.**

A clinical practice guideline (CPG):

“Statements that include recommendations intended to optimise patient care that are informed by a systematic review of evidence and an assessment of the benefits and harms of alternative care options.”[67, 68]

A clinical standard[11]:

- is an agreed process that should be undertaken or an outcome that should be achieved for a particular circumstance, symptom, sign or diagnosis (or a defined combination of these)
- should be evidence-based, specific, feasible to apply, easy and unambiguous to measure, and produce a clinical benefit and/or improve the safety and/or quality of care, at least at the population level.

If a standard cannot or should not be complied with, the reason/s should be briefly stated.

A clinical indicator[11]:

- describes a measurable component of the standard, with explicit criteria for inclusion, exclusion, time frame and setting.

A clinical tool[11, 69–72]:

- should implicitly or explicitly incorporate a standard or a component of a standard
- should constitute a guide to care that facilitates compliance with the standard
- should be easy to audit, preferably electronically, to provide feedback
- should be able to be incorporated into workflows and medical records.

Appropriate care[11, 51, 73]:

- care in line with evidence-based or consensus-based guidelines

A wiki[66, 74, 75]

- is an interactive information management system which will allow users (e.g. healthcare professionals who register for, and login to the wiki) to collaborate directly in formulating and refining indicators that are relevant to their clinical practice and lived experience.

Underuse[76]

“Failure to deliver a service that is highly likely to improve the quality or quantity of life, that represents good value for money, and that patients who were fully informed of its potential benefits and harms would have wanted.”

Overuse[76]

“Provision of a service that is unlikely to increase the quality or quantity of life, that poses more harm than benefit, or that patients who were fully informed of its potential benefits and harms would not have wanted.”

We initially identified 21 common paediatric conditions (Box 2). Clinical indicators representative of their appropriate care were developed, using a four-stage process:

1. systematic search and source relevant CPGs;
2. select, draft and format proposed clinical indicators;
3. review indicators internally and externally (via a modified Delphi approach); and
4. refine and convert indicators to individual medical record audit indicator items suitable for use in a wide range of circumstances[64].

**Box 2. Paediatric conditions included in the indicator development process in the *CareTrack Kids* study (n = 21)**

           Acronym           Condition

           ABDO           Acute abdominal pain

+           ADHD           Attention Deficit Hyperactivity Disorder

               AGE           Acute gastroenteritis

+           ANXI           Anxiety

+           ASTH           Asthma

               AUTI           Autism

               BRON           Acute bronchiolitis

               CROU           Croup

+           DEPR           Depression

+           DIAB           Diabetes

               ECZE           Eczema

               FEVE           Fever

               GORD           Gastro–Oesophageal Reflux Disease

+           HEAD           Head injury

+           OBES           Obesity

               OTIT           Otitis media

+           PREV           Preventive care

               SEIZ           Seizures

               TONS           Tonsillitis

               URTI           Upper Respiratory Tract Infection

               URIN           Urinary Tract Infection

+           *NHPA           National Health Priority Area*

### Stage 1 Search and source relevant CPGs

Clinical indicators were derived from published CPGs relevant for 2012 and 2013. A systematic search was undertaken, in order of priority, for national-level CPGs from Australia (e.g. from the National Health and Medical Research Council (NHMRC))[77], and internationally[78–80]. In the absence of Australian, national or international CPGs, those from relevant professional medical colleges and associations were examined, as well as those from state jurisdictional bodies or professional groups [81, 82]. Three Research Group members (LKM, TDH, PDH) conducted the CPG searches and developed the initial set of clinical indicators. Full details of the search strategy are provided in online S1 Table of our protocol paper[65].

### Stage 2 Select, draft and format the proposed indicators

Recommendations from each CPG were extracted verbatim, along with their documented grade or level of evidence, and compiled in a Microsoft Excel spreadsheet. In instances where there was more than one guideline for a recommendation, we recorded all grades and levels of evidence. Similar recommendations across CPGs were grouped together to minimise duplication. Not all published recommendations became indicators; using our experience of developing and ‘field-testing’ 522 indicators in the CareTrack Australia (adult) study[51], we applied a set of exclusion criteria (Table 1) based on:

- Strength/certainty of the wording of the recommendation (i.e. “may”, “could” and “consider” statements were excluded)
- Low likelihood of information being documented in the medical record
- Guiding statements without recommended actions
- Out of scope of the CTK study (i.e. “structure-level” recommendations aimed at attributes of the settings in which care is delivered[12, 43]).

**Table 1:** Examples of current recommendations which would meet exclusion criteria.

Indicator eligibility criteria	Example exclusions	Rationale for exclusion
Strength/certainty of wording	Healthcare professionals who routinely use disposable chemical dot thermometers should consider using an alternative type of thermometer when multiple temperature measurements are required [83].	Use of term “consider” does not provide a certain or conclusive action against which medical record compliance can be assessed
Likelihood of documentation	People with autism are not prescribed medication to address the core features of autism [84]	The rationale for prescribing medication in this context is unlikely to be documented in a medical record
Guiding statement without recommended action	Be aware that the aim of weight management programmes for children and young people can vary. The focus may be on either weight maintenance or weight loss, depending on the person's age and stage of growth [85].	Guiding statement with no specific actions able to be used to determine compliance
Out of CTK scope	Streamlined referral pathways should be developed for tests not available or appropriate in primary care [86]	Structure-level recommendation for which data cannot be obtained by way of a medical record audit

All clinical indicators were written in a structured and standardised format, commencing with the inclusion criteria followed by the compliance action[51]. For example, the inclusion criteria defined the age (infant, child, adolescent), condition, and the phase of care (at diagnosis/presentation or “with”, indicating the diagnosis is existing). The compliance action defined the recommended appropriate care. Indicators were arranged chronologically according to phases of care (Table 2).

**Table 2:** Examples of clinical indicators from *CareTrack Australia* [51] that were written in a structured and standardised format.

Condition	Phase of care	Indicator (number)
Obesity	Screening	Patients who are being assessed for obesity should have their *BMI and waist circumference measured at least once in 2 years* (469).
Depression	Diagnosis	Patients newly diagnosed with depression had their *co-morbidities documented* (332).
Asthma	Treatment	Patients who presented to an emergency room or to their GP with an exacerbation of asthma and a PEFR/peak flow or FEV1 less than 70% of baseline were *prescribed an inhaled corticosteroid* (106).
Diabetes	Ongoing management	Patients with existing type 2 diabetes were *referred to a dietician or provided with dietary advice every year* (360).

inclusion criteria underlined

*compliance actions* italicised

### Stage 3 Subject the indicators to several rounds of internal and external review

**Fig 1:**
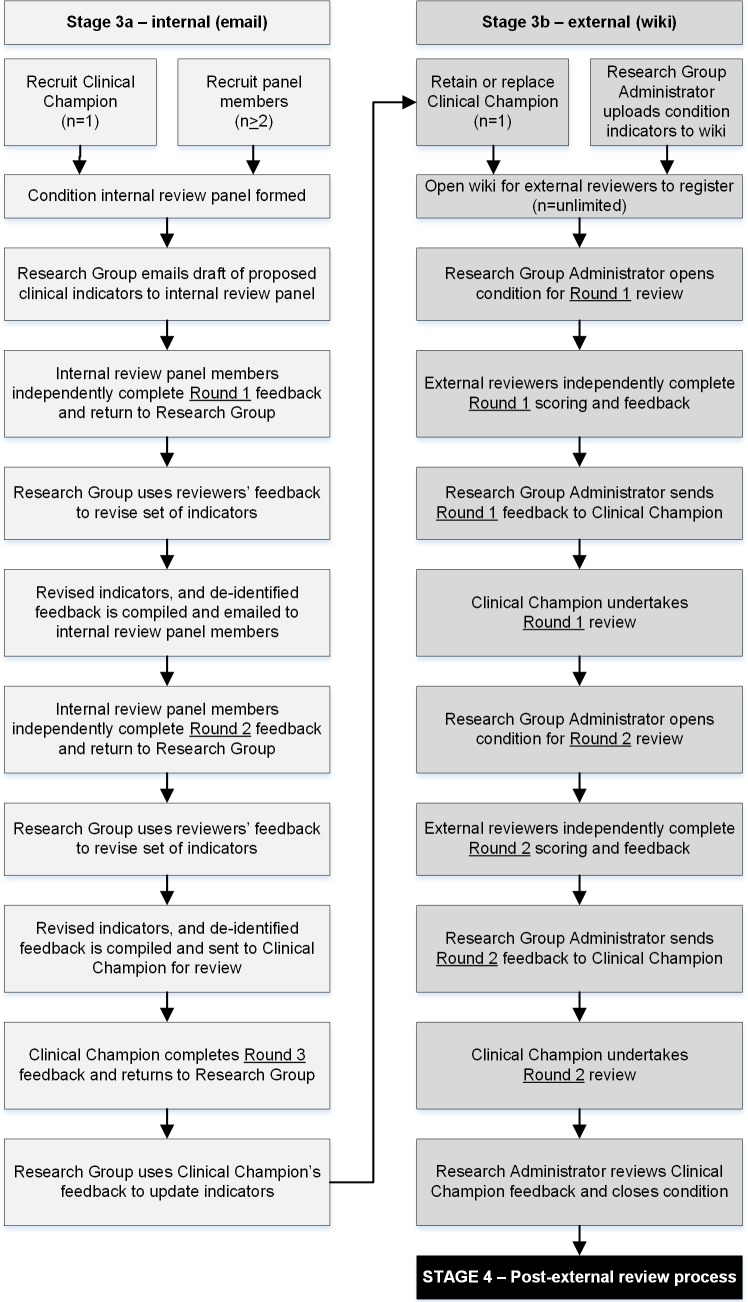
Overview of the internal and external indicator review process.

There were two stages involved in the indicator review (Fig 1). The proposed clinical indicators were subjected to an internal review (Stage 3a), followed by an external wiki style review (Stage 3b) using a modified Delphi process[87]. This multi-round, multi-modality approach aimed to enhance methodological rigor and optimise consensus with respect to the content and face validity of the final set of clinical indicators [17]. We conducted three rounds for the internal review (Stage 3a), and two rounds in the external review (Stage 3b).

### Stage 3a Internal review processes

In accordance with selection strategies employed within the Delphi process literature[17, 88], internal reviews were conducted by paediatricians and general practitioners who were identified by the research team and their professional networks. Clinical Champions, who led the internal review panel members, were employed as the head or director of a relevant paediatric department in a large hospital, held at least an adjunct academic appointment or were directly involved in clinical care. The clinical indicators for each condition were reviewed via email by a panel of at least three reviewers. Reviewers completed their assignments independently to minimise bias from “group-think”[89, 90].

The review criteria were based on methods developed in previous US and Australian studies[23, 49, 51]. Internal reviewers were asked to: score each indicator using one of three responses (yes, no, not applicable to area of expertise or clinical setting) against three key criteria; acceptability, feasibility and impact (Box 3)[65]; to recommend the indicators for inclusion or exclusion; and provide any additional comments. Research Group members (LKW, TDH, PDH) collated the feedback and revised the content, structure, and format of each indicator between review rounds.

**Box 3. Information for scoring criteria for clinical indicators[65]**

**Acceptability (A)**

- Level of evidence or grade of recommendations. In some instances a level of evidence or grade of recommendation may not have been provided. In these cases, absence of evidence should not be the only grounds for exclusion of the indicator (i.e. expert consensus may be acceptable).
- Non-Australian clinical guideline recommendations. There are some indicators where the primary source is a non-Australian clinical guideline from a reputable organisation (e.g. NICE). In the absence of Australian guidelines, it is important to consider whether such a guideline reflects what is practical within the context of Australian healthcare settings.
- Non-national Australian clinical guideline recommendations. In the absence of nationally-based Australian AND international guidelines, some indicators have been sourced using guidelines from one state or organisation e.g. NSW Health, or Royal Children’s Hospital in Melbourne.
- Recommendation is made in more than one clinical guideline.
- Reflects “essential” (i.e. independent of resources) Australian clinical practice during 2012 and 2013.

**Feasibility (F)**

- Indicators with multiple eligibility criteria tend to have lower numbers of eligible encounters.
- Compliance can be determined preferentially from one encounter with one healthcare provider, or at least within a 1–2 year period (our sample will be the medical records of healthcare encounters for children during the 2012–2013 period).
- Likely to be documented in the medical record, for example: indicators associated with lifestyle or exercise advice are less likely to be documented.

**Impact (I)**

- “High impact” on the patient in terms of domains of quality i.e. safety, effectiveness, patient experience, or access.
- “High impact” within Australian healthcare settings (e.g. what will be the frequency/ prevalence of presentation).

### Stage 3b external wiki-based review

External reviews were conducted by invited paediatricians and general practitioners. Relevant medical colleges, professional associations and networks were contacted, requesting assistance with the recruitment of clinical experts to register as external reviewers. Invitations comprised direct email requests to members, media releases and articles within newsletters. Clinical experts self-nominated as reviewers for one or more of the CTK conditions based on their interest, scope of practice and clinical experience[17, 88]. All reviewers were required to complete a Conflict of Interest (COI) declaration[91–93]; COIs were recorded for each reviewer and managed according to the NHMRC protocol[94].

Indicators for each condition (from round three of the internal review) were posted to an online wiki site. The aim was for each condition to be independently reviewed by a minimum of nine experts. In addition to the scoring criteria used in the internal review process, indicators were scored on a nine-point Likert scale as representative of appropriate care delivered to children during 2012 and 2013[23, 51, 95]. With the support of a Research Group member as a wiki site Administrator, the Clinical Champion for each condition followed-up and managed external reviewers’ responses, and made final recommendations regarding the inclusion, content, structure and format of indicators (Table 3). For most conditions, the Clinical Champion’s role was undertaken by one of the Stage 3a internal reviewers. In the second wiki round, experts had access to de-identified comments from the first round.

**Table 3:** Clinical champion management of external reviewers’ responses.

Options	Reason	Implication
Mark as final	• High ‘appropriateness’ scores • Good agreement among external reviewers	External reviewers required to provide final approval (yes/no) in Round 2
Mark updated	• Consistent feedback from external reviewers suggesting changes • Evidence exists/provided to support suggested changes	External reviewers required to rescore updated indicator using original criteria (Box 3)
Reject (add reason)	• Low ‘appropriateness’ scores • Good agreement among external reviewers to reject	External reviewers provided with the rationale for rejecting indicators

#### Consensus business rules

For the Stage 3 internal and external reviews, consensus was defined as the majority agreeing to include or exclude; when a clear majority was not able to be achieved we opted to retain the indicator and subject it to additional feedback over subsequent rounds of review. In order to facilitate consensus, the Research Group and wiki Administrator and Clinical Champion used comments fields to provide indicator reviewers with a summary of the feedback obtained to date, where relevant

### Stage 4 Refine and convert indicators to individual medical record audit indicator items

During the Stage 4 refinement process, we flagged indicators with an appropriateness score of less than 7, or more than three inclusion criteria, as these are likely to have lower prevalence, and sought the condition Clinical Champion’s approval for their exclusion. For all indicators approved for inclusion, the Research Group converted each inclusion criterion and compliance action into an individual medical record audit indicator item and formatted these such that “not applicable” (i.e. medical record did not meet the indicator’s inclusion criteria) or binary responses (yes/no) could be recorded (S1 Table). We also analysed the final set of indicators to ensure all phases of care were covered, and that the relevant indicators were ‘feasible’ for the main medical record audit[64]. Each Clinical Champion checked the individual medical record audit indicator items for their nominated condition to ensure the ‘spirit’ of the original recommendations and reviewers’ feedback from previous rounds had been accurately captured.

### Data analysis

Reviewers’ scores and comments from the internal (manually entered) and external (exported from the wiki) reviews were entered into Microsoft Excel (2013) spreadsheets. Analysis and reporting of study data is in the form of descriptive statistics for the resultant processes, provenance and products.

## Results

### Processes

A panel of 24 experts completed the internal review; each condition was allocated at least three reviewers, and each reviewer undertook reviews for no more than three conditions. For the external review, 79 participants registered and were approved for the wiki site; 37 (47%) undertook the Round 1 review for their nominated condition(s), and 24 (30%) went on to complete Round 2. The demographic characteristics of indicator reviewers are presented in Table 4. In the external review, there was a mean of 5 (SD 2.7) reviewers per condition (range 1–14; median 4) (S1 Table).

**Table 4:** Characteristics of indicator reviewers.

**Internal review**		N	%
**Profession**			
	Paediatricians	15	63
	General practitioners	7	29
	Psychiatrists	2	8
**Additional appointments**			
	Formal university affiliation	9	38
	Director Medical Unit / Hospital Service	5	21
	Research network/institute membership	4	17
	Government health department	2	8
**External review**		N	%
**Profession#**			
	Medically trained	35	83
	Paediatricians	29	83*
	General practitioners	4	11*
	Psychiatrists	2	6*
	Nursing	5	12
	Psychology	2	5
**Employment#**			
	Hospital	36	86
	Private practice	4	10
	Health-related government department^	3	7
**Appointments#**			
	Formal university affiliation	26	62
	Research network/institute membership	2	7
	Professional association role	2	7
	National accreditation organisation	2	7

# presented as a percentage of reviewers who completed either Round 1 or Round 2 (n = 42)

* presented as a percentage of medically-trained professionals

^ n = 1 had a joint appointment across hospital and government settings

### Provenance

We identified 113 relevant CPGs with supporting references, from which 1432 original recommendations were extracted. Over one-fifth of extracted recommendations (n = 334, 23%) were initially excluded by the Research Group (S2 Table). The information contained in some of these exclusions was covered in other recommendations which were included in our sample (n = 86 of 334, 26% of guiding statements; and n = 3, 4% of those excluded due to strength/certainty of wording) (S2 Table). In addition, a small proportion of the excluded guiding statements (n = 3, 1%) were incorporated into definitions which were to be provided to research surveyors to assist them in completing the medical record audit.

**Fig 2:**
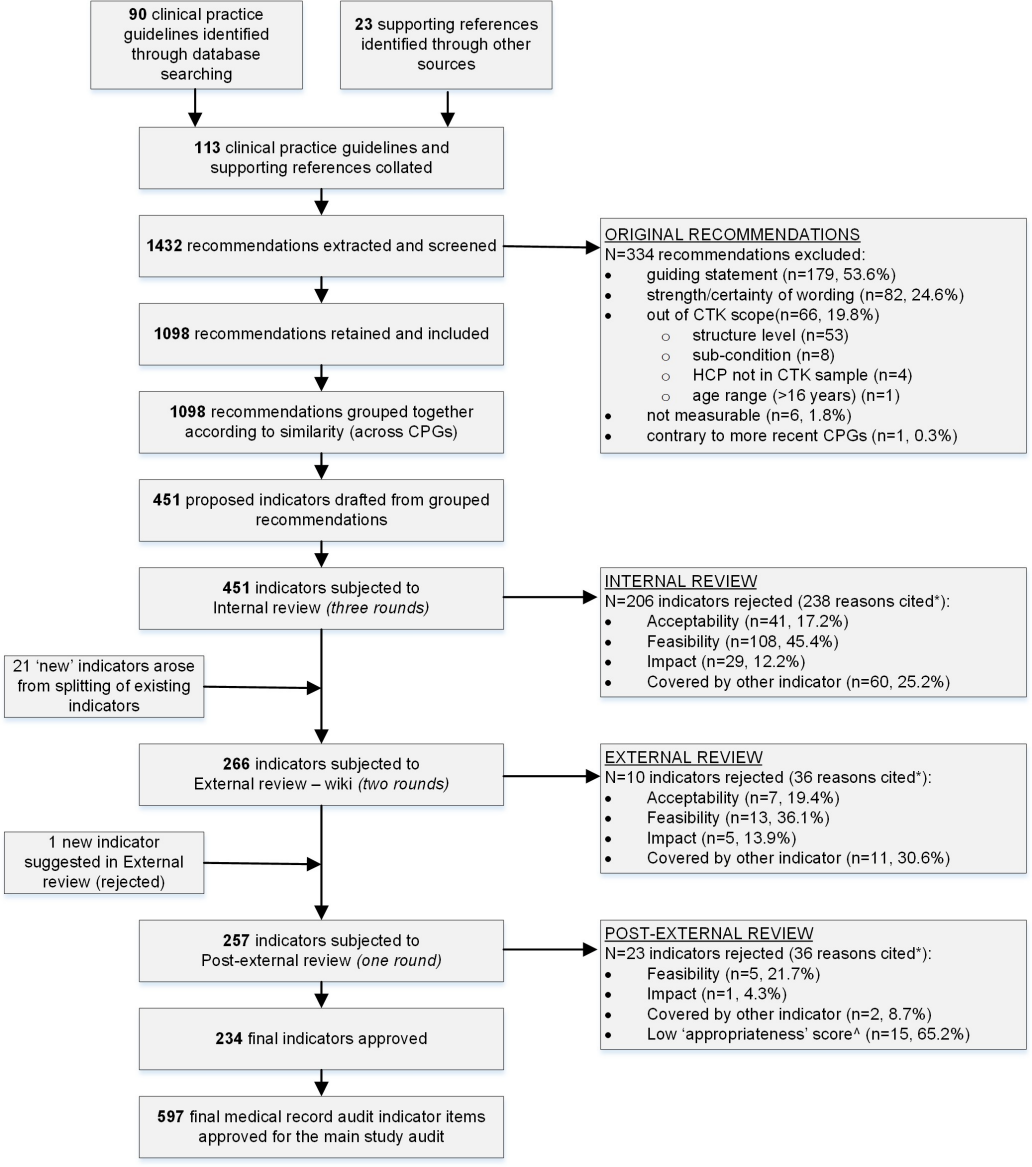
Provenance of CPGs, original recommendations, indicators and medical record audit indicator questions. * some indicators were rejected for more than one reason ^ ‘appropriateness’ score less than seven out of nine.

**Fig 3:**
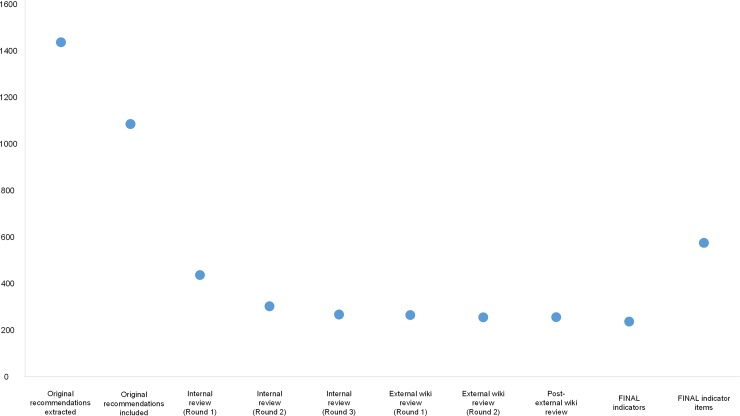
Evolution of the total number of indicators over the development process, from original recommendations to the final medical record audit indicators and indicator items.

The remaining original recommendations (n = 1098) were used to draft 451 proposed indicators for circulation among the internal review panel members (Fig 2). Following three rounds of internal review, almost half were rejected (n = 206, 46%) mainly due to concerns around the feasibility of capturing indicator information by way of a medical record audit (e.g. likelihood of the necessary documentation being present) (S3 Table). During the internal review, 245 indicators were approved for posting to the wiki for the external review, together with twenty-one ‘new’ indicators (S4 Table) developed by splitting existing indicators which contained more than one eligibility criterion and/or compliance action. The external review yielded 257 indicators (97%), with the main reasons for exclusion mirroring those from the internal review (Fig 2). S5 Table and Fig 3 present, by condition, the evolution of numbers of indicators over the development process, from original recommendations to the final indicators and medical record audit indicator items.

### Products: Indicators and medical record audit indicator items

The final 234 indicators were used to develop 597 individual medical record audit indicator items for the medical audit review (Table 5, S1 Table). In terms of classification according to phase of care, most medical record audit indicator items related to ‘treatment’ (n = 273, 46%) (S6 Table). Most items were geared towards capturing information about under-use in healthcare (n = 551, 92%) across emergency department (n = 457, 77%), hospital (n = 450, 75%) and general practice (n = 434, 73%) healthcare facilities, and were based on consensus level recommendations (n = 451, 76%).

**Table 5:** Examples of indicators with multiple inclusion criteria and/or compliance actions being converted into individual medical record items.

Indicator	Item no.	Item	Rationale
Children aged between 2–16 years are *diagnosed as follows*: - *overweight* (if BMI for age and sex in 85th - 94th percentile) OR - *obese* (if BMI for age and sex greater than 95th percentile).	OBES01	Children aged between 2–16 years with a BMI for age and sex in 85th - 94th percentile *were diagnosed as overweight*.	Multiple inclusion criteria and compliance actions
	OBES02	Children aged between 2–16 years with a BMI for age and sex greater than 95th percentile *were diagnosed as obese*.	
Children and adolescents with depression are provided with: - *evidence-based management of depression (e*.*g*. *information leaflets/booklets/reliable websites such as Beyond Blue*, *Black Dog Institute)* AND - *offered community supports (e*.*g*. *information about support services*, *such as Lifeline phone number*, *Community mental health team)*.	DEPR06	Children and adolescents with depression *were provided information and resources about evidence-based management*.	Multiple compliance actions
	DEPR07	Children and adolescents with depression *were offered community supports*.	

inclusion criteria underlined

*compliance actions* italicised

## Discussion

To our knowledge, this is the first study to detail the processes, provenance and products of developing clinical indicators of appropriate care for a range of common paediatric conditions for use across Australian primary, secondary and tertiary healthcare practice facilities. Paediatric indicator development studies over the last decade have focused on fewer paediatric conditions[23, 96, 97] or specific types of illnesses and/or healthcare practice facilities[96–99]. Our methodology was strengthened by employing a transparent, multi-stage and multi-modality modified-Delphi process which aimed to contextualise the recommendations published in CPGs (including scientific evidence) to the clinical setting (expert opinion). The Delphi procedure was reported in accordance with current recommendations[17] (S7 Table). Using our approach and definitions, we were able to achieve consensus on appropriate care for 21 paediatric conditions (in Australia for the years 2012–2013), and embody these in clinical indicators.

The main reason for excluding indicators was feasibility which included multiple eligibility criteria, compliance unlikely to be determined during a medical record audit, low likelihood of information being documented (Box 3, Fig 2), and is supported by internationally-recognised organisations[22] and literature[17]. A potential consequence of this is that using ‘feasibility’ as an eligibility criterion may serve to drag down the standard of measuring what is deemed appropriate care towards the care we are expecting to be documented rather than that which should be delivered. Recommendations were also excluded due to the strength/certainty of their wording (e.g. “may”, “could”, “consider” statements), which means that our indicator set did not cover aspects of care that may be influenced by situational factors and/or patient preferences; this presents a gap in their clinical utility. A first step to capturing information about these aspects of care is improving the detail and consistency of clinicians’ documentation (e.g. consideration of differential diagnoses, decision making based on situational factors and/or patient preferences). In the future, and as standardised electronic medical records become more commonplace and sophisticated, this may be facilitated by structured and mandatory fields of entry[11], as well as shared access and decision-making between patients and clinicians using integrated electronic apps and medical record software to inform, guide and record care; and especially variations in care as a result of situational factors or preferences [100, 101].

Application of the CTK indicators for research purposes has been described in the main results paper [102] and a condition-specific analysis for tonsillitis [103]. While originally developed for use in a large-scale research medical record audit, the CTK indicator set can be used by clinicians and organisations to measure and reflect on their own local practice (Table 6). In this way, data can be aggregated by individuals or groups of practitioners, hospital departments and local or jurisdictional health networks to determine baseline adherence with recommended care [104–106], and identify and target practice gaps with professional development and other quality improvement activities. For aspects of care that are not covered by the current CTK indicator set, supplementary data collection methods such as case studies, patient satisfaction surveys, narrative-text analyses of clinical notes, and clinician/patient interviews may need to be considered[107].

**Table 6:** Guidance on the clinical application of the CTK indicators in a medical record audit.

CTK indicator feature	Clinical application in a medical record audit
HCP type	Specifies the setting(s) for which each indicator is applicable
Inclusion criteria	• Specifies the patients who are eligible to have their documented care assessed against the indicator • The number of patients within a sample who are eligible form the denominator in calculations of percentage adherence
Compliance action	• The number of patients within a sample whose care was adherent to the compliance action form the numerator in calculations of percentage adherence

There are several caveats to our findings. First, the final set of clinical indicator items was based on recommendations in CPGs relevant for the years 2012–2013, with priority given to those published in Australia. While this limits the applicability and generalisability of the indicators beyond these contexts, they do provide a basis from which new indicators may be derived and adapted to local settings. We did not critically appraise the quality of included CPGs; for three conditions (i.e. acute abdominal pain, head injury, and preventive care) we were unable to identify CPGs where “a systematic review of evidence and an assessment of the benefits and harms of alternative care options” had been undertaken by the CPG developers to inform the recommendations[67, 68]. As a result, we accepted CPGs and protocols for managing care produced at state, national or local hospital level which means that there was little depth to the evidence base underpinning the indicator sets for these three conditions. Grades of recommendation and levels of evidence were recorded verbatim from included CPGs. CPGs did not consistently report sufficient information about the primary evidence or decision-making used to formulate recommendations to allow the author team to uniformly apply an established evidence grading system to extracted recommendations, such as GRADE[108]. As a reflection of the CPGs from which they were born, the majority of medical record audit indicator items were based on consensus-level recommendations and pertained to under-use (S6 Table). However, in recent years there has been growing awareness of ‘overuse’ of healthcare resources(6, 9, 76, 104, 105) and to perceive this as a source of not only waste but of healthcare related harm(11, 106). We found 45 (8%) of our indicator items sought to evaluate some aspect of over-use. A range of national standards and accreditation processes (e.g. Choosing Wisely(109)) are working to champion reduction of over-use and unwarranted healthcare variation(3, 14) and low value care(9, 15).

Second, the indicators represent the opinions of individuals who chose to participate in this study. Internal review panel members were non-randomly selected for invitation, and external reviewers were targeted through relevant medical colleges, professional associations and networks which may have skewed our sample or amplified any effects of self-selection bias. We met our goal of achieving at least nine external reviewers for only one (BRON) of the 21 conditions (S1 Table); attracting a lower critical mass of experts than expected may limit the face validity of our indicator sets when applied to the clinical setting (e.g. response bias, non-representative process measures, reduced endorsement and uptake in the wider community)[109, 110].Tempering this, our internal review panel had extensive clinical and quality improvement experience in paediatric care in Australia, and most external reviewers had university-based affiliations in addition to their clinical roles which may have assisted to refine the indicators in a manner underpinned by both scientific evidence and clinical experience. Importantly, paediatricians working in hospital settings dominated our expert panels; their review of clinical indicators geared towards capturing information about care provided in general practice may have lacked relevance. Patient and public involvement in guideline development aims to improve patient-centred health care provision, foster democratic healthcare policy-making, and enhance the quality of healthcare and related policy[111]; CTK indicators were developed without patient consultation, which is a limitation of our process.

Third, we did not formally evaluate the methodological rigor of our indicator development process with a validated quality appraisal tool, such as AIRE (Appraisal of Indicators through Research and Evaluation). While this paper reports on aspects related to the first three AIRE domains (Purpose, relevance and organizational context; Stakeholder involvement; Scientific evidence)[112], further details about the fourth AIRE domain (Additional evidence, formulation, usage) are available in the supplementary material of the results paper of the *CareTrack Kids* multistage stratified sample medical record review. Our development process did not involve reviewers meeting face to face. The use of online technologies was specifically chosen to enhance transparency, accessibility and timeliness of the development process and minimise “group-think”[69, 75]; however it could be argued that an opportunity for reviewers to meet may have stimulated useful discussion on contentious issues[97, 113]. While we did encourage experts to make comments (which were included in de-identified format with the next iterations of indicators presented to reviewers in subsequent rounds) in both Stages 3a and 3b, information from the internal review was not able to be conveyed to external reviews (due to project and wiki system constraints).

Based on our experience, and emerging standards around new approaches for evidence development[68], it is recommended that future clinical indicator developers look to further harness available technology such as wikis to help facilitate the rate at which consensus can be achieved, and to optimise its transparency (i.e. ability to capture discussion threads) and reach[11], and include patients within review panels to ensure their perspectives as key stakeholders are captured and considered[114, 115]. However, as an interim step and to address the issue of feasibility of measurement and clinical utility of indicators), qualitative research seeking insights from those who develop CPGs, indicators, and medical record software and tools[11, 69–71] as well as users (e.g. clinicians, healthcare organisations), could help to bridge the gaps between what we consider to be appropriate care, how it may be relevantly documented, and used to evaluate the quality of clinical practice.

## Conclusion

Findings from the modified Delphi approach presented in this study address recommendations for methodological rigor and transparency of reporting[17], and provide an inventory of our experiences and learnings from developing clinical indicators of appropriate care for common paediatric medical conditions. In a critical next step, these clinical indicators will form the criteria against which the *CTK* study can, for the first time in Australia, measure appropriateness of paediatric care in 2012 and 2013[64]. Our Delphi approach could be used by others to refine this suite of clinical indicators to local contexts to assist point-of-care decision-making, or providing a starting point for undertaking similar analyses of healthcare practices for benchmarking purposes.

## Supporting information

S1 Table *CareTrack Kids* final clinical indicators and items developed to assess compliance for 21 paediatric conditions. (DOCX)

S2 Table *CareTrack Kids* excluded original recommendations from included clinical practice guidelines. (DOCX)

S3 Table *CareTrack Kids* list of excluded clinical indicators. (DOCX)

S4 Table *CareTrack Kids* ‘new’ indicators by way of splitting existing indicators mapped to their corresponding final medical record indicator items. (DOCX)

S5 Table Evolution of numbers of indicators over the development process, from original recommendations to the final indicators and medical record audit indicator items, per condition. (DOCX)

S6 Table Characteristics of included medical record audit indicator items. (DOCX)

S7 Table Mapping of the CareTrack Kids approach to practical guidance for using and reporting Delphi procedures. (DOCX)
