# Supplementary material for: Clinical indicators for common paediatric conditions: Processes, provenance and products of the CareTrack Kids study
Source: PLoS One. 2019 Jan 9;14(1):e0209637. doi: 10.1371/journal.pone.0209637 (PMC6326465; doi:10.1371/journal.pone.0209637)
Supplement: S1 Table — (DOCX) [file pone.0209637.s001.docx]

**S1 Table. *CareTrack Kids* final clinical indicators and items developed to assess compliance for 21 paediatric conditions.**

The clinical indicators and items are presented by condition, with their source, the number of reviewers (external wiki review), mean score (SD) for appropriateness, level of evidence, and whether they were measured for under- or over-use.

^ N=3 indicator items (BRON05, DIAB01, DIAB02) included in final sample with mean appropriateness scores of less than seven (based on advice from Clinical Champions)

* Within conditions, the number of reviewers per indicator varied (reviewers could select an option ‘NA’ if the indicator was not applicable to their area of expertise or the clinical setting in which they worked)

U / O underuse / overuse

HCP healthcare practice

ED Emergency Department

Hosp Hospital (inpatients)

GP General Practice

Spec Specialist (e.g. general paediatrician)

|  | **Indicator** | **Item No** | **Item^** | **Number of reviewers*** | **Source** | **Mean score (appropriateness) (SD)** | **Level of evidence** | **U / O** | **Classification** | **HCP facilities** |
| --- | --- | --- | --- | --- | --- | --- | --- | --- | --- | --- |
| **ABDOMINAL PAIN** | | | | | | | | | | |
| 1 | Children presenting with acute abdominal pain have the following documented:  - Pain history e.g. onset, location, severity, progression, character AND  - characteristics of other associated features e.g. fever, cough, vomiting , pallor, lethargy, anorexia AND  - Possibility of urinary tract infection e.g. history of UTI, offensive urine, dysuria, frequency AND  - gynaecological history in teenage girls AND  - history of bowel movements e.g. stool pattern, stool quality (size, hard / soft, odour), constipation, diarrhoea AND  Past medical history e.g. surgical, medical, family, and travel. | ABDO01 | Children who presented with acute abdominal pain had their pain history documented (e.g. onset, location, severity, progression, character) | 5 | The RCH Melbourne. 2013. (1)  Macaluso C, McNamara RM. 2012.(2)  Makin E, Davenport M. 2012.(3)  Leung AKC, Sigalet DL. 2003.(4)  Cheung C. 2011.(5)  Saucier A, Huang EY, Emeremni CA, Pershad J 2014(6) | 8.8  (0.4) | Consensus-based recommendation | U | Diagnosis | ED  GP  Hosp  Spec |
|  |  | ABDO02 | Children who presented with acute abdominal pain were screened for other associated features (e.g. fever, cough, vomiting, pallor, lethargy, anorexia). | 6 | The RCH Melbourne. 2013. (1)  Macaluso C, McNamara RM. 2012.(2)  Makin E, Davenport M. 2012.(3)  Leung AKC, Sigalet DL. 2003.(4)  Cheung C. 2011.(5)  Saucier A, Huang EY, Emeremni CA, Pershad J 2014(6) | 8.7  (0.8) | Consensus-based recommendation | U | Diagnosis | ED  GP  Hosp  Spec |
|  |  | ABDO03 | Children who presented with acute abdominal pain were assessed for possible urinary tract infection (e.g. offensive urine, dysuria, frequency). | 6 | The RCH Melbourne. 2013. (1)  Macaluso C, McNamara RM. 2012.(2)  Makin E, Davenport M. 2012.(3)  Leung AKC, Sigalet DL. 2003.(4)  Cheung C. 2011.(5)  Saucier A, Huang EY, Emeremni CA, Pershad J 2014(6) | 8.2  (1.0) | Consensus-based recommendation | U | Diagnosis | ED  GP  Hosp  Spec |
|  |  | ABDO04 | Children who presented with acute abdominal pain had their gynaecological history documented. | 6 | The RCH Melbourne. 2013. (1)  Macaluso C, McNamara RM. 2012.(2)  Makin E, Davenport M. 2012.(3)  Leung AKC, Sigalet DL. 2003.(4)  Cheung C. 2011.(5)  Saucier A, Huang EY, Emeremni CA, Pershad J 2014 (6) | 8.5  (0.8) | Consensus-based recommendation | U | Diagnosis | ED  GP  Hosp  Spec |
|  |  | ABDO05 | Children who presented with acute abdominal pain had their history of bowel movements documented (e.g. stool pattern, stool quality [size, hard / soft, odour], constipation, and diarrhoea). | 6 | The RCH Melbourne. 2013. (1)  Macaluso C, McNamara RM. 2012.(2)  Makin E, Davenport M. 2012.(3)  Leung AKC, Sigalet DL. 2003.(4)  Cheung C. 2011.(5)  Saucier A, Huang EY, Emeremni CA, Pershad J 2014(6) | 8.3  (0.8) | Consensus-based recommendation | U | Diagnosis | ED  GP  Hosp  Spec |
|  |  | ABDO06 | Children who presented with acute abdominal pain had their past medical history documented (e.g. surgical, medical, family, and travel). | 6 | The RCH Melbourne. 2013.(1)  Macaluso C, McNamara RM. 2012.(2)  Makin E, Davenport M. 2012.(3)  Leung AKC, Sigalet DL. 2003.(4)  Cheung C. 2011.(5)  Saucier A, Huang EY, Emeremni CA, Pershad J 2014(6) | 7.7  (1.8) | Consensus-based recommendation | U | Diagnosis | ED  GP  Hosp  Spec |
| 2 | Children presenting with acute abdominal pain have the following documented:  - vital signs including heart rate and temperature AND  - severity of dehydration e.g. absent, mild, moderate or severe dehydration AND  - abdominal assessment for tenderness e.g. local or generalised tenderness AND  - abdominal assessment for signs of acute abdomen e.g. rebound, guarding or rigidity AND  other abdominal findings e.g. masses, distention, palpable faeces, bowel sounds AND  - assessment of inguinoscrotal area in boys, e.g. swelling or tenderness. | ABDO07 | Children who presented with acute abdominal pain had their vital signs (including heart rate and temperature) documented. | 5 | The RCH Melbourne. 2013. (1)  Macaluso C, McNamara RM. 2012.(2)  Leung AKC, Sigalet DL. 2003.(4)  Cheung C. 2011.(5)  Saucier A, Huang EY, Emeremni CA, Pershad J 2014(6) | 8.2  (1.3) | Consensus-based recommendation | U | Diagnosis | ED  GP  Hosp  Spec |
|  |  | ABDO08 | Children who presented with acute abdominal pain had the severity of their dehydration (e.g. absent, mild, moderate or severe dehydration) documented. | 5 | The RCH Melbourne. 2013. (1)  Macaluso C, McNamara RM. 2012.(2)  Leung AKC, Sigalet DL. 2003.(4)  Cheung C. 2011.(5)  Saucier A, Huang EY, Emeremni CA, Pershad J 2014(6) | 8  (1.4) | Consensus-based recommendation | U | Diagnosis | ED  GP  Hosp  Spec |
|  |  | ABDO09 | Children who presented with acute abdominal pain received an abdominal assessment for tenderness (e.g. local or generalised tenderness). | 5 | The RCH Melbourne. 2013.(1)  Macaluso C, McNamara RM. 2012.(2)  Leung AKC, Sigalet DL. 2003.(4)  Cheung C. 2011.(5)  Saucier A, Huang EY, Emeremni CA, Pershad J 2014(6) | 8.8  (0.4) | Consensus-based recommendation | U | Diagnosis | ED  GP  Hosp  Spec |
|  |  | ABDO10 | Children who presented with acute abdominal pain received an abdominal assessment for signs of acute abdomen (e.g. rebound, guarding or rigidity.) | 5 | The RCH Melbourne. 2013. (1)  Macaluso C, McNamara RM. 2012.(2)  Leung AKC, Sigalet DL. 2003.(4)  Cheung C. 2011.(5)  Saucier A, Huang EY, Emeremni CA, Pershad J 2014(6) | 8.6  (0.5) | Consensus-based recommendation | U | Diagnosis | ED  GP  Hosp  Spec |
|  |  | ABDO11 | Children who presented with acute abdominal pain had other abdominal findings (e.g. masses, distention, palpable faeces, bowel sounds) documented. | 5 | The RCH Melbourne. 2013. (1)  Macaluso C, McNamara RM. 2012.(2)  Leung AKC, Sigalet DL. 2003.(4)  Cheung C. 2011.(5)  Saucier A, Huang EY, Emeremni CA, Pershad J 2014(6) | 8.8  (0.4) | Consensus-based recommendation | U | Diagnosis | ED  GP  Hosp  Spec |
|  |  | ABDO12 | Children who presented with acute abdominal pain received an assessment of their inguinoscrotal area (e.g. swelling or tenderness). | 5 | The RCH Melbourne. 2013.(1)  Macaluso C, McNamara RM. 2012.(2)  Leung AKC, Sigalet DL. 2003.(4)  Cheung C. 2011.(5)  Saucier A, Huang EY, Emeremni CA, Pershad J 2014(6) | 8.6  (0.5) | Consensus-based recommendation | U | Diagnosis | ED  GP  Hosp  Spec |
| 3 | All children presenting with non-traumatic acute abdominal pain do NOT receive an abdominal x-ray or CT scan, with the exception of the following circumstances:  - to exclude a differential diagnosis of acute obstruction or perforation OR"  - bile (yellow or green) stained vomit OR  - suspected ingestion of radiopaque foreign objects. | ABDO13 | Children who presented with non-traumatic acute abdominal pain who do not require exclusion of a differential diagnosis of acute obstruction or perforation, did not receive an abdominal x-ray or CT scan. | 5 | Holland AJA. 2009(7) | 8.6  (0.5) | Consensus-based recommendation | O | Diagnosis | ED  GP  Hosp  Spec |
|  |  | ABDO14 | Children who presented with non-traumatic acute abdominal pain, and NO bile (yellow or green) stained vomit, did not receive an abdominal x-ray or CT scan. | 5 | Holland AJA. 2009(7) | 8.8  (0.4) | Consensus-based recommendation | O | Diagnosis | ED  GP  Hosp  Spec |
|  |  | ABDO15 | Children who presented with non-traumatic acute abdominal pain, and NO suspected ingestion of radiopaque foreign objects, did not receive an abdominal x-ray or CT scan. | 5 | Holland AJA. 2009(7) | 7.6  (2.6) | Consensus-based recommendation | O | Diagnosis | ED  GP  Hosp  Spec |
| 4 | Children presenting with acute abdominal pain classified as severe pain are administered IV morphine or intranasal fentanyl. | ABDO16 | Children who presented with acute severe abdominal pain were administered IV morphine or intranasal fentanyl. | 5 | Makin E, Davenport M. 2012.(3)  Leung AKC, Sigalet DL. 2003.(4)  NSW Kids and Families. 2005.(8) | 7.8  (1.4) | Grade A | U | Treatment | ED hosp |
| 5 | Children presenting with acute abdominal pain classified as moderate pain are administered oxycodone along with paracetamol or ibuprofen. | ABDO17 | Children who presented with acute mild abdominal pain, who require analgesia, were administered paracetamol or ibuprofen. | 5 | Makin E, Davenport M. 2012.(3)  Leung AKC, Sigalet DL. 2003.(4)  NSW Kids and Families. 2005.(8) | 7  (1) | Grade A | U | Treatment | ED  GP  Hosp  Spec |
| 6 | Children presenting with acute abdominal pain classified as mild pain are administered paracetamol or ibuprofen if analgesia is given. | ABDO18 | Children who presented with acute abdominal pain who were moderately dehydrated had their blood sugar measured. | 5 | The RCH Melbourne. 2013.(1) | 8  (1.4) | Consensus-based recommendation | U | Treatment | ED  GP  Hosp  Spec |
| 7 | Children presenting with acute abdominal pain who are classified as severely dehydrated OR shocked are managed as follows:  - electrolytes measured AND  - blood sugar measured AND  - fluid resuscitation (initial bolus 20ml/kg normal saline). | ABDO19 | Children who presented with acute abdominal pain who were severely dehydrated OR shocked, had their electrolytes measured. | 5 | The RCH Melbourne. 2013. (1) | 8.2  (1.3) | Consensus-based recommendation | U | Treatment | ED  GP  Hosp  Spec |
|  |  | ABDO20 | Children who presented with acute abdominal pain who were severely dehydrated OR shocked, had their blood sugar measured. | 5 | The RCH Melbourne. 2013.(1) | 8.2  (1.3) | Consensus-based recommendation | U | Treatment | ED  GP  Hosp  Spec |
|  |  | ABDO21 | Children who presented with acute abdominal pain who were severely dehydrated OR shocked, receive fluid resuscitation (initial bolus 20ml/kg normal saline). | 5 | The RCH Melbourne. 2013. (1) | 8.8  (0.4) | Consensus-based recommendation | U | Treatment | ED hosp |
| **ACUTE GASTROENTERITIS** | | | | | | | | | | |
| 1 | Children presenting with gastroenteritis have the following history recorded:  - fluid intake AND  - urine output AND  - frequency of vomiting and diarrhoea AND  - duration of illness. | AGE01 | Children who presented with gastroenteritis had their fluid intake recorded. | 3 | NSW Kids and Families. 2010.(9)  Guarino A, Albano F, Ashkenazi S, et al. 2008(10)  Cincinnati Children's hospital medical center. 2011.(11) | 8.7  (0.6) | Consensus-based recommendation | U | Diagnosis | ED  GP  Hosp |
|  |  | AGE02 | Children who presented with gastroenteritis had their urine output recorded. | 3 | NSW Kids and Families. 2010.(9)  Guarino A, Albano F, Ashkenazi S, et al. 2008 (10)  Cincinnati Children's hospital medical center. 2011.(11) | 8.7  (0.6)) | Consensus-based recommendation | U | Diagnosis | ED  GP  Hosp |
|  |  | AGE03 | Children who presented with gastroenteritis had the frequency of their vomiting and diarrhoea recorded. | 3 | NSW Kids and Families. 2010.(9)  Guarino A, Albano F, Ashkenazi S, et al. 2008(10)  Cincinnati Children's hospital medical center. 2011.(11) | 8.7  (0.6) | Consensus-based recommendation | U | Diagnosis | ED  GP  Hosp |
|  |  | AGE04 | Children who presented with gastroenteritis had the duration of their illness recorded. | 3 | NSW Kids and Families. 2010.(9)  Guarino A, Albano F, Ashkenazi S, et al. 2008(10)  Cincinnati Children's hospital medical center. 2011.(11) | 8.7  (0.6) | Consensus-based recommendation | U | Diagnosis | ED  GP  Hosp |
| 2 | Children presenting with gastroenteritis have their weight recorded. | AGE05 | Children who presented with gastroenteritis had their weight recorded. | 3 | NSW Kids and Families. 2010.(9)  Guarino A, Albano F, Ashkenazi S, et al. 2008(10)  Cincinnati Children's hospital medical center. 2011.(11) | 7.7  (2.3) | Consensus-based recommendation | U | Diagnosis | ED  GP  Hosp |
| 3 | Children presenting with gastroenteritis have the following assessed:  - lethargy AND  - mucous membranes AND  - depressed fontanelle in babies (age 0-12 months) AND  - Observations - Temp, Heart Rate, Resp, BP. | AGE06 | Children who presented with gastroenteritis were assessed for lethargy. | 3 | NSW Kids and Families. 2010.(9)  Guarino A, Albano F, Ashkenazi S, et al. 2008(10)  Cincinnati Children's hospital medical center. 2011.(11) | 8  (1.7) | Consensus-based recommendation | U | Diagnosis | ED  GP  Hosp |
|  |  | AGE07 | Children who presented with gastroenteritis had their mucous membranes assessed. | 3 | NSW Kids and Families. 2010.(9)  Guarino A, Albano F, Ashkenazi S, et al. 2008(10)  Cincinnati Children's hospital medical center. 2011.(11) | 7.7  (2.3) | Consensus-based recommendation | U | Diagnosis | ED  GP  Hosp |
|  |  | AGE08 | Babies (aged <12 months) who presented with gastroenteritis had their fontanelle assessed. | 3 | NSW Kids and Families. 2010.(9)  Guarino A, Albano F, Ashkenazi S, et al. 2008(10)  Cincinnati Children's hospital medical center. 2011.(11) | 7.3  (2.9) | Consensus-based recommendation | U | Diagnosis | ED  GP  Hosp |
|  |  | AGE09 | Children who presented with gastroenteritis had their observations (Temp, Heart Rate, Resp, BP) assessed. | 3 | NSW Kids and Families. 2010.(9)  Guarino A, Albano F, Ashkenazi S, et al. 2008(10)  Cincinnati Children's hospital medical center. 2011.(11) | 7.3  (2.9) | Consensus-based recommendation | U | Diagnosis | ED  GP  Hosp |
| 4 | Children presenting with gastroenteritis are assessed to determine the degree of dehydration. | AGE10 | Children who presented with gastroenteritis had their degree of dehydration assessed. | 3 | NSW Kids and Families. 2010.(9)  Guarino A, Albano F, Ashkenazi S, et al. 2008(10)  Cincinnati Children's hospital medical center. 2011.(11) | 9  (0) | Consensus-based recommendation | U | Diagnosis | ED  GP  Hosp |
| 5 | Children with gastroenteritis presenting to the ED with any of the following receive electrolytes and venous blood gas:  - requiring intravenous therapy (IVT) OR  - severe dehydration OR  - altered conscious state / convulsions OR  - pre-existing medical conditions that predispose to electrolyte abnormalities e.g. cystic fibrosis, renal impairment, diabetes. | AGE11 | Children who presented to the ED with gastroenteritis and required intravenous therapy (IVT), received electrolytes. | 3 | Cincinnati Children's hospital medical center. 2011(11)  Heinz P. 2008(12) | 8.3  (1.2) | Consensus-based recommendation | U | Treatment | ED |
|  |  | AGE12 | Children who presented to the ED with gastroenteritis and required intravenous therapy (IVT), received a venous blood gas. | 3 | Cincinnati Children's hospital medical center. 2011(11)  Heinz P. 2008(12) | 8.3  (1.2) | Consensus-based recommendation | U | Treatment | ED |
|  |  | AGE13 | Children who presented to the ED with gastroenteritis and severe dehydration, received electrolytes. | 3 | Cincinnati Children's hospital medical center. 2011(11)  Heinz P. 2008(12) | 8.7  (0.6) | Consensus-based recommendation | U | Treatment | ED |
|  |  | AGE14 | Children who presented to the ED with gastroenteritis and severe dehydration, received a venous blood gas. | 3 | Cincinnati Children's hospital medical center. 2011(11)  Heinz P. 2008(12) | 8.7  (0.6) | Consensus-based recommendation | U | Treatment | ED |
|  |  | AGE15 | Children who presented to the ED with gastroenteritis and altered conscious state / convulsions received electrolytes. | 3 | Cincinnati Children's hospital medical center. 2011(11)  Heinz P. 2008(12) | 9  (0) | Consensus-based recommendation | U | Treatment | ED |
|  |  | AGE16 | Children who presented to the ED with gastroenteritis and altered conscious state / convulsions received a venous blood gas. | 3 | Cincinnati Children's hospital medical center. 2011(11)  Heinz P. 2008(12) | 9  (0) | Consensus-based recommendation | U | Treatment | ED |
|  |  | AGE17 | Children who presented to the ED with gastroenteritis and pre-existing medical conditions that predispose to electrolyte abnormalities (e.g. cystic fibrosis, renal impairment, diabetes), received electrolytes. | 3 | Cincinnati Children's hospital medical center. 2011(11)  Heinz P. 2008(12) | 8.7  (0.6) | Consensus-based recommendation | U | Treatment | ED |
|  |  | AGE18 | Children who presented to the ED with gastroenteritis and pre-existing medical conditions that predispose to electrolyte abnormalities (e.g. cystic fibrosis, renal impairment, diabetes), received a venous blood gas. | 3 | Cincinnati Children's hospital medical center. 2011(11)  2011.Heinz P. 2008(12) | 8.7  (0.6) | Consensus-based recommendation | U | Treatment | ED |
| 6 | Children with gastroenteritis do NOT receive routine blood tests unless there are signs and symptoms of dehydration. | AGE19 | Children with gastroenteritis and NO signs and symptoms of dehydration, did not receive routine blood tests. | 3 | Cincinnati Children's hospital medical center. 2011.(11) | 9  (0) | Consensus-based recommendation | O | Treatment | ED  GP  Hosp |
| 7 | Children with gastroenteritis without signs of infection are NOT prescribed:  - anti-diarrhoeals (such as loperimide, kaolin), OR  - antiemetics other than single dose ondansetron (not maxalon, stemetil, multi-dose ondansetron) OR  - antibiotics. | AGE20 | Children with gastroenteritis and no signs of infection were not prescribed anti-diarrhoeals (such as loperimide, kaolin). | 3 | Cincinnati Children's hospital medical center. 2011.(11)  The Royal Children's Hospital Melbourne. 2013. (13)  Farthing M, Lindberg G, Dite P, et al. 2008.(14) | 9  (0) | Consensus-based recommendation | O | Treatment | ED  GP  Hosp |
|  |  | AGE21 | Children with gastroenteritis and no signs of infection were not prescribed maxalon, stemetil, multi-dose ondansetron. | 2 | Cincinnati Children's hospital medical center. 2011.(11)  The Royal Children's Hospital Melbourne. 2013. (13)  Farthing M, Lindberg G, Dite P, et al. 2008.(14) | 9  (0) | Consensus-based recommendation | O | Treatment | ED  GP  Hosp |
|  |  | AGE22 | Children with gastroenteritis and no signs of infection were not prescribed antibiotics. | 3 | Cincinnati Children's hospital medical center. 2011.(11)  The Royal Children's Hospital Melbourne. 2013. (13)  Farthing M, Lindberg G, Dite P, et al. 2008.(14) | 9  (0) | Consensus-based recommendation | O | Treatment | ED  GP  Hosp |
| 8 | Children presenting with gastroenteritis who are severely dehydrated receive IV fluid rehydration including a 20ml/kg bolus. | AGE23 | Children who presented with gastroenteritis and were severely dehydrated, received IV fluid rehydration including a 20ml/kg bolus. | 3 | Cincinnati Children's hospital medical center. 2011.(11) | 9  (0) | Consensus-based recommendation | U | Treatment | ED Hosp |
| 9 | Children presenting with gastroenteritis who have no or mild signs of dehydration and are able to tolerate oral fluids are:  - discharged from hospital AND  - advised to re-present if symptoms are unchanged or worsen, AND  - advised to continue with usual diet, AND/OR  - provided with information on age-appropriate oral fluid replacement (small fluids often; breastfeeding / formula, oral rehydration solution or dilute clear fluids). | AGE24 | Children who presented with gastroenteritis, had no or mild signs of dehydration, and were able to tolerate oral fluids were discharged from hospital. | 3 | NSW Kids and Families. 2010.(9)  Sydney Children's Hospital. 2004.(15)  National Institute for Health and Care Excellence (NICE). 2009.(16)  Churgay CA, Aftab Z. 2012.(17)  Women's and Children's Health Network. 2010.(18)  Kelly A, Cheong E. 2007.(19)  Cincinnati Children's hospital medical center. 2011.(11) | 8.7  (0.6) | Consensus-based recommendation | U | Ongoing management | ED Hosp |
|  |  | AGE25 | Children who presented with gastroenteritis, had no or mild signs of dehydration, and were able to tolerate oral fluids were advised to re-present if symptoms are unchanged or worsen. | 3 | NSW Kids and Families. 2010.(9)  Sydney Children's Hospital. 2004.(15)  National Institute for Health and Care Excellence (NICE). 2009.(16)  Churgay CA, Aftab Z. 2012.(17)  Women's and Children's Health Network. 2010.(18)  Kelly A, Cheong E. 2007.  Cincinnati Children's hospital medical center. 2011.(11) | 8.5  (0.7) | Consensus-based recommendation | U | Ongoing management | ED  GP  Hosp |
|  |  | AGE26 | Children who presented with gastroenteritis, had no or mild signs of dehydration, and were able to tolerate oral fluids were advised to continue with usual diet. | 3 | NSW Kids and Families. 2010.(9)  Sydney Children's Hospital. 2004.(15)  National Institute for Health and Care Excellence (NICE). 2009.(16)  Churgay CA, Aftab Z. 2012.(17)  Women's and Children's Health Network. 2010.(18)  Kelly A, Cheong E. 2007.(19)  Cincinnati Children's hospital medical center. 2011.(11) | 7  (1.4) | Consensus-based recommendation | U | Ongoing management | ED  GP  Hosp |
|  |  | AGE27 | Children who presented with gastroenteritis, had no or mild signs of dehydration, and were able to tolerate oral fluids were provided with information on age-appropriate oral fluid replacement (small fluids often; breastfeeding / formula, oral rehydration solution or dilute clear fluids). | 3 | NSW Kids and Families. 2010.(9)  Sydney Children's Hospital. 2004.(15)  National Institute for Health and Care Excellence (NICE). 2009.(16)  Churgay CA, Aftab Z. 2012.(17)  Women's and Children's Health Network. 2010.(18)  Kelly A, Cheong E. 2007.(19)  Cincinnati Children's hospital medical center. 2011.(11) | 8.7  (0.6) | Consensus-based recommendation | U | Ongoing management | ED  GP  Hosp |
| 10 | Children presenting to the GP with gastroenteritis who have any of the following are referred to hospital or the ED:  - moderate or severe dehydration. | AGE28 | Children who presented to the GP with gastroenteritis and moderate or severe dehydration were referred to hospital or the ED. | 3 | NSW Kids and Families. 2010.(9) | 8.7  (0.6) | Consensus-based recommendation | U | Ongoing management | GP |
| 11 | Children with gastroenteritis who are moderately to severely dehydrated AND have received rehydration are clinically reassessed within 6hrs for the following:  - weight AND  - clinical signs of dehydration AND  - urine output AND  - ongoing diarrhoea / vomiting AND  - signs of fluid overload (puffy face and extremities). | AGE29 | Children who presented with gastroenteritis, were moderately to severely dehydrated AND received rehydration, had their weight reassessed within 6 hours. | 3 | The Royal Children's Hospital Melbourne. 2013.(13) | 7  (2.8) | Consensus-based recommendation | U | Ongoing management | ED Hosp |
|  |  | AGE30 | Children who presented with gastroenteritis, were moderately to severely dehydrated AND received rehydration, were reassessed for clinical signs of dehydration within 6 hours. | 3 | The Royal Children's Hospital Melbourne. 2013.(13) | 8.7  (0.6) | Consensus-based recommendation | U | Ongoing management | ED Hosp |
|  |  | AGE31 | Children who presented with gastroenteritis, were moderately to severely dehydrated AND received rehydration, had their urine output reassessed within 6 hours. | 3 | The Royal Children's Hospital Melbourne. 2013.(13) | 8  (1.7) | Consensus-based recommendation | U | Ongoing management | ED Hosp |
|  |  | AGE32 | Children who presented with gastroenteritis, were moderately to severely dehydrated AND received rehydration, were reassessed for ongoing diarrhoea / vomiting within 6 hours. | 3 | The Royal Children's Hospital Melbourne. 2013.(13) | 8.7  (0.6) | Consensus-based recommendation | U | Ongoing management | ED Hosp |
|  |  | AGE33 | Children who presented with gastroenteritis, were moderately to severely dehydrated AND received rehydration, were reassessed for signs of fluid overload (puffy face and extremities) within 6 hours. | 3 | The Royal Children's Hospital Melbourne. 2013.(13) | 7.7  (2.3) | Consensus-based recommendation | U | Ongoing management | ED Hosp |
| 12 | Children with gastroenteritis and the following are discharged (even if there is some vomiting):  - sufficient rehydration achieved as indicated by weight gain and/or clinical status (child is rehydrated or only mildly dehydrated) AND"  - gastrointestinal loss is not profuse (oral intake equals or exceeds losses) AND | AGE34 | Children with gastroenteritis who were sufficiently rehydrated as indicated by weight gain and/or clinical status (child is rehydrated or only mildly dehydrated) were discharged. | 3 | NSW Kids and Families. 2010.(9)  Cincinnati Children's hospital medical center. 2011.(11) | 8.7  (0.6) | Consensus-based recommendation | U | Ongoing management | ED Hosp |
|  |  | AGE35 | Children with gastroenteritis who had gastrointestinal loss that was not profuse (oral intake equals or exceeds losses), were discharged. | 3 | NSW Kids and Families. 2010.(9)  Cincinnati Children's hospital medical center. 2011.(11) | 8  (1.7) | Consensus-based recommendation | U | Ongoing management | ED Hosp |
| **ATTENTION DEFICIT HYPERACTIVITY DISORDER** | | | | | | | | | | |
| 1 | Children presenting to a GP with symptoms of ADHD have the following documented:  an initial assessment AND  referral to a specialist clinician AND  provided with information on parent education and training programmes. | ADHD01 | Children who presented to their GP with symptoms/signs of ADHD had an initial assessment documented. | 8 | National Health and Medical Research Council.2012.(20)  American Academy of Pediatrics: Subcommittee on Attention-Deficit/Hyperactivity Disorder - Steering committee on quality improvement and management. 2011.(21) | 8.8  (0.5) | Grade B | U | Diagnosis | GP |
|  |  | ADHD02 | Children who presented to their GP with symptoms/signs of ADHD were referred to a clinical specialist. | 9 | National Health and Medical Research Council.2012.(20)  American Academy of Pediatrics: Subcommittee on Attention-Deficit/Hyperactivity Disorder - Steering committee on quality improvement and management. 2011.(21) | 7.7  (1.8) | Grade B | U | Diagnosis | GP |
|  |  | ADHD03 | Parents of children who presented to their GP with symptoms/signs of ADHD were provided educational and training program information. | 8 | National Health and Medical Research Council.2012.(20)  American Academy of Pediatrics: Subcommittee on Attention-Deficit/Hyperactivity Disorder - Steering committee on quality improvement and management. 2011.(21) | 7.3  (1.7) | Grade B | U | Diagnosis | GP |
| 2 | Children presenting to a specialist clinician with symptoms of ADHD have the following documented:  a comprehensive medical, developmental and mental health assessment of the child / adolescent AND  a psychosocial assessment of the child / adolescent and their family AND  holistic assessment of the individual's needs, family, social and educational circumstances AND  assessment for co-existing illnesses AND  assessment for other comorbid diagnosis. | ADHD04 | Children who presented to a clinical specialist with symptoms/signs of ADHD had a comprehensive medical, developmental and mental health assessment. | 8 | National Health and Medical Research Council.2012.(20) | 8.6  (1.1) | Consensus-based recommendation | U | Diagnosis | Spec |
|  |  | ADHD05 | Children who presented to a clinical specialist with symptoms/signs of ADHD had a psychosocial assessment which included their family. | 8 | National Health and Medical Research Council.2012.(20) | 8.4  (1.4) | Consensus-based recommendation | U | Diagnosis | Spec |
|  |  | ADHD06 | Children who presented to a clinical specialist with symptoms/signs of ADHD had a holistic assessment which included their needs, family, social and educational circumstances. | 8 | National Health and Medical Research Council.2012.(20) | 8.9  (0.4) | Consensus-based recommendation | U | Diagnosis | Spec |
|  |  | ADHD07 | Children who presented to a clinical specialist with symptoms/signs of ADHD were assessed for co-existing illnesses. | 8 | National Health and Medical Research Council.2012.(20) | 8.5  (1.1) | Consensus-based recommendation | U | Diagnosis | Spec |
|  |  | ADHD08 | Children who presented to a clinical specialist with symptoms/signs of ADHD were assessed for comorbid diagnosis. | 8 | National Health and Medical Research Council.2012.(20) | 8.8  (0.7) | Consensus-based recommendation | U | Diagnosis | Spec |
| 3 | Children diagnosed with ADHD (hyperactive, impulsive and inattentive behaviour) have evidence in the medical record that ALL of the following criteria (DSM-IV or ICD10 criteria) were met before a diagnosis of ADHD was made:  symptom onset in early childhood (before age twelve) AND  symptoms are maladaptive and excessive for the child / adolescent's age and developmental level AND  symptoms have persisted over time (at least six months) AND  symptoms evident in more than one setting AND  symptoms cause significant functional impairment AND  no better alternative explanation, such as another mental disorder. | ADHD09 | Children newly diagnosed with ADHD had an onset of their symptoms in early childhood (before aged 12). | 8 | National Health and Medical Research Council.2012.(20)  American Academy of Pediatrics: Subcommittee on Attention-Deficit/Hyperactivity Disorder - Steering committee on quality improvement and management. 2011.(21)  Kohn M. 2008.(22)  NICE guidelines. 2008.(23) | 7.4  (2.1) | Consensus-based recommendation | U | Diagnosis | GP Spec |
|  |  | ADHD10 | Children newly diagnosed with ADHD showed symptoms which were maladaptive and excessive for their age and developmental level. | 8 | National Health and Medical Research Council.2012.(20)  American Academy of Pediatrics: Subcommittee on Attention-Deficit/Hyperactivity Disorder - Steering committee on quality improvement and management. 2011.(21)  Kohn M. 2008.(22)  NICE guidelines. 2008.(23) | 8.8  (0.5) | Consensus-based recommendation | U | Diagnosis | GP Spec |
|  |  | ADHD11 | Children newly diagnosed with ADHD had symptoms which persisted over time (at least 6 months). | 8 | National Health and Medical Research Council.2012.(20)  American Academy of Pediatrics: Subcommittee on Attention-Deficit/Hyperactivity Disorder - Steering committee on quality improvement and management. 2011.(21)  Kohn M. 2008.(22)  NICE guidelines. 2008.(23) | 9  (0) | Consensus-based recommendation | U | Diagnosis | GP Spec |
|  |  | ADHD12 | Children newly diagnosed with ADHD had symptoms which were evident in more than one setting. | 8 | National Health and Medical Research Council.2012.(20)  American Academy of Pediatrics: Subcommittee on Attention-Deficit/Hyperactivity Disorder - Steering committee on quality improvement and management. 2011.(21)  Kohn M. 2008.(22)  NICE guidelines. 2008.(23) | 9  (0) | Consensus-based recommendation | U | Diagnosis | GP Spec |
|  |  | ADHD13 | Children newly diagnosed with ADHD had symptoms which caused significant functional impairment. | 8 | National Health and Medical Research Council.2012.(20)  American Academy of Pediatrics: Subcommittee on Attention-Deficit/Hyperactivity Disorder - Steering committee on quality improvement and management. 2011.(21)  Kohn M. 2008.(22)  NICE guidelines. 2008.(23) | 9  (0) | Consensus-based recommendation | U | Diagnosis | GP Spec |
|  |  | ADHD14 | Children were diagnosed with ADHD where there was no better alternative explanation (such as another mental disorder). | 8 | National Health and Medical Research Council.2012.(20)  American Academy of Pediatrics: Subcommittee on Attention-Deficit/Hyperactivity Disorder - Steering committee on quality improvement and management. 2011.(21)  Kohn M. 2008.(22)  NICE guidelines. 2008.(23) | 8.6  (1.1) | Consensus-based recommendation | U | Diagnosis | GP Spec |
| 4 | Children with ADHD have evidence that their level of impairment was assessed by gathering information from multiple sources (child / adolescent, parent, carers, teachers, health professionals). | ADHD15 | Children with ADHD had their level of impairment assessed by gathering information from multiple sources. | 8 | National Health and Medical Research Council.2012.(20)  NICE guidelines. 2008.(23) | 8.5  (0.9) | Consensus-based recommendation | U | Diagnosis | GP Spec |
| 5 | Children with ADHD receive psychological, pharmacological or educational interventions used alone or in combination. | ADHD16 | Children with ADHD received psychological, pharmacological or educational interventions. | 8 | National Health and Medical Research Council.2012.(20)  Kohn M. 2008.(22)  NICE guidelines. 2008.(23) | 8.8  (0.7) | Consensus-based recommendation | U | Treatment | GP Spec |
| 6 | Parents of children being managed for ADHD were provided with the following:  information on the diagnosis and management plan AND  any potential adverse effects of treatment. | ADHD17 | Parents of children with ADHD were provided with information on the diagnosis and management plan | 7 | National Health and Medical Research Council.2012.(20)  Kohn M. 2008.(22)  NICE guidelines. 2008.(23) | 8.6  (0.8) | Consensus-based recommendation | U | Treatment | GP Spec |
|  |  | ADHD18 | Parents of children with ADHD were advised of the potential for adverse effects of the treatment. | 7 | National Health and Medical Research Council.2012.(20)  Kohn M. 2008.(22)  NICE guidelines. 2008.(23) | 8.9  (0.4) | Consensus-based recommendation | U | Treatment | GP Spec |
| 7 | Children with ADHD who are prescribed medication had stimulant medications (methylphenidate and dexamphetamine sulphate) prescribed as first line treatment. | ADHD19 | Children with ADHD requiring medication were first prescribed a stimulant medication. | 6 | NICE guidelines. 2008.(23) | 7.5  (1.8) | Grade A | U | Treatment | GP Spec |
| 8 | Children with ADHD have the following documented before prescribing stimulant medications (methylphenidate and dexamphetamine sulphate):  baseline physical assessment data, including, as a minimum, pulse, blood pressure, weight and height AND  If there are any abnormal symptoms, findings or history regarding cardiovascular status, appropriate investigation, a referral to a cardiologist should be organised AND  potential harms, allergies, adverse effects and contraindications, including diversion of medications for misuse and abuse AND  duration of treatment and signals to stop treatment AND  schedule for follow-up, monitoring and review. | ADHD20 | Children with ADHD prescribed stimulant medication (methylphenidate and dexamphetamine sulphate) received a baseline physical assessment, including as a minimum pulse, blood pressure, weight and height prior to prescription. | 6 | National Health and Medical Research Council.2012.(20)  NICE guidelines. 2008.(23) | 9  (0) | Consensus-based recommendation | U | Treatment | GP Spec |
|  |  | ADHD21 | Children with ADHD prescribed stimulant medication (methylphenidate and dexamphetamine sulphate) and with abnormal cardiovascular symptoms, findings or history, were referred to a cardiologist prior to prescription. | 6 | National Health and Medical Research Council.2012.(20)  NICE guidelines. 2008.(23) | 7.7  (1.5) | Consensus-based recommendation | U | Treatment | GP Spec |
|  |  | ADHD22 | Children with ADHD prescribed stimulant medication (methylphenidate and dexamphetamine sulphate) had potential harms, allergies, adverse effects and contraindications, including diversion of medications for misuse and abuse documented, prior to prescription. | 6 | National Health and Medical Research Council.2012.(20)  NICE guidelines. 2008.(23) | 8.3  (1.2) | Consensus-based recommendation | U | Treatment | GP Spec |
|  |  | ADHD23 | Children with ADHD prescribed stimulant medication (methylphenidate and dexamphetamine sulphate) had the treatment duration and signals for stopping documented prior to prescription. | 6 | National Health and Medical Research Council.2012.(20)  NICE guidelines. 2008.(23) | 7.5  (1.2) | Consensus-based recommendation | U | Treatment | GP Spec |
|  |  | ADHD24 | Children with ADHD prescribed stimulant medication had a planned schedule (follow-up, monitoring and review) documented prior to prescription. | 6 | National Health and Medical Research Council.2012.(20)  NICE guidelines. 2008.(23) | 9  (0) | Consensus-based recommendation | U | Treatment | GP Spec |
| 9 | Children with ADHD have documented evidence of:  ongoing monitoring at each visit AND  at least 6-monthly review of their management plan has occurred AND  the management plan is relevant to their current symptoms. | ADHD25 | Children with ADHD were monitored at each visit. | 7 | National Health and Medical Research Council.2012.(20)  Kohn M. 2008.(22)  NICE guidelines. 2008.(23) | 8.3  (1.5) | Consensus-based recommendation | U | Ongoing management | GP Spec |
|  |  | ADHD26 | Children with ADHD had their management plan reviewed at least every 6 months. | 7 | National Health and Medical Research Council.2012.(20)  Kohn M. 2008.(22)  NICE guidelines. 2008.(23) | 7.6  (3.0) | Consensus-based recommendation | U | Ongoing management | GP Spec |
|  |  | ADHD27 | Children with ADHD had a management plan which was relevant to their current symptoms. | 7 | National Health and Medical Research Council.2012.(20)  Kohn M. 2008.(22)  NICE guidelines. 2008.(23) | 8.9  (0.4) | Consensus-based recommendation | U | Ongoing management | GP Spec |
| 10 | Children with ADHD prescribed stimulant medication (methylphenidate and dexamphetamine sulphate) have their medication ceased if:  there is no evidence of improvement, AND  there are unacceptable side effects. | ADHD28 | Children with ADHD and no evidence of improvement had their stimulant medication (methylphenidate and dexamphetamine sulphate) ceased. | 6 | National Health and Medical Research Council.2012.(20)  NICE guidelines. 2008.(23) | 7.3  (3.2) | Consensus-based recommendation | U | Ongoing management | GP Spec |
|  |  | ADHD29 | Children with ADHD and unacceptable side effects had their stimulant medication (methylphenidate and dexamphetamine sulphate) ceased. | 6 | National Health and Medical Research Council.2012.(20)  NICE guidelines. 2008.(23) | 9 (0) | Consensus-based recommendation | U | Ongoing management | GP Spec |
| 11 | Children with ADHD who have been prescribed stimulant medication (methylphenidate and dexamphetamine sulphate) have a clinical assessment and review every 3-6 months that includes:  assessment of side effects, particularly psychological symptoms AND  plotting of growth parameters AND  measurement of heart rate AND  measurement of blood pressure. | ADHD30 | Children with ADHD prescribed stimulant medication (methylphenidate and dexamphetamine sulphate) had their psychological symptoms and side effects assessed every 6 months. | 6 | National Health and Medical Research Council.2012.(20)  Kohn M. 2008.(22)  NICE guidelines. 2008.(23) | 7.4  (3.6) | Consensus-based recommendation | U | Ongoing management | GP Spec |
|  |  | ADHD31 | Children with ADHD prescribed stimulant medication (methylphenidate and dexamphetamine sulphate) had their growth parameters recorded every 6 months. | 6 | National Health and Medical Research Council.2012.(20)  Kohn M. 2008.(22)  NICE guidelines. 2008.(23) | 8.5  (0.8) | Consensus-based recommendation | U | Ongoing management | GP Spec |
|  |  | ADHD32 | Children with ADHD prescribed stimulant medication (methylphenidate and dexamphetamine sulphate) had their heart rate measured every 6 months. | 6 | National Health and Medical Research Council.2012.(20)  Kohn M. 2008.(22)  NICE guidelines. 2008.(23) | 7.7  (1.5) | Consensus-based recommendation | U | Ongoing management | GP Spec |
|  |  | ADHD33 | Children with ADHD prescribed stimulant medication (methylphenidate and dexamphetamine sulphate) had their blood pressure measured every 6 months. | 6 | National Health and Medical Research Council.2012.(20)  Kohn M. 2008.(22)  NICE guidelines. 2008.(23) | 7.5  (1.8) | Consensus-based recommendation | U | Ongoing management | GP Spec |
| 12 | Children aged less than 7 years, with ADHD who have been prescribed stimulant medication (methylphenidate and dexamphetamine sulphate) are:  assessed for adverse effects (including BP, height, weight monitored). | ADHD34 | Children aged < 7 years, with ADHD prescribed stimulant medication (methylphenidate and dexamphetamine sulphate) were assessed for adverse effects (BP, height and weight). | 6 | National Health and Medical Research Council.2012.(20)  Kohn M. 2008.(22)  NICE guidelines. 2008.(23) | 8.8  (0.4) | Consensus-based recommendation | U | Ongoing management | GP Spec |
| **ANXIETY** | | | | | | | | | | |
| 1 | Children who present with suspected anxiety receive an assessment which includes:  appraisal of family circumstances AND  Social functioning  analysis of anxious behaviour (demonstrated by one or more of: behavioural analysis, detailed documentation of an episode, phenomenology of anxiety experience) AND  Co morbid depression  impact on functioning AND  exclusion of other causes (e.g. physical illness, medication or illicit drug effect). | ANXI01 | Children who presented with suspected anxiety had their family circumstances assessed. | 3 | Royal Children's Hospital Melbourne. 2009.(24)  Sakolsky D, and Birmaher, B. 2008,(25)  British Columbia. 2010.(26)  American Academy of Child and Adolescent Psychiatry. 2007.  NICE. 2013. | 9  (0) | Consensus-based recommendation | U | Diagnosis | ED  GP  Hosp  Spec |
|  |  | ANXI02 | Children who presented with suspected anxiety had their behaviour assessed. | 3 | Royal Children's Hospital Melbourne. 2009.(24)  Sakolsky D, and Birmaher, B. 2008,(25)  British Columbia. 2010.(26)  American Academy of Child and Adolescent Psychiatry. 2007.  NICE. 2013. | 9  (0) | Consensus-based recommendation | U | Diagnosis | ED  GP  Hosp  Spec |
|  |  | ANXI03 | Children who presented with suspected anxiety had their level of functioning assessed. | 3 | Royal Children's Hospital Melbourne. 2009.(24)  Sakolsky D, and Birmaher, B. 2008,(25)  British Columbia. 2010.(26)  American Academy of Child and Adolescent Psychiatry. 2007.  NICE. 2013. | 9  (0) | Consensus-based recommendation | U | Diagnosis | ED  GP  Hosp  Spec |
|  |  | ANXI04 | Children who presented with suspected anxiety were assessed for other causes (e.g. physical illness, co-morbid depression, medication or illicit drug effect). | 3 | Royal Children's Hospital Melbourne. 2009.(24)  Sakolsky D, and Birmaher, B. 2008,(25)  British Columbia. 2010.(26)  American Academy of Child and Adolescent Psychiatry. 2007.(27)  NICE. 2013.(28) | 9  (0) | Consensus-based recommendation | U | Diagnosis | ED  GP  Hosp  Spec |
| 2 | Children with anxiety have a documented:  treatment / management plan. | ANXI05 | Children with anxiety had a documented treatment / management plan. | 3 | British Columbia. 2010.(26) | 9  (0) | Consensus-based recommendation | U | Treatment | ED  GP  Hosp  Spec |
| 3 | Children with anxiety (and their parents/carers) receive first-line management including:  psychotherapy (CB) or behavioural therapy, AND  education and support. | ANXI06 | Children with anxiety were provided psychotherapy (CBT) OR behavioural therapy as first line management. | 3 | Sakolsky D, and Birmaher, B. 2008,(25)  British Columbia. 2010.(26)  American Academy of Child and Adolescent Psychiatry. 2007.(27)  NICE. 2013. (28)  Western Australian Therapeutic Advisory Group. 2008.(29)  Canadian Psychiatric Association. 2006.(30)  Madden S. 2007.(31) | 8  (1.7) | Consensus-based recommendation | U | Treatment | ED  GP  Hosp  Spec |
|  |  | ANXI07 | Children with anxiety were provided education and support as first line management. | 3 | Sakolsky D, and Birmaher, B. 2008,(25)  British Columbia. 2010.(26)  American Academy of Child and Adolescent Psychiatry. 2007.(27)  NICE. 2013. (28)  Western Australian Therapeutic Advisory Group. 2008.(29)  Canadian Psychiatric Association. 2006.(30)  Madden S. 2007.(31) | 9  (0) | Consensus-based recommendation | U | Treatment | ED  GP  Hosp  Spec |
| 4 | Children with moderate or severe anxiety who are unable to participate in or only partially respond to psychotherapy receive selective serotonin reuptake inhibitors (SSRIs). | ANXI08 | Children with moderate / severe anxiety who were unable to participate in or only partially responded to psychotherapy were prescribed a SSRI (selective serotonin reuptake inhibitor). | 3 | Sakolsky D, and Birmaher, B. 2008,(25)  British Columbia. 2010.(26)  American Academy of Child and Adolescent Psychiatry. 2007.(27)  Madden S. 2007.(31) | 7.7  (2.3) | Consensus-based recommendation | U | Treatment | GP Spec |
| 5 | Children with anxiety who receive medication are:  monitored (adverse drug reactions, mental state and general progress) AND  parents / family are informed of risks and benefit. | ANXI09 | Children with anxiety who were prescribed medication were monitored for adverse events, their mental state and general progress. | 3 | British Columbia. 2010.(26) | 9  (0) | Consensus-based recommendation | U | Treatment | GP Spec |
|  |  | ANXI10 | Parents and family of children with anxiety who were prescribed medication were informed of the risks and benefits. | 3 | British Columbia. 2010.(26) | 9  (0) | Consensus-based recommendation | U | Treatment | GP Spec |
| 6 | Children with anxiety managed with SSRIs who have symptom remission have their medication tapered slowly. | ANXI11 | Children with anxiety who were prescribed an SSRI and had a remission of their symptoms had their medication tapered slowly. | 3 | British Columbia. 2010.(26) | 8  (1.4) | Consensus-based recommendation | U | Treatment | GP Spec |
| 7 | Children with anxiety unless under specialist review and where SSRIs have failed or are contraindicated do not receive:  Venlafaxine OR  Benzodiazepines. | ANXI12 | Children with anxiety who are not under review by a specialist AND where the SSRI had not failed / not contraindicated were not prescribed Venlafaxine. | 3 | British Columbia. 2010.(26)  Western Australian Therapeutic Advisory Group. 2008.(29)  Canadian Psychiatric Association. 2006.(30)  Madden S. 2007.(31) | 9  (0) | Consensus-based recommendation | O | Treatment | GP Spec |
|  |  | ANXI13 | Children with anxiety who are not under review by a specialist AND where the SSRI had not failed / not contraindicated were not prescribed a Benzodiazepine. | 3 | British Columbia. 2010.(26)  Western Australian Therapeutic Advisory Group. 2008.(29)  Canadian Psychiatric Association. 2006.(30)  Madden S. 2007.(31) | 9  (0) | Consensus-based recommendation | O | Treatment | GP Spec |
| **ASTHMA** | | | | | | | | | | |
| 1 | Children aged greater than 2 years with asthma who present with an acute exacerbation have the following observations recorded:  - conscious level AND  - SpO2 AND  - pulse rate AND  - assessment of work of breathing. | ASTH01 | Children aged > 2 years who presented with an acute exacerbation of asthma had their conscious level documented. | 5 | British Thoracic Society, Scottish Intercollegiate Guidelines Network. 2012.(32)  National Asthma Council Australia. 2006.(33) | 7.4  (3.0) | Consensus-based recommendation | U | Diagnosis | ED  GP  Hosp  Spec |
|  |  | ASTH02 | Children aged > 2 years who presented with an acute exacerbation of asthma had their SpO2 recorded. | 5 | British Thoracic Society, Scottish Intercollegiate Guidelines Network. 2012.(32)  National Asthma Council Australia. 2006.(33) | 8.8  (0.4) | Consensus-based recommendation | U | Diagnosis | ED  GP  Hosp  Spec |
|  |  | ASTH03 | Children aged > 2 years who presented with an acute exacerbation of asthma had their pulse rate recorded. | 5 | British Thoracic Society, Scottish Intercollegiate Guidelines Network. 2012.(32)  National Asthma Council Australia. 2006.(33) | 8.6  (0.5) | Consensus-based recommendation | U | Diagnosis | ED  GP  Hosp  Spec |
|  |  | ASTH04 | Children aged > 2 years who presented with an acute exacerbation of asthma had their work of breathing assessed. | 5 | British Thoracic Society, Scottish Intercollegiate Guidelines Network. 2012.(32)  National Asthma Council Australia. 2006.(33) | 9  (0) | Consensus-based recommendation | U | Diagnosis | ED  GP  Hosp  Spec |
| 2 | Children presenting with symptoms suggestive of a high probability of having persistent asthma are:  commenced on a trial of salbutamol, Montelukast (Singulair), cromones, or inhaled steroid (NOT Seretide) AND  reviewed within 3 months to assess response. | ASTH05 | Children who presented with symptoms which were suggestive of persistent asthma were prescribed a trial of salbutamol, Montelukast (Singulair), cromones, or inhaled steroid (NOT Seretide). | 4 | British Thoracic Society, Scottish Intercollegiate Guidelines Network. 2012.(32)  National Asthma Council Australia. 2006.(33) | 8.3  (1.5) | Level II | U | Treatment | ED  GP  Hosp  Spec |
|  |  | ASTH06 | Children who presented with symptoms which were suggestive of persistent asthma and were commenced on a trial of salbutamol, Montelukast (Singulair), cromones, or inhaled steroid (NOT Seretide) had the response assessed within 3 months. | 4 | British Thoracic Society, Scottish Intercollegiate Guidelines Network. 2012.(32)  National Asthma Council Australia. 2006.(33) | 8.5  (1) | Consensus-based recommendation | U | Treatment | ED  GP  Hosp  Spec |
| 3 | Children diagnosed with asthma who are receiving drug therapy have ALL of the following checked prior to commencing a new drug therapy:  compliance with existing therapies AND  inhaler technique checked AND  trigger factors identified / documented (URTIs, exercise, allergens) | ASTH07 | Children with asthma already prescribed medication were reviewed for compliance with existing therapies prior to commencing a new drug therapy. | 4 | British Thoracic Society, Scottish Intercollegiate Guidelines Network. 2012.(32) | 8.8  (0.5) | Consensus-based recommendation | U | Treatment | ED  GP  Hosp  Spec |
|  |  | ASTH08 | Children with asthma already prescribed medication had their inhaler technique checked prior to commencing a new drug therapy. | 4 | British Thoracic Society, Scottish Intercollegiate Guidelines Network. 2012.(32) | 9  (0) | Consensus-based recommendation | U | Treatment | ED  GP  Hosp  Spec |
|  |  | ASTH09 | Children with asthma already prescribed medication had their trigger factors documented prior to commencing a new drug therapy. | 4 | British Thoracic Society, Scottish Intercollegiate Guidelines Network. 2012.(32) | 8  (1.4) | Consensus-based recommendation | U | Treatment | ED  GP  Hosp  Spec |
| 4 | Children aged less than 2 years with asthma that present with an acute exacerbation are NOT administered oral Beta2 agonists. | ASTH10 | Children aged <2 years who presented with an acute exacerbation of asthma were not prescribed an oral beta2 agonist. | 5 | British Thoracic Society, Scottish Intercollegiate Guidelines Network. 2012.(32)  National Asthma Council Australia. 2006.(33) | 8.8  (0.4) | Consensus-based recommendation | O | Treatment | ED  GP  Hosp  Spec |
| 5 | Children aged greater than 2 years with asthma presenting with an acute exacerbation are prescribed inhaled beta2 agonists:  via a spacer if described as mild / moderate OR  oxygen driven nebuliser if life-threatening. | ASTH11 | Children aged >2 years who presented with a mild / moderate exacerbation of asthma were prescribed an inhaled beta2 agonist via a spacer. | 5 | British Thoracic Society, Scottish Intercollegiate Guidelines Network. 2012.(32)  National Asthma Council Australia. 2006.(33) | 8.8  (0.4) | Consensus-based recommendation | U | Treatment | ED  GP  Hosp  Spec |
|  |  | ASTH12 | Children aged >2 years who presented with a severe exacerbation of asthma were prescribed an inhaled beta2 agonist via an oxygen driven nebuliser. | 5 | British Thoracic Society, Scottish Intercollegiate Guidelines Network. 2012.(32)  National Asthma Council Australia. 2006.(33) | 8.3  (0.5) | Consensus-based recommendation | U | Treatment | ED  GP  Hosp  Spec |
| 6 | Children aged greater than 2 years with life threatening asthma or a SpO2 less than 95% receive supplemental oxygen. | ASTH13 | Children aged > 2 years with life threatening asthma or a SpO2 < 95% received supplemental oxygen. | 5 | British Thoracic Society, Scottish Intercollegiate Guidelines Network. 2012.(32)  National Asthma Council Australia.(33) | 7.4  (3.6) | Consensus-based recommendation | U | Treatment | ED  GP  Hosp  Spec |
| 7 | Children aged greater than 2 years with asthma who present with an acute exacerbation (and have a poor response to initial treatment) have inhaled ipratropium bromide added to their treatment. | ASTH14 | Children aged >2 years who presented with an acute exacerbation of asthma where there was no response to initial treatment were prescribed ipratropium bromide (250mcg via inhalation). | 5 | British Thoracic Society, Scottish Intercollegiate Guidelines Network. 2012.(32)  National Asthma Council Australia. 2006.(33) | 7.2  (3.0) | Consensus-based recommendation | U | Treatment | ED  GP  Hosp  Spec |
| 8 | Children aged greater than 2 years with mild to moderate asthma who present with an acute exacerbation are NOT administered aminophylline. | ASTH15 | Children aged >2 years who presented with an acute mild / moderate exacerbation of asthma were not prescribed aminophylline. | 5 | British Thoracic Society, Scottish Intercollegiate Guidelines Network. 2012.(32)  National Asthma Council Australia. 2006.(33) | 8.8  (0.4) | Consensus-based recommendation | O | Treatment | ED  GP  Hosp  Spec |
| 9 | Children aged greater than 2 years with asthma who present with an acute exacerbation are NOT prescribed antibiotics without documentation of another condition which requires antibiotic therapy. | ASTH16 | Children aged >2 years who presented with an acute exacerbation of asthma and who received antibiotics had another condition requiring antibiotic therapy. | 5 | British Thoracic Society, Scottish Intercollegiate Guidelines Network. 2012.(32)  National Asthma Council Australia. 2006.(33) | 7.6  (2.6) | Consensus-based recommendation | O | Treatment | ED  GP  Hosp  Spec |
| 10 | Children aged less than 5 years with intermittent asthma are  prescribed inhaled short acting Beta2 agonist AND  NOT prescribed an oral SABA | ASTH17 | Children aged < 5 years with intermittent asthma were prescribed short acting β2 agonist (inhaled) | 5 | British Thoracic Society, Scottish Intercollegiate Guidelines Network. 2012.(32)  National Asthma Council Australia. 2006.(33) | 8.2  (1.3) | Consensus-based recommendation | U | Treatment | ED  GP  Hosp  Spec |
|  |  | ASTH18 | Children aged < 5 years with intermittent asthma were not prescribed an oral short-acting beta agonist (SABA) | 5 | British Thoracic Society, Scottish Intercollegiate Guidelines Network. 2012.(32)  National Asthma Council Australia. 2006.(33) | 8.8  (0.4) | Consensus-based recommendation | O | Treatment | ED  GP  Hosp  Spec |
| 11 | Children aged 5-12 years with intermittent asthma are  prescribed inhaled short acting Beta2 agonists AND  NOT prescribed oral short acting Beta2 agonists | ASTH19 | Children aged 5-12 years with intermittent asthma were prescribed short acting β2 agonist (inhaled) | 5 | British Thoracic Society, Scottish Intercollegiate Guidelines Network. 2012.(32) | 9  (0) | Consensus-based recommendation | U | Treatment | ED  GP  Hosp  Spec |
|  |  | ASTH20 | Children aged 5-12 years with intermittent asthma were not prescribed oral short acting Beta2 agonists. | 5 | British Thoracic Society, Scottish Intercollegiate Guidelines Network. 2012.(32) | 8.8  (0.4) | Consensus-based recommendation | O | Treatment | ED  GP  Hosp  Spec |
| 12 | Children aged 5-12 years with frequent intermittent asthma who require regular preventer medication are prescribed:  a LTRA, or cromone, or low dose ICS (FP / BDP less than 250 mcgs / day or ciclesonide less than equals to 160mcgs / day), AND  NOT prescribed Seretide | ASTH21 | Children aged 5-12 years with frequent intermittent asthma who required regular preventer medication were prescribed a LTRA, or cromone, or low dose ICS | 5 | British Thoracic Society, Scottish Intercollegiate Guidelines Network. 2012.(32) | 8.5  (1.0) | Consensus-based recommendation | U | Treatment | ED  GP  Hosp  Spec |
|  |  | ASTH22 | Children aged 5-12 years with frequent intermittent asthma who required regular preventer medication were not prescribed Seretide | 5 | British Thoracic Society, Scottish Intercollegiate Guidelines Network. 2012.(32) | 7.8  (2.5) | Consensus-based recommendation | O | Treatment | ED  GP  Hosp  Spec |
| 13 | Children aged 5-12 years with persistent poorly controlled asthma are:  prescribed inhaled steroids (flixotide maximum dose 400mcg per day OR beclomethasone maximum dose of 800mcg per day) AND  referred to a specialist (general or respiratory paediatrician) if prescribed the maximum dose | ASTH23 | Children aged 5-12 years with persistent poorly controlled asthma were prescribed inhaled steroids. | 5 | British Thoracic Society, Scottish Intercollegiate Guidelines Network. 2012.(32)  National Asthma Council Australia. 2006.(33) | 8.8  (0.5) | Consensus-based recommendation | U | Treatment | ED  GP  Hosp  Spec |
|  |  | ASTH24 | Children aged 5-12 years with persistent poorly controlled asthma requiring the maximum dose of inhaled steroids were referred to a specialist. | 5 | British Thoracic Society, Scottish Intercollegiate Guidelines Network. 2012.(32)  National Asthma Council Australia. 2006.(33) | 8.7  (0.5) | Consensus-based recommendation | U | Treatment | ED  GP  Hosp  Spec |
| 14 | Children with asthma prescribed inhaler medication have evidence in the medical record that they:  received training on the use of the device AND  had their inhaler technique reassessed within 6 months. | ASTH25 | Children with asthma prescribed inhaler medication received training on how to use the inhaler device. | 5 | British Thoracic Society, Scottish Intercollegiate Guidelines Network. 2012.(32)  National Asthma Council Australia. 2006.(33) | 7.6  (2.1) | Consensus-based recommendation | U | Ongoing management | ED  GP  Hosp  Spec |
|  |  | ASTH26 | Children with asthma prescribed inhaler medication had their technique reassessed within 6 months | 5 | British Thoracic Society, Scottish Intercollegiate Guidelines Network. 2012.(32)  National Asthma Council Australia. 2006.(33) | 7.5  (2.4) | Consensus-based recommendation | U | Ongoing management | ED  GP  Hosp  Spec |
| 15 | Children with asthma aged less than 5 years who require regular preventer medication are commenced on:  leukotriene receptor antagonist OR  a low dose ICS (less than 200 FP equivalent) | ASTH27 | Children aged < 5 years with asthma who required regular preventer medication were prescribed a leukotriene receptor antagonist. | 5 | British Thoracic Society, Scottish Intercollegiate Guidelines Network. 2012.(32)  National Asthma Council Australia. 2006.(33) | 7.3  (3.5) | Consensus-based recommendation | U | Ongoing management | ED  GP  Hosp  Spec |
|  |  | ASTH28 | Children aged < 5 years with asthma who required regular preventer medication were prescribed a low dose inhaled corticosteroid. | 5 | British Thoracic Society, Scottish Intercollegiate Guidelines Network. 2012.(32)  National Asthma Council Australia. 2006.(33) | 8  (2.0) | Consensus-based recommendation | U | Ongoing management | ED  GP  Hosp  Spec |
| 16 | Children aged 5- 12 yrs with asthma which is not controlled with LTRA who require preventer therapy are given:  a trial of low dose ICS, OR  a low dose ICS are prescribed a trial of moderate dose ICS (and not LABA). | ASTH29 | Children aged 5- 12 yrs with asthma which was not controlled with a LTRA and who required a preventer medication were prescribed a trial of a low dose inhaled corticosteroid. | 5 | British Thoracic Society, Scottish Intercollegiate Guidelines Network. 2012.(32)  National Asthma Council Australia. 2006.(33) | 9  (0) | Consensus-based recommendation | U | Ongoing management | ED  GP  Hosp  Spec |
|  |  | ASTH30 | Children aged 5- 12 yrs with asthma which was not controlled with a low dose inhaled corticosteroid (ICS) and who required a preventer medication were prescribed a trial of a moderate dose ICS. | 5 | British Thoracic Society, Scottish Intercollegiate Guidelines Network. 2012.(32)  National Asthma Council Australia. 2006.(33) | 9  (0) | Consensus-based recommendation | U | Ongoing management | ED  GP  Hosp  Spec |
| 17 | Children aged 5-12 years with asthma who require continuous or frequent use of oral steroids are managed as follows:  prescribed a daily steroid tablet at lowest dose to provide adequate control AND  referred to a respiratory paediatrician (or general paediatrician if respiratory paediatrician not feasible due to locality). | ASTH31 | Children aged 5-12 years with asthma who required continuous or frequent use of oral steroids were prescribed a daily steroid tablet at the lowest dose. | 5 | British Thoracic Society, Scottish Intercollegiate Guidelines Network. 2012.(32) | 9  (0) | Consensus-based recommendation | U | Ongoing management | ED  GP  Hosp  Spec |
|  |  | ASTH32 | Children aged 5-12 years with asthma who required continuous or frequent use of oral steroids were referred to a respiratory paediatrician. | 5 | British Thoracic Society, Scottish Intercollegiate Guidelines Network. 2012.(32) | 9  (0) | Consensus-based recommendation | U | Ongoing management | ED  GP  Hosp  Spec |
| 18 | Children with asthma who present with an acute exacerbation in primary care are transferred to hospital urgently if they are:  aged less than 6 years and have not improved after 4-6 puffs of Beta2 agonist OR  aged greater than 6 years and have not improved after 8-12 puffs of Beta2 agonist. | ASTH33 | Children aged <6 years who presented to a primary care setting with an acute exacerbation of asthma and had not improved after 4-6 puffs of a beta2 agonist were transferred urgently to hospital. | 5 | British Thoracic Society, Scottish Intercollegiate Guidelines Network. 2012.(32)  National Asthma Council Australia. 2006.(33) | 8.8  (0.5) | Consensus-based recommendation | U | Ongoing management | GP |
|  |  | ASTH34 | Children aged >6 years who presented to a primary care setting with an acute exacerbation of asthma and had not improved after 8-12 puffs of a beta2 agonist were transferred urgently to hospital. | 5 | British Thoracic Society, Scottish Intercollegiate Guidelines Network. 2012.(32)  National Asthma Council Australia. 2006.(33) | 8.8  (0.5) | Consensus-based recommendation | U | Ongoing management | GP |
| 19 | Children with asthma on preventer medication are reviewed at least every 6 months for the following:  assessment of asthma control (symptoms, nocturnal symptoms, reliever use, physical activity limitations; exacerbations; ED / hospital admissions ) AND  assessment of potential side effects | ASTH35 | Children with asthma prescribed preventer medication had their asthma control assessed at least every 6 months | 5 | British Thoracic Society, Scottish Intercollegiate Guidelines Network. 2012.(32) | 9  (0) | Consensus-based recommendation | U | Ongoing management | ED  GP  Hosp  Spec |
|  |  | ASTH36 | Children with asthma prescribed preventer medication were assessed for side effects at least every 6 months | 5 | British Thoracic Society, Scottish Intercollegiate Guidelines Network. 2012.(32) | 9  (0) | Consensus-based recommendation | U | Ongoing management | ED  GP  Hosp  Spec |
| 20 | Children with asthma who are prescribed preventer therapy have a:  medical review at least every 6 months (medical review can be a doctor or a nurse practitioner/ asthma educator review ) AND  written asthma action plan. | ASTH37 | Children with asthma prescribed preventer therapy had a medical review at least every 6 months | 5 | British Thoracic Society, Scottish Intercollegiate Guidelines Network. 2012.(32) | 9  (0) | Consensus-based recommendation | U | Ongoing management | ED  GP  Hosp  Spec |
|  |  | ASTH38 | Children with asthma prescribed preventer therapy had a written asthma action plan. | 5 | British Thoracic Society, Scottish Intercollegiate Guidelines Network. 2012.(32) | 9  (0) | Consensus-based recommendation | U | Ongoing management | ED  GP  Hosp  Spec |
| 21 | Children discharged from hospital after an acute asthma episode have a written asthma action plan. | ASTH39 | Children discharged from hospital after an acute asthma episode had a written asthma action plan. | 5 | British Thoracic Society, Scottish Intercollegiate Guidelines Network. 2012.(32)  National Asthma Council Australia. 2013.(34) | 9  (0) | Consensus-based recommendation | U | Ongoing management | Hosp |
| **AUTISM** | | | | | | | | | | |
| 1 | Children being investigated for ASD have documented:  a comprehensive medical and family history, AND  a full physical examination, AND  parent interview. | AUTI01 | Children who were investigated for ASD had a comprehensive medical and family history documented. | 5 | Royal Australasian College of Physicians. 2008.(35)  Scottish Intercollegiate Guidelines Network. 2007.(36)  National Institute for Health and Care Excellence (NICE). 2011.(37) | 8.6  (0.9) | Grade C | U | Diagnosis | GP Spec |
|  |  | AUTI02 | Children who were investigated for ASD had a full physical examination documented. | 5 | Royal Australasian College of Physicians. 2008.(35)  Scottish Intercollegiate Guidelines Network. 2007.(36)  National Institute for Health and Care Excellence (NICE). 2011.(37) | 8.4  (1.3) | Grade C | U | Diagnosis | GP Spec |
|  |  | AUTI03 | Children who were investigated for ASD had a parent interview documented. | 5 | Royal Australasian College of Physicians. 2008.(35)  Scottish Intercollegiate Guidelines Network. 2007.(36)  National Institute for Health and Care Excellence (NICE). 2011.(37) | 9  (0) | Grade C | U | Diagnosis | GP Spec |
| 2 | Children are formally diagnosed with ASD using the diagnostic criteria of DSM IV, DSM 5 OR ICD 10. | AUTI04 | Children were diagnosed with ASD using the criteria of DSM IV, DSM 5 OR ICD 10. | 4 | Royal Australasian College of Physicians. 2008.(35)  Tonge B, Brereton A. 2011.(38)  National Institute for Health and Care Excellence (NICE). 2011.(37) | 9  (0) | Consensus-based recommendation | U | Diagnosis | GP Spec |
| 3 | Children are formally diagnosed with ASD by a paediatrician, psychiatrist or a multidisciplinary team (which may include a psychologist, a speech pathologist, or an occupational therapist). | AUTI05 | Children were formally diagnosed with ASD by a paediatrician, psychiatrist or a multidisciplinary team (which may include a psychologist, a speech pathologist, or an occupational therapist). | 4 | Tonge B, Brereton A. 2011.(38) | 7.3  (2.1) | Consensus-based recommendation | U | Diagnosis | GP Spec |
| 4 | Children being assessed for ASD have the following reviewed:  reports from educators (pre-school, child care and school as applicable) OR  their behaviour, play and communication directly observed in a natural setting. | AUTI06 | Children who were assessed for ASD had reports from educators (pre-school, child care and school as applicable) reviewed. | 4 | Royal Australasian College of Physicians. 2008.(35)  Scottish Intercollegiate Guidelines Network. 2007.(36)  Australian Government Department of Health and Ageing. 2007.(39) | 9  (0) | Grade D | U | Diagnosis | GP Spec |
|  |  | AUTI07 | Children who were assessed for ASD had their behaviour, play and communication directly observed in a natural setting reviewed. | 4 | Royal Australasian College of Physicians. 2008.(35)  Scottish Intercollegiate Guidelines Network. 2007.(36)  Australian Government Department of Health and Ageing. 2007.(39) | 9  (0) | Grade D | U | Diagnosis | GP Spec |
| 5 | Children of pre-school age being assessed for ASD have their developmental and adaptive function assessed. | AUTI08 | Children of pre-school age who were assessed for ASD had their developmental and adaptive function assessed. | 4 | Royal Australasian College of Physicians. 2008.(35) | 9  (0) | Consensus-based recommendation | U | Diagnosis | GP Spec |
| 6 | Children suspected of having ASD who have specific difficulties identified during the assessment (e.g. language delay, fine motor difficulties) receive the following:  referral to appropriate therapists (e.g. occupational therapy, speech pathology) AND  have a review appointment arranged . | AUTI09 | Children suspected of having ASD who had specific difficulties identified during the assessment (e.g. language delay, fine motor difficulties) were referred to appropriate therapists (e.g. occupational therapy, speech pathology). | 4 | Royal Australasian College of Physicians. 2008.(35)  Scottish Intercollegiate Guidelines Network. 2007.(36) | 8.8  (0.5) | Grade D | U | Treatment | GP Spec |
|  |  | AUTI10 | Children suspected of having ASD who had specific difficulties identified during the assessment (e.g. language delay, fine motor difficulties) had a review appointment arranged. | 4 | Royal Australasian College of Physicians. 2008.(35)  Scottish Intercollegiate Guidelines Network. 2007.(36) | 7.8  (1.9) | Grade D | U | Treatment | GP Spec |
| 7 | Children diagnosed with ASD have a comprehensive report including:  medical assessment AND  general development AND  developmental or psychometric assessment results AND  language and communication assessment. | AUTI11 | Children diagnosed with ASD had a comprehensive report including a medical assessment. | 4 | Royal Australasian College of Physicians. 2008.(35) | 8.3  (1.0) | Grade D | U | Treatment | GP Spec |
|  |  | AUTI12 | Children diagnosed with ASD had a comprehensive report including their general development. | 4 | Royal Australasian College of Physicians. 2008.(35) | 8.3  (1.0) | Grade D | U | Treatment | GP Spec |
|  |  | AUTI13 | Children diagnosed with ASD had a comprehensive report including developmental or psychometric assessment results. | 4 | Royal Australasian College of Physicians. 2008.(35) | 8.8  (0.5) | Grade D | U | Treatment | GP Spec |
|  |  | AUTI14 | Children diagnosed with ASD had a comprehensive report including a language and communication assessment. | 4 | Royal Australasian College of Physicians. 2008.(35) | 8.8  (0.5) | Grade D | U | Treatment | GP Spec |
| 8 | Children diagnosed with ASD have their progress and developmental and behaviour parameters reviewed at each visit. | AUTI15 | Children diagnosed with ASD had their progress and developmental and behaviour parameters reviewed at each visit. | 4 | Royal Australasian College of Physicians. 2008.(35) | 8.3  (1.5) | Consensus-based recommendation | U | Ongoing management | GP Spec |
| 9 | Children diagnosed with ASD are assessed and monitored for co-morbid disorders (e.g. epilepsy, sleep disorders, anxiety disorder, OCD, ADHD and depression). | AUTI16 | Children diagnosed with ASD were assessed and monitored for co-morbid disorders (e.g. epilepsy, sleep disorders, anxiety disorder, OCD, ADHD and depression). | 4 | Royal Australasian College of Physicians. 2008.(35)  Scottish Intercollegiate Guidelines Network. 2007.(36) | 9  (0) | Grade C | U | Ongoing management | GP Spec |
| 10 | Children diagnosed with ASD who have disruptive, aggressive and self-injurious behaviour who are prescribed risperidone are monitored for serious side effects (e.g. dystonic reactions, weight gain, metabolic disorder, behavioural deterioration). | AUTI17 | Children diagnosed with ASD who were prescribed risperidone, were monitored for serious side effects (e.g. dystonic reactions, weight gain, metabolic disorder, behavioural deterioration). | 3 | Scottish Intercollegiate Guidelines Network. 2007.(36)  Tonge B, Brereton A. 2011(38) | 9  (0) | Consensus-based recommendation | U | Ongoing management | GP Spec |
| **BRONCHIOLITIS** | | | | | | | | | | |
| 1 | Infants (aged less than 12months) presenting with acute bronchiolitis have the following history recorded:  duration and progression of symptoms AND  presence of apnoea AND  feeding AND  previous episodes of bronchiolitis, AND  family history of atopy or asthma, AND  pre-existing conditions. | BRON01 | Infants (aged less than 12months) presenting with acute bronchiolitis had the duration and progression of their symptoms recorded. | 14 | SA Child Health Clinical Network. 2013.(40)  NSW Kids and Families. 2012.(41)  The Royal Children's Hospital Melbourne. Bronchiolitis Guideline. 2012.(42)  The Royal Children's Hospital Melbourne. 2013.(43)  Sydney Children's Hospital. 2011.(44)  Zentz SE. 2011.(45)  American Adacemy of Pediatrics. 2006.(46)  Scottish Intercollegiate Guidelines Network. 2006.(47) | 8.1  (1.2) | Grade B | U | Diagnosis | ED  GP  Hosp |
|  |  | BRON02 | Infants (aged less than 12months) presenting with acute bronchiolitis had the presence of apnoea recorded. | 13 | SA Child Health Clinical Network. 2013.(40)  NSW Kids and Families. 2012.(41)  The Royal Children's Hospital Melbourne. Bronchiolitis Guideline. 2012.(42)  The Royal Children's Hospital Melbourne. 2013.(43)  Sydney Children's Hospital. 2011.(44)  Zentz SE. 2011.(45)  American Adacemy of Pediatrics. 2006.(46)  Scottish Intercollegiate Guidelines Network. 2006.(47) | 8.2  (1.3) | Grade B | U | Diagnosis | ED  GP  Hosp |
|  |  | BRON03 | Infants (aged less than 12months) presenting with acute bronchiolitis had their feeding history recorded. | 14 | SA Child Health Clinical Network. 2013.(40)  NSW Kids and Families. 2012.(41)  The Royal Children's Hospital Melbourne. Bronchiolitis Guideline. 2012.(42)  The Royal Children's Hospital Melbourne. 2013.(43)  Sydney Children's Hospital. 2011.(44)  Zentz SE. 2011.(45)  American Adacemy of Pediatrics. 2006.(46)  Scottish Intercollegiate Guidelines Network. 2006.(47) | 8.9  (0.4) | Grade B | U | Diagnosis | ED  GP  Hosp |
|  |  | BRON04 | Infants (aged less than 12months) presenting with acute bronchiolitis had the presence of previous episodes of bronchiolitis recorded. | 14 | SA Child Health Clinical Network. 2013.(40)  NSW Kids and Families. 2012.(41)  The Royal Children's Hospital Melbourne. Bronchiolitis Guideline. 2012.(42)  The Royal Children's Hospital Melbourne. 2013.(43)  Sydney Children's Hospital. 2011.(44)  Zentz SE. 2011.(45)  American Adacemy of Pediatrics. 2006.(46)  Scottish Intercollegiate Guidelines Network. 2006.(47) | 7.4  (1.4) | Grade B | U | Diagnosis | ED  GP  Hosp |
|  |  | BRON05 | Infants (aged less than 12months) presenting with acute bronchiolitis had their family history of atopy or asthma recorded. | 14 | SA Child Health Clinical Network. 2013.(40)  NSW Kids and Families. 2012.(41)  The Royal Children's Hospital Melbourne. Bronchiolitis Guideline. 2012.(42)  The Royal Children's Hospital Melbourne. 2013.(43)  Sydney Children's Hospital. 2011.(44)  Zentz SE. 2011.(45)  American Adacemy of Pediatrics. 2006.(46)  Scottish Intercollegiate Guidelines Network. 2006.(47) | 6.3  (1.6) | Grade B | U | Diagnosis | ED  GP  Hosp |
|  |  | BRON06 | Infants (aged less than 12months) presenting with acute bronchiolitis had the presence of pre-existing conditions recorded. | 14 | SA Child Health Clinical Network. 2013.(40)  NSW Kids and Families. 2012.(41)  The Royal Children's Hospital Melbourne. Bronchiolitis Guideline. 2012.(42)  The Royal Children's Hospital Melbourne. 2013.(43)  Sydney Children's Hospital. 2011.(44)  Zentz SE. 2011.(45)  American Adacemy of Pediatrics. 2006.(46)  Scottish Intercollegiate Guidelines Network. 2006.(47) | 8.4  (0.6) | Grade B | U | Diagnosis | ED  GP  Hosp |
| 2 | Infants (aged less than 12months) presenting with acute bronchiolitis have the following examined:  General appearance and basic observations (Temp, RR, HR, SaO2, colour) AND  Hydration (review of skin turgor, capillary refill, peripheral refill, mucous membranes, fontanelle) AND  Respiratory examination (work of breathing, recession, auscultation) AND  Feeding (duration and volume, saturations whilst feeding). | BRON07 | Infants (aged less than 12months) presenting with acute bronchiolitis had their general appearance and basic observations (Temp, RR, HR, SaO2) examined. | 14 | SA Child Health Clinical Network. 2013.(40)  NSW Kids and Families. 2012.(41)  The Royal Children's Hospital Melbourne. Bronchiolitis Guideline. 2012.(42)  The Royal Children's Hospital Melbourne. 2013.(43)  Sydney Children's Hospital. 2011.(44)  Zentz SE. 2011.(45)  American Adacemy of Pediatrics. 2006.(46)  Scottish Intercollegiate Guidelines Network. 2006.(47) | 8.5  (1.2) | Grade D | U | Diagnosis | ED  GP  Hosp |
|  |  | BRON08 | Infants (aged less than 12months) presenting with acute bronchiolitis had their hydration status reviewed. | 14 | SA Child Health Clinical Network. 2013.(40)  NSW Kids and Families. 2012.(41)  The Royal Children's Hospital Melbourne. Bronchiolitis Guideline. 2012.(42)  The Royal Children's Hospital Melbourne. 2013.(43)  Sydney Children's Hospital. 2011.(44)  Zentz SE. 2011.(45)  American Adacemy of Pediatrics. 2006.(46)  Scottish Intercollegiate Guidelines Network. 2006.(47) | 8.4  (1.2) | Grade D | U | Diagnosis | ED  GP  Hosp |
|  |  | BRON09 | Infants (aged less than 12months) presenting with acute bronchiolitis received a respiratory examination (work of breathing, recession, auscultation). | 14 | SA Child Health Clinical Network. 2013.(40)  NSW Kids and Families. 2012.(41)  The Royal Children's Hospital Melbourne. Bronchiolitis Guideline. 2012.(42)  The Royal Children's Hospital Melbourne. 2013.(43)  Sydney Children's Hospital. 2011.(44)  Zentz SE. 2011.(45)  American Adacemy of Pediatrics. 2006.(46)  Scottish Intercollegiate Guidelines Network. 2006.(47) | 8.9  (0.4) | Grade D | U | Diagnosis | ED  GP  Hosp |
|  |  | BRON10 | Infants (aged less than 12months) presenting with acute bronchiolitis had their feeding (duration and volume, oxygen saturations whilst feeding) examined. | 14 | SA Child Health Clinical Network. 2013.(40)  NSW Kids and Families. 2012.(41)  The Royal Children's Hospital Melbourne. Bronchiolitis Guideline. 2012.(42)  The Royal Children's Hospital Melbourne. 2013.(43)  Sydney Children's Hospital. 2011.(44)  Zentz SE. 2011.(45)  American Adacemy of Pediatrics. 2006.(46)  Scottish Intercollegiate Guidelines Network. 2006.(47) | 8  (1.2) | Grade D | U | Diagnosis | ED  GP  Hosp |
| 3 | Infants (aged less than 12 months) presenting with any of the following signs / symptoms are diagnosed with mild acute bronchiolitis:  appear well AND  mild tachypnoea (RR less than 60/min) AND  normal or mildly increased work of breathing (WOB) i.e. no nasal flaring / grunting AND  wheeze or crackles AND  no cyanosis AND  SaO2 greater than 93% on air AND  mild or no tachycardia AND  normal / slightly decreased feeding or may take longer to feed, intermittently stops feeding. | BRON11 | Infants (aged <12 months) who had any of the following signs / symptoms: * appear well *mild tachypnoea (RR < 60/min) *normal or mildly increased work of breathing (WOB) i.e. no nasal flaring/grunting *wheeze at end expiratory or crackles *no cyanosis *SaO2 > 93% on air *no tachycardia *normal/slightly decreased feeding or may take longer to feed, intermittently stops feeding  were diagnosed with mild acute bronchiolitis | 12 | NSW Kids and Families. 2012.(41)  Sydney Children's Hospital. 2011.(44)  Princess Margaret Hospital for Children.2008. | 8.1  (1.5) | Consensus-based recommendation | U | Diagnosis | ED  GP  Hosp |
| 4 | Infants (aged less than 12 months) presenting with two or more of the following signs / symptoms are diagnosed with moderate acute bronchiolitis:  appear mildly unwell OR  moderate tachypnoea (RR greater than 60/min) OR  mild to moderate WOB (i.e. some chest wall retractions, mild nasal flaring and tracheal tug) OR  no cyanosis OR  SaO2 90-95% on air OR  moderate tachycardia OR  difficult feeding but able to take greater than 50% of normal feed, frequent stops. | BRON12 | Infants (aged <12 months) who had two or more of the following signs / symptoms: *appear mildly unwell *moderate tachypnoea (RR > 60/min) *mild to moderate WOB *no cyanosis *SaO2 90-95% on air *mild tachycardia *difficult feeding but able to take >50% of normal feed, frequent stops were diagnosed with moderate acute bronchiolitis. | 12 | NSW Kids and Families. 2012.(41)  Sydney Children's Hospital. 2011.(44)  Princess Margaret Hospital for Children.2008.(48) | 7.8  (1.3) | Consensus-based recommendation | U | Diagnosis | ED  GP  Hosp |
| 5 | Infants (aged less than 12 months) presenting with two or more of the following signs / symptoms are diagnosed with severe / life threatening acute bronchiolitis:  appear unwell (lethargic, restless) OR  severe tachypnoea greater than 70 OR  bradypnoea less than 30 OR  moderate to severe WOB (i.e. grunting, marked chest wall retractions, nasal flaring) OR  may be cyanosed or pale OR  SaO2 less than 90% on air, less than 92% on oxygen OR  tachycardia greater than 180 OR  difficult feeding taking less than 50% of normal feed, not interested OR  poor capillary refill greater than 3 secs. | BRON13 | Infants (aged <12 months) who had two or more of the following signs: *appear unwell (lethargic, restless) *severe tachypnoea >70 * bradypnoea <30 *moderate to severe WOB  *may be cyanosed or pale *SaO2 <90% on air, <92% on oxygen *tachycardia >180 *difficult feeding taking <50% of normal feed, not interested *poor capillary refill > 3 secs were diagnosed with severe/life threatening acute bronchiolitis. | 12 | NSW Kids and Families. 2012.(41)  Sydney Children's Hospital. 2011.(44)  Princess Margaret Hospital for Children.2008.(48) | 8.4  (0.4) | Consensus-based recommendation | U | Diagnosis | ED  GP  Hosp |
| 6 | Children diagnosed with acute mild / moderate bronchiolitis DO NOT have any of the following:  chest x-ray OR  routine blood tests OR  ABG OR  chest physiotherapy. | BRON14 | Children diagnosed with acute mild / moderate bronchiolitis did not have a chest x-ray. | 10 | NSW Kids and Families. 2012.(41)  The Royal Children's Hospital Melbourne. 2012.(42)  Sydney Children's Hospital. 2011.(44)  Zentz SE. 2011.(45)  Scottish Intercollegiate Guidelines Network. 2006.(47)  Princess Margaret Hospital for Children.2008.(48)  Royal Children's Hospital. 2009.(49) | 8.3  (0.8) | Level I  Grade C | O | Treatment | ED  GP  Hosp |
|  |  | BRON15 | Children diagnosed with acute mild / moderate bronchiolitis did not have routine blood tests. | 11 | NSW Kids and Families. 2012.(41)  The Royal Children's Hospital Melbourne. 2012.(42)  Sydney Children's Hospital. 2011.(44)  Zentz SE. 2011.(45)  Scottish Intercollegiate Guidelines Network. 2006.(47)  Princess Margaret Hospital for Children.2008.(48)  Royal Children's Hospital. 2009.(49) | 8.5  (0.8) | Grade C | O | Treatment | ED  GP  Hosp |
|  |  | BRON16 | Children diagnosed with acute mild / moderate bronchiolitis did not have an ABG. | 11 | NSW Kids and Families. 2012.(41)  The Royal Children's Hospital Melbourne. 2012.(42)  Sydney Children's Hospital. 2011.(44)  Zentz SE. 2011.(45)  Scottish Intercollegiate Guidelines Network. 2006.(47)  Princess Margaret Hospital for Children.2008.(48)  Royal Children's Hospital. 2009.(49) | 8.8  (0.4) | Level I  Grade C | O | Treatment | ED  GP  Hosp |
|  |  | BRON17 | Children diagnosed with acute mild / moderate bronchiolitis did not have chest physiotherapy. | 11 | NSW Kids and Families. 2012.(41)  The Royal Children's Hospital Melbourne. 2012.(42)  Sydney Children's Hospital. 2011.(44)  Zentz SE. 2011.(45)  Scottish Intercollegiate Guidelines Network. 2006.(47)  Princess Margaret Hospital for Children.2008.(48)  Royal Children's Hospital. 2009.(49) | 8.5  (0.9) | Level III  Grade A | O | Treatment | ED  GP  Hosp |
| 7 | Infants (aged less than 12 months) with mild bronchiolitis DO NOT receive:  prescribed oxygen NOR  further investigations (i.e. blood tests, chest x-ray). | BRON18 | Infants (aged less than 12 months) with mild bronchiolitis did not receive prescribed oxygen. | 11 | NSW Kids and Families. 2012.(41)  The Royal Children's Hospital Melbourne. 2012.(42)  The Royal Children's Hospital Melbourne. 2013.(43)  Scottish Intercollegiate Guidelines Network. 2006.(47)  Royal Children's Hospital. 2009.(49) | 8.5  (0.7) | Grade B | O | Treatment | ED  GP  Hosp |
|  |  | BRON19 | Infants (aged less than 12 months) with mild bronchiolitis did not receive further investigations (i.e. blood tests, chest x-ray). | 11 | NSW Kids and Families. 2012.(41)  The Royal Children's Hospital Melbourne. 2012.(42)  The Royal Children's Hospital Melbourne. 2013.(43)  Scottish Intercollegiate Guidelines Network. 2006.(47)  Royal Children's Hospital. 2009.(49) | 8.8  (0.4) | Grade D  (blood tests)  Grade C  (chest x-ray) | O | Treatment | ED  GP  Hosp |
| 8 | Infants (aged less than 12months) with moderate bronchiolitis receive:  prescribed oxygen to maintain saturations greater than equals to 93% AND  have small frequent feeds or consider NG feeds AND  if prescribed oxygen have continuous SaO2 monitoring AND  Do NOT have further investigations (i.e. blood tests, chest x-ray) AND  have at least two hourly observations recorded (temp 4/24). | BRON20 | Infants (aged <12months) with moderate bronchiolitis were prescribed oxygen to maintain saturation levels of ≥93% | 11 | NSW Kids and Families. 2012.(41)  The Royal Children's Hospital Melbourne. 2012.(42)  Royal Children's Hospital. 2009.(49) | 8.3  (0.6) | Grade D | U | Treatment | ED Hosp |
|  |  | BRON21 | Infants (aged <12months) with moderate bronchiolitis were provided with frequent feeds or NG feeds were considered | 11 | NSW Kids and Families. 2012.(41)  The Royal Children's Hospital Melbourne. 2012.(42)  Royal Children's Hospital. 2009.(49) | 8.3  (1.0) | Consensus-based recommendation | U | Treatment | ED Hosp |
|  |  | BRON22 | Infants (aged <12months) with moderate bronchiolitis and prescribed oxygen had continuous saturation monitoring and hourly observations. | 10 | NSW Kids and Families. 2012.(41)  The Royal Children's Hospital Melbourne. 2012.(42)  Royal Children's Hospital. 2009.(49) | 7.2  (1.9) | Consensus-based recommendation | U | Treatment | ED Hosp |
|  |  | BRON23 | Infants (aged <12months) with moderate bronchiolitis did not have further investigations performed (i.e. blood tests, chest x-ray). | 11 | NSW Kids and Families. 2012.(41)  The Royal Children's Hospital Melbourne. 2012.(42)  Royal Children's Hospital. 2009.(49) | 8.3  (0.8) | Grade D  (blood tests)  Grade C  (chest x-ray) | O | Treatment | ED Hosp |
|  |  | BRON24 | Infants (aged <12months) with moderate bronchiolitis had two hourly observations performed. | 11 | NSW Kids and Families. 2012.(41)  The Royal Children's Hospital Melbourne. 2012.(42)  Royal Children's Hospital. 2009.(49) | 8.2  (1.1) | Consensus-based recommendation | U | Treatment | ED Hosp |
| 9 | Infants (aged less than 12 months) with mild to moderate bronchiolitis caused by a viral infection are NOT prescribed antibiotics. | BRON25 | Infants (aged < 12 months) with mild to moderate bronchiolitis caused by a viral infection were not prescribed antibiotics. | 11 | NSW Kids and Families. 2012.(41)  The Royal Children's Hospital Melbourne. 2012.(42)  Zentz SE. 2011.(45)  American Adacemy of Pediatrics. 2006.(46)  Scottish Intercollegiate Guidelines Network. 2006.(47)  Princess Margaret Hospital for Children. 2008.(48) | 8.7  (0.6) | Level I  Grade B | O | Treatment | ED  GP  Hosp |
| 10 | Infants (aged less than 12months) with severe bronchiolitis are managed as follows:  prescribed oxygen to maintain saturations greater than equals to 93% AND  prescribed IV fluids or supplemental NG feeds AND  have their blood glucose assessed at least once AND  have continuous cardio-respiratory and SpO2 monitoring and observations recorded. | BRON26 | Infants (aged <12months) with severe bronchiolitis were prescribed oxygen to maintain saturation levels of ≥93%. | 11 | NSW Kids and Families. 2012.(41)  The Royal Children's Hospital Melbourne. 2012.(42)  Royal Children's Hospital. 2009.(49) | 7.9  (1.8) | Grade D | U | Treatment | ED Hosp |
|  |  | BRON27 | Infants (aged <12months) with severe bronchiolitis were prescribed IV fluids and nil by mouth. | 11 | NSW Kids and Families. 2012.(41)  The Royal Children's Hospital Melbourne. 2012.(42)  Royal Children's Hospital. 2009.(49) | 7.3  (2.2) | Consensus-based recommendation | U | Treatment | ED Hosp |
|  |  | BRON28 | Infants (aged <12months) with severe bronchiolitis had their blood glucose assessed at least once during this presentation/admission. | 11 | NSW Kids and Families. 2012.(41)  The Royal Children's Hospital Melbourne. 2012.(42)  Royal Children's Hospital. 2009.(49) | 7.8  (1.6) | Consensus-based recommendation | U | Treatment | ED Hosp |
|  |  | BRON29 | Infants (aged <12months) with severe bronchiolitis had continuous cardio-respiratory and saturation monitoring and hourly observations. | 11 | NSW Kids and Families. 2012.(41)  The Royal Children's Hospital Melbourne. 2012.(42)  Royal Children's Hospital. 2009.(49) | 8.5  (0.9) | Consensus-based recommendation | U | Treatment | ED Hosp |
| 11 | Infants (aged less than 12 months) presenting to the ED with acute bronchiolitis and any of the following are reviewed within 30 minutes:  lethargy OR  presence of nasal flaring and/or grunting OR  oxygen saturation less than 93% on air OR  uncertainty regarding diagnosis. | BRON30 | Infants (aged <12 months) who presented to the ED with acute bronchiolitis and any of the following: *lethargy OR  *presence of nasal flaring and/or grunting OR *oxygen saturation <95% on air OR *uncertainty regarding diagnosis were reviewed within 30 minutes. | 10 | Scottish Intercollegiate Guidelines Network. 2006.(47) | 8.1  (1.2) | Consensus-based recommendation | U | Treatment | ED |
| 12 | Infants (aged less than 12 months) presenting to the ED with acute bronchiolitis and any of the following are reviewed within 30 minutes (Triage Category 3):  respiratory rate greater than 60/min or less than 30/min OR  presence of nasal flaring and/or grunting OR  SpO2 less than 92% on air OR  severe chest wall recession OR  cyanosis | BRON31 | Infants (aged. <12 months) who presented to the ED with acute bronchiolitis and any of the following: *respiratory rate >60/min or <30/min *presence of nasal flaring and/or grunting *SpO2 < 92% on air *severe chest wall recession *cyanosis were reviewed immediately. | 10 | Scottish Intercollegiate Guidelines Network. 2006.(47) | 8.4  (1.8) | Consensus-based recommendation | U | Treatment | ED |
| 13 | Infants (aged less than 12 months) with acute bronchiolitis are NOT prescribed any of the following medications:  nebulised adrenaline OR  bronchodilators (if aged less than 6 months) OR  corticosteroid medication (unless asthma or chronic neonatal lung disease) OR  ipratropium bromide (unless asthma or chronic neonatal lung disease) OR  ribavirin (antiviral) unless there is significant immunosuppression. | BRON32 | Infants (aged <12 months) with acute bronchiolitis were not prescribed any of the following medications:  *nebulised adrenaline *bronchodilators (if aged <6 months) *corticosteroid medication (unless asthma or chronic neonatal lung disease) * ipratropium bromide (possible asthma or chronic neonatal lung disease) *ribavirin (antiviral)unless there is significant immunosuppression | 10 | NSW Kids and Families. 2012.(41)  The Royal Children's Hospital Melbourne. 2012.(42)  Sydney Children's Hospital. 2011.(44)   \|  \| Zentz SE. 2011.(45) \| \| --- \| --- \|   American Adacemy of Pediatrics. 2006.(46)  Scottish Intercollegiate Guidelines Network. 2006.(47)  Princess Margaret Hospital for Children. 2008.(48) | 8.1  (1.1) | Consensus-based recommendation | O | Treatment | ED  GP  Hosp |
| 14 | Parents of infants (aged less than 12 months) with mild bronchiolitis receive:  advice to provide small frequent feeds AND  written information at discharge on the expected course of the illness and how to recognise any deterioration in condition AND  advised to seek review by a health professional within in 24hrs (i.e. GP, OPD, clinic). | BRON33 | Parents of infants (aged <12 months) with mild bronchiolitis received advice to provide small frequent feeds | 10 | NSW Kids and Families. 2012.(41)  The Royal Children's Hospital Melbourne. 2012.(42)  The Royal Children's Hospital Melbourne. 2013.(43)  Scottish Intercollegiate Guidelines Network. 2006.(47)  Royal Children's Hospital. 2009.(49) | 8  (1.5) | Consensus-based recommendation | U | Ongoing management | ED  GP  Hosp |
|  |  | BRON34 | Parents of infants (aged <12 months) with mild bronchiolitis were provided written information prior to discharge. | 10 | NSW Kids and Families. 2012.(41)  The Royal Children's Hospital Melbourne. 2012.(42)  The Royal Children's Hospital Melbourne. 2013.(43)  Scottish Intercollegiate Guidelines Network. 2006.(47)  Royal Children's Hospital. 2009.(49) | 8.2  (1.3) | Consensus-based recommendation | U | Ongoing management | ED Hosp |
|  |  | BRON35 | Parents of infants (aged <12 months) with mild bronchiolitis were advised to follow-up with a health professional within 24hrs. | 10 | NSW Kids and Families. 2012.(41)  The Royal Children's Hospital Melbourne. 2012.(42)  The Royal Children's Hospital Melbourne. 2013.(43)  Scottish Intercollegiate Guidelines Network. 2006.(47)  Royal Children's Hospital. 2009.(49) | 7.6  (2.0) | Consensus-based recommendation | U | Ongoing management | ED Hosp |
| 15 | Infants (aged less than 12 months) presenting to the GP with acute bronchiolitis and two of the following are referred to hospital:  poor feeding (less than 50% of usual fluid intake in preceding 24 hours) OR  lethargy OR  history of apnoea OR  respiratory rate greater than 60/min or less than 30/min OR  presence of nasal flaring and / or grunting OR  severe chest wall recession or tracheal tug OR  cyanosis OR  oxygen saturation less than 95% on air OR  uncertainty regarding diagnosis. | BRON36 | Infants (aged <12 months) who presented to the GP with acute bronchiolitis and two of the following: *poor feeding (<50% of usual fluid intake in preceding 24 hours *lethargy *history of apnoea *respiratory rate >60/min OR <30/min *presence of nasal flaring and/or grunting *severe chest wall recession or tracheal tug *cyanosis *oxygen saturation <95% on air *uncertainty regarding diagnosis were referred to hospital. | 9 | Scottish Intercollegiate Guidelines Network. 2006.(47) | 8.3  (1.3) | Consensus-based recommendation | U | Ongoing management | GP |
|  | Infants (aged less than 12 months) with bronchiolitis can be discharged home if the following criteria are met  minimal respiratory distress AND  maintaining an adequate daily oral intake (greater than 75% of usual intake) AND  SpO2 greater than equals to 92% on room air (including during sleep periods) AND  parents and carers have received education and written information (see definitions), support and follow-up arrangements. | BRON37 | Infants (aged <12 months) with bronchiolitis who were discharged had minimal respiratory distress. | 10 | NSW Kids and Families. 2012.(41)  The Royal Children's Hospital Melbourne. 2013.(43)  Scottish Intercollegiate Guidelines Network, 2006.(47) | 8.7  (0.5) | Consensus-based recommendation | U | Ongoing management | ED Hosp |
|  |  | BRON38 | Infants (aged <12 months) with bronchiolitis who were discharged maintained an adequate daily oral intake (>75% of usual intake). | 10 | NSW Kids and Families. 2012.(41)  The Royal Children's Hospital Melbourne. 2013.(43)  Scottish Intercollegiate Guidelines Network, 2006.(47) | 8.3  (0.8) | Consensus-based recommendation | U | Ongoing management | ED Hosp |
|  |  | BRON39 | Infants (aged <12 months) with bronchiolitis who were discharged had oxygen saturations which were ≥ 92% on room air (including during sleep periods). | 10 | NSW Kids and Families. 2012.(41)  The Royal Children's Hospital Melbourne. 2013.(43)  Scottish Intercollegiate Guidelines Network, 2006.(47) | 7.7  (2.5) | Consensus-based recommendation | U | Ongoing management | ED Hosp |
|  |  | BRON40 | Parents / carers of infants (aged <12 months) with bronchiolitis who were discharged were provided: *education and written information  *support and follow-up arrangements | 9 | NSW Kids and Families. 2012.(41)  The Royal Children's Hospital Melbourne. 2013.(43)  Scottish Intercollegiate Guidelines Network, 2006.(47) | 8.2  (1.1) | Consensus-based recommendation | U | Ongoing management | ED Hosp |
| **CROUP** | | | | | | | | | | |
| 1 | Children diagnosed with croup have the following assessed to determine the severity:  heart rate AND  mental state AND  work of breathing AND  stridor AND  SpO2 / oxygen requirement. | CROU01 | Children diagnosed with croup had their heart rate assessed. | 8 | The Royal Children's Hospital Melbourne. 2011.(50)  NSW Health. 2010.(51)  Rajapaksa S, Starr M. 2010.(52)  Health for Kids in the South East, Southern Health. 2007.(53)  Sydney West Area Health Service. 2004.(54)  Harrison J, Massie J.2009.(55) | 7.8  (1.6) | Grade D | U | Diagnosis | ED  GP  Hosp |
|  |  | CROU02 | Children diagnosed with croup had their mental state assessed. | 8 | The Royal Children's Hospital Melbourne. 2011.(50)  NSW Health. 2010.(51)  Rajapaksa S, Starr M. 2010.(52)  Health for Kids in the South East, Southern Health. 2007.(53)  Sydney West Area Health Service. 2004.(54)  Harrison J, Massie J.2009.(55) | 8.4  (0.9) | Grade D | U | Diagnosis | ED  GP  Hosp |
|  |  | CROU03 | Children diagnosed with croup had their work of breathing assessed. | 8 | The Royal Children's Hospital Melbourne. 2011.(50)  NSW Health. 2010.(51)  Rajapaksa S, Starr M. 2010.(52)  Health for Kids in the South East, Southern Health. 2007.(53)  Sydney West Area Health Service. 2004.(54)  Harrison J, Massie J.2009.(55) | 8.9  (0.4) | Grade D | U | Diagnosis | ED  GP  Hosp |
|  |  | CROU04 | Children diagnosed with croup were assessed for stridor. | 8 | The Royal Children's Hospital Melbourne. 2011.(50)  NSW Health. 2010.(51)  Rajapaksa S, Starr M. 2010.(52)  Health for Kids in the South East, Southern Health. 2007.(53)  Sydney West Area Health Service. 2004.(54)  Harrison J, Massie J.2009.(55) | 8.9  (0.4) | Grade D | U | Diagnosis | ED  GP  Hosp |
|  |  | CROU05 | Children diagnosed with croup had their SpO2 and oxygen requirement assessed. | 8 | The Royal Children's Hospital Melbourne. 2011.(50)  NSW Health. 2010.(51)  Rajapaksa S, Starr M. 2010.(52)  Health for Kids in the South East, Southern Health. 2007.(53)  Sydney West Area Health Service. 2004.(54)  Harrison J, Massie J.2009.(55) | 8  (1.2) | Grade D | U | Diagnosis | ED  GP  Hosp |
| 2 | Children diagnosed with croup have the severity recorded as mild / moderate / severe. | CROU06 | Children diagnosed with croup had their severity recorded as mild, moderate, or severe. | 8 | The Royal Children's Hospital Melbourne. 2011.(50)  NSW Health. 2010.(51)  Rajapaksa S, Starr M. 2010.(52)  Health for Kids in the South East, Southern Health. 2007.(53)  Sydney West Area Health Service. 2004.(54)  Harrison J, Massie J.2009.(55) | 7.5  (1.2) | Grade D | U | Diagnosis | ED  GP  Hosp |
| 3 | Children diagnosed with croup do NOT have any of the following investigations:  nasopharyngeal aspirate OR  chest x-ray OR  lateral neck x-ray OR  blood tests. | CROU07 | Children diagnosed with croup did not have a nasopharyngeal aspirate. | 8 | The Royal Children's Hospital Melbourne. 2011.(50)  Health for Kids in the South East, Southern Health. 2007.(53)  Sydney West Area Health Service. 2004.(54)  Alberta Medical Association. 2008.(56)  Joondalup Health Campus.(57) | 7.8  (2.1) | Grade D | O | Diagnosis | ED  GP  Hosp |
|  |  | CROU08 | Children diagnosed with croup did not have a chest x-ray. | 8 | The Royal Children's Hospital Melbourne. 2011.(50)  Health for Kids in the South East, Southern Health. 2007.(53)  Sydney West Area Health Service. 2004.(54)  Alberta Medical Association. 2008.(56)  Joondalup Health Campus.(57) | 7.5  (2.3) | Grade C | O | Diagnosis | ED  GP  Hosp |
|  |  | CROU09 | Children diagnosed with croup did not have a lateral neck x-ray. | 8 | The Royal Children's Hospital Melbourne. 2011.(50)  Health for Kids in the South East, Southern Health. 2007.(53)  Sydney West Area Health Service. 2004.(54)  Alberta Medical Association. 2008.(56)  Joondalup Health Campus.(57) | 7.1  (2.8) | Consensus based recommendation | O | Diagnosis | ED  GP  Hosp |
|  |  | CROU10 | Children diagnosed with croup did not have blood tests. | 8 | The Royal Children's Hospital Melbourne. 2011.(50)  Health for Kids in the South East, Southern Health. 2007.(53)  Sydney West Area Health Service. 2004.(54)  Alberta Medical Association. 2008.(56)  Joondalup Health Campus.(57) | 7.3  (2.8) | Grade D | O | Diagnosis | ED  GP  Hosp |
| 4 | Children aged less than 3 months who present with croup and any of the following (Expiratory wheeze or loss of voice, Toxic appearance or high-grade fever, Drooling, difficulty swallowing, anxiety OR Prolonged, or recurrent stridor) are assessed for the following conditions:  epiglottitis AND  inhaled foreign body AND  bacterial tracheitis. | CROU11 | Children aged less than 3 months who presented with croup and any of the following:  - expiratory wheeze or loss of voice - toxic appearance or high-grade fever - drooling - difficulty swallowing - anxiety - prolonged or recurrent stridor were assessed for epiglottitis. | 8 | Health for Kids in the South East, Southern Health. 2007.(53) | 8.3  (1.4) | Grade D | U | Diagnosis | ED  GP  Hosp |
|  |  | CROU12 | Children aged less than 3 months who presented with croup and any of the following:  - expiratory wheeze or loss of voice - toxic appearance or high-grade fever - drooling - difficulty swallowing - anxiety - prolonged or recurrent stridor were assessed for an inhaled foreign body. | 8 | Health for Kids in the South East, Southern Health. 2007.(53) | 8  (1.2) | Grade D | U | Diagnosis | ED  GP  Hosp |
|  |  | CROU13 | Children aged less than 3 months who presented with croup and any of the following:  - expiratory wheeze or loss of voice - toxic appearance or high-grade fever - drooling - difficulty swallowing - anxiety - prolonged or recurrent stridor were assessed for bacterial tracheitis. | 8 | Health for Kids in the South East, Southern Health. 2007.(53) | 8  (1.2) | Grade D | U | Diagnosis | ED  GP  Hosp |
| 5 | Children diagnosed with croup are NOT treated with any of the following:  mist, humidified or cold air OR  anti-tussives OR  antibiotics (includes the reason for prescription) OR  sedatives. | CROU14 | Children diagnosed with croup were not treated with mist, humidified or cold air. | 8 | The Royal Children's Hospital Melbourne. 2011.(50)  Health for Kids in the South East, Southern Health. 2007.(53)  Harrison J, Massie J. 2009.(55)  Alberta Medical Association. 2009.(56)  National Institute for Health and Care Excellence. 2012.(58) | 8.1  (1.5) | Grade B | O | Treatment | ED  GP  Hosp |
|  |  | CROU15 | Children diagnosed with croup were not treated with anti-tussives. | 8 | The Royal Children's Hospital Melbourne. 2011.(50)  Health for Kids in the South East, Southern Health. 2007.(53)  Harrison J, Massie J. 2009.(55)  Alberta Medical Association. 2009.(56)  National Institute for Health and Care Excellence. 2012.(58) | 8.9  (0.4) | Consensus-based recommendation | O | Treatment | ED  GP  Hosp |
|  |  | CROU16 | Children diagnosed with croup were not treated with antibiotics. | 8 | The Royal Children's Hospital Melbourne. 2011.(50)  Health for Kids in the South East, Southern Health. 2007.(53)  Harrison J, Massie J. 2009.(55)  Alberta Medical Association. 2009.(56)  National Institute for Health and Care Excellence. 2012.(58) | 7.5  (2.8) | Consensus-based recommendation | O | Treatment | ED  GP  Hosp |
|  |  | CROU17 | Children diagnosed with croup were not treated with sedatives. | 8 | The Royal Children's Hospital Melbourne. 2011.(50)  Health for Kids in the South East, Southern Health. 2007.(53)  Harrison J, Massie J. 2009.(55)  Alberta Medical Association. 2009.(56)  National Institute for Health and Care Excellence. 2012.(58) | 9  (0) | Consensus-based recommendation | O | Treatment | ED  GP  Hosp |
| 6 | Children diagnosed with mild to moderate croup who have signs of stridor are prescribed steroids as follows:  Prednisolone 1mg/kg, and repeat 12-24 hrs later OR  a single dose of Oral Dexamethasone 0.15mg/kg OR  Nebulised Budesonide 2 mg if oral is not tolerated. | CROU18 | Children diagnosed with mild to moderate croup and who had signs of stridor were prescribed: - prednisolone at 1mg/kg, and repeated 12-24 hrs later OR - a single dose of Oral Dexamethasone 0.15mg/kg, OR - Nebulised Budesonide 2 mg if oral is not tolerated. | 8 | The Royal Children's Hospital Melbourne. 2011.(50)  NSW Health. 2010.(51)  Rajapaksa S, Starr M. 2010.(52)  Sydney West Area Health Service. 2004.(54)   \|  \| Harrison J, Massie J. 2009.(55) \| \| --- \| --- \|   Alberta Medical Association. 2008.(56)  Joondalup Health Campus.(57)  National Institute for Health and Care Excellence. 2012.(58) | 7.9  (1.9) | Level IV  (steroids)  Level I  (oral steroid)  Level II  (nebulised budenoside) | U | Treatment | ED  GP  Hosp |
| 7 | Children with moderate to severe croup AND SpO2 less than 93% have oxygen administered. | CROU19 | Children with moderate to severe croup AND SpO2 less than 93% had oxygen administered. | 8 | NSW Health. 2010.(51)  Health for Kids in the South East, Southern Health. 2007.(53) | 8  (1.8) | Consensus-based recommendation | U | Treatment | ED  GP  Hosp |
| 8 | Children diagnosed with severe croup receive the following treatment:  O2 if SpO2 less than 93% AND  Nebulised adrenaline AND  Dexamethasone (IM/IV/PO) (or prednisolone if dexamethasone unavailable) or Nebulised Budesonide. | CROU20 | Children diagnosed with severe croup and had a SpO2 of less than 93% received O2. | 8 | The Royal Children's Hospital Melbourne. 2011.(50)  NSW Health. 2010.(51)  Rajapaksa S, Starr M. 2010.(52)  Health for Kids in the South East, Southern Health. 2007.(53)  Harrison J, Massie J. 2009.(55)  Alberta Medical Association. 2008.(56)  National Institute for Health and Care Excellence. 2012.(58) | 8.3  (1.0) | Grade D | U | Treatment | ED  GP  Hosp |
|  |  | CROU21 | Children diagnosed with severe croup received nebulised adrenaline. | 8 | The Royal Children's Hospital Melbourne. 2011.(50)  NSW Health. 2010.(51)  Rajapaksa S, Starr M. 2010.(52)  Health for Kids in the South East, Southern Health. 2007.(53)  Harrison J, Massie J. 2009.(55)  Alberta Medical Association. 2008.(56)  National Institute for Health and Care Excellence. 2012.(58) | 8.9  (0.4) | Level II  Grade A | U | Ongoing management | ED Hosp |
|  |  | CROU22 | Children diagnosed with severe croup received Dexamethasone or Prednisolone (IM/IV/PO), or Nebulised Budesonide. | 8 | The Royal Children's Hospital Melbourne. 2011.(50)  NSW Health. 2010.(51)  Rajapaksa S, Starr M. 2010.(52)  Health for Kids in the South East, Southern Health. 2007.(53)  Harrison J, Massie J. 2009.(55)  Alberta Medical Association. 2008.(56)  National Institute for Health and Care Excellence. 2012.(58) | 8.9  (0.4) | Level II  Grade A  (steroids)  Level II  (nebulised budesonide) | U | Ongoing management | ED  GP  Hosp |
| 9 | Children with severe croup who present to their GP are transferred by ambulance to an emergency department / hospital. | CROU23 | Children with severe croup who presented to their GP were transferred by ambulance to an emergency department / hospital. | 8 | The Royal Children's Hospital Melbourne. 2011.(50)  NSW Health. 2010.(51)  Health for Kids in the South East, Southern Health. 2007.(53)  Harrison J, Massie J. 2009.(55)  National Institute for Health and Care Excellence. 2012.(58) | 9  (0) | Consensus-based recommendation | U | Ongoing management | GP |
| 10 | Children diagnosed with severe croup who were administered nebulised adrenaline are managed as follows:  - Improvement noted - observed for 4 hours OR" | CROU24 | Children diagnosed with severe croup who were administered nebulised adrenaline and improved, were observed for 4 hours. | 8 | The Royal Children's Hospital Melbourne. 2011.(50)  NSW Health. 2010.(51) | 8.8  (0.5) | Consensus-based recommendation | U | Ongoing management | ED Hosp |
| 11 | Children diagnosed with severe croup are discharged when all of the following have occurred:  stridor free at rest (four hours post nebulised adrenaline) AND  parents provided with croup factsheet, education or advice. | CROU25 | Children diagnosed with severe croup who were stridor free at rest (four hours post nebulised adrenaline) AND whose parents were provided with croup factsheet, education or advice, were discharged. | 8 | The Royal Children's Hospital Melbourne. 2011.(50)  NSW Health. 2010.(51)  Harrison J, Massie J. 2009.(55) | 8.3  (1.0) | Grade D | U | Ongoing management | ED Hosp |
| 12 | Parents / carers of children with croup are advised to seek urgent medical advice if any of the following occur:  child becomes toxic (pale, very high fever, tachycardic). | CROU26 | Parents / carers of children with croup who become toxic (pale, very high fever, tachycardic) were advised to seek urgent medical advice. | 8 | The Royal Children's Hospital Melbourne. 2011.(50)  NSW Health. 2010. (51)  Health for Kids in the South East, Southern Health. 2007.(53)  Sydney West Area Health Service. 2004.(54)  Alberta Medical Association. 2008.(56)  Joondalup Health Campus.(57)  National Institute for Health and Care Excellence. 2012.(58) | 8.9  (0.4) | Grade D | U | Ongoing management | ED  GP  Hosp |
| **DIABETES** | | | | | | | | | | |
| 1 | Children and adolescents with type 1 diabetes have the following additional investigations performed at diagnosis:  Insulin antibodies AND  GAD antibodies. | DIAB01 | Children and adolescents with type 1 diabetes, at diagnosis, received investigations for insulin antibodies. | 5 | The Royal Childrens Hospital Melbourne. 2013.(59) | 5  (3.8) | Consensus-based recommendation | U | Diagnosis | ED  GP  Hosp  Spec |
|  |  | DIAB02 | Children and adolescents with type 1 diabetes, at diagnosis, received investigations for GAD antibodies. | 5 | The Royal Childrens Hospital Melbourne. 2013.(59) | 5  (3.8) | Consensus-based recommendation | U | Diagnosis | ED  GP  Hosp  Spec |
| 2 | Children and adolescents newly diagnosed with type 1 diabetes are screened for:  coeliac disease (total IgA, anti-gliadin Ab, tissue transglutaminase Ab) AND  thyroid dysfunction (TSH, FT4) | DIAB03 | Children and adolescents newly diagnosed with type 1 diabetes were screened for coeliac disease (total IgA, anti-gliadin Ab, tissue transglutaminase Ab). | 5 | Australasian Paediatric Endocrine Group, and the Australian Diabetes Society. 2011.(60) | 8.2  (0.8) | Grade B | U | Treatment | ED  GP  Hosp  Spec |
| 3 |  | DIAB04 | Children and adolescents newly diagnosed with type 1 diabetes were screened for thyroid dysfunction (TSH, FT4). | 5 | Australasian Paediatric Endocrine Group, and the Australian Diabetes Society. 2011.(60) | 8.6  (0.5) | Grade B | U | Treatment | ED  GP  Hosp  Spec |
| 4 | Children and adolescents diagnosed with type 1 diabetes who present with any of the following are assessed for co-occurrence of psychological disorders using a validated screening tool:  suboptimal glycaemic control (e.g. HbA1c greater than 10) OR"  insulin omission OR  disordered eating behaviours OR  recurrent admissions for diabetic ketoacidosis (DKA). | DIAB05 | Children and adolescents diagnosed with type 1 diabetes who presented with suboptimal glycaemic control (e.g. HbA1c greater than 10) were assessed for co-occurrence of psychological disorders using a validated screening tool. | 3 | Australasian Paediatric Endocrine Group, and the Australian Diabetes Society. 2011.(60) | 7.3  (1.5) | Consensus-based recommendation | U | Treatment | ED  GP  Hosp  Spec |
|  |  | DIAB06 | Children and adolescents diagnosed with type 1 diabetes who presented with insulin omission were assessed for co-occurrence of psychological disorders using a validated screening tool. | 3 | Australasian Paediatric Endocrine Group, and the Australian Diabetes Society. 2011.(60) | 7.3  (1.5) | Consensus-based recommendation | U | Treatment | ED  GP  Hosp  Spec |
|  |  | DIAB07 | Children and adolescents diagnosed with type 1 diabetes who presented with disorder eating behaviours were assessed for co-occurrence of psychological disorders using a validated screening tool. | 3 | Australasian Paediatric Endocrine Group, and the Australian Diabetes Society. 2011.(60) | 7.7  (1.5) | Consensus-based recommendation | U | Treatment | ED  GP  Hosp  Spec |
|  |  | DIAB08 | Children and adolescents diagnosed with type 1 diabetes who presented with recurrent admissions for diabetic ketoacidosis (DKA) were assessed for co-occurrence of psychological disorders using a validated screening tool. | 3 | Australasian Paediatric Endocrine Group, and the Australian Diabetes Society. 2011.(60) | 8.3  (1.2) | Consensus-based recommendation | U | Treatment | Hosp  Spec |
| 5 | Children and adolescents with type 1 diabetes have an intensive glycaemic control plan implemented to reduce the risk of development and/or progression of microvascular and macrovascular diabetes complications that includes:  MDI or CSII AND  frequent insulin dose adjustment AND  blood glucose level monitoring at least four times per day, AND  monitoring of HbA1c at least 4 monthly. | DIAB09 | Children and adolescents with type 1 diabetes had an intensive glycaemic control plan that included MDI or CSII. | 3 | Australasian Paediatric Endocrine Group, and the Australian Diabetes Society. 2011.(60) | 8.7  (0.6) | Grade B | U | Treatment | ED  GP  Hosp  Spec |
|  |  | DIAB10 | Children and adolescents with type 1 diabetes had an intensive glycaemic control plan implemented that included frequent insulin dose adjustment. | 3 | Australasian Paediatric Endocrine Group, and the Australian Diabetes Society. 2011.(60) | 8  (0) | Grade B | U | Treatment | ED  GP  Hosp  Spec |
|  |  | DIAB11 | Children and adolescents with type 1 diabetes had an intensive glycaemic control plan implemented that included blood glucose level monitoring at least four times per day. | 3 | Australasian Paediatric Endocrine Group, and the Australian Diabetes Society. 2011.(60) | 8.7  (0.6) | Grade B | U | Treatment | ED  GP  Hosp  Spec |
|  |  | DIAB12 | Children and adolescents with type 1 diabetes had an intensive glycaemic control plan implemented to reduce the risk of development and/or progression of microvascular and macrovascular diabetes complications that included monitoring of HbA1c at least 4 monthly. | 3 | Australasian Paediatric Endocrine Group, and the Australian Diabetes Society. 2011.(60) | 8.7  (0.6) | Grade B | U | Treatment | ED  GP  Hosp  Spec |
| 6 | Children and adolescents with type 1 diabetes presenting with signs of DKA have:  their level of dehydration assessed as mild (less than 4%), moderate (4-7%) or severe (greater than 7%) recorded AND  vital signs monitored AND  their level of consciousness assessed using the Glasgow coma scale AND  their airway and breathing assessed and maintained. | DIAB13 | Children and adolescents with type 1 diabetes who presented with signs of DKA had their level of dehydration recorded as mild (less than 4%), moderate (4-7%) or severe (greater than 7%). | 3 | The Royal Childrens Hospital Melbourne. 2013.(59) | 9  (0) | Consensus-based recommendation | U | Treatment | ED Hosp |
|  |  | DIAB14 | Children and adolescents with type 1 diabetes who presented with signs of DKA had their vital signs monitored. | 3 | The Royal Childrens Hospital Melbourne. 2013.(59) | 9  (0) | Consensus-based recommendation | U | Treatment | ED Hosp |
|  |  | DIAB15 | Children and adolescents with type 1 diabetes who presented with signs of DKA had their level of consciousness assessed using the Glasgow coma scale. | 3 | The Royal Childrens Hospital Melbourne. 2013.(59) | 9  (0) | Consensus-based recommendation | U | Treatment | ED Hosp |
|  |  | DIAB16 | Children and adolescents with type 1 diabetes who presented with signs of DKA had their airway and breathing assessed and maintained. | 3 | The Royal Childrens Hospital Melbourne. 2013.(59) | 8.3  (1.2) | Consensus-based recommendation | U | Treatment | ED Hosp |
| 7 | Children and adolescents with type 1 diabetes presenting with signs of DKA have the following assessments undertaken at the time of presentation:  Blood glucose, urea and electrolytes (sodium, potassium, calcium, magnesium, phosphate) AND  blood ketones (bedside test) AND  Venous blood gas (including bicarb). | DIAB17 | Children and adolescents with type 1 diabetes who presented with signs of DKA had their blood glucose, urea and electrolytes (sodium, potassium, calcium, magnesium, phosphate) assessed at the time of presentation. | 3 | The Royal Childrens Hospital Melbourne. 2013.(59) | 9  (0) | Consensus-based recommendation | U | Treatment | ED Hosp |
|  |  | DIAB18 | Children and adolescents with type 1 diabetes who presented with signs of DKA had their blood ketones (bedside test) assessed at the time of presentation. | 3 | The Royal Childrens Hospital Melbourne. 2013.(59) | 8.7  (0.6) | Consensus-based recommendation | U | Treatment | ED Hosp |
|  |  | DIAB19 | Children and adolescents with type 1 diabetes who presented with signs of DKA had their venous blood gas (including bicarb) assessed at the time of presentation. | 3 | The Royal Childrens Hospital Melbourne. 2013.(59) | 9  (0) | Consensus-based recommendation | U | Treatment | ED Hosp |
| 8 | Children and adolescents with type 1 diabetes presenting with signs of DKA and any of the following are managed with subcutaneous insulin:  test negative for ketones OR  have a normal pH in the presence of ketones. | DIAB20 | Children and adolescents with type 1 diabetes who presented with signs of DKA and tested negative for ketones were managed with subcutaneous insulin. | 3 | The Royal Childrens Hospital Melbourne. 2013.(59) | 7  (1.7) | Consensus-based recommendation | U | Treatment | ED Hosp |
|  |  | DIAB21 | Children and adolescents with type 1 diabetes who presented with signs of DKA and had a normal pH in the presence of ketones were managed with subcutaneous insulin. | 3 | The Royal Childrens Hospital Melbourne. 2013.(59) | 7.7  (0.6) | Consensus-based recommendation | U | Treatment | ED Hosp |
| 9 | Children and adolescents with type 1 diabetes presenting with signs of DKA and a BGL greater than equals to 11.1mmol/l have:  blood ketones tested on a capillary sample. | DIAB22 | Children and adolescents with type 1 diabetes who presented with signs of DKA and a BGL greater than or equal to 11.1mmol/l had blood ketones tested on a capillary sample. | 3 | The Royal Childrens Hospital Melbourne. 2013.(59) | 9  (0) | Consensus-based recommendation | U | Treatment | ED Hosp |
| 10 | Children and adolescents with type 1 diabetes presenting with severe DKA (blood glucose > 11 mmol/L, venous pH < 7.1, bicarbonate < 5 mmol/L) and hypoperfusion (delayed capillary return, tachycardia for age) receive:  a bolus of 0.9% normal saline (10ml/kg) AND  rehydration with normal saline and potassium AND  have fluid type adjusted according to ongoing Na, K and Glucose levels. | DIAB23 | Children and adolescents with type 1 diabetes who presented with severe DKA (blood glucose > 11 mmol/L, venous pH < 7.1, bicarbonate < 5 mmol/L) and hypoperfusion (delayed capillary return, tachycardia for age) received a bolus of 0.9% normal saline (10ml/kg). | 2 | The Royal Childrens Hospital Melbourne. 2013.(59) | 8.5  (0.7) | Consensus-based recommendation | U | Treatment | ED Hosp |
|  |  | DIAB24 | Children and adolescents with type 1 diabetes who presented with severe DKA (blood glucose > 11 mmol/L, venous pH < 7.1, bicarbonate < 5 mmol/L) and hypoperfusion (delayed capillary return, tachycardia for age) received rehydration with normal saline and potassium. | 3 | The Royal Childrens Hospital Melbourne. 2013.(59) | 9  (0) | Consensus-based recommendation | U | Treatment | ED Hosp |
|  |  | DIAB25 | Children and adolescents with type 1 diabetes who presented with severe DKA (blood glucose > 11 mmol/L, venous pH < 7.1, bicarbonate < 5 mmol/L) and hypoperfusion (delayed capillary return, tachycardia for age) had their fluid type adjusted according to ongoing Na, K and Glucose levels. | 3 | The Royal Childrens Hospital Melbourne. 2013.(59) | 9  (0) | Consensus-based recommendation | U | Treatment | ED Hosp |
| 11 | Children and adolescents with type 1 diabetes presenting with DKA are given potassium replacement therapy as follows:  Potassium is greater than 5.5 mmol/l or patient is anuric, defer start. | DIAB26 | Children and adolescents with type 1 diabetes who presented with DKA and a potassium greater than 5.5 mmol/l, or were anuric, had commencement of potassium replacement therapy deferred. | 3 | The Royal Childrens Hospital Melbourne. 2013.(59) | 7.7  (2.3) | Consensus-based recommendation | U | Treatment | ED Hosp |
| 12 | Children and adolescents with type 1 diabetes presenting with moderate to severe DKA have a repeat serum potassium within one hour of insulin being commenced. | DIAB27 | Children and adolescents with type 1 diabetes who presented with moderate to severe DKA had a repeat serum potassium within one hour of insulin being commenced. | 3 | The Royal Childrens Hospital Melbourne. 2013.(59) | 7.7  (2.3) | Consensus-based recommendation | U | Treatment | ED Hosp |
| 13 | Children and adolescents with type 1 diabetes are provided with face-to-face education within 6 weeks of diagnosis by a qualified dietician on:  accurate carbohydrate counting. | DIAB28 | Children and adolescents with type 1 diabetes were provided with face-to-face education within 6 weeks of diagnosis by a qualified dietician on accurate carbohydrate counting. | 2 | Australasian Paediatric Endocrine Group, and the Australian Diabetes Society. 2011.(60) | 8.5  (0.7) | Consensus-based recommendation | U | Ongoing management | ED  GP  Hosp  Spec |
| 14 | Children and adolescents with type 1 diabetes have a comprehensive sick-day management plan in their medical record that includes:  blood ketone measurement (or urine ketone measurement if blood ketone is not available) AND  written guidelines and details on 24 hour access to clinical advice | DIAB29 | Children and adolescents with type 1 diabetes had a comprehensive sick-day management plan in their medical record that included blood ketone measurement (or urine ketone measurement if blood ketone is not available). | 3 | Australasian Paediatric Endocrine Group, and the Australian Diabetes Society 2011.(60) | 7  (3.5) | Consensus-based recommendation | U | Ongoing management | ED  GP  Hosp  Spec |
|  |  | DIAB30 | Children and adolescents with type 1 diabetes had a comprehensive sick-day management plan in their medical record that included written guidelines and details on 24 hour access to clinical advice. | 3 | Australasian Paediatric Endocrine Group, and the Australian Diabetes Society 2011.(60) | 8.7  (0.6) | Consensus-based recommendation | U | Ongoing management | ED  GP  Hosp  Spec |
| 15 | Children and adolescents with type 1 diabetes who have any of the following are referred at presentation for consultation with local paediatric team:  DKA OR  hyper / hyponatremia. | DIAB31 | Children and adolescents with type 1 diabetes with DKA were referred at presentation for consultation with local paediatric team. | 3 | The Royal Childrens Hospital Melbourne. 2013.(59) | 7.7  (2.3) | Consensus-based recommendation | U | Ongoing management | ED Hosp |
|  |  | DIAB32 | Children and adolescents with type 1 diabetes with hypernatremia or hyponatremia were referred at presentation for consultation with local paediatric team. | 3 | The Royal Childrens Hospital Melbourne. 2013.(59) | 7.7  (2.3) | Consensus-based recommendation | U | Ongoing management | ED  GP  Hosp  Spec |
| 16 | Children and adolescents with type 1 diabetes presenting with DKA are transferred to and/or consulted with tertiary care for intensive care monitoring if they fit any of the following criteria:  Children less than 18 months of age OR  Coma OR  Signs of cerebral oedema. | DIAB33 | Children aged less than 18 months with type 1 diabetes who presented with DKA were transferred to and/or consulted with tertiary care for intensive care monitoring. | 3 | The Royal Childrens Hospital Melbourne. 2013.(59) | 9  (0) | Consensus-based recommendation | U | Ongoing management | ED Hosp |
|  |  | DIAB34 | Children and adolescents with type 1 diabetes who presented with DKA and coma were transferred to and/or consulted with tertiary care for intensive care monitoring. | 3 | The Royal Childrens Hospital Melbourne. 2013.(59) | 7.7  (0.6) | Consensus-based recommendation | U | Ongoing management | ED Hosp |
|  |  | DIAB35 | Children and adolescents with type 1 diabetes who presented with DKA and signs of cerebral oedema were transferred to and/or consulted with tertiary care for intensive care monitoring. | 3 | The Royal Childrens Hospital Melbourne. 2013.(59) | 9  (0) | Consensus-based recommendation | U | Ongoing management | ED Hosp |
| **DEPRESSION** | | | | | | | | | | |
| 1 | Children who present with suspected depression receive an assessment which includes:  appraisal of family circumstances AND  attempt to understand the presentation in terms of personal, family, interpersonal circumstances AND  impact on functioning AND  self-harm and/or suicidal intent AND  exclusion of other causes (e.g. physical illness, medication or illicit drug effect). | DEPR01 | Children who presented with suspected depression had their family circumstances assessed. | 3 | Royal Children's Hospital Melbourne. 2009.(61)  British Columbia.2010,(26)  NHMRC. 2011(62)  NICE. 2005(63), | 9  (0) | Consensus-based recommendation | U | Diagnosis | ED  GP  Hosp  Spec |
|  |  | DEPR02 | Children who presented with suspected depression had their personal and interpersonal circumstances assessed. | 3 | Royal Children's Hospital Melbourne. 2009.(61)  British Columbia. 2010(26),  NHMRC. 2011(62)  NICE. 2005(63), | 8.7  (0.6) | Consensus-based recommendation | U | Diagnosis | ED  GP  Hosp  Spec |
|  |  | DEPR03 | Children who presented with suspected depression had their functional level assessed. | 3 | Royal Children's Hospital Melbourne. 2009.(61)  British Columbia. 2010(26)  NHMRC. 2011(62)  NICE. 2005(63), | 9  (0) | Consensus-based recommendation | U | Diagnosis | ED  GP  Hosp  Spec |
|  |  | DEPR04 | Children who presented with suspected depression were assessed for self-harm and/or suicidal intent. | 3 | Royal Children's Hospital Melbourne. 2009.(61)  British Columbia. 2010(26)  NHMRC. 2011(62)  NICE. 2005(63), | 9  (0) | Consensus-based recommendation | U | Diagnosis | ED  GP  Hosp  Spec |
|  |  | DEPR05 | Children who presented with suspected depression were assessed for other causes. | 3 | Royal Children's Hospital Melbourne. 2009.(61)  British Columbia. 2010(26)  NHMRC. 2011(62)  NICE. 2005(63), | 9  (0) | Consensus-based recommendation | U | Diagnosis | ED  GP  Hosp  Spec |
| 2 | Children and adolescents with depression are provided with:  evidence-based management of depression (e.g. information leaflets/booklets/reliable websites such as Beyond Blue, Black Dog Institute) AND  offered community supports (e.g. information about support services, such as Lifeline phone number, Community mental health team). | DEPR06 | Children and adolescents with depression were provided information and resources about evidence-based management. | 3 | NICE. 2005,(63)  Cheung A. H et al. 2007.(64)  Zuckerbrot R. A., et al. 2007.(65) | 9  (0) | Grade A | U | Diagnosis | ED  GP  Hosp  Spec |
|  |  | DEPR07 | Children and adolescents with depression were offered community supports. | 3 | NICE. 2005(63),  Cheung A. H et al. 2007.(64)  Zuckerbrot R. A., et al. 2007.(65) | 9  (0) | Grade C | U | Diagnosis | ED  GP  Hosp  Spec |
| 3 | Children and adolescents with depression have treatment/management goals set. | DEPR08 | Children and adolescents with depression had treatment / management goals set. | 3 | Zuckerbrot R. A., et al. 2007.(65) | 9  (0) | Grade C | U | Treatment | ED  GP  Hosp  Spec |
| 4 | Children and adolescents with depression have a safety plan which includes developing an emergency communication mechanism. | DEPR09 | Children and adolescents with depression had an emergency safety plan. | 3 | British Columbia. 2010(26)  Zuckerbrot R. A., et al. 2007.(65) | 9  (0) | Grade C | U | Treatment | ED  GP  Hosp  Spec |
| 5 | Children and adolescents with mild depression are NOT prescribed antidepressant medication as a first-line intervention. | DEPR10 | Children and adolescents with mild depression were not prescribed antidepressant medication as a first-line intervention. | 3 | NICE. 2005(63).  Cheung A. H et al. 2007.(64) | 7  (2.8) | Grade B | O | Treatment | ED  GP  Hosp  Spec |
| 6 | Children and adolescents with moderate to severe depression receive psychological therapy as a first-line treatment. | DEPR11 | Children and adolescents with moderate / severe depression received psychological therapy as a first-line treatment. | 3 | NHMRC. 2011(62).  Cheung A. H et al. 2007.(64) | 7.3  (1.5) | Grade B | U | Treatment | GP Spec |
| 7 | Children and adolescents commencing SSRI therapy are monitored for:  adverse drug reactions (e.g. onset or increase in suicidal ideation) AND  mental state. | DEPR12 | Children and adolescents prescribed SSRI therapy were monitored for adverse drug reactions. | 3 | NHMRC. 2011(62).  Cheung A. H et al. 2007.(64)  The Royal Australian and New Zealand College of Psychiatrists, The Royal Australian College of General Practitioners & The Royal Australian College of Physicians. 2005.(66) | 9  (0) | Grade B | U | Treatment | ED  GP  Hosp  Spec |
|  |  | DEPR13 | Children and adolescents prescribed SSRI therapy had their mental state monitored. | 3 | NHMRC. 2011(62).  Cheung A. H et al. 2007.(64)  The Royal Australian and New Zealand College of Psychiatrists, The Royal Australian College of General Practitioners & The Royal Australian College of Physicians. 2005.(66) | 9  (0) | Grade B | U | Treatment | ED  GP  Hosp  Spec |
| 8 | Children and adolescents with depression are reviewed within 8 weeks of initial diagnosis and treatment to evaluate goals and outcomes relating to function in the following settings:  home, AND  school. | DEPR14 | Children and adolescents with depression had their level of functioning at home and their goals and outcomes assessed within 8 weeks of initial diagnosis. | 3 | Cheung A. H et al. 2007.(64) | 7.7  (1.2) | Grade D | U | Treatment | ED  GP  Hosp  Spec |
|  |  | DEPR15 | Children and adolescents with depression had their level of functioning at school and their goals and outcomes assessed within 8 weeks of initial diagnosis. | 3 | Cheung A. H et al. 2007.(64) | 7.7  (1.2) | Grade D | U | Treatment | ED  GP  Hosp  Spec |
| **ECZEMA** | | | | | | | | | | |
| 1 | Children who present with an itch (pruritus) and 3 or more of the following are diagnosed with atopic eczema:  History of involvement in skin creases (or face or extensor surfaces if under 18 months) OR"  A history of dry skin (xerosis) in the last year OR  Visible flexural eczema (or over face or extensor surfaces if less than 18 months of age). | ECZE01 | Children who presented with an itch (pruritus) and 3 or more of the following: - history of involvement in skin creases (or face or extensor surfaces if under 18 months) OR - history of dry skin (xerosis) in the last year OR - visible flexural eczema (or over face or extensor surfaces if less than 18 months of age) were diagnosed with atopic eczema. | 3 | Royal Children's Hospital. 2013.(67)  Scottish Intercollegiate Guidelines Network. 2011.(68) | 8.3  (0.6) | Consensus-based recommendation | U | Diagnosis | ED  GP  Hosp  Spec |
| 2 | Children diagnosed with atopic eczema have the severity of their eczema documented (mild / moderate / severe). | ECZE02 | Children diagnosed with atopic eczema had the severity of their eczema documented (mild / moderate / severe). | 3 | Royal Children's Hospital. 2013.(67)  Scottish Intercollegiate Guidelines Network. 2011.(68) | 8  (1) | Consensus-based recommendation | U | Diagnosis | ED  GP  Hosp  Spec |
| 3 | Children with atopic eczema who present with a flare up (acute deterioration) are prescribed topical:  - steroids (which should be applied once or twice daily). | ECZE03 | Children with atopic eczema who presented with a flare up (acute deterioration) were prescribed topical steroids (which should be applied once or twice daily). | 3 | Royal Children's Hospital. 2013.(67)  Scottish Intercollegiate Guidelines Network. 2011.(68) | 8  (1.7) | Grade B | U | Treatment | ED  GP  Hosp  Spec |
| 4 | Children with atopic eczema who presented with a flare up (acute deterioration) had wet dressings applied. | ECZE04 | Children with atopic eczema who presented with a flare up (acute deterioration) had wet dressings applied. | 2 | Royal Children's Hospital. 2013.(67) | 8.5  (0.7) | Consensus-based recommendation | U | Treatment | ED  GP  Hosp  Spec |
| 5 | Children with atopic eczema who present with a flare (acute deterioration) and infection are prescribed:  oral antibiotics (cephalexin or flucloxacillin) OR"  antivirals if secondary infection present OR  IV antibiotics if severe infection or sepsis. | ECZE05 | Children with atopic eczema who presented with a flare up (acute deterioration) and infection were prescribed: - oral antibiotics (cephalexin or flucloxacillin) OR - antivirals if secondary infection present OR - IV antibiotics if severe infection or sepsis. | 3 | Royal Children's Hospital. 2013.(67) | 8.7  (0.6) | Consensus-based recommendation | U | Treatment | ED  GP  Hosp  Spec |
| 6 | Children with eczema where an infection is suspected have swabs taken. | ECZE06 | Children with eczema where an infection was suspected had swabs taken. | 3 | Royal Children's Hospital. 2013.(67) | 7  (1.7) | Consensus-based recommendation | U | Treatment | ED  GP  Hosp  Spec |
| 7 | Children with atopic eczema and NO signs of infection are NOT prescribed antibiotics. | ECZE07 | Children with atopic eczema and no signs of infection were not prescribed antibiotics. | 3 | Scottish Intercollegiate Guidelines Network. 2011.(68) | 8.8  (0.5) | Grade B | O | Treatment | ED  GP  Hosp  Spec |
| 8 | Parents of children diagnosed with atopic eczema are advised to provide ongoing everyday treatments to avoid irritants. | ECZE08 | Parents of children diagnosed with atopic eczema were advised to provide ongoing everyday treatments to avoid irritants. | 2 | Royal Children's Hospital. 2013.(67)  Scottish Intercollegiate Guidelines Network. 2011.(68) | 7.5  (2.1) | Grade C | U | Ongoing management | ED  GP  Hosp  Spec |
| 9 | Children with atopic eczema who are admitted to hospital are discharged with a written eczema treatment plan. | ECZE09 | Children with atopic eczema who were admitted to hospital were discharged with a written eczema treatment plan. | 3 | Royal Children's Hospital. 2013.(67) | 8.5  (1) | Consensus-based recommendation | U | Ongoing management | Hosp |
| **FEVER** | | | | | | | | | | |
| 1 | All children presenting with fever (greater than 38 degress Celsius) have the following history documented:  recent antibiotic treatment, AND  GBS status of mother (if neonate aged less than 1 month) AND  fluid intake AND  length of illness (how long the child has been unwell)  recent travel AND  immunisation status AND  direct contact with people who are unwell AND  headache (not in infants / toddlers) AND  diarrhoea and vomiting AND  abdominal pain AND  joint symptoms (not in infants). | FEVE01 | Children with a fever (over 38oC) had all recent antibiotic treatment documented. | 7 | SA Child Health Clinical Network. 2013.(69)  National Institute for Health and Care Excellence. 2013.(70)  Cincinnati Children's Hospital Medical Center. 2010.(71)  The Royal Children's Hospital Melbourne. 2011.(72) | 7.4  (2.3) | Consensus-based recommendation | U | Diagnosis | ED  GP  Hosp  Spec |
|  |  | FEVE02 | Neonates aged <1 month with a fever (over 38oC) had the GBS status of their mother documented. | 7 | SA Child Health Clinical Network. 2013.(69)  National Institute for Health and Care Excellence. 2013.(70)  Cincinnati Children's Hospital Medical Center. 2010.(71)  The Royal Children's Hospital Melbourne. 2011.(72) | 7.9  (1.7) | Consensus-based recommendation | U | Diagnosis | ED  GP  Hosp  Spec |
|  |  | FEVE03 | Children with a fever (over 38oC) had their fluid intake documented. | 7 | SA Child Health Clinical Network. 2013.(69)  National Institute for Health and Care Excellence. 2013.(70)  Cincinnati Children's Hospital Medical Center. 2010.(71)  The Royal Children's Hospital Melbourne. 2011.(72) | 8.4  (1.0) | Consensus-based recommendation | U | Diagnosis | ED  GP  Hosp  Spec |
|  |  | FEVE04 | Children with a fever (over 38oC) had their length of illness documented. | 7 | SA Child Health Clinical Network. 2013.(69)  National Institute for Health and Care Excellence. 2013.(70)  Cincinnati Children's Hospital Medical Center. 2010.(71)  The Royal Children's Hospital Melbourne. 2011.(72) | 8.1  (1.5)) | Consensus-based recommendation | U | Diagnosis | ED  GP  Hosp  Spec |
|  |  | FEVE05 | Children with a fever (over 38oC) had any recent travel documented. | 7 | SA Child Health Clinical Network. 2013.(69)  National Institute for Health and Care Excellence. 2013.(70)  Cincinnati Children's Hospital Medical Center. 2010.(71)  The Royal Children's Hospital Melbourne. 2011.(72) | 7  (1.7) | Consensus-based recommendation | U | Diagnosis | ED  GP  Hosp  Spec |
|  |  | FEVE06 | Children with a fever (over 38oC) had their immunisation status documented. | 7 | SA Child Health Clinical Network. 2013.(69)  National Institute for Health and Care Excellence. 2013.(70)  Cincinnati Children's Hospital Medical Center. 2010.(71)  The Royal Children's Hospital Melbourne. 2011.(72) | 7.9  (1.9) | Consensus-based recommendation | U | Diagnosis | ED  GP  Hosp  Spec |
|  |  | FEVE07 | Children with a fever (over 38oC) had whether they were in direct contact with unwell people documented. | 7 | SA Child Health Clinical Network. 2013.(69)  National Institute for Health and Care Excellence. 2013.(70)  Cincinnati Children's Hospital Medical Center. 2010.(71)  The Royal Children's Hospital Melbourne. 2011.(72) | 7.7  (1.5) | Consensus-based recommendation | U | Diagnosis | ED  GP  Hosp  Spec |
|  |  | FEVE08 | Children with a fever (over 38oC) had the presence of headaches documented. | 7 | SA Child Health Clinical Network. 2013.(69)  National Institute for Health and Care Excellence. 2013.(70)  Cincinnati Children's Hospital Medical Center. 2010.(71)  The Royal Children's Hospital Melbourne. 2011.(72) | 7.1  (2.3) | Consensus-based recommendation | U | Diagnosis | ED  GP  Hosp  Spec |
|  |  | FEVE09 | Children with a fever (over 38oC) had the presence of diarrhoea and vomiting documented. | 7 | SA Child Health Clinical Network. 2013.(69)  National Institute for Health and Care Excellence. 2013.(70)  Cincinnati Children's Hospital Medical Center. 2010.(71)  The Royal Children's Hospital Melbourne. 2011.(72) | 8.3  (1.1) | Consensus-based recommendation | U | Diagnosis | ED  GP  Hosp  Spec |
|  |  | FEVE10 | Children with a fever (over 38oC) had the presence of abdominal pain documented. | 7 | SA Child Health Clinical Network. 2013.(69)  National Institute for Health and Care Excellence. 2013.(70)  Cincinnati Children's Hospital Medical Center. 2010.(71)  The Royal Children's Hospital Melbourne. 2011.(72) | 7.3  (1.4) | Consensus-based recommendation | U | Diagnosis | ED  GP  Hosp  Spec |
|  |  | FEVE11 | Children with a fever (over 38oC) had the presence of joint symptoms documented. | 7 | SA Child Health Clinical Network. 2013.(69)  National Institute for Health and Care Excellence. 2013.(70)  Cincinnati Children's Hospital Medical Center. 2010.(71)  The Royal Children's Hospital Melbourne. 2011.(72) | 7.3  (1.4) | Consensus-based recommendation | U | Diagnosis | ED  GP  Hosp  Spec |
| 2 | All children presenting with fever (greater than 38 degress Celsius) are assessed for the following:  alertness (arousal, alertness or activity decreased) AND  vital signs (Temperature, Heart Rate, Respiratory Rate) AND  airway / breathing / stridor AND  circulation / capillary refill AND  cough AND  coryza AND  photophobia (not in infants / toddlers) AND  neck stiffness AND  rash AND  otitis media (or receive an examination of eardrums). | FEVE12 | Children with a fever (over 38oC) had their alertness assessed. | 7 | National Institute for Health and Care Excellence. 2013.(70)  The Royal Children's Hospital Melbourne. 2011.(72) | 8.9  (0.4) | Level II | U | Diagnosis | ED  GP  Hosp  Spec |
|  |  | FEVE13 | Children with a fever (over 38oC) had their vital signs assessed. | 7 | National Institute for Health and Care Excellence. 2013.(70)  The Royal Children's Hospital Melbourne. 2011.(72) | 8.9  (0.4) | Consensus-based recommendation | U | Diagnosis | ED  GP  Hosp  Spec |
|  |  | FEVE14 | Children with a fever (over 38oC) had their airway, breathing and any signs of stridor assessed. | 7 | National Institute for Health and Care Excellence. 2013.(70)  The Royal Children's Hospital Melbourne. 2011.(72) | 8.9  (0.4) | Level II | U | Diagnosis | ED  GP  Hosp  Spec |
|  |  | FEVE15 | Children with a fever (over 38oC) had their circulation and capillary refill assessed. | 7 | National Institute for Health and Care Excellence. 2013.(70)  The Royal Children's Hospital Melbourne. 2011.(72) | 8.3  (1.0) | Level II | U | Diagnosis | ED  GP  Hosp  Spec |
|  |  | FEVE16 | Children with a fever (over 38oC) had their cough assessed. | 7 | National Institute for Health and Care Excellence. 2013.(70)  The Royal Children's Hospital Melbourne. 2011.(72) | 8  (1.5) | Consensus-based recommendation | U | Diagnosis | ED  GP  Hosp  Spec |
|  |  | FEVE17 | Children with a fever (over 38oC) had their mucous membranes assessed. | 7 | National Institute for Health and Care Excellence. 2013.(70)  The Royal Children's Hospital Melbourne. 2011.(72) | 8.2  (1.6) | Consensus-based recommendation | U | Diagnosis | ED  GP  Hosp  Spec |
|  |  | FEVE18 | Children with a fever (over 38oC) were assessed for photophobia. | 7 | National Institute for Health and Care Excellence. 2013.(70)  The Royal Children's Hospital Melbourne. 2011.(72) | 7.6  (1.9) | Consensus-based recommendation | U | Diagnosis | ED  GP  Hosp  Spec |
|  |  | FEVE19 | Children with a fever (over 38oC) were assessed for the presence of any neck stiffness. | 7 | National Institute for Health and Care Excellence. 2013.(70)  The Royal Children's Hospital Melbourne. 2011.(72) | 7.9  (2.0) | Consensus-based recommendation | U | Diagnosis | ED  GP  Hosp  Spec |
|  |  | FEVE20 | Children with a fever (over 38oC) were assessed for a rash. | 7 | National Institute for Health and Care Excellence. 2013.(70)  The Royal Children's Hospital Melbourne. 2011.(72) | 8.7  (0.5) | Consensus-based recommendation | U | Diagnosis | ED  GP  Hosp  Spec |
|  |  | FEVE21 | Children with a fever (over 38oC) were assessed for otitis media or received an examination of their eardrums. | 7 | National Institute for Health and Care Excellence. 2013.(70)  The Royal Children's Hospital Melbourne. 2011.(72) | 7.7  (3.0) | Consensus-based recommendation | U | Diagnosis | ED  GP  Hosp  Spec |
| 3 | Infants less than 1 month of age presenting to the GP with documented fever (greater than 38 degrees Celsius) are referred to hospital. | FEVE22 | Infants aged < 1 month presenting to the GP with a fever (over 38oC) were referred to hospital. | 7 | SA Child Health Clinical Network. 2013.(69)  National Institute for Health and Care Excellence. 2013.(70)  Cincinnati Children's Hospital Medical Center. 2010.(71)  The Royal Children's Hospital Melbourne. 2011.(72) | 8.4  (1.1) | Consensus-based recommendation | U | Treatment | GP |
| 4 | Infants 0-3 months presenting with fever (greater than 38 degress Celsius) are:  referred to hospital AND  have sepsis work-up AND  receive parenteral antibiotics (aged 0-1 month) | FEVE23 | Infants aged 0-3 months with a fever (over 38oC) were referred to hospital. | 7 | SA Child Health Clinical Network. 2013.(69) | 8.4  (1.1) | Consensus-based recommendation | U | Treatment | GP |
|  |  | FEVE24 | Infants aged 0-3 months with a fever (over 38oC) received a sepsis work-up. | 7 | SA Child Health Clinical Network. 2013.(69) | 7.7  (1.1) | Consensus-based recommendation | U | Treatment | ED  GP  Hosp  Spec |
|  |  | FEVE25 | Infants aged 0-1 months with a fever (over 38oC) received parenteral antibiotics. | 7 | SA Child Health Clinical Network. 2013.(69) | 8.9  (0.4) | Consensus-based recommendation | U | Treatment | ED  GP  Hosp  Spec |
| 5 | Children aged 3 months to 3 years with temperature greater than 38 degrees Celsius who have no clear source of infection, are well appearing and fully immunised are managed as follows:  have urine microscopy AND  discharged home AND  parents advised to have child reviewed if they deteriorate. | FEVE26 | Children aged 3 months to 3 years with a fever (over 38oC) who had no clear source of infection, appeared well and were fully immunised received urine microscopy. | 7 | The Royal Children's Hospital Melbourne. 2011.(72)  NSW Kids and Families. 2010.(73) | 8.3  (0.8) | Consensus-based recommendation | U | Treatment | ED Hosp |
|  |  | FEVE27 | Children aged 3 months to 3 years with a fever (over 38oC) who had no clear source of infection, appeared well and were fully immunised were discharged home. | 7 | The Royal Children's Hospital Melbourne. 2011.(72)  NSW Kids and Families. 2010.(73) | 8  (1.4) | Consensus-based recommendation | U | Treatment | ED Hosp |
|  |  | FEVE28 | Parents of children aged 3 months to 3 years with a fever (over 38oC) who had no clear source of infection, appeared well and were fully immunised were advised to have their child reviewed if they deteriorate. | 7 | The Royal Children's Hospital Melbourne. 2011.(72)  NSW Kids and Families. 2010.(73) | 8.6  (0.8) | Consensus-based recommendation | U | Treatment | ED  GP  Hosp  Spec |
| 6 | Children aged greater than 3 years, presenting with fever (greater than 38 degrees Celsius) without a clinical focus and are well are NOT prescribed antibiotics. | FEVE29 | Children aged > 3years with a fever (over 38oC), no clinical focus and who were well were not prescribed antibiotics. | 7 | NSW Kids and Families. 2010.(73) | 8.7  (0.5) | Consensus-based recommendation | O | Treatment | ED  GP  Hosp  Spec |
| 7 | Infants / children presenting to ED with fever (greater than 38 degrees Celsius) who are shocked OR unrousable OR showing signs of meningococcal disease receive:  immediate antibiotics AND  immediate fluid resuscitation AND  referral to retrieval service or PICU if not responding to treatment | FEVE30 | Infants and children who presented to ED with a fever (over 38oC) who were shocked, unrousable OR showing signs of meningococcal disease received immediate antibiotics. | 7 | NSW Kids and Families. 2010.(73) | 9  (0) | Consensus-based recommendation | U | Treatment | ED Hosp |
|  |  | FEVE31 | Infants and children who presented to ED with a fever (over 38oC) and were shocked, unrousable OR showing signs of meningococcal disease received immediate fluid resuscitation. | 7 | NSW Kids and Families. 2010.(73) | 9  (0) | Consensus-based recommendation | U | Treatment | ED Hosp |
|  |  | FEVE32 | Infants and children who presented to ED with a fever (over 38oC) and were shocked, unrousable OR showing signs of meningococcal disease were referred or retrieved to a PICU. | 7 | NSW Kids and Families. 2010.(73) | 8.3  (1.3) | Consensus-based recommendation | U | Treatment | ED Hosp |
| 8 | Infants / children presenting to a GP with fever (greater than 38 degress Celsius) who are shocked OR unrousable OR showing signs of meningococcal disease receive:  immediate antibiotics AND  transfer to hospital. | FEVE33 | Infants and children who presented to their GP with a fever (over 38oC) and were shocked, unrousable OR showing signs of meningococcal disease received immediate antibiotics. | 7 | NSW Kids and Families. 2010.(73) | 9  (0) | Consensus-based recommendation | U | Treatment | GP |
|  |  | FEVE34 | Infants and children who presented to their GP with a fever (over 38oC) and were shocked, unrousable OR showing signs of meningococcal disease were transferred to hospital. | 7 | NSW Kids and Families. 2010.(73) | 9  (0) | Consensus-based recommendation | U | Treatment | GP |
| 9 | Infants aged less than 3 months of age presenting to the ED with fever (greater than 38 degress Celsius) have the following investigations performed:  CBE with differential and CRP AND  Blood culture AND  Urinalysis and culture (SPA / catheter) | FEVE35 | Infants aged < 3 months who presented to the ED with a fever (over 38oC) had a CBE (with differential) and CRP performed. | 7 | SA Child Health Clinical Network. 2013.(69)  National Institute for Health and Care Excellence. 2013.(70)  Cincinnati Children's Hospital Medical Center. 2010.(71)  NSW Kids and Families. 2010.(73) | 7.3  (1.9) | Consensus-based recommendation | U | Treatment | ED Hosp |
|  |  | FEVE36 | Infants aged < 3 months who presented to the ED with a fever (over 38oC) had a blood cultures taken. | 7 | SA Child Health Clinical Network. 2013.(69)  National Institute for Health and Care Excellence. 2013.(70)  Cincinnati Children's Hospital Medical Center. 2010.(71)  NSW Kids and Families. 2010.(73) | 8.8  (0.4) | Consensus-based recommendation | U | Treatment | ED Hosp |
|  |  | FEVE37 | Infants aged < 3 months who presented to the ED with a fever (over 38oC) had a urinalysis with culture performed. | 7 | SA Child Health Clinical Network. 2013.(69)  National Institute for Health and Care Excellence. 2013.(70)  Cincinnati Children's Hospital Medical Center. 2010.(71)  NSW Kids and Families. 2010.(73) | 9  (0) | Consensus-based recommendation | U | Treatment | ED Hosp |
| 10 | Children with a fever (greater than 38 degress Celsius) who are toxic or unwell and no focus of infection:  have a blood count performed AND  have blood cultures taken at the same time. | FEVE38 | Children with a fever (over 38oC) who were toxic or unwell and had no focus of infection had a blood count (CBE) performed. | 7 | NSW Kids and Families. 2010.(73) | 7.7  (2.0) | Consensus-based recommendation | U | Treatment | ED  GP  Hosp  Spec |
|  |  | FEVE39 | Children with a fever (over 38oC) who were toxic or unwell and had no focus of infection had a blood cultures taken at the same time as other blood tests. | 7 | NSW Kids and Families. 2010.(73) | 8.1  (1.2) | Consensus-based recommendation | U | Treatment | ED  GP  Hosp  Spec |
| 11 | Children aged 3 months to 3 years with temperature greater than 38 degress Celsius showing signs of shock and where there is no clear source of infection are managed as follows:  venous blood gas AND  blood culture AND  SPA or catheter urine AND  CXR (if respiratory symptoms / signs) AND  empiric IV antibiotics AND  fluid resuscitation. | FEVE40 | Children aged 3 months to 3 years with a fever (> 38oC) who showed signs of shock and had no clear source of infection had a venous blood gas taken. | 7 | SA Child Health Clinical Network. 2013.(69)  NSW Kids and Families. 2010.(73) | 7.2  (2.8) | Consensus-based recommendation | U | Treatment | ED  GP  Hosp  Spec |
|  |  | FEVE41 | Children aged 3 months to 3 years with a fever (> 38oC) who showed signs of shock and had no clear source of infection had blood cultures taken. | 7 | SA Child Health Clinical Network. 2013.(69)  NSW Kids and Families. 2010.(73) | 7.7  (1.5) | Consensus-based recommendation | U | Treatment | ED  GP  Hosp  Spec |
|  |  | FEVE42 | Children aged 3 months to 3 years with a fever (> 38oC) who showed signs of shock and had no clear source of infection had urine sample taken. | 7 | SA Child Health Clinical Network. 2013.(69)  NSW Kids and Families. 2010.(73) | 8  (1.3) | Consensus-based recommendation | U | Treatment | ED  GP  Hosp  Spec |
|  |  | FEVE43 | Children aged 3 months to 3 years with a fever (> 38oC) who showed signs of shock and had no clear source of infection but with respiratory symptoms/signs had a chest x-ray taken. | 7 | SA Child Health Clinical Network. 2013.(69)  NSW Kids and Families. 2010.(73) | 7.7  (1.5) | Consensus-based recommendation | U | Treatment | ED  GP  Hosp  Spec |
|  |  | FEVE44 | Children aged 3 months to 3 years with a fever (> 38oC) who showed signs of shock and had no clear source of infection were admitted to hospital for empiric IV antibiotics. | 7 | SA Child Health Clinical Network. 2013.(69)  NSW Kids and Families. 2010.(73) | 8.9  (0.4) | Consensus-based recommendation | U | Treatment | ED  GP  Hosp  Spec |
|  |  | FEVE45 | Children aged 3 months to 3 years with a fever (> 38oC) who showed signs of shock and had no clear source of infection were admitted to hospital for fluid resuscitation. | 7 | SA Child Health Clinical Network. 2013.(69)  NSW Kids and Families. 2010.(73) | 8.7  (0.5) | Consensus-based recommendation | U | Treatment | ED  GP  Hosp  Spec |
| 12 | All children presenting with fever suspected of having a urinary tract infection have a urine culture before commencing antibiotics. | FEVE46 | Children with a fever (over 38oC) where a UTI was suspected had a urine culture taken before commencing antibiotics. | 7 | NSW Kids and Families. 2010.(73) | 8.8  (0.4) | Consensus-based recommendation | U | Treatment | ED  GP  Hosp  Spec |
| 13 | Children with a fever (greater than 38 degress Celsius) who are discharged receive a fever fact sheet. | FEVE47 | Parents of children with a fever (over 38oC) who were discharged received a fever fact sheet. | 7 | NSW Kids and Families. 2010.(73) | 8.2  (1.3) | Consensus-based recommendation | U | Ongoing management | ED Hosp |
| **GASTRO-OESOPHAGEAL REFLUX DISORDER** | | | | | | | | | | |
| 1 | Infants / Children presenting with regurgitation have the following documented:  weight and height (growth chart) AND  allergies (skin rash / urticaria / eczema / diarrhoea / perineal / perianal excoriation), food and milk intolerances (cow's milk) AND  history of regurgitation / vomiting, cough, epigastric pain / heartburn (children aged 6-17 years). | GORD01 | Infants / Children who presented with regurgitation had their weight and height (growth chart) documented. | 1 | Queensland Government Children's Health Service Royal Children's Hospital. 2011.(74)  Queensland Government Children's Health Service Royal Children's Hospital. 2011.(75)  The Royal Children's Hospital Melbourne. 2012.(76)  Vandenplas Y, Rudolph CD, Di Lorenzo C, et al. 2009.(77)  Lightdale JR, Gremse DA, Heitlinger LA, et al. 2013.(78) | 7 | Consensus-based recommendation | U | Diagnosis | ED  GP  Hosp  Spec |
|  |  | GORD02 | Infants / Children who presented with regurgitation had their allergies (skin rash / urticaria / eczema / diarrhoea / perineal / perianal excoriation), food and milk intolerances (cow's milk) documented. | 1 | Queensland Government Children's Health Service Royal Children's Hospital. 2011.(74)  Queensland Government Children's Health Service Royal Children's Hospital. 2011.(75)  The Royal Children's Hospital Melbourne. 2012.(76)  Vandenplas Y, Rudolph CD, Di Lorenzo C, et al. 2009.(77)  Lightdale JR, Gremse DA, Heitlinger LA, et al. 2013.(78) | 7 | Consensus-based recommendation | U | Diagnosis | ED  GP  Hosp  Spec |
|  |  | GORD03 | Infants / Children aged 6-17 years who presented with regurgitation had their history of regurgitation / vomiting, cough, epigastric pain / heartburn documented. | 1 | Queensland Government Children's Health Service Royal Children's Hospital. 2011.(74)  Queensland Government Children's Health Service Royal Children's Hospital. 2011.(75)  The Royal Children's Hospital Melbourne. 2012.(76)  Vandenplas Y, Rudolph CD, Di Lorenzo C, et al. 2009.(77)  Lightdale JR, Gremse DA, Heitlinger LA, et al. 2013.(78) | 8 | Consensus-based recommendation | U | Diagnosis | ED  GP  Hosp  Spec |
| 2 | Infants / children presenting with any of the following have their weight and height (growth chart) recorded AND receive a urine MC&S:  - history of food refusal OR  - regurgitation / vomiting. | GORD04 | Infants / children who presented with a history of food refusal OR regurgitation / vomiting, had their weight and height (growth chart) recorded. | 1 | Queensland Government Children's Health Service Royal Children's Hospital. 2011.(74)  Queensland Government Children's Health Service Royal Children's Hospital. 2011.(75)  The Royal Children's Hospital Melbourne. 2012.(76)  Vandenplas Y, Rudolph CD, Di Lorenzo C, et al. 2009.(77)  Lightdale JR, Gremse DA, Heitlinger LA, et al. 2013.(78) | 7 | Consensus-based recommendation | U | Diagnosis | ED  GP  Hosp  Spec |
|  |  | GORD05 | Infants / children who presented with a history of food refusal OR regurgitation / vomiting, received a urine MC&S. | 1 | Queensland Government Children's Health Service Royal Children's Hospital. 2011.(74)  Queensland Government Children's Health Service Royal Children's Hospital. 2011.(75)  The Royal Children's Hospital Melbourne. 2012.(76)  Vandenplas Y, Rudolph CD, Di Lorenzo C, et al. 2009.(77)  Lightdale JR, Gremse DA, Heitlinger LA, et al. 2013.(78) | 7 | Consensus-based recommendation | U | Diagnosis | ED  GP  Hosp  Spec |
| 3 | Infants (aged less than 12 months) with recurrent regurgitation and poor weight gain despite adequate energy intake have the following assessed to determine a diagnosis:  diet history AND  urinalysis AND  complete blood count AND  serum electrolytes AND  Blood Urea Nitrogen AND  serum creatinine. | GORD06 | Infants aged less than 12 months with recurrent regurgitation and poor weight gain despite adequate energy intake had their diet history assessed. | 1 | Vandenplas Y, Rudolph CD, Di Lorenzo C, et al. 2009.(77) | 8 | Grade D | U | Diagnosis | ED  GP  Hosp  Spec |
|  |  | GORD07 | Infants aged less than 12 months with recurrent regurgitation and poor weight gain despite adequate energy intake received a urinalysis. | 1 | Vandenplas Y, Rudolph CD, Di Lorenzo C, et al. 2009.(77) | 8 | Grade D | U | Diagnosis | ED  GP  Hosp  Spec |
|  |  | GORD08 | Infants aged less than 12 months with recurrent regurgitation and poor weight gain despite adequate energy intake received a complete blood count. | 1 | Vandenplas Y, Rudolph CD, Di Lorenzo C, et al. 2009.(77) | 8 | Grade D | U | Diagnosis | ED  GP  Hosp  Spec |
|  |  | GORD09 | Infants aged less than 12 months with recurrent regurgitation and poor weight gain despite adequate energy intake had their serum electrolytes assessed. | 1 | Vandenplas Y, Rudolph CD, Di Lorenzo C, et al. 2009.(77) | 8 | Grade D | U | Diagnosis | ED  GP  Hosp  Spec |
|  |  | GORD10 | Infants aged less than 12 months with recurrent regurgitation and poor weight gain despite adequate energy intake had their Blood Urea Nitrogen assessed. | 1 | Vandenplas Y, Rudolph CD, Di Lorenzo C, et al. 2009.(77) | 7 | Grade D | U | Diagnosis | ED  GP  Hosp  Spec |
|  |  | GORD11 | Infants aged less than 12 months with recurrent regurgitation and poor weight gain despite adequate energy intake had their serum creatinine assessed. | 1 | Vandenplas Y, Rudolph CD, Di Lorenzo C, et al. 2009.(77) | 8 | Grade D | U | Diagnosis | ED  GP  Hosp  Spec |
| 4 | Infants who have uncomplicated recurrent regurgitation ("happy spitters") have the following treatment:  feeding and feeding practices reviewed AND  parental reassurance and education provided. | GORD12 | Infants who had uncomplicated recurrent regurgitation “happy spitters” had their feeding and feeding practices reviewed. | 1 | Queensland Government Children's Health Service Royal Children's Hospital. 2011.(75)  Vandenplas Y, Rudolph CD, Di Lorenzo C, et al. 2009.(77)  Royal Children's Hospital. 2009.(49)  Bhavsar H, Cullen M, Beattie RM. 2011.(79) | 9 | Grade C | U | Treatment | ED  GP  Hosp  Spec |
|  |  | GORD13 | Infants who had uncomplicated recurrent regurgitation "happy spitters" were provided with parental reassurance and education. | 1 | Queensland Government Children's Health Service Royal Children's Hospital. 2011.(75)  Vandenplas Y, Rudolph CD, Di Lorenzo C, et al. 2009.(77)  Royal Children's Hospital. 2009.(49)  Bhavsar H, Cullen M, Beattie RM. 2011.(79) | 9 | Grade C | U | Treatment | ED  GP  Hosp  Spec |
| 5 | Infants / children presenting with uncomplicated recurrent regurgitation should NOT have a barium swallow and meal to confirm the diagnosis of GORD. | GORD14 | Infants / children who presented with uncomplicated recurrent regurgitation did not have a barium swallow and meal. | 1 | Lightdale JR, Gremse DA, Heitlinger LA, et al. 2013.(78) | 9 | Consensus-based recommendation | O | Treatment | ED  GP  Hosp  Spec |
| 6 | Children aged greater than 18 months who present with dysphagia or odynophagia are referred to a paediatric gastroenterologist. | GORD15 | Children aged greater than 18 months who presented with dysphagia or odynophagia were referred to a paediatric gastroenterologist. | 1 | Vandenplas Y, Rudolph CD, Di Lorenzo C, et al. 2009.(77) | 7 | Consensus-based recommendation | U | Treatment | ED  GP  Hosp  Spec |
| 7 | Children aged greater than 18 months who present with dysphagia or odynophagia receive a barium swallow. | GORD16 | Children aged greater than 18 months who presented with dysphagia or odynophagia received a barium swallow. | 1 | Vandenplas Y, Rudolph CD, Di Lorenzo C, et al. 2009.(77) | 7 | Grade D | U | Treatment | ED  GP  Hosp  Spec |
| 8 | Infants with reflux who are healthy and thriving and who present with any of the following are NOT prescribed acid suppression medication at the first presentation:  - irritability or unexplained crying OR  - feeding refusal OR  - frequent regurgitation. | GORD17 | Infants with reflux who were healthy and thriving and presented with irritability or unexplained crying were not prescribed acid suppression medication at the first presentation. | 1 | Vandenplas Y, Rudolph CD, Di Lorenzo C, et al. 2009.(77)  Royal Children's Hospital. 2009.(49)  Allen K. 2012.(80) | 9 | Consensus-based recommendation | O | Treatment | ED  GP  Hosp  Spec |
|  |  | GORD18 | Infants with reflux who were healthy and thriving and presented with feeding refusal were not prescribed acid suppression medication at the first presentation. | 1 | Vandenplas Y, Rudolph CD, Di Lorenzo C, et al. 2009.(77)  Royal Children's Hospital. 2009.(49)  Allen K. 2012.(80) | 9 | Consensus-based recommendation | O | Treatment | ED  GP  Hosp  Spec |
|  |  | GORD19 | Infants with reflux who were healthy and thriving and presented with frequent regurgitation were not prescribed acid suppression medication at the first presentation. | 1 | Vandenplas Y, Rudolph CD, Di Lorenzo C, et al. 2009.(77)  Royal Children's Hospital. 2009.(49)  Allen K. 2012.(80) | 9 | Consensus-based recommendation | O | Treatment | ED  GP  Hosp  Spec |
| 9 | Children with Barrett's Oesophagus have multiple biopsies obtained at time of endoscopy to confirm the diagnosis. | GORD20 | Children with Barrett's Oesophagus had multiple biopsies obtained at time of endoscopy. | 1 | Vandenplas Y, Rudolph CD, Di Lorenzo C, et al. 2009.(77) | 9 | Consensus-based recommendation | U | Treatment | ED  GP  Hosp  Spec |
| 10 | Children with Barrett's Oesophagus are prescribed acid suppression. | GORD21 | Children with Barrett's Oesophagus were prescribed acid suppression. | 1 | Vandenplas Y, Rudolph CD, Di Lorenzo C, et al. 2009.(77) | 9 | Consensus-based recommendation | U | Treatment | ED  GP  Hosp  Spec |
| 11 | Older children / adolescents presenting with heartburn are managed as follows:  assessed for lifestyle factors (diet, alcohol, weight, sleeping position, smoking) AND  prescribed a PPI for 4 weeks. | GORD22 | Older children / adolescents who presented with heartburn were assessed for lifestyle factors (diet, alcohol, weight, sleeping position, smoking). | 1 | Vandenplas Y, Rudolph CD, Di Lorenzo C, et al. 2009.(77) | 8 | Grade A | U | Treatment | ED  GP  Hosp  Spec |
|  |  | GORD23 | Older children / adolescents who presented with heartburn were prescribed a PPI for 4 weeks. | 1 | Vandenplas Y, Rudolph CD, Di Lorenzo C, et al. 2009.(77) | 8 | Grade A | U | Ongoing management | ED  GP  Hosp  Spec |
| 12 | Older children / adolescents presenting with heartburn who have been prescribed and used a PPI for 4 weeks are reviewed by their GP and if:  symptoms resolved / improved, continue PPI for 3 months OR  recurrent / persistent symptoms, they are referred to a gastroenterologist. | GORD24 | Older children / adolescents who presented with heartburn, had been prescribed and used a PPI for 4 weeks, and their symptoms had resolved/improved were reviewed by their GP and had their PPI continued for 3 months. | 1 | Vandenplas Y, Rudolph CD, Di Lorenzo C, et al. 2009.(77) | 8 | Grade D | U | Ongoing management | GP |
|  |  | GORD25 | Older children / adolescents who presented with heartburn, had been prescribed and used a PPI for 4 weeks, and they had recurrent / persistent symptoms were reviewed by their GP and referred to a gastroenterologist. | 1 | Vandenplas Y, Rudolph CD, Di Lorenzo C, et al. 2009.(77) | 8 | Grade D | U | Ongoing management | GP |
| 13 | Infants / Children (aged less than 18 months) with reflux oesophagitis are managed as follows:  family lifestyle factors recorded (diet, alcohol, weight, sleeping position, smoking) AND  have symptoms reassessed at each review. | GORD26 | Infants / Children (aged less than 18 months) with reflux oesophagitis had their family lifestyle factors recorded (diet, alcohol, weight, sleeping position, smoking). | 1 | Vandenplas Y, Rudolph CD, Di Lorenzo C, et al. 2009.(77) | 7 | Grade A | U | Ongoing management | ED  GP  Hosp  Spec |
|  |  | GORD27 | Infants / Children (aged less than 18 months) with reflux oesophagitis had their symptoms reassessed at each review. | 1 | Vandenplas Y, Rudolph CD, Di Lorenzo C, et al. 2009.(77) | 9 | Grade A | U | Ongoing management | ED  GP  Hosp  Spec |
| 14 | Infants / children who meet any of the following criteria are referred to a paediatric gastroenterologist:  presence of warning signs (see definition) OR  difficulty swallowing / history of obstruction OR  weight loss / anorexia / poor feeding OR  symptoms persist during and after PPI therapy. | GORD28 | Infants / children who had the presence of warning signs (see definition) were referred to a paediatric gastroenterologist. | 1 | Queensland Government Children's Health Service Royal Children's Hospital. 2011.(74)  Queensland Government Children's Health Service Royal Children's Hospital. 2011.(75)  Vandenplas Y, Rudolph CD, Di Lorenzo C, et al. 2009.(77) | 9 | Grade A | U | Ongoing management | ED  GP  Hosp  Spec |
|  |  | GORD29 | Infants / children who had difficulty swallowing or a history of obstruction were referred to a paediatric gastroenterologist. | 1 | Queensland Government Children's Health Service Royal Children's Hospital. 2011.(74)  Queensland Government Children's Health Service Royal Children's Hospital. 2011.(75)  Vandenplas Y, Rudolph CD, Di Lorenzo C, et al. 2009.(77) | 9 | Consensus-based recommendation | U | Ongoing management | ED  GP  Hosp  Spec |
|  |  | GORD30 | Infants / children who had weight loss / anorexia / poor feeding were referred to a paediatric gastroenterologist. | 1 | Queensland Government Children's Health Service Royal Children's Hospital. 2011.(74)  Queensland Government Children's Health Service Royal Children's Hospital. 2011.(75)  Vandenplas Y, Rudolph CD, Di Lorenzo C, et al. 2009.(77) | 7 | Consensus-based recommendation | U | Ongoing management | ED  GP  Hosp  Spec |
|  |  | GORD31 | Infants / children whose symptoms persisted during and after PPI therapy were referred to a paediatric gastroenterologist. | 1 | Queensland Government Children's Health Service Royal Children's Hospital. 2011.(74)  Queensland Government Children's Health Service Royal Children's Hospital. 2011.(75)  Vandenplas Y, Rudolph CD, Di Lorenzo C, et al. 2009.(77) | 9 | Consensus-based recommendation | U | Ongoing management | ED  GP  Hosp  Spec |
| 15 | Infants / Children with uncomplicated recurrent regurgitation who present with any of the following are immediately referred to a hospital emergency department:  projectile vomiting OR  haematemesis OR  bile-stained vomiting. | GORD32 | Infants / Children with uncomplicated recurrent regurgitation who presented with projectile vomiting OR haematemesis OR bile-stained vomiting were immediately referred to a hospital emergency department. | 1 | Queensland Government Children's Health Service Royal Children's Hospital. 2011.(74)  Queensland Government Children's Health Service Royal Children's Hospital. 2011.(75) | 9 | Consensus-based recommendation | U | Ongoing management | ED  GP  Hosp  Spec |
| **HEAD INJURY** | | | | | | | | | | |
| 1 | Children presenting with a head injury and any of the following are categorised as a Triage 1 patient:  - unconscious / responding only to pain OR  - fitting OR  - signs of cardiovascular compromise | HEAD01 | Children who presented with a head injury and any of the following: - unconscious / responding only to pain OR - fitting OR - signs of cardiovascular compromise were categorised as a Triage 1 patient. | 4 | NSW Kids and Families. 2011.(81) | 8.5  (1) | Consensus-based recommendation | U | Diagnosis | ED Hosp |
| 2 | Children presenting with a head injury and any of the following are categorised as a Triage 2 patient:  Abnormal drowsiness / responding only to voice OR"  Loss of consciousness of more than 5 minutes OR  Focal signs OR  Severe pain or headache OR  High risk mechanism. | HEAD02 | Children who presented with a head injury and any of the following: - abnormal drowsiness / responding only to voice OR - loss of consciousness of more than 5 minutes OR - focal signs OR - severe pain or headache OR - high risk mechanism were categorised as a Triage 2 patient. | 3 | NSW Kids and Families. 2011.(81) | 8.3  (1.2) | Consensus-based recommendation | U | Diagnosis | ED Hosp |
| 3 | Children presenting with a head injury and any of the following are categorised as a Triage 3 patient:  Alert but altered behaviour OR  Loss of consciousness less than 5 minutes OR  Moderate pain or headache OR  Moderate risk mechanism OR  Significant neurological, developmental or bleeding co-morbidities OR  Less than one year of age OR  Possible inflicted head injury, otherwise well. | HEAD03 | Children who presented with a head injury and any of the following: - alert but altered behaviour OR - loss of consciousness less than 5 minutes OR - moderate pain or headache OR - moderate risk mechanism OR - significant neurological, developmental or bleeding co-morbidities OR - less than one year of age OR - possible inflicted head injury, otherwise well were categorised as a Triage 3 patient. | 3 |  | 7  (1.7) | Consensus-based recommendation | U | Diagnosis | ED Hosp |
| 4 | Children presenting with an acute head injury and ONLY the following features are categorised as a Triage 4 or 5 patient:  No neurological signs or symptoms AND"  Low impact mechanism AND  No co-morbidities or concerns regarding inflicted head injury AND  More than one year of age. | HEAD04 | Children aged > 12 months who presented with an acute head injury and ONLY the following features: - low impact mechanism AND - NO neurological signs or symptoms AND - NO co-morbidities or concerns regarding inflicted head injury were categorised as a Triage 4 or 5 patient. | 4 |  | 8.8  (0.5) | Consensus-based recommendation | U | Diagnosis | ED Hosp |
| 5 | Children presenting with a moderate to severe head injury (GCS 3-13) receive a primary survey which includes an assessment of the following:  Airway (with cervical spine immobilisation) AND  breathing function AND  circulation AND  Pupil size and reaction to light, AND  GCS or AVPU, AND  blood glucose. | HEAD05 | Children who presented with a moderate to severe head injury (GCS 3-13) received a primary survey and assessment of their airway (with cervical spine immobilisation). | 4 |  | 8.5  (0.6) | Consensus-based recommendation | U | Treatment | ED  GP  Hosp |
|  |  | HEAD06 | Children who presented with a moderate to severe head injury (GCS 3-13) received a primary survey and assessment of their breathing function. | 4 | NSW Kids and Families. 2011.(81)  The Royal Children's Hospital Melbourne.(82) | 8.5  (0.6) | Consensus-based recommendation | U | Treatment | ED  GP  Hosp |
|  |  | HEAD07 | Children who presented with a moderate to severe head injury (GCS 3-13) received a primary survey and assessment of their circulation. | 4 | NSW Kids and Families. 2011.(81)  The Royal Children's Hospital Melbourne.(82) | 8.5  (0.6) | Consensus-based recommendation | U | Treatment | ED  GP  Hosp |
|  |  | HEAD08 | Children who presented with a moderate to severe head injury (GCS 3-13) received a primary survey and assessment of their pupil size and reaction to light. | 4 | NSW Kids and Families. 2011.(81)  The Royal Children's Hospital Melbourne.(82) | 7.8  (1.5) | Consensus-based recommendation | U | Treatment | ED  GP  Hosp |
|  |  | HEAD09 | Children who presented with a moderate to severe head injury (GCS 3-13) received a primary survey and assessment of their GCS or AVPU. | 5 | NSW Kids and Families. 2011.(81)  The Royal Children's Hospital Melbourne.(82) | 8.4  (0.9) | Consensus-based recommendation | U | Treatment | ED  GP  Hosp |
|  |  | HEAD10 | Children who presented with a moderate to severe head injury (GCS 3-13) received a primary survey and assessment of their blood glucose. | 4 | NSW Kids and Families. 2011.(81)  The Royal Children's Hospital Melbourne.(82) | 7.8  (1.9) | Consensus-based recommendation | U | Treatment | ED  GP  Hosp |
| 6 | Children presenting with a moderate to severe head injury (GCS 3-13) receive a secondary survey which includes:  palpation for bogginess, swelling or bruising of the scalp, AND  looking for signs of base of skull fracture such as Battle's sign (bruising over mastoid), 'raccoon' eyes or blood behind the ear drum, AND  examination for haemo-tympanum or signs of CSF leak from ears or nose AND  facial (e.g. nose, mouth, ears) deformities, swelling, bleeding, lacerations, tenderness AND  cervical spine deformity, tenderness, muscle spasm, crepitus, motor function, reflexes and lateralising signs. | HEAD11 | Children who presented with a moderate to severe head injury (GCS 3-13) received a secondary survey which included palpation for bogginess, swelling or bruising of the scalp. | 4 | NSW Kids and Families. 2011.(81)  The Royal Children's Hospital Melbourne.(82) | 7.8  (1.9) | Consensus-based recommendation | U | Treatment | ED  GP  Hosp |
|  |  | HEAD12 | Children who presented with a moderate to severe head injury (GCS 3-13) received a secondary survey which included looking for signs of base of skull fracture such as Battle's sign (bruising over mastoid), 'raccoon' eyes or blood behind the ear drum. | 4 | NSW Kids and Families. 2011.(81)  The Royal Children's Hospital Melbourne.(82) | 7.5  (1.9) | Consensus-based recommendation | U | Treatment | ED  GP  Hosp |
|  |  | HEAD13 | Children who presented with a moderate to severe head injury (GCS 3-13) received a secondary survey which included examination for haemo-tympanum or signs of CSF leak from ears or nose. | 4 | NSW Kids and Families. 2011.(81)  The Royal Children's Hospital Melbourne.(82) | 7.8  (1.9) | Consensus-based recommendation | U | Treatment | ED  GP  Hosp |
|  |  | HEAD14 | Children who presented with a moderate to severe head injury (GCS 3-13) received a secondary survey which included an examination for facial (e.g. nose, mouth, ears) deformities, swelling, bleeding, lacerations, tenderness. | 4 | NSW Kids and Families. 2011.(81)  The Royal Children's Hospital Melbourne.(82) | 7.5  (1.9) | Consensus-based recommendation | U | Treatment | ED  GP  Hosp |
|  |  | HEAD15 | Children who presented with a moderate to severe head injury (GCS 3-13) received a secondary survey which included examination for cervical spine deformity, tenderness, muscle spasm, crepitus, motor function, reflexes and lateralising signs. | 4 | NSW Kids and Families. 2011.(81)  The Royal Children's Hospital Melbourne.(82) | 7.8  (1.9) | Consensus-based recommendation | U | Treatment | ED  GP  Hosp |
| 7 | Children presenting with a head injury have a thorough history documented which includes:  time of injury AND  mechanism of injury, AND  recall of events, AND  loss or impairment of consciousness (and duration), AND  presence / absence of seizures, AND  behaviour and activity since time of injury AND  nausea and vomiting AND  clinical course prior to consultation, e.g. stable, deteriorating, improving AND  other injuries sustained, AND  comorbidities that predispose to intracranial injury (intra-cerebral shunt, AV malformation, bleeding disorders (including vitamin K deficiency)). | HEAD16 | Children who presented with a head injury had their history documented which included the time of injury. | 4 | NSW Kids and Families. 2011.(81)  The Royal Children's Hospital Melbourne.(82) | 7.5  (1.7) | Consensus-based recommendation | U | Treatment | ED  GP  Hosp |
|  |  | HEAD17 | Children who presented with a head injury had their history documented which included mechanism of injury. | 4 | NSW Kids and Families. 2011.(81)  The Royal Children's Hospital Melbourne.(82) | 7.8  (1.9) | Consensus-based recommendation | U | Treatment | ED  GP  Hosp |
|  |  | HEAD18 | Children who presented with a head injury had their history documented which included a recall of events. | 4 | NSW Kids and Families. 2011.(81)  The Royal Children's Hospital Melbourne.(82) | 7.5  (1.9) | Consensus-based recommendation | U | Treatment | ED  GP  Hosp |
|  |  | HEAD19 | Children who presented with a head injury had their history documented which included whether there was loss or impairment of consciousness (and duration). | 4 | NSW Kids and Families. 2011.(81)  The Royal Children's Hospital Melbourne.(82) | 8.5  (0.6) | Consensus-based recommendation | U | Treatment | ED  GP  Hosp |
|  |  | HEAD20 | Children who presented with a head injury had their history documented which included the presence / absence of seizures. | 4 | NSW Kids and Families. 2011.(81)  The Royal Children's Hospital Melbourne.(82) | 8.5  (0.6) | Consensus-based recommendation | U | Treatment | ED  GP  Hosp |
|  |  | HEAD21 | Children who presented with a head injury had their history documented which included their behaviour and activity since the time of injury. | 4 | NSW Kids and Families. 2011.(81)  The Royal Children's Hospital Melbourne.(82) | 8.5  (0.6) | Consensus-based recommendation | U | Treatment | ED  GP  Hosp |
|  |  | HEAD22 | Children who presented with a head injury had their history documented which included whether they had any nausea or vomiting. | 4 | NSW Kids and Families. 2011.(81)  The Royal Children's Hospital Melbourne.(82) | 8  (1.4) | Consensus-based recommendation | U | Treatment | ED  GP  Hosp |
|  |  | HEAD23 | Children who presented with a head injury had their history documented which included their clinical course prior to consultation, e.g. stable, deteriorating, improving. | 4 | NSW Kids and Families. 2011.(81)  The Royal Children's Hospital Melbourne.(82) | 8.8  (0.5) | Consensus-based recommendation | U | Treatment | ED  GP  Hosp |
|  |  | HEAD24 | Children who presented with a head injury had their history documented which included any other injuries sustained. | 4 | NSW Kids and Families. 2011.(81)  The Royal Children's Hospital Melbourne.(82) | 8.5  (0.6) | Consensus-based recommendation | U | Treatment | ED  GP  Hosp |
|  |  | HEAD25 | Children who presented with a head injury had their history documented which included comorbidities that predispose to intracranial injury (intra-cerebral shunt, AV malformation, bleeding disorders (including vitamin K deficiency)). | 4 | NSW Kids and Families. 2011.(81)  The Royal Children's Hospital Melbourne.(82) | 9  (0) | Consensus-based recommendation | U | Treatment | ED  GP  Hosp |
| 8 | Children presenting to the ED with a head injury and any of the following are classified as severe and should be intubated and ventilated:  GCS persistently less than or equal to 8 OR  Loss of protective laryngeal reflexes OR  Abnormal breathing pattern or hypoventilation OR  Oxygen saturation less than or equal to 95% or a PaO2 less than 80mmHg on maximal facial oxygen OR  PaCO2 less than 30mmHg or PaCO2 greater than 44mmHg. | HEAD26 | Children who presented to the ED with a head injury and any of the following: - GCS persistently less than or equal to 8 OR - loss of protective larygeal reflexes OR - abnormal breathing pattern or hypoventilation OR - oxygen saturation less than or equal to SpO2 95% or a PaO2 less than 80mmHg on maximal facial oxygen OR - PaCO2 less than 30mmHg or PaCO2 greater than 44mmHg were classified as severe and were intubated and ventilated. | 4 | NSW Kids and Families. 2011.(81)  The Royal Children's Hospital Melbourne.(82) | 8.8  (0.5) | Consensus-based recommendation | U | Treatment | ED |
| 9 | Children with a severe head injury (GCS 3-8) receive:  immobilisation of cervical spine AND  nursed 20-30 degrees head up (once fluid resuscitation complete) AND  continuous cardio-respiratory (respiratory rate, pulse) and oxygen saturation monitoring AND  BP measured every 15-30mins AND  GCS recorded every 15-30mins unless intubated AND  an urgent head CT AND  an urgent c-spine CT AND  a consultation with ICU and neurosurgical specialists. | HEAD27 | Children with a severe head injury (GCS 3-8) received immobilisation of their cervical spine. | 4 | The Royal Children's Hospital Melbourne.(82) | 7.5  (1.7) | Consensus-based recommendation | U | Treatment | ED Hosp |
|  |  | HEAD28 | Children with a severe head injury (GCS 3-8) who had completed their fluid resuscitation, were nursed 20-30 degrees head up. | 4 | The Royal Children's Hospital Melbourne.(82) | 8.5  (0.6) | Consensus-based recommendation | U | Treatment | ED Hosp |
|  |  | HEAD29 | Children with a severe head injury (GCS 3-8) received continuous cardio-respiratory (respiratory rate, pulse) and oxygen saturation monitoring. | 4 | The Royal Children's Hospital Melbourne.(82) | 8.5  (0.6) | Consensus-based recommendation | U | Treatment | ED Hosp |
|  |  | HEAD30 | Children with a severe head injury (GCS 3-8) had their BP measured every 15-30 minutes. | 4 | The Royal Children's Hospital Melbourne.(82) | 8.5  (0.6) | Consensus-based recommendation | U | Treatment | ED Hosp |
|  |  | HEAD31 | Children with a severe head injury (GCS 3-8) who were not intubated, had their GCS recorded every 15-30 minutes. | 5 | The Royal Children's Hospital Melbourne.(82) | 8  (1.2) | Consensus-based recommendation | U | Treatment | ED Hosp |
|  |  | HEAD32 | Children with a severe head injury (GCS 3-8) received an urgent CT head. | 4 | The Royal Children's Hospital Melbourne.(82) | 8.8  (0.5) | Grade B | U | Treatment | ED Hosp |
|  |  | HEAD33 | Children with a severe head injury (GCS 3-8) received an urgent C-Spine CT. | 4 | The Royal Children's Hospital Melbourne.(82) | 8.5  (0.6) | Consensus-based recommendation | U | Treatment | ED Hosp |
|  |  | HEAD34 | Children with a severe head injury (GCS 3-8) received a consultation with ICU and neurosurgical specialists. | 4 | The Royal Children's Hospital Melbourne.(82) | 8.4  (0.5) | Consensus-based recommendation | U | Treatment | ED Hosp |
| 10 | Children presenting with moderate head injury (GCS 9-13) without neurological deterioration are observed in hospital at least half-hourly for a minimum of four hours including:  GCS, AND  pulse rate, AND  respiratory rate, AND  blood pressure, AND  pupil assessment, AND  limb strength. | HEAD35 | Children who presented with moderate head injury (GCS 9-13) without neurological deterioration had their GCS observed in hospital at least half-hourly for a minimum of four hours. | 4 | NSW Kids and Families. 2011.(81)  The Royal Children's Hospital Melbourne.(82) | 8.5  (1) | Consensus-based recommendation | U | Treatment | ED Hosp |
|  |  | HEAD36 | Children who presented with moderate head injury (GCS 9-13) without neurological deterioration had their pulse rate observed in hospital at least half-hourly for a minimum of four hours. | 4 | NSW Kids and Families. 2011.(81)  The Royal Children's Hospital Melbourne.(82) | 7.5  (1.9) | Consensus-based recommendation | U | Treatment | ED Hosp |
|  |  | HEAD37 | Children who presented with moderate head injury (GCS 9-13) without neurological deterioration had their respiratory rate observed in hospital at least half-hourly for a minimum of four hours. | 4 | NSW Kids and Families. 2011.(81)  The Royal Children's Hospital Melbourne.(82) | 7.5  (1.9) | Consensus-based recommendation | U | Treatment | ED Hosp |
|  |  | HEAD38 | Children who presented with moderate head injury (GCS 9-13) without neurological deterioration had their blood pressure observed in hospital at least half-hourly for a minimum of four hours. | 4 | NSW Kids and Families. 2011.(81)  The Royal Children's Hospital Melbourne.(82) | 7.3  (1.7) | Consensus-based recommendation | U | Treatment | ED Hosp |
|  |  | HEAD39 | Children who presented with moderate head injury (GCS 9-13) without neurological deterioration had their pupils assessed in hospital at least half-hourly for a minimum of four hours. | 4 | NSW Kids and Families. 2011.(81)  The Royal Children's Hospital Melbourne.(82) | 7.5  (1.9) | Consensus-based recommendation | U | Treatment | ED Hosp |
|  |  | HEAD40 | Children who presented with moderate head injury (GCS 9-13) without neurological deterioration had their limb strength assessed in hospital at least half-hourly for a minimum of four hours. | 4 | NSW Kids and Families. 2011.(81)  The Royal Children's Hospital Melbourne.(82) | 7.5  (1.9) | Consensus-based recommendation | U | Treatment | ED Hosp |
| 11 | Children with a moderate / intermediate head injury (GCS 9-13) receive a CT head if they experience an acute deterioration or prolonged observation if they have persistent symptoms (at 6 hours post injury) including:  vomiting OR  headache OR  irritability OR  abnormal behaviour / neurological abnormality OR  unsteady gait. | HEAD41 | Children with a moderate / intermediate head injury (GCS 9-13) who experienced an acute deterioration including persistent vomiting (at 6 hours post injury) received a CT head. | 5 | NSW Kids and Families. 2011.(81)  The Royal Children's Hospital Melbourne.(82) | 8  (1) | Grade B | U | Treatment | ED Hosp |
|  |  | HEAD42 | Children with a moderate / intermediate head injury (GCS 9-13) who experienced an acute deterioration including persistent headache (at 6 hours post injury) received a CT head. | 5 | NSW Kids and Families. 2011.(81)  The Royal Children's Hospital Melbourne.(82) | 7.6  (1.7) | Grade B | U | Treatment | ED Hosp |
|  |  | HEAD43 | Children with a moderate / intermediate head injury (GCS 9-13) who experienced an acute deterioration including persistent irritability (at 6 hours post injury) received a CT head. | 5 | NSW Kids and Families. 2011.(81)  The Royal Children's Hospital Melbourne.(82) | 8  (1) | Grade B | U | Treatment | ED Hosp |
|  |  | HEAD44 | Children with a moderate / intermediate head injury (GCS 9-13) who experienced an acute deterioration including persistent abnormal behaviour / neurological abnormality (at 6 hours post injury) received a CT head. | 5 | NSW Kids and Families. 2011.(81)  The Royal Children's Hospital Melbourne.(82) | 8.4  (0.9) | Grade B | U | Treatment | ED Hosp |
|  |  | HEAD45 | Children with a moderate / intermediate head injury (GCS 9-13) who experienced an acute deterioration including persistent unsteady gait (at 6 hours post injury) received a CT head. | 5 | NSW Kids and Families. 2011.(81)  The Royal Children's Hospital Melbourne.(82) | 7.8  (1.3) | Grade B | U | Treatment | ED Hosp |
| 12 | Children presenting with a head injury:  are NOT intubated via a nasotracheal airway AND  do NOT receive a nasogastric tube. | HEAD46 | Children who presented with a head injury were not intubated via a nasotracheal airway. | 4 | NSW Kids and Families. 2011.(81) | 8.5  (1) | Consensus-based recommendation | O | Treatment | ED Hosp |
|  |  | HEAD47 | Children who presented with a head injury did not receive a nasogastric tube. | 4 | NSW Kids and Families. 2011.(81) | 8.3  (1.0) | Consensus-based recommendation | O | Treatment | ED Hosp |
| 13 | Children with a head injury who are intubated have the following:  end tidal CO2 monitoring, AND  PaO2 greater than 80mmHg (SaO2 greater than 95%), AND  PaCO2 between 35-40 mmHg. | HEAD48 | Children with a head injury who were intubated had end tidal CO2 monitoring. | 4 | NSW Kids and Families. 2011.(81) | 8.5  (0.6) | Consensus-based recommendation | U | Treatment | ED Hosp |
|  |  | HEAD49 | Children with a head injury who were intubated had PaO2 greater than 80mmHg (SaO2 greater than 95%). | 4 | NSW Kids and Families. 2011.(81) | 8.5  (0.6) | Consensus-based recommendation | U | Treatment | ED Hosp |
|  |  | HEAD50 | Children with a head injury who were intubated had PaCO2 between 35-40 mmHg. | 4 | NSW Kids and Families. 2011.(81) | 8.3  (1.0) | Consensus-based recommendation | U | Treatment | ED Hosp |
| 14 | Children presenting with a head injury and any of the following receive cervical spine precautions:  GCS is less than 15 OR  Posterior bony neck pain or tenderness OR  Focal neurological deficit at any time since injury OR  Paraesthesia in the extremities OR  Distracting injury OR  Intoxication. | HEAD51 | Children who presented with a head injury and any of the following: - GCS less than 15 OR - posterior bony neck pain or tenderness OR - focal deficit at any time since injury OR - paraethesia in the extremities OR - distracting injury OR - intoxication received cervical spine precautions. | 4 | NSW Kids and Families. 2011.(81) | 7.8  (1.9) | Consensus-based recommendation | U | Treatment | ED Hosp |
| 15 | Children presenting with head injury who are seizing are immediately administered the following:  midazolam (0.15mg/kg bolus IV), OR  diazepam (0.25mg/kg bolus IV) OR  midazolam 0.15mg/kg IM, 0.5 mg/kg IN or 0.5 mg/kg buccal. | HEAD52 | Children who presented with head injury who were seizing, were immediately administered: - midazolam (0.15mg/kg bolus IV), OR - diazepam (0.25mg/kg bolus IV) OR - midazolam 0.15mg/kg IM, 0.5 mg/kg IN or 0.5 mg/kg buccal. | 4 | NSW Kids and Families. 2011.(81) | 8.8  (0.5) | Consensus-based recommendation | U | Treatment | ED Hosp |
| 16 | Children presenting with head injury who receive sedation and/or opioid analgesia have GCS recorded every 15 minutes until GCS returns to pre-sedation level. | HEAD53 | Children who presented with head injury and received sedation and/or opioid analgesia had their GCS recorded every 15 minutes until their GCS returned to the pre-sedation level. | 4 | NSW Kids and Families. 2011.(81) | 7.4  (1.7) | Consensus-based recommendation | U | Treatment | ED Hosp |
| 17 | Children with a minor / mild head injury (GCS 14-15) where information is provided to parents on when to return to the ED if deterioration occurs, are discharged from the ED without a period of observation. | HEAD54 | Children with a minor / mild head injury (GCS 14-15) whose parents were provided with information on when to return to the ED if deterioration occurs, were discharged from the ED without a period of observation. | 5 | NSW Kids and Families. 2011.(81)  The Royal Children's Hospital Melbourne.(82) | 7  (3.4) | Consensus-based recommendation | U | Ongoing management | ED |
| **OBESITY** | | | | | | | | | | |
| 1 | Children aged between 2-16 years are diagnosed as follows:  overweight (if BMI for age and sex in 85th - 94th percentile) OR  obese (if BMI for age and sex greater than 95th percentile). | OBES01 | Children aged between 2-16 years with a BMI for age and sex in 85th - 94th percentile were diagnosed as overweight. | 3 | National Health and Medical Research Council. 2013.(83)  Scottish Intercollegiate Guidelines Network. 2010.(84)  Lau DC, Douketis JD, Morrison KM, et al. 2006.(85)  Ministry of Health - Clinical Trials Research Unit. 2009.(86)  Calonge N, Petitti DB, DeWitt TG, et al.(87)  Barlow SE. 2007.(88)  August GP, Caprio S, Fennoy I, et al.(89) | 8.5  (1) | Grade C | U | Diagnosis | GP  Spec |
|  |  | OBES02 | Children aged between 2-16 years with a BMI for age and sex greater than 95th percentile were diagnosed as obese. | 3 | National Health and Medical Research Council. 2013.(83)  Scottish Intercollegiate Guidelines Network. 2010.(84)  Lau DC, Douketis JD, Morrison KM, et al. 2006.(85)  Ministry of Health - Clinical Trials Research Unit. 2009.(86)  Calonge N, Petitti DB, DeWitt TG, et al. (87)  Barlow SE. 2007.(88)  August GP, Caprio S, Fennoy I, et al.(89) | 8.5  (1) | Grade C | U | Diagnosis | GP Spec |
| 2 | Children who are overweight or obese have the following recorded at the time of diagnosis:  current physical (e.g. snoring and joint problems) and social (e.g. isolation, bullying, and depression) consequences of overweight AND  family history of obesity, early cardiovascular disease, diabetes or dyslipidaemia AND  medication history. | OBES03 | Children who were overweight or obese had their current physical (e.g. snoring and joint problems) and social (e.g. isolation, bullying, and depression) consequences of overweight recorded at the time of diagnosis. | 3 | Lau DC, Douketis JD, Morrison KM, et al. 2006.(85)  Ministry of Health - Clinical Trials Research Unit. 2009.(86)  Barlow SE. 2007.(88)  Royal Childrens Hospital. 2009.(49)  National Institute for Health and Clinical Excellence. 2006.(90) | 7  (1.7) | Consensus-based recommendation | U | Diagnosis | GP Spec |
|  |  | OBES04 | Children who were overweight or obese had their family history of obesity, early cardiovascular disease, diabetes or dyslipidaemia recorded at the time of diagnosis. | 3 | Lau DC, Douketis JD, Morrison KM, et al. 2006.(85)  Ministry of Health - Clinical Trials Research Unit. 2009.(86)  Barlow SE. 2007.(88)  Royal Childrens Hospital. 2009.(49)  National Institute for Health and Clinical Excellence. 2006.(90) | 7  (1.7) | Consensus-based recommendation | U | Diagnosis | GP Spec |
|  |  | OBES05 | Children who were overweight or obese had their medication history recorded at the time of diagnosis. | 3 | Lau DC, Douketis JD, Morrison KM, et al. 2006.(85)  Ministry of Health - Clinical Trials Research Unit. 2009.(86)  Barlow SE. 2007.(88)  Royal Childrens Hospital. 2009.(49)  National Institute for Health and Clinical Excellence. 2006.(90) | 8.3  (1.2) | Consensus-based recommendation | U | Diagnosis | GP  Spec |
| 3 | Children who are overweight or obese have a clinical examination at the time of diagnosis which includes:  height and weight to calculate and confirm BMI AND"  abnormal gait, flat feet, or problems with hips and knees AND  presence of striae, intertrigo, or hepatomegaly AND  assessment for short stature, a low height velocity, or bruising or purple striae AND  pubertal stage (Tanner stage). | OBES06 | Children who were overweight or obese had a clinical examination of their height and weight to calculate and confirm BMI at the time of diagnosis. | 3 | Ministry of Health - Clinical Trials Research Unit. 2009.(86)  Royal Childrens Hospital. 2009.(49) | 9  (0) | Consensus-based recommendation | U | Diagnosis | GP Spec |
|  |  | OBES07 | Children who were overweight or obese had a clinical examination of their gait, feet, hips and knees at the time of diagnosis. | 3 | Ministry of Health - Clinical Trials Research Unit. 2009.(86)  Royal Childrens Hospital. 2009.(49) | 7.3  (2.1) | Consensus-based recommendation | U | Diagnosis | GP Spec |
|  |  | OBES08 | Children who were overweight or obese had a clinical examination for the presence of striae, intertrigo, or hepatomegaly at the time of diagnosis. | 3 | Ministry of Health - Clinical Trials Research Unit. 2009.(86)  Royal Childrens Hospital. 2009.(49) | 7  (2) | Consensus-based recommendation | U | Diagnosis | GP Spec |
|  |  | OBES09 | Children who were overweight or obese had a clinical examination for short stature, a low height velocity, or bruising or purple striae at the time of diagnosis. | 3 | Ministry of Health - Clinical Trials Research Unit. 2009.(86)  Royal Childrens Hospital. 2009.(49) | 7.3  (2.1) | Consensus-based recommendation | U | Diagnosis | GP Spec |
|  |  | OBES10 | Children who were overweight or obese had a clinical examination of their pubertal stage (Tanner stage) at the time of diagnosis. | 3 | Ministry of Health - Clinical Trials Research Unit. 2009.(86)  Royal Childrens Hospital. 2009.(49) | 7.3  (2.1) | Consensus-based recommendation | U | Diagnosis | GP  Spec |
| 4 | Overweight children have written evidence that they were advised of making the following lifestyle changes:  reducing energy intake AND  referred to a dietician AND  reducing level of sedentary behaviour AND  increasing physical activity (at least 60 mins/day) AND  advised on family based behavioural strategies. | OBES11 | Overweight children were advised to reduce their energy intake. | 3 | National Health and Medical Research Council. 2013.(83) | 8.3  (1.2) | Grade B | U | Treatment | GP Spec |
|  |  | OBES12 | Overweight children were referred to a dietician. | 3 | National Health and Medical Research Council. 2013.(83) | 7.3  (2.1) | Grade B | U | Treatment | GP Spec |
|  |  | OBES13 | Overweight children were advised to reduce their level of sedentary behaviour. | 3 | National Health and Medical Research Council. 2013.(83) | 8.3  (1.2) | Grade B | U | Treatment | GP Spec |
|  |  | OBES14 | Overweight children were advised to increase their physical activity (to at least 60 mins/day). | 3 | National Health and Medical Research Council. 2013.(83) | 8  (1) | Grade B | U | Treatment | GP Spec |
|  |  | OBES15 | Overweight children were advised on family based behavioural strategies. | 3 | National Health and Medical Research Council. 2013.(83) | 8.7  (0.6) | Grade B | U | Treatment | GP Spec |
| 5 | Overweight children are referred to a paediatrician or weight management clinic if they have any of the following:  aged between 2 and 16 years and have a BMI the 95th percentile on US-CDC growth charts or the 97th percentile on WHO charts OR  aged less than 2 years, above the 97th percentile on WHO growth charts OR  serious related comorbidities that require weight management (e.g. sleep apnoea, orthopaedic problems, liver dysfunction, endocrine disorder, risk factors for cardiovascular disease or type 2 diabetes, psychological distress). | OBES16 | Overweight children aged between 2 and 16 years, and a BMI in the 95th percentile on US-CDC growth charts OR the 97th percentile on WHO charts, were referred to a paediatrician or a weight management clinic. | 3 | National Health and Medical Research Council. 2013.(83)  Scottish Intercollegiate Guidelines Network. 2010.(84)  Lau DC, Douketis JD, Morrison KM, et al. 2006.(85)  August GP, Caprio S, Fennoy I, et al. 2008.(89)  National Institute for Health and Clinical Excellence. 2006.(90) | 7  (2) | Consensus-based recommendation | U | Treatment | GP Spec |
|  |  | OBES17 | Overweight children aged less than 2 years, above the 97th percentile on WHO growth charts, were referred to a paediatrician or a weight management clinic. | 3 | National Health and Medical Research Council. 2013.(83)  Scottish Intercollegiate Guidelines Network. 2010.(84)  Lau DC, Douketis JD, Morrison KM, et al. 2006.(85)  August GP, Caprio S, Fennoy I, et al. 2008.(89)  National Institute for Health and Clinical Excellence. 2006.(90) | 7.7  (2.3) | Consensus-based recommendation | U | Treatment | GP Spec |
|  |  | OBES18 | Overweight children with serious related comorbidities that require weight management (e.g. sleep apnoea, orthopaedic problems, liver dysfunction, endocrine disorder, risk factors for cardiovascular disease or type 2 diabetes, psychological distress), were referred to a paediatrician or a weight management clinic. | 3 | National Health and Medical Research Council. 2013.(83)  Scottish Intercollegiate Guidelines Network. 2010.(84)  Lau DC, Douketis JD, Morrison KM, et al. 2006.(85)  August GP, Caprio S, Fennoy I, et al. 2008.(89)  National Institute for Health and Clinical Excellence. 2006.(90) | 8.7  (0.6) | Consensus-based recommendation | U | Ongoing management | GP Spec |
| **OTITIS MEDIA** | | | | | | | | | | |
| 1 | Children aged 12 months to 2 years diagnosed with AOM are managed as follows:  advise parents to observe the child for up to 48hrs from the onset of symptoms AND  provide systemic analgesics (paracetamol (PO) OR Ibuprofen) AND  arrange follow up visit at 48hrs AND  prescribe antibiotics after 24-48 hours if symptoms are unchanged or worsen | OTIT01 | The parents of children aged 12 months to 2 years diagnosed with AOM were advised to observe the child for up to 48hrs from the onset of symptoms. | 3 | Gunasekera H. 2008.(91)  NSW Kids and Families. 2005.(92)  British Columbia Medical Association. 2010.(93) | 9  (0) | Consensus-based recommendation | U | Diagnosis | ED  GP  Hosp  Spec |
|  |  | OTIT02 | Children aged 12 months to 2 years diagnosed with AOM were provided systemic analgesics (paracetamol (PO) OR Ibuprofen). | 3 | Gunasekera H. 2008.(91)  NSW Kids and Families. 2005.(92)  British Columbia Medical Association. 2010.(93) | 9  (0) | Consensus-based recommendation | U | Diagnosis | ED  GP  Hosp  Spec |
|  |  | OTIT03 | Children aged 12 months to 2 years diagnosed with AOM had a follow-up visit arranged at 48 hours. | 3 | Gunasekera H. 2008.(91)  NSW Kids and Families. 2005.(92)  British Columbia Medical Association. 2010.(93) | 9  (0) | Consensus-based recommendation | U | Treatment | ED  GP  Hosp  Spec |
|  |  | OTIT04 | Children aged 12 months to 2 years diagnosed with AOM, whose symptoms were unchanged or worsened after 24-48 hours, were prescribed antibiotics. | 3 | Gunasekera H. 2008.(91)  NSW Kids and Families. 2005.(92)  British Columbia Medical Association. 2010.(93) | 9  (0) | Grade B | U | Treatment | ED  GP  Hosp  Spec |
| 2 | Children with AOM aged greater than 12 months who are mildly unwell are NOT prescribed antibiotics. | OTIT05 | Children with AOM aged greater than 12 months who were mildly unwell were not prescribed antibiotics. | 3 | Royal Childrens Hospital. 2009.(49)  Lieberthal AS, Carroll AE, Chonmaitree T, et al. 2013.(94)  Scottish Intercollegiate Guidelines Network. 2003.(95) | 9  (0) | Grade B | O | Treatment | ED  GP  Hosp  Spec |
| 3 | Children with AOM and any of the following are prescribed an antibiotic:  - aged less than 6 months OR  - aged greater than 6 months, severe symptoms or severely unwell, diagnosis is certain or bilateral AOM OR  - child is Aboriginal or Torres Strait Islander OR  - severe symptoms (moderate or severe otalgia or otalgia for at least 48 hours or temperature 39 degrees Celsius or higher) OR  - child distressed beyond 24-48 hours OR  - Inflammation does not resolve within 48 hours | OTIT06 | Children aged less than 6 months with AOM were prescribed an antibiotic. | 3 | Queensland Government Royal Children's Hospital Brisbane. 2010.(96)  Gunasekera H. 2008.(91) | 9  (0) | Consensus-based recommendation | U | Treatment | ED  GP  Hosp  Spec |
|  |  | OTIT07 | Children aged > 6 months with AOM, who had severe symptoms or were severely unwell, or their diagnosis was certain or they had bilateral AOM, were prescribed an antibiotic. | 3 | Queensland Government Royal Children's Hospital Brisbane. 2010.(96)  Gunasekera H. 2008.(91) | 8.7  (0.6) | Consensus-based recommendation | U | Treatment | ED  GP  Hosp  Spec |
|  |  | OTIT08 | Children who were Aboriginal or a Torres Strait Islander with AOM were prescribed an antibiotic. | 3 | Queensland Government Royal Children's Hospital Brisbane. 2010.(96)  Gunasekera H. 2008.(91) | 9  (0) | Consensus-based recommendation | U | Treatment | ED  GP  Hosp  Spec |
|  |  | OTIT09 | Children with AOM and severe symptoms (moderate or severe otalgia or otalgia for at least 48 hours or temperature 39 degrees Celsius or higher), were prescribed an antibiotic. | 3 | Queensland Government Royal Children's Hospital Brisbane. 2010.(96)  Gunasekera H. 2008.(91) | 9  (0) | Consensus-based recommendation | U | Treatment | ED  GP  Hosp  Spec |
|  |  | OTIT10 | Children with AOM who were distressed for more than 24-48 hours, were prescribed an antibiotic. | 3 | Queensland Government Royal Children's Hospital Brisbane. 2010.(96)  Gunasekera H. 2008.(91) | 9  (0) | Consensus-based recommendation | U | Treatment | ED  GP  Hosp  Spec |
|  |  | OTIT11 | Children with AOM and for whom their inflammation did not resolve within 48 hours, were prescribed an antibiotic. | 3 | Queensland Government Royal Children's Hospital Brisbane. 2010.(96)  Gunasekera H. 2008.(91) | 8.7  (0.6) | Consensus-based recommendation | U | Treatment | ED  GP  Hosp  Spec |
| 4 | Children with AOM are prescribed the following antibiotics:  Amoxicillin 45mg/kg/day for 5 days OR  Roxithromycin 2.5-mg/kg (max 150mg) oral 12hrly for 5 days or Cefaclor 10mg/kg up to 250 mg PO, 8 hourly for 5 days if allergic to penicillin OR  Aboriginal or Torres Strait Islander - amoxicillin 50mg/kg for 7 days OR  Aboriginal or Torres Strait Islander with perforation - amoxicillin 50-90mg/kg for 14 days. | OTIT12 | Children with AOM were prescribed the following antibiotics: - Amoxicillin 45mg/kg/day for 5 days OR - Roxithromycin 2.5-mg/kg (max 150mg) oral 12hrly for 5 days or Cefaclor 10mg/kg up to 250 mg PO, 8 hourly for 5 days if allergic to penicillin. | 3 | Gunasekera H. 2008.(91)  Department of Health Western Australia. 2013.(97) | 8.7  (0.6) | Grade B | U | Treatment | ED  GP  Hosp  Spec |
|  |  | OTIT13 | Children with AOM who were Aboriginal or Torres Strait Islander were prescribed amoxicillin 50mg/kg for 7 days. | 3 | Gunasekera H. 2008.(91)  Department of Health Western Australia. 2013.(97) | 8.7  (0.6) | Consensus-based recommendation | U | Treatment | ED  GP  Hosp  Spec |
|  |  | OTIT14 | Children with perforated AOM who were Aboriginal or Torres Strait Islander were prescribed amoxicillin 50-90mg/kg for 14 days. | 3 | Gunasekera H. 2008.(91)  Department of Health Western Australia. 2013.(97) | 8.7  (0.6) | Consensus-based recommendation | U | Treatment | ED  GP  Hosp  Spec |
| 5 | Children with AOM who require antibiotics and meet any of the following criteria are also prescribed Beta-lactamase coverage: received amoxicillin in the last 30 days OR  concurrent purulent conjunctivitis OR  history of recurrent AOM unresponsive to amoxicillin | OTIT15 | Children with AOM who required antibiotics and had a history of recurrent AOM unresponsive to amoxicillin, OR concurrent purulent conjunctivitis, OR received amoxicillin in the last 30 days were also prescribed Beta-lactamase coverage. | 3 | Lieberthal AS, Carroll AE, Chonmaitree T, et al. 2013.(94) | 8.7  (0.6) | Grade C | U | Treatment | ED  GP  Hosp  Spec |
| 6 | Children with OME without hearing loss are NOT prescribed or advised to use:  antibiotics OR  decongestants OR  antihistamines OR  mucolytics OR  steroids (topical or systemic) | OTIT16 | Children with OME without hearing loss were not prescribed or advised to use antibiotics, OR decongestants, OR antihistamines, OR mucolytics OR steroids (topical or systemic). | 3 | British Columbia Medical Association. 2010.(93)  Scottish Intercollegiate Guidelines Network. 2003.(95) | 9  (0) | Grade D  (antibiotics)  Grade B  (decongestants, antihistamines, mucolytics)  Grade B  (steroids) | O | Treatment | ED  GP  Hosp  Spec |
| 7 | Children with AOM and any of the following are referred to an ENT specialist:  persisting AOM with speech or general developmental delay OR"  underlying ENT abnormalities OR  an effusion lasting longer than 3 months with bilateral hearing impairment (greater than 20dB) OR  recurrent symptomatic AOM episodes (greater than 4 times in 6mths) OR  cholesteatoma, mastoiditis or facial nerve palsies OR  chronic perforation not responding to treatment over 3 months OR  immunosuppressed | OTIT17 | Children with AOM and persistent AOM with speech or general developmental delay, were referred to an ENT specialist. | 3 | Royal Childrens Hospital. 2009.(49)  Gunasekera H. 2008.(91)  Scottish Intercollegiate Guidelines Network. 2003.(95) | 9  (0) | Consensus-based recommendation | U | Ongoing management | ED  GP  Hosp  Spec |
|  |  | OTIT18 | Children with AOM and underlying ENT abnormalities, were referred to an ENT specialist. | 3 | Royal Childrens Hospital. 2009.(49)  Gunasekera H. 2008.(91)  Scottish Intercollegiate Guidelines Network. 2003.(95) | 9  (0) | Consensus-based recommendation | U | Ongoing management | ED  GP  Hosp  Spec |
|  |  | OTIT19 | Children with AOM and an effusion lasting longer than 3 months with bilateral hearing impairment (greater than 20dB), were referred to an ENT specialist. | 3 | Royal Childrens Hospital. 2009.(49)  Gunasekera H. 2008.(91)  Scottish Intercollegiate Guidelines Network. 2003.(95) | 8.7  (0.6) | Consensus-based recommendation | U | Ongoing management | ED  GP  Hosp  Spec |
|  |  | OTIT20 | Children with recurrent symptomatic AOM episodes (greater than 4 times in 6mths), were referred to an ENT specialist. | 3 | Royal Childrens Hospital. 2009.(49)  Gunasekera H. 2008.(91)  Scottish Intercollegiate Guidelines Network. 2003.(95) | 8.3  (1.2) | Grade D | U | Ongoing management | ED  GP  Hosp  Spec |
|  |  | OTIT21 | Children with AOM and cholesteatoma, mastoiditis or facial nerve palsies, were referred to an ENT specialist. | 3 | Royal Childrens Hospital. 2009.(49)  Gunasekera H. 2008.(91)  Scottish Intercollegiate Guidelines Network. 2003.(95) | 9  (0) | Consensus-based recommendation | U | Ongoing management | ED  GP  Hosp  Spec |
|  |  | OTIT22 | Children with AOM and chronic perforation not responding to treatment over 3 months, were referred to an ENT specialist. | 3 | Royal Childrens Hospital. 2009.(49)  Gunasekera H. 2008.(91)  Scottish Intercollegiate Guidelines Network. 2003.(95) | 9  (0) | Consensus-based recommendation | U | Ongoing management | ED  GP  Hosp  Spec |
|  |  | OTIT23 | Children with AOM and who were immunosuppressed, were referred to an ENT specialist. | 3 | Royal Childrens Hospital. 2009.(49)  Gunasekera H. 2008.(91)  Scottish Intercollegiate Guidelines Network. 2003.(95) | 9  (0) | Consensus-based recommendation | U | Ongoing management | ED  GP  Hosp  Spec |
| 8 | Children diagnosed with OME are managed as follows:  reviewed in 3 months AND  if symptoms continue refer for an audiogram | OTIT24 | Children diagnosed with OME were reviewed in 3 months. | 3 | Gunasekera H. 2008.(91) | 9  (0) | Consensus-based recommendation | U | Ongoing management | ED  GP  Hosp  Spec |
|  |  | OTIT25 | Children diagnosed with OME and continued symptoms were referred for an audiogram. | 3 | Gunasekera H. 2008.(91) | 9  (0) | Consensus-based recommendation | U | Ongoing management | ED  GP  Hosp  Spec |
| 9 | Children aged less than 3 years with OME who have the following are reviewed every 3 months but NOT referred for surgery:  persistent bilateral OME AND  hearing loss of less than or equal to 25 dB AND  no speech and language, development or behavioural problems | OTIT26 | Children aged less than 3 years with persistent bilateral OME were reviewed every 3 months. | 3 | Gunasekera H. 2008.(91)  Scottish Intercollegiate Guidelines Network. 2003.(95) | 8.7  (0.6) | Grade A | U | Ongoing management | ED  GP  Hosp  Spec |
|  |  | OTIT27 | Children aged less than 3 years with persistent bilateral OME were not referred for surgery. | 3 | Gunasekera H. 2008.(91)  Scottish Intercollegiate Guidelines Network. 2003.(95) | 8.7  (0.6) | Grade A | O | Ongoing management | ED  GP  Hosp  Spec |
|  |  | OTIT28 | Children aged less than 3 years with OME and hearing loss of less than or equal to 25 dB were reviewed every 3 months. | 3 | Gunasekera H. 2008.(91)  Scottish Intercollegiate Guidelines Network. 2003.(95) | 8.7  (0.6) | Grade A | U | Ongoing management | ED  GP  Hosp  Spec |
|  |  | OTIT29 | Children aged less than 3 years with OME and hearing loss of less than equals to 25 dB were not referred for surgery. | 3 | Gunasekera H. 2008.(91)  Scottish Intercollegiate Guidelines Network. 2003.(95) | 8.7  (0.6) | Grade A | O | Ongoing management | ED  GP  Hosp  Spec |
|  |  | OTIT30 | Children aged less than 3 years with OME and no speech, language development or behavioural problems, were reviewed every 3 months. | 3 | Gunasekera H. 2008.(91)  Scottish Intercollegiate Guidelines Network. 2003. (95) | 8.7  (0.6) | Grade A | U | Ongoing management | ED  GP  Hosp  Spec |
|  |  | OTIT31 | Children aged less than 3 years with OME and no speech, language development or behavioural problems, were not referred for surgery. | 3 | Gunasekera H. 2008.(91)  Scottish Intercollegiate Guidelines Network. 2003.(95) | 8.7  (0.6) | Grade A | O | Ongoing management | ED  GP  Hosp  Spec |
| 10 | Children with OME with any of the following are referred to an ENT specialist:  aged greater than 3years with persistent bilateral OME OR  speech and language, developmental or behavioural problems | OTIT32 | Children aged greater than 3 years with persistent bilateral OME were referred to an ENT specialist. | 3 | Scottish Intercollegiate Guidelines Network. 2003.(95) | 8.7  (0.6) | Grade B | U | Ongoing management | ED  GP  Hosp  Spec |
|  |  | OTIT33 | Children aged greater than 3 years with OME and speech and language, developmental or behavioural problems were referred to an ENT specialist. | 3 | Scottish Intercollegiate Guidelines Network. 2003.(95) | 9  (0) | Grade B | U | Ongoing management | ED  GP  Hosp  Spec |
| 11 | Children with OME and any of the following are referred to an ENT specialist  OME greater than 3 months with evidence of hearing loss OR  greater than equals to 3 episodes of AOM in six month period OR  greater than equals to 4 episodes of AOM in 12 month period OR  retracted tympanic membrane | OTIT34 | Children with OME for more than 3 months and evidence of hearing loss, were referred to an ENT specialist. | 3 | NSW Kids and Families. 2005.(92) | 9  (0) | Consensus-based recommendation | U | Ongoing management | ED  GP  Hosp  Spec |
|  |  | OTIT35 | Children with OME and at least 3 episodes of AOM in a six month period, were referred to an ENT specialist. | 3 | NSW Kids and Families. 2005.(92) | 8.7  (0.6) | Consensus-based recommendation | U | Ongoing management | ED  GP  Hosp  Spec |
|  |  | OTIT36 | Children with OME and at least 4 episodes of AOM in a twelve month period, were referred to an ENT specialist. | 3 | NSW Kids and Families. 2005.(92) | 8.3  (1.2) | Consensus-based recommendation | U | Ongoing management | ED  GP  Hosp  Spec |
|  |  | OTIT37 | Children with OME and a retracted tympanic membrane, were referred to an ENT specialist. | 3 | NSW Kids and Families. 2005.(92) | 7.7  (2.3) | Consensus-based recommendation | U | Ongoing management | ED  GP  Hosp  Spec |
| **PREVENTIVE CARE** | | | | | | | | | | |
| 1 | Infants aged 2, 4 and 6 months have the following documented:  Weight AND  Length AND  Head circumference AND  Eyes: observation, fixation and following AND  Cardiovascular status AND  Hips, limbs, joints AND  Developmental progress AND  parental concerns. | PREV01 | Infants aged 2 months were weighed | 3 | Royal Australian College of General Practitioners. 2013.(98) | 9  (0) | Consensus-based recommendation | U | Screening | GP |
|  |  | PREV02 | Infants aged 4 months were weighed | 3 | Royal Australian College of General Practitioners. 2013.(98) | 9  (0) | Consensus-based recommendation | U | Screening | GP |
|  |  | PREV03 | Infants aged 6 months were weighed | 3 | Royal Australian College of General Practitioners. 2013.(98) | 9  (0) | Consensus-based recommendation | U | Screening | GP |
|  |  | PREV04 | Infants aged 2 months had their length measured | 3 | Royal Australian College of General Practitioners. 2013.(98) | 9  (0) | Consensus-based recommendation | U | Screening | GP |
|  |  | PREV05 | Infants aged 4 months had their length measured | 3 | Royal Australian College of General Practitioners. 2013.(98) | 9  (0) | Consensus-based recommendation | U | Screening | GP |
|  |  | PREV06 | Infants aged 6 months had their length measured | 3 | Royal Australian College of General Practitioners. 2013.(98) | 9  (0) | Consensus-based recommendation | U | Screening | GP |
|  |  | PREV07 | Infants aged 2 months had their head circumference measured | 3 | Royal Australian College of General Practitioners. 2013.(98) | 9  (0) | Consensus-based recommendation | U | Screening | GP |
|  |  | PREV08 | Infants aged 4 months had their head circumference measured | 3 | Royal Australian College of General Practitioners. 2013.(98) | 9  (0) | Consensus-based recommendation | U | Screening | GP |
|  |  | PREV09 | Infants aged 6 months had their head circumference measured | 3 | Royal Australian College of General Practitioners. 2013.(98) | 9  (0) | Consensus-based recommendation | U | Screening | GP |
|  |  | PREV10 | Infants aged 2 months had their eyes examined. | 3 | Royal Australian College of General Practitioners. 2013.(98) | 9  (0) | Consensus-based recommendation | U | Screening | GP |
|  |  | PREV11 | Infants aged 4 months had their eyes examined. | 3 | Royal Australian College of General Practitioners. 2013.(98) | 9  (0) | Consensus-based recommendation | U | Screening | GP |
|  |  | PREV12 | Infants aged 6 months had their eyes examined. | 3 | Royal Australian College of General Practitioners. 2013.(98) | 9  (0) | Consensus-based recommendation | U | Screening | GP |
|  |  | PREV13 | Infants aged 2 months had their cardiovascular status examined. | 3 | Royal Australian College of General Practitioners. 2013.(98) | 9  (0) | Consensus-based recommendation | U | Screening | GP |
|  |  | PREV14 | Infants aged 4 months had their cardiovascular status examined. | 3 | Royal Australian College of General Practitioners. 2013.(98) | 9  (0) | Consensus-based recommendation | U | Screening | GP |
|  |  | PREV15 | Infants aged 6 months had their cardiovascular status examined. | 3 | Royal Australian College of General Practitioners. 2013.(98) | 9  (0) | Consensus-based recommendation | U | Screening | GP |
|  |  | PREV16 | Infants aged 2 months had their hips, limbs and joints examined. | 3 | Royal Australian College of General Practitioners. 2013.(98) | 7.7  () | Consensus-based recommendation | U | Screening | GP |
|  |  | PREV17 | Infants aged 4 months had their hips, limbs and joints examined. | 3 | Royal Australian College of General Practitioners. 2013.(98) | 7.7 | Consensus-based recommendation | U | Screening | GP |
|  |  | PREV18 | Infants aged 6 months had their hips, limbs and joints examined. | 3 | Royal Australian College of General Practitioners. 2013.(98) | 7.7  (1.5) | Consensus-based recommendation | U | Screening | GP |
|  |  | PREV19 | Infants aged 2 months had their developmental progress examined. | 3 | Royal Australian College of General Practitioners. 2013.(98) | 9  (0) | Consensus-based recommendation | U | Screening | GP |
|  |  | PREV20 | Infants aged 4 months had their developmental progress examined. | 3 | Royal Australian College of General Practitioners. 2013.(98) | 9  (0) | Consensus-based recommendation | U | Screening | GP |
|  |  | PREV21 | Infants aged 6 months had their developmental progress examined. | 3 | Royal Australian College of General Practitioners. 2013.(98) | 9  (0) | Consensus-based recommendation | U | Screening | GP |
|  |  | PREV22 | Infants aged 2 months had any parental concerns documented. | 3 | Royal Australian College of General Practitioners. 2013.(98) | 8.7  (0.6) | Consensus-based recommendation | U | Screening | GP |
|  |  | PREV23 | Infants aged 4 months had any parental concerns documented. | 3 | Royal Australian College of General Practitioners. 2013.(98) | 8.7  (0.6) | Consensus-based recommendation | U | Screening | GP |
|  |  | PREV24 | Infants aged 6 months had any parental concerns documented. | 3 | Royal Australian College of General Practitioners. 2013.(98) | 8.7  (0.6) | Consensus-based recommendation | U | Screening | GP |
| 2 | Infants aged 2, 4 and 6 months receive a nutrition assessment (breastfeeding, solids). | PREV25 | Infants aged 2 months had their nutrition assessed. | 3 | Royal Australian College of General Practitioners. 2013.(98) | 8.3  (1.2) | Grade B | U | Screening | GP |
|  |  | PREV26 | Infants aged 4 months had their nutrition assessed. | 3 | Royal Australian College of General Practitioners. 2013.(98) | 8.3  (1.2) | Grade B | U | Screening | GP |
|  |  | PREV27 | Infants aged 6 months had their nutrition assessed. | 3 | Royal Australian College of General Practitioners. 2013.(98) | 8.3  (1.2) | Grade B | U | Screening | GP |
| 3 | Infants aged 12 and 18 months have the following documented:  Weight velocity AND  Height velocity.  Eyes and vision: observation, fixation and following, corneal light reflex AND  Developmental progress. | PREV28 | Infants aged 12 months were weighed. | 3 | Royal Australian College of General Practitioners. 2013.(98) | 9  (0) | Consensus-based recommendation | U | Screening | GP |
|  |  | PREV29 | Infants aged 18 months were weighed. | 3 | Royal Australian College of General Practitioners. 2013.(98) | 9  (0) | Consensus-based recommendation | U | Screening | GP |
|  |  | PREV30 | Infants aged 12 months had their height measured. | 3 | Royal Australian College of General Practitioners. 2013.(98) | 7.7  (2.3) | Consensus-based recommendation | U | Screening | GP |
|  |  | PREV31 | Infants aged 18 months had their height measured. | 3 | Royal Australian College of General Practitioners. 2013.(98) | 7.7  (2.3) | Consensus-based recommendation | U | Screening | GP |
|  |  | PREV32 | Infants aged 12 months had their eyes and vision examined. | 3 | Royal Australian College of General Practitioners. 2013.(98) | 8  (1.7) | Consensus-based recommendation | U | Screening | GP |
|  |  | PREV33 | Infants aged 18 months had their eyes and vision examined. | 3 | Royal Australian College of General Practitioners. 2013.(98) | 8  (1.7) | Consensus-based recommendation | U | Screening | GP |
|  |  | PREV34 | Infants aged 12 months had their developmental progress examined. | 3 | Royal Australian College of General Practitioners. 2013.(98) | 7.7  (2.3) | Consensus-based recommendation | U | Screening | GP |
|  |  | PREV35 | Infants aged 18 months had their developmental progress examined. | 3 | Royal Australian College of General Practitioners. 2013.(98) | 7.7  (2.3) | Consensus-based recommendation | U | Screening | GP |
| 4 | Children aged 2 years have the following documented:  Weight velocity AND"  Height AND  Assess development and behaviour. | PREV36 | Children aged 2 years were weighed. | 3 | Royal Australian College of General Practitioners. 2013.(98) | 7.7  (2.3) | Grade C | U | Screening | GP |
|  |  | PREV37 | Children aged 2 years had their height measured | 3 | Royal Australian College of General Practitioners. 2013.(98) | 7.7  (2.3) | Grade C | U | Screening | GP |
|  |  | PREV38 | Children aged 2 years had their development and behaviour assessed. | 3 | Royal Australian College of General Practitioners. 2013.(98) | 9  (0) | Grade C | U | Screening | GP |
| 5 | Infants aged 2, 4 and 6 months received immunisation according to DOHA immunisation schedule. | PREV39 | Infants aged 2 months received immunisation according to DOHA immunisation schedule | 3 | Royal Australian College of General Practitioners. 2013.(98) | 9  (0) | Consensus-based recommendation | U | Treatment | GP |
|  |  | PREV40 | Infants aged 4 months received immunisation according to DOHA immunisation schedule | 3 | Royal Australian College of General Practitioners. 2013.(98) | 9  (0) | Consensus-based recommendation | U | Treatment | GP |
|  |  | PREV41 | Infants aged 6 months received immunisation according to DOHA immunisation schedule | 3 | Royal Australian College of General Practitioners. 2013.(98) | 9  (0) | Consensus-based recommendation | U | Treatment | GP |
| 6 | Children aged 2 years received immunisation according to DOHA immunisation schedule. | PREV42 | Children aged 2 years received immunisation according to DOHA immunisation schedule | 3 | Royal Australian College of General Practitioners. 2013.(98) | 9  (0) | Consensus-based recommendation | U | Treatment | GP |
| 7 | Children aged 4 years are immunised according to DOHA immunisation schedule. | PREV43 | Children aged 4 years are immunised according to DOHA immunisation schedule | 3 | Royal Australian College of General Practitioners. 2013.(98) | 9  (0) | Consensus-based recommendation | U | Treatment | GP |
| **SEIZURES** | | | | | | | | | | |
| 1 | Children presenting with seizures have a history recorded that includes documentation of:  Current febrile illness AND  Neurologic state prior to the seizure AND  Recent trauma AND  History of epilepsy AND  Current medication and allergies AND  Potential for ingestion of medications or poisons AND  Past medical history including anti-convulsant toxicity AND  Immunisation history. | SEIZ01 | Children who presented with seizures had a history recorded including assessment for the presence of current febrile illness. | 4 | NSW Kids and Families. 2009.(99) | 8.8  (0.5) | Grade D | U | Diagnosis | ED Hosp |
|  |  | SEIZ02 | Children who presented with seizures had a history recorded including assessment of their neurologic state prior to the seizure. | 4 | NSW Kids and Families. 2009.(99) | 8.3  (1.0) | Grade D | U | Diagnosis | ED Hosp |
|  |  | SEIZ03 | Children who presented with seizures had a history recorded including an assessment for any recent trauma. | 4 | NSW Kids and Families. 2009.(99) | 8.3  (1.0) | Grade D | U | Diagnosis | ED Hosp |
|  |  | SEIZ04 | Children who presented with seizures had a history recorded including an assessment for epilepsy. | 4 | NSW Kids and Families. 2009.(99) | 8.5  (0.6) | Grade D | U | Diagnosis | ED Hosp |
|  |  | SEIZ05 | Children who presented with seizures had a history recorded including current medication and allergies. | 4 | NSW Kids and Families. 2009.(99) | 9  (0) | Grade D | U | Diagnosis | ED Hosp |
|  |  | SEIZ06 | Children who presented with seizures had a history recorded including an assessment of the potential for ingestion of medications or posions. | 5 | NSW Kids and Families. 2009.(99) | 8.2  (0.8) | Grade D | U | Diagnosis | ED Hosp |
|  |  | SEIZ07 | Children who presented with seizures had a history recorded including an assessment for anti-convulsant toxicity. | 4 | NSW Kids and Families. 2009.(99) | 7.5  (1.7) | Grade D | U | Diagnosis | ED Hosp |
|  |  | SEIZ08 | Children who presented with seizures had a history recorded including an assessment of their immunisation history. | 4 | NSW Kids and Families. 2009.(99) | 8  (2) | Grade D | U | Diagnosis | ED Hosp |
| 2 | Children presenting with seizures have evidence of the following assessments at the time of presentation:  pupillary size, reaction and symmetry AND  focal neurological signs during or after the seizure AND  signs of meningism AND  rashes or bruising which may indicate sepsis or injury AND  BP as soon as the seizure has ended. | SEIZ09 | Children who presented with seizures were, at the time of presentation, assessed for pupillary size, reaction and symmetry. | 4 | NSW Kids and Families. 2009.(99)  Scottish Intercollegiate Guidelines Network. 2005.(100) | 8.3  (1.0) | Consensus-based recommendation | U | Diagnosis | ED Hosp |
|  |  | SEIZ10 | Children who presented with seizures were, at the time of presentation, assessed for focal neurological signs during or after the seizure. | 4 | NSW Kids and Families. 2009.(99)  Scottish Intercollegiate Guidelines Network. 2005.(100) | 8.5  (0.6) | Consensus-based recommendation | U | Diagnosis | ED Hosp |
|  |  | SEIZ11 | Children who presented with seizures were, at the time of presentation, assessed for signs of meningism. | 5 | NSW Kids and Families. 2009.(99)  Scottish Intercollegiate Guidelines Network. 2005.(100) | 8  (2) | Consensus-based recommendation | U | Diagnosis | ED Hosp |
|  |  | SEIZ12 | Children who presented with seizures were, at the time of presentation, assessed for rashes or bruising which may indicate sepsis or injury. | 4 | NSW Kids and Families. 2009.(99)  Scottish Intercollegiate Guidelines Network. 2005.(100) | 8.8  (0.5) | Consensus-based recommendation | U | Diagnosis | ED Hosp |
| 3 | Children presenting with seizures have an assessment of their central capillary refill documented in the medical record. | SEIZ13 | Children who presented with seizures had an assessment of their central capillary refill. | 4 | NSW Kids and Families. 2009.(99)  Scottish Intercollegiate Guidelines Network. 2005.(100) | 7.3  (2.2) | Consensus-based recommendation | U | Diagnosis | ED Hosp |
| 4 | Children presenting with seizures have:  their blood glucose measured AND  if hypoglycaemic are given 2-5 mL/kg of 10 % dextrose OR IM glucagon (if no IV/IO access). | SEIZ14 | Children who presented with seizures had their blood glucose measured. | 4 | NSW Kids and Families. 2009.(99)  Scottish Intercollegiate Guidelines Network. 2005.(100) | 9  (0) | Consensus-based recommendation | U | Diagnosis | ED Hosp |
|  |  | SEIZ15 | Children who presented with seizures and were hypoglycaemic, were given: - 2-5 mL/kg of 10 % dextrose OR  - IM glucagon (if no IV/IO access). | 5 | NSW Kids and Families. 2009.(99)  Scottish Intercollegiate Guidelines Network. 2005.(100) | 8.7  (0.5) | Consensus-based recommendation | U | Treatment | ED Hosp |
| 5 | Children presenting with seizures who are suspected of having septicaemia or meningitis have:  a blood culture taken AND  then commenced on a broad spectrum antibiotic (third generation cephalosporin). | SEIZ16 | Children who presented with seizures and were suspected of having septicaemia or meningitis, had a blood culture taken. | 4 | NSW Kids and Families. 2009.(99)  Scottish Intercollegiate Guidelines Network. 2005.(100) | 9  (0) | Consensus-based recommendation | U | Treatment | ED Hosp |
|  |  | SEIZ17 | Children who presented with seizures and were suspected of having septicaemia or meningitis, were commenced on a broad spectrum antibiotic (third generation cephalosporin). | 4 | NSW Kids and Families. 2009.(99)  Scottish Intercollegiate Guidelines Network. 2005.(100) | 9  (0) | Consensus-based recommendation | U | Treatment | ED Hosp |
| 6 | Children presenting to ED with seizures have their vital signs recorded and reassessed as follows:  after each dose of anti-epileptic medication AND  every 15 minutes while seizure continues AND  every 30 minutes after a seizure until level of consciousness returns to normal. | SEIZ18 | Children who presented to ED with seizures had their vital signs recorded and reassessed after each dose of anti-epileptic medication. | 4 | NSW Kids and Families. 2009.(99) | 8.8  (0.5) | Consensus-based recommendation | U | Treatment | ED |
|  |  | SEIZ19 | Children who presented to ED with seizures had their vital signs recorded and reassessed every 15 minutes during the seizure. | 4 | NSW Kids and Families. 2009.(99) | 9  (0) | Consensus-based recommendation | U | Treatment | ED |
|  |  | SEIZ20 | Children who presented to ED with seizures had their vital signs recorded and reassessed every 30 minutes after a seizure until level of consciousness returned to normal. | 4 | NSW Kids and Families. 2009.(99) | 8.8  (0.5) | Consensus-based recommendation | U | Treatment | ED |
| 7 | Children presenting with seizures of 5 minutes or longer who have vascular access obtained immediately following presentation are treated with initial doses of medication as follows:  midazolam 0.1 - 0.2 mg/kg IV OR  diazepam 0.25mg/kg IV max 10mg | SEIZ21 | Children who presented with seizures of 5 minutes or longer, and who had vascular access obtained immediately following presentation were treated with initial doses of medication as follows: - midazolam 0.1 - 0.2 mg/kg IV OR - diazepam 0.25mg/kg IV max 10mg. | 4 | NSW Kids and Families. 2009.(99) | 8.7  (0.5) | Consensus-based recommendation | U | Treatment | ED Hosp |
| 8 | Children presenting with seizures who have vascular access and are still fitting 5 minutes (total time 5-10 minutes) after an initial dose of midazolam or diazepam have medication repeated as follows:  - midazolam 0.1 - 0.2 mg/kg IV OR  - diazepam 0.25 mg/kg IV. | SEIZ22 | Children who presented with seizures, had vascular access and were still fitting 5 minutes (total time 5-10 minutes) after an initial dose of midazolam or diazepam, had medication repeated as follows: - midazolam 0.1 - 0.2 mg/kg IV OR - diazepam 0.25 mg/kg IV. | 5 | NSW Kids and Families. 2009.(99) | 8.7  (0.5) | Consensus-based recommendation | U | Treatment | ED Hosp |
| 9 | "Children presenting with seizures who have vascular access (IV or IO) and are still fitting after a further 5 minutes are administered medication as follows:  - phenytoin 20mg/kg IV/IO over 20 minutes OR"  - phenobarbitone 20mg/kg IV/IO. | SEIZ23 | Children who presented with seizures, had vascular access (IV or IO) and were still fitting after a further 5 minutes, were administered medication as follows: - phenytoin 20mg/kg IV/IO over 20 minutes OR - phenobarbitone 20mg/kg IV/IO. | 4 | NSW Kids and Families. 2009.(99)  Scottish Intercollegiate Guidelines Network. 2005.(100) | 8.3  (1.0) | Grade B | U | Treatment | ED Hosp |
| 10 | Children presenting with seizures of 5 minutes or longer and where IV access cannot be obtained immediately following presentation are administered:  midazolam 0.3mg/kg buccal or intranasal (max 10mg) OR  midazolam 0.15mg/kg IM (max 5mg) OR  diazepam 0.5mg/kg PR (max 10mg). | SEIZ24 | Children who presented with seizures of at least 5 minutes duration, and where IV access could not be obtained immediately following presentation were administered: - midazolam 0.3mg/kg buccal or intranasal (max 10mg) OR - midazolam 0.15mg/kg IM (max 5mg) OR - diazepam 0.5mg/kg PR (max 10mg). | 4 | NSW Kids and Families. 2009.(99) | 8.5  (0.8) | Consensus-based recommendation | U | Treatment | ED Hosp |
| 11 | Children presenting with seizures who have no vascular access and continue to fit after an initial dose of midazolam or diazepam (total time 5-10 minutes) have a repeated dose of:  midazolam 0.3mg/kg buccal or midazolam 0.15mg/kg IM OR  diazepam 0.5mg/kg PR. | SEIZ25 | Children who presented with seizures, had no vascular access, and continued to fit after an initial dose of midazolam or diazepam (total time 5-10 minutes) received a repeated dose of: - midazolam 0.3mg/kg buccal or midazolam 0.15mg/kg IM OR - diazepam 0.5mg/kg PR. | 4 | NSW Kids and Families. 2009.(99) | 8.8  (0.5) | Consensus-based recommendation | U | Treatment | ED Hosp |
| 12 | Children presenting with seizures who after 30 minutes are still fitting are managed as follows:  referred to a consultant AND  given rapid sequence induction with thiopentone or propofol. | SEIZ26 | Children who presented with seizures and were still fitting after 30 minutes, were referred to a consultant. | 4 | NSW Kids and Families. 2009.(99)  Scottish Intercollegiate Guidelines Network. 2005.(100) | 8  (1.4) | Consensus-based recommendation | U | Treatment | ED Hosp |
|  |  | SEIZ27 | Children who presented with seizures and were still fitting after 30 minutes, were given rapid sequence induction with thiopentone or propofol. | 5 | NSW Kids and Families. 2009.(99)  Scottish Intercollegiate Guidelines Network. 2005.(100) | 8.3  (0.8) | Consensus-based recommendation | U | Treatment | ED Hosp |
| 13 | Children presenting with seizures have the following investigations performed:  Electrolytes collected at presentation if associated with repeated diarrhoea and vomiting AND  Calcium level measured if afebrile and seizures are new AND  Cerebral imaging if they have new focal signs AND  Lumbar puncture performed if suspected of having meningitis and the procedure is not contraindicated. | SEIZ28 | Children who presented with seizures and repeated diarrhoea and vomiting had electrolytes collected at presentation. | 4 | NSW Kids and Families. 2009.(99) | 8.5  (0.6) | Consensus-based recommendation | U | Treatment | ED Hosp |
|  |  | SEIZ29 | Children who presented with seizures and new focal signs had cerebral imaging. | 5 | NSW Kids and Families. 2009.(99) | 8.2  (1.0) | Consensus-based recommendation | U | Treatment | ED Hosp |
|  |  | SEIZ30 | Children who presented with seizures and were suspected of having meningitis had a lumbar puncture performed (if not contraindicated). | 4 | NSW Kids and Families. 2009.(99) | 9  (0) | Consensus-based recommendation | U | Treatment | ED Hosp |
| 14 | Children presenting to hospital with seizures are only discharged home after the following has occurred:  they have regained full consciousness AND  - they have a clear plan for medical follow up AND  - instructions for management of a recurrence. | SEIZ31 | Children who presented to hospital with seizures had regained full consciousness were discharged home. | 4 | NSW Kids and Families. 2009.(99) | 8.8  (0.5) | Consensus-based recommendation | U | Ongoing management | ED Hosp |
|  |  | SEIZ32 | Children who presented to hospital with seizures had a clear plan for medical follow up were discharged home. | 4 | NSW Kids and Families. 2009.(99) | 8.5  (0.6) | Consensus-based recommendation | U | Ongoing management | ED Hosp |
|  |  | SEIZ33 | Children who presented to hospital with seizures had received instructions for management of a recurrence were discharged home. | 4 | NSW Kids and Families. 2009.(99) | 8.8  (0.5) | Consensus-based recommendation | U | Ongoing management | ED Hosp |
| **TONSILLITIS** | | | | | | | | | | |
| 1 | Children aged 3-14 years who present with a sore throat have evidence in the medical record they were assessed for temperature greater than 38 degrees Celsius. | TONS01 | Children aged 3-14 years with a sore throat had their temperature assessed. | 4 | NSW Kids and Families. 2006.(101)  Scottish Intercollegiate Guidelines Network. 2010.(102)  Heart Foundation NZ TCSoAaNZ. 2007.(103)  Royal Childrens Hospital. 2009.(49)  Pelucchi C, Grigoryan L, Galeone C, et al. 2012.(104) | 7.3  (0.5) | Grade C | U | Diagnosis | ED  GP  Hosp  Spec |
| 2 | Children who present with a sore throat and no other symptoms or signs of tonsillitis should NOT be prescribed antibiotics. | TONS02 | Children with a sore throat and with no other symptoms or signs of tonsillitis were not prescribed antibiotics. | 4 | Scottish Intercollegiate Guidelines Network. 2010.(102)  Heart Foundation NZ TCSoAaNZ. 2007.(103)  Pelucchi C, Grigoryan L, Galeone C, et al. 2012.(104)  The Royal Children's Hospital Melbourne. 2009.(49) | 8.5  (1) | Grade A | O | Treatment | ED  GP  Hosp  Spec |
| 3 | Parents of children who present with a sore throat are instructed to provide symptomatic treatment which includes provision of fluids. | TONS03 | Parents of children with a sore throat were instructed to provide fluids. | 4 | NSW Kids and Families. 2006.(101)  Scottish Intercollegiate Guidelines Network. 2010.(102)  Heart Foundation NZ TCSoAaNZ. 2007.(103)  Pelucchi C, Grigoryan L, Galeone C, et al. 2012.(104) | 7  (0) | Grade A | U | Treatment | ED  GP  Hosp  Spec |
| 4 | Children aged less than 4 years who have a sore throat with associated cough who do not require hospitalisation are NOT prescribed antibiotics. | TONS04 | Children aged <4 years with a sore throat and associated cough who did not require hospitalisation were not prescribed antibiotics. | 4 | Royal Children's Hospital. 2009.(49) | 7  (1.4) | Consensus-based recommendation | O | Treatment | ED  GP  Hosp  Spec |
| 5 | Children aged 3-14 assessed as High Risk or GABHS positive and allergic to penicillin are prescribed oral Erythromycin. | TONS05 | Children aged 3-14 assessed as High Risk or GABHS positive and allergic to penicillin were prescribed oral Erythromycin. | 4 | Heart Foundation NZ TCSoAaNZ. 2007.(103)  Royal Children's Hospital. 2009.(49) | 8  (0) | Consensus-based recommendation | U | Treatment | ED  GP  Hosp  Spec |
| 6 | Children with recurrent acute sore throat due to tonsillitis are indicated for tonsillectomy if the following criteria are met:  episodes of sore throat are disabling and prevent normal functioning. | TONS06 | Children with recurrent acute sore throat with episodes that were disabling and prevented normal functioning were indicated for tonsillectomy. | 4 | Scottish Intercollegiate Guidelines Network. 2010.(102)  Royal Australasian College of Physicians\|Australian Society of Otolaryngology Head and Neck Surgery. 2008.(105)  Urquhart DSM, M. 2012.(106) | 7.7  (1.5) | Grade D | U | Ongoing management | ED  GP  Hosp  Spec |
| 7 | Children admitted to hospital having a tonsillectomy and adenoidectomy are managed as follows:  NOT administered perioperative antibiotics AND  given a stat dose of dexamethasone (0.15 - 1mg/kg; max dose 8-25mg) AND  prescribed anti-emetic medication post-surgery AND  informed of the potential for pain to increase for up to 6 days post-surgery.  informed parents/carers of the risk of post-operative haemorrhage: primary (within 24 hours) and secondary (4-9 days) after surgery. | TONS07 | Children who had a tonsillectomy and adenoidectomy were not administered perioperative antibiotics. | 4 | Scottish Intercollegiate Guidelines Network. 2010.(102)  Baugh RF, Archer SM, Mitchel RB, et al. 2011.(107) | 7  (0) | Consensus-based recommendation | O | Ongoing management | Hosp |
|  |  | TONS08 | Children who had a tonsillectomy and adenoidectomy were given a stat dose of dexamethasone. | 4 | Scottish Intercollegiate Guidelines Network. 2010.(102)  Baugh RF, Archer SM, Mitchel RB, et al. 2011.(107) | 7  (0) | Grade A | U | Ongoing management | Hosp |
|  |  | TONS09 | Children who had a tonsillectomy and adenoidectomy were prescribed anti-emetic medication post-surgery. | 4 | Scottish Intercollegiate Guidelines Network. 2010.(102)  Baugh RF, Archer SM, Mitchel RB, et al. 2011.(107) | 7  (0) | Grade A | U | Ongoing management | Hosp |
|  |  | TONS10 | Children who had a tonsillectomy and adenoidectomy were informed of the potential for pain to increase for up to 6 days post-surgery. | 4 | Scottish Intercollegiate Guidelines Network. 2010.(102)  Baugh RF, Archer SM, Mitchel RB, et al. 2011.(107) | 7  (0) | Grade D | U | Ongoing management | Hosp |
|  |  | TONS11 | Parents/carers of children who had a tonsillectomy and adenoidectomy were informed of the risk of post-operative haemorrhage: primary (within 24 hours) and secondary (4-9 days) after surgery. | 4 | Scottish Intercollegiate Guidelines Network. 2010.(102)  Baugh RF, Archer SM, Mitchel RB, et al. 2011.(107) | 7  (0) | Consensus-based recommendation | U | Ongoing management | Hosp |
| **UPPER RESPIRATORY TRACT INFECTION** | | | | | | | | | | |
| 1 | Children presenting with Upper Respiratory Tract Infection (URTI) have the following symptoms, risk factors and medical history documented:  rhinorrhea AND  cough AND  fever AND  comorbidities AND  previous medical history AND  all current medications AND  physical examination. | URTI01 | Children who presented with URTI symptoms had the presence of a runny nose (rhinorrhea) documented. | 7 | National Institute for Health and Care Excellence. 2008.(108)  Snellman L, Adams W, Anderson G, et al. 2013.(109) | 7  (1.4) | Consensus-based recommendation | U | Diagnosis | ED  GP  Hosp |
|  |  | URTI02 | Children who presented with URTI symptoms had the presence of a cough documented. | 7 | National Institute for Health and Care Excellence. 2008.(108)  Snellman L, Adams W, Anderson G, et al. 2013.(109) | 7.4  (1.8) | Consensus-based recommendation | U | Diagnosis | ED  GP  Hosp |
|  |  | URTI03 | Children who presented with URTI symptoms had the presence of a fever documented. | 7 | National Institute for Health and Care Excellence. 2008.(108)  Snellman L, Adams W, Anderson G, et al. 2013.(109) | 7.4  (2.1) | Consensus-based recommendation | U | Diagnosis | ED  GP  Hosp |
|  |  | URTI04 | Children who presented with an URTI had their comorbidities documented. | 7 | National Institute for Health and Care Excellence. 2008.(108)  Snellman L, Adams W, Anderson G, et al. 2013.(109) | 7.4  (1.5) | Consensus-based recommendation | U | Diagnosis | ED  GP  Hosp |
|  |  | URTI05 | Children who presented with an URTI had their previous medical history documented. | 7 | National Institute for Health and Care Excellence. 2008.(108)  Snellman L, Adams W, Anderson G, et al. 2013.(109) | 7.9  (1.2) | Consensus-based recommendation | U | Diagnosis | ED  GP  Hosp |
|  |  | URTI06 | Children who presented with an URTI had their current medications documented. | 7 | National Institute for Health and Care Excellence. 2008.(108)  Snellman L, Adams W, Anderson G, et al. 2013.(109) | 8.1  (1.2) | Consensus-based recommendation | U | Diagnosis | ED  GP  Hosp |
|  |  | URTI07 | Children who presented with an URTI had a physical examination. | 7 | National Institute for Health and Care Excellence. 2008.(108)  Snellman L, Adams W, Anderson G, et al. 2013.(109) | 8.6  (0.5) | Consensus-based recommendation | U | Diagnosis | ED  GP  Hosp |
| 2 | Clinicians should reassure parents that antibiotics are not needed immediately for URTI because of the following:  they are likely to make little difference to symptoms as the likeliest cause is viral AND  may have side effects (diarrhoea, vomiting and rash). | URTI08 | Parents of children with an URTI were advised against antibiotics as they are likely to make little difference to the symptoms. | 7 | National Institute for Health and Care Excellence. 2008.(108) | 8.8  (0.4) | Consensus-based recommendation | U | Treatment | ED  GP  Hosp |
|  |  | URTI09 | Parents of children with an URTI were advised against antibiotics as they may have side effects. | 7 | National Institute for Health and Care Excellence. 2008.(108) | 7  (2.8) | Consensus-based recommendation | U | Treatment | ED  GP  Hosp |
| 3 | Children with URTI who have the following diagnoses or comorbidities are prescribed antibiotics  pneumonia OR  peritonsillar abscess OR  bordetella pertussis OR  acute bacterial sinusitis with moderate / severe symptoms | URTI10 | Children with an URTI and pneumonia were prescribed antibiotics | 7 | National Institute for Health and Care Excellence. 2008.(108)  Wong DM, Blumberg DA, Lowe LG. 2006. | 8.2  (1.1) | Consensus-based recommendation | U | Treatment | ED  GP  Hosp |
|  |  | URTI11 | Children with an URTI and a peritonsillar abscess were prescribed antibiotics | 7 | National Institute for Health and Care Excellence. 2008.(108)  Wong DM, Blumberg DA, Lowe LG. 2006.(110) | 8.2  (1.8) | Consensus-based recommendation | U | Treatment | ED  GP  Hosp |
|  |  | URTI12 | Children with an URTI and bordetella pertussis were prescribed antibiotics | 7 | National Institute for Health and Care Excellence. 2008.(108)  Wong DM, Blumberg DA, Lowe LG. 2006.(110) | 7.8  (1.8) | Consensus-based recommendation | U | Treatment | ED  GP  Hosp |
|  |  | URTI13 | Children with an URTI and acute moderate / severe bacterial sinusitis were prescribed antibiotics. | 7 | National Institute for Health and Care Excellence. 2008.(108)  Wong DM, Blumberg DA, Lowe LG. 2006.(110) | 7.4  (2.2) | Consensus-based recommendation | U | Treatment | ED  GP  Hosp |
| 4 | Parents of children with an URTI should be advised to return if the condition worsens or becomes prolonged. | URTI14 | Parents of children with an URTI were advised to return if the condition worsens or becomes prolonged. | 7 | National Institute for Health and Care Excellence. 2008.(108) | 8.2  (1.8) | Consensus-based recommendation | U | Ongoing management | ED  GP  Hosp |
| **URINARY TRACT INFECTION** | | | | | | | | | |  |
| 1 | Children presenting with a suspected UTI have the following history documented that includes asking about presence of:  fever AND  vomiting AND  loin or abdominal pain | URIN01 | Children who presented with a suspected UTI have a history documented which included asking about the presence of fever. | 6 | Royal Children's Hospital Melbourne. 2011.(111)  National Institute of Health and Clinical Excellence. 2007.(112) | 8.7  (0.8) | Consensus-based recommendation | U | Diagnosis | ED Hosp  GP |
|  |  | URIN02 | Children who presented with a suspected UTI have a history documented which included asking about presence of vomiting. | 6 | Royal Children's Hospital Melbourne. 2011.(111)  National Institute of Health and Clinical Excellence. 2007.(112) | 7.8  (1.5) | Consensus-based recommendation | U | Diagnosis | ED Hosp  GP |
|  |  | URIN03 | Children who presented with a suspected UTI have a history documented which included asking about presence of loin or abdominal pain. | 6 | Royal Children's Hospital Melbourne. 2011.(111)  National Institute of Health and Clinical Excellence. 2007.(112) | 8.3  (0.8) | Consensus-based recommendation | U | Diagnosis | ED Hosp  GP |
| 2 | Children with a suspected UTI have the following documented:  temperature AND  palpable bladder or other abdominal masses (or have the absence of such masses documented) AND  Heart Rate AND  Capillary Refill Time AND  Respiratory Rate. | URIN04 | Children with a suspected UTI had their temperature documented. | 6 | Royal Children's Hospital Melbourne. 2011.(111)  National Institute of Health and Clinical Excellence. 2007.(112) | 8.8  (0.4) | Consensus-based recommendation | U | Diagnosis | ED Hosp  GP |
|  |  | URIN05 | Children with a suspected UTI had the presence of a palpable bladder or other abdominal masses (or have the absence of such masses) documented. | 6 | Royal Children's Hospital Melbourne. 2011.(111)  National Institute of Health and Clinical Excellence. 2007.(112) | 7.2  (1.8) | Consensus-based recommendation | U | Diagnosis | ED Hosp  GP |
|  |  | URIN06 | Children with a suspected UTI had their heart rate documented. | 5 | Royal Children's Hospital Melbourne. 2011.(111)  National Institute of Health and Clinical Excellence. 2007.(112) | 8.4  (0.9) | Consensus-based recommendation | U | Diagnosis | ED Hosp  GP |
|  |  | URIN07 | Children with a suspected UTI had their capillary refill time documented. | 5 | Royal Children's Hospital Melbourne. 2011.(111)  National Institute of Health and Clinical Excellence. 2007.(112) | 7.4  (1.8) | Consensus-based recommendation | U | Diagnosis | ED Hosp  GP |
|  |  | URIN08 | Children with a suspected UTI had their respiratory rate documented. | 5 | Royal Children's Hospital Melbourne. 2011.(111)  National Institute of Health and Clinical Excellence. 2007.(112) | 8.4  (0.9) | Consensus-based recommendation | U | Diagnosis | ED Hosp  GP |
| 3 | Children with a suspected UTI have documented that a clean specimen of urine was obtained for urinalysis. | URIN09 | Children with a suspected UTI had documented that a clean specimen of urine was obtained for urinalysis. | 6 | Royal Children's Hospital Melbourne. 2011.(111)  National Institute of Health and Clinical Excellence. 2007.(112) | 9  (0) | Consensus-based recommendation | U | Diagnosis | ED Hosp  GP |
| 4 | Infants aged less than 6 months with a confirmed UTI are managed as follows:  consulted with a paediatrician for consideration of parenteral antibiotics AND  have blood cultures taken if they have a fever and look unwell AND  given IV fluid resuscitation if shocked. | URIN10 | Infants aged less than 6 months with a confirmed UTI had a consult with a paediatrician for consideration of parenteral antibiotics. | 6 | Royal Children's Hospital Melbourne. 2011.(111)  National Institute of Health and Clinical Excellence. 2007.(112) | 7.8  (1.5) | Consensus-based recommendation | U | Treatment | ED Hosp |
|  |  | URIN11 | Infants aged less than 6 months with a confirmed UTI, fever, and who looked unwell had blood cultures taken. | 6 | Royal Children's Hospital Melbourne. 2011.(111)  National Institute of Health and Clinical Excellence. 2007.(112) | 8.8  (0.4) | Consensus-based recommendation | U | Treatment | ED Hosp |
|  |  | URIN12 | Infants aged less than 6 months with a confirmed UTI who were shocked, were given IV fluid resuscitation. | 6 | Royal Children's Hospital Melbourne. 2011.(111)  National Institute of Health and Clinical Excellence. 2007.(112) | 8.8  (0.4) | Consensus-based recommendation | U | Treatment | ED Hosp |
| 5 | Children aged greater than or equal to 6 months with a confirmed UTI who appear septic are managed as follows:  admitted for IV antibiotics AND  have blood cultures and electrolytes performed AND  given IV fluid resuscitation if shocked. | URIN13 | Children aged greater than or equal to 6 months with a confirmed UTI who appeared septic were admitted for IV antibiotics. | 6 | Royal Children's Hospital Melbourne. 2011.(111)  National Institute of Health and Clinical Excellence. 2007.(112) | 9 (0) | Consensus-based recommendation | U | Treatment | ED Hosp |
|  |  | URIN14 | Children aged greater than or equal to 6 months with a confirmed UTI who appeared septic had blood cultures and electrolytes performed. | 6 | Royal Children's Hospital Melbourne. 2011.(111)  National Institute of Health and Clinical Excellence. 2007.(112) | 8.8  (0.4) | Consensus-based recommendation | U | Treatment | ED Hosp |
|  |  | URIN15 | Children aged greater than or equal to 6 months with a confirmed UTI who appeared septic and were shocked, were given IV fluid resuscitation. | 6 | Royal Children's Hospital Melbourne. 2011.(111)  National Institute of Health and Clinical Excellence. 2007.(112) | 8.8  (0.4) | Consensus-based recommendation | U | Treatment | ED Hosp |
| 6 | Children with confirmed UTI are prescribed oral antibiotics as follows:  trimethoprim 4-6mg/kg (300 mg max) daily OR  trimethoprim and sulphamethoxazole 200mg sulphamethoxazole/40mg trimethoprim per 5 mLs (dose 4mg/kg of the trimethoprim component BD) (maximum 1600mg sulphamethoxazole/320 mg trimethoprim per day) OR  cephalexin 12.5-25mg/kg (500mg max) QID OR  Augmentin Duo 400mg amoxicillin/5 mLs (15mg/kg/dose if < 3 months, 22.5 mg/kg/dose if > 3 months) BD. | URIN16 | Children with confirmed UTI were prescribed oral antibiotics as follows: - trimethoprim 4-6mg/kg (300 mg max) daily OR - trimethoprim and sulphamethoxazole 200mg sulphamethoxazole/40mg trimethoprim per 5 mLs (dose 4mg/kg of the trimethoprim component BD) (maximum 1600mg sulphamethoxazole/320 mg trimethoprim per day) OR - cephalexin 12.5-25mg/kg (500mg max) QID OR - Augmentin Duo 400mg amoxicillin/5 mLs (15mg/kg/dose if < 3 months, 22.5 mg/kg/dose if > 3 months) BD. | 6 | Royal Children's Hospital Melbourne. 2011.(111)  National Institute of Health and Clinical Excellence. 2007.(112)  Therapeutic Guidelines. 2014.(113) | 7.8  (1.6) | Consensus-based recommendation | U | Treatment | ED Hosp |
| 7 | All infants and children with confirmed UTI who are treated with antibiotics have sensitivities assessed and if required the antibiotic regime adjusted within 48 hours of starting treatment. | URIN17 | All infants and children with confirmed UTI who were treated with antibiotics had sensitivities assessed, and if required the antibiotic regime adjusted within 48 hours of starting treatment. | 6 | Royal Children's Hospital Melbourne. 2011.(111)  National Institute of Health and Clinical Excellence. 2007.(112) | 8.7  (0.5) | Consensus-based recommendation | U | Treatment | ED Hosp |
| 8 | Children presenting with a UTI and any of the following are referred to the local paediatric team:  aged less than 6 months OR  known renal tract abnormalities OR  severely unwell requiring full septic workup. | URIN18 | Children aged less than 6 months, who presented with a UTI, were referred to the local paediatric team. | 6 | Royal Children's Hospital Melbourne. 2011.(111) | 7.8  (1.6) | Consensus-based recommendation | U | Treatment | ED Hosp |
|  |  | URIN19 | Children who presented with a UTI and known renal tract abnormalities, were referred to the local paediatric team. | 6 | Royal Children's Hospital Melbourne. 2011.(111) | 9  (0) | Consensus-based recommendation | U | Treatment | ED Hosp |
|  |  | URIN20 | Children who presented with a UTI, who were severely unwell, and required a full septic workshop, were referred to the local paediatric team. | 6 | Royal Children's Hospital Melbourne. 2011.(111) | 8.8  (0.4) | Consensus-based recommendation | U | Treatment | ED Hosp |
| 9 | Children who are treated for an atypical UTI have a renal ultrasound during the acute infection phase. | URIN21 | Children who were treated for an atypical UTI had a renal ultrasound during the acute infection phase. | 6 | National Institute of Health and Clinical Excellence. 2007.(112) | 8.3 (1) | Consensus-based recommendation | U | Treatment | ED Hosp |
| 10 | Children aged 6 months or under with a UTI who have responded well to treatment within 48 hours have a renal ultrasound within 6 weeks. | URIN22 | Children aged 6 months or under with a UTI who responded well to treatment within 48 hours, had a renal ultrasound within 6 weeks. | 5 | National Institute of Health and Clinical Excellence. 2007.(112) | 8.4  (1.3) | Consensus-based recommendation | U | Ongoing management | ED Hosp |
| 11 | Infants (aged < 6 months) with a UTI and who have an abnormal ultrasound, atypical UTI or recurrent UTI receive a voiding cystourethrogram (VCUG). | URIN23 | Infants (aged < 6 months) with a UTI and who had an abnormal ultrasound, atypical UTI or recurrent UTI, received a voiding cystourethrogram (VCUG). | 6 | National Institute of Health and Clinical Excellence. 2007.(112) | 7.5  (1.9) | Consensus-based recommendation | U | Ongoing management | ED Hosp |
| 12 | Children (aged > 3 years) with atypical or recurrent UTI do NOT receive a voiding cystourethrogram (VCUG). | URIN24 | Children (aged > 3 years) with atypical or recurrent UTI received a voiding cystourethrogram (VCUG). | 6 | Royal Children's Hospital Melbourne. 2011.(111)  National Institute of Health and Clinical Excellence. 2007.(112) | 8.2 (1.1) | Consensus-based recommendation | U | Ongoing management | ED Hosp |

**References**

1. The Royal Children's Hospital M. Abdominal pain 2013. Available from: <http://www.rch.org.au/clinicalguide/guideline_index/Abdominal_pain/>.

2. Macaluso CR, McNamara RM. Evaluation and management of acute abdominal pain in the emergency department. Neurology and Neurosurgery. International Journal of General Medicine. New Zealand: DOVE Medical Press Ltd.; 2012. p. 789-97.

3. Makin E, Davenport M. Evaluation of the acute abdomen. Paediatrics and Child Health (United Kingdom). 2012;22 (6):217-23.

4. Leung AKC, Sigalet DL. Acute abdominal pain in children. American Family Physician. 2003;67 (11):2321-6.

5. Cheung C. Approach to pediatric abdominal pain 2011. Available from: <http://learnpediatrics.sites.olt.ubc.ca/files/2011/11/abdominal-pain.pdf>.

6. Saucier A, Huang EY, Emeremni CA, Pershad J. Prospective evaluation of a clinical pathway for suspected appendicitis. Pediatrics. 2014;133(1):e88-95.

7. Holland A. Acute abdominal pain in children. Australian Doctor. 2009:25-32.

8. NSW Kids and Families. Infants and children: acute management of abdominal pain 2005. Available from: <http://www0.health.nsw.gov.au/policies/PD/2005/pdf/PD2005_384.pdf>.

9. NSW Kids and Families. Children and infants with gastroenteritis - acute management 2010. Available from: <http://www0.health.nsw.gov.au/policies/pd/2010/pdf/pd2010_009.pdf>.

10. Guarino A, Albano F, Ashkenazi S, Gendrel D, Hoekstra JH, Shamir R, et al. European Society for Paediatric Gastroenterology, Hepatology, and Nutrition/European Society for Paediatric Infectious Diseases evidence-based guidelines for the management of acute gastroenteritis in children in Europe: executive summary. Journal of Pediatric Gastroenterology and Nutrition. 2008;46 (5):619-21.

11. Acute Gastroenteritis Guideline Team Cincinnati Children's hospital medical center. Evidence based care guideline -Prevention and management of Acute Gastroenteritis (AGE) in children aged 2 months to 18 years Cincinnati, Ohio2011. Available from: <http://www.cincinnatichildrens.org/search/default/?q=prevention%20and%20management%20of%20acute%20gastroenteritis&start=0&site=>.

12. Heinz P. Management of acute gastroenteritis in children. Paediatrics and Child Health. 2008;18(10):453-7.

13. The Royal Children's Hospital Melbourne. Gastroenteritis Melbourne2013. Available from: <http://www.rch.org.au/clinicalguide/guideline_index/Gastroenteritis/>.

14. Farthing M, Lindberg G, Dite P, Khalif I, Salazar-Lindo E, Ramakrishna BS, et al. World Gastroenterology Organisation practice guideline: Acute diarrhea WGO Practice Guidelines. 2008:1-28.

15. Sydney Children's Hospital. Gastroenteritis Clinical Guideline Sydney2004. Available from: <http://www.sch.edu.au/health/professionals/cpg/gastroenteritis_clinical_guideline.pdf>.

16. National Institute for Health and Clinical Excellence (NICE). Diarrhoea and vomiting in children Diarrhoea and vomiting caused by gastroenteritis: diagnosis, assessment and management in children younger than 5 years London2009. Available from: <http://www.nice.org.uk/nicemedia/live/11846/47350/47350.pdf>.

17. Churgay CA, Aftab Z. Gastroenteritis in children: Part II. prevention and management. American Family Physician. 2012;85(11):1066-70.

18. Women's and Children's Health Network. Gastroenteritis Adelaide2010. Available from: <http://www.cyh.com/HealthTopics/HealthTopicDetails.aspx?p=114&np=303&id=1845>.

19. Kelly A, Cheong, E. Paediatric gastroenteritis Sydney2007. Available from: <http://www.australiandoctor.com.au/search?q=paediatric%20gastoenteritis>.

20. National Health and Medical Research Council. Clinical practice points on the diagnosis, assessment and management of Attention Deficit Hyperactivity Disorder in children and adolescents. Secondary Clinical practice points on the diagnosis, assessment and management of Attention Deficit Hyperactivity Disorder in children and adolescents 2012. Available from: <https://www.nhmrc.gov.au/guidelines/publications/mh26>.

21. American Academy of Pediatrics; Subcommittee on Attention-Deficit/Hyperactivity Disorder - Steering committee on quality improvement and management. ADHD: Clinical practice guideline for the diagnosis, evaluation, and treatment of Attention-Deficit.Hyperactivity Disorder in children and adolescents. . Pediatrics. 2011;128.

22. Kohn M. Child and adolescent ADHD. Australian Doctor. 2008(November).

23. National Institute for Health and Care Excellence (NICE). Attention deficit hyperactivity disorder: diagnosis and management 2008 updated 2016. Available from: <https://www.nice.org.uk/guidance/cg72>.

24. Royal Children's Hospital. Child psychiatry 2009. 187-201].

25. Sakolsky D, Birmaher B. Pediatric anxiety disorders: management in primary care. Current Opinion in Pediatrics. 2008;20(5):538-43.

26. British Columbia Medical Association. Anxiety and Depression in Children and Youth – Diagnosis and Treatment 2010. Available from: <http://www.bcguidelines.ca/pdf/depressyouth.pdf>.

27. American Academy of Child and Adolescent Psychiatry. Practice parameter for the assessment and treatment of children and adolescents with anxiety disorders. Journal of the American Academy of Child and Adolescent Psychiatry. 2007;46 (2):267-83.

28. National Institute for Health and Care Excellence (NICE). Social anxiety disorder: recognition, assessment and treatment 2013. Available from: <http://pathways.nice.org.uk/pathways/social-anxiety-disorder>.

29. Western Australian Therapeutic Advisory Group (WA TAG). "Antidepressant combination and augmentation" and "Antipsychotic Combinations". 2008. Available from: <http://www.watag.org.au/wapdc/guidelines.cfm>.

30. Canadian Psychiatric Association (CPA). Clinical practice guidelines: management of anxiety disorders 2006. Available from: <https://ww1.cpa-apc.org/Publications/CJP/supplements/july2006/anxiety_guidelines_2006.pdf>.

31. Madden S. How to treat anxiety disorder in children and adolescents. Australian Doctor. 2007(October 5):31-8.

32. British Thoracic Society, Scottish Intercollegiate Guidelines Network. British guideline on the management of asthma 2012 2012. Available from: <http://sign.ac.uk/guidelines/fulltext/101/>.

33. National Asthma Council Australia. Asthma Management Handbook 2006. National Asthma Council; 2006.

34. National Asthma Council Australia. <http://www.nationalasthma.org.au/handbook> 2013.

35. Royal Australasian College of Physicians. A consensus approach for the paediatrician's role in the diagnosis and assessment of Autism Spectrum Disorders in Australia 2008. Available from: <https://www.racp.edu.au/index.cfm?objectid=B55D4FBA-BFD6-A9E7-152508A3109AC225>.

36. Scottish Intercollegiate Guidelines Network (SIGN). Assessment, diagnosis and clinical interventions for children and young people with autism spectrum disorders 2007. Available from: <http://www.sign.ac.uk/guidelines/fulltext/98/index.html>.

37. National Institute for Health and Care Excellence (NICE). Autism diagnosis in children and young people: Recognition, referral and diagnosis of children and young people on the autism spectrum 2011. Available from: [www.nice.org.uk/guidance/cg128/resources/guidance-autism-diagnosis-in-children-and-young-people-pdf](http://www.nice.org.uk/guidance/cg128/resources/guidance-autism-diagnosis-in-children-and-young-people-pdf).

38. Tonge B, & Brereton A. Autism spectrum disorders. Australian Family Physician. 2011;40(9):672-77.

39. Australian Government Department of Health and Ageing. Early intervention for children with autism spectrum disorders: guidelines for best practice 2007. Available from: [www.health.gov.au/internet/publications/publishing.nsf/Content/mental-child-autbro-toc~mental-child-autbro-best](http://www.health.gov.au/internet/publications/publishing.nsf/Content/mental-child-autbro-toc~mental-child-autbro-best) - no longer available online.

40. SA Child Health Clinical Network. Clinical Guideline: Management of Bronchiolitis in children 2013. Available from: <http://www.sahealth.sa.gov.au/wps/wcm/connect/0a3fd50040d03f4d96fbbe40b897efc8/Bronchiolitis+in+Children_Aug2013.pdf?MOD=AJPERES&CACHEID=0a3fd50040d03f4d96fbbe40b897efc8>.

41. NSW Kids and Families. Infant and Children - Acute Management of Bronchiolitis 2012. Available from: <http://www0.health.nsw.gov.au/policies/pd/2012/pdf/PD2012_004.pdf>.

42. The Royal Children's Hospital Melbourne. Bronchiolitis Guideline. Secondary Bronchiolitis Guideline 2012 2012. Available from: <http://www.rch.org.au/clinicalguide/guideline_index/Bronchiolitis_Guideline/>.

43. The Royal Children's Hospital Melbourne. Bronchiolitis - Ongoing management. Secondary Bronchiolitis - Ongoing management 2013. Available from: <http://www.rch.org.au/clinicalguide/guideline_index/Bronchiolitis_Guideline/>.

44. Sydney Children's Hospital. Viral Bronchiolitis Inpatient Clinical Guidelines Sydney2011. Available from: <http://www.sch.edu.au/health/professionals/cpg/viral_bronchiolitis_inpatient_clinical_guidelines.pdf>.

45. Zentz SE. Care of Infants and Children With Bronchiolitis: A Systematic Review. Journal of Pediatric Nursing. 2011;26(6):519-29.

46. American Academy of paediatrics (AAP): subcommittee on diagnosis and management of bronchiolitis. Diagnosis and management of bronchiolitis. Pediatrics. 2006;118 (4):1774-93.

47. Scottish Intercollegiate Guidelines Network (SIGN). Bronchiolitis in children - a national clinical guideline no.91 Edinburgh2006. Available from: <http://www.sign.ac.uk/pdf/sign91.pdf>.

48. Princess Margaret Hospital for Children. Child and adolescent health service. Clinical Practice Guideline: Bronchiolitis 2008. Available from: <http://www.pmh.health.wa.gov.au/development/manuals/clinical_practice_guidelines/documents/bronchiolitis_cpg.pdf>.

49. Royal Childrens Hospital Melbourne. Paediatric Handbook - Eighth edition. Melbourne, Australia: Wiley-Blackwell; 2009.

50. The Royal Children's Hospital Melbourne. Clinical practice guidelines: Croup (Laryngotacheobronchitis). 2011. Available from: <http://www.rch.org.au/clinicalguide/guideline_index/Croup_Laryngotracheobronchitis/>.

51. NSW Health. Children and infants - Acute management of croup 2010. Available from: <http://www0.health.nsw.gov.au/policies/pd/2010/PD2010_053.html>.

52. Rajapaksa S., M. S. Croup: Assessment and management. Australian Family Physician 2010;39(5):280-82.

53. Health for Kids in the South East SH. Evidence-based practice guideline for the management of croup in children 2007. Available from: No longer available online.

54. Sydney West Area Health Service. Nurse Practitioner clinical practice guidelines for the management of croup 2004. Available from: No longer available online.

55. Harrison J, J. M. Acute respiratory infections in children. Australian Doctor. 2009(May):27-34.

56. Association. AM. Guideline for the diagnosis and management of croup. 2009. Available from: <http://www.topalbertadoctors.org/download/252/croup_guideline.pdf>.

57. Campus JH. Nurse Practitioner - emergency services: Clinical practice guideline management of croup. 2006. Available from: <http://www.nursing.health.wa.gov.au/docs/career/np/joondalup/CPG_Croup.pdf>.

58. National Institute for Health and Care Excellence (NICE). Croup 2012.

59. The Royal Children's Hospital Melbourne. Diabetes Mellitus. Secondary Diabetes Mellitus 2013. Available from: <http://www.rch.org.au/clinicalguide/guideline_index/Diabetes_Mellitus/>.

60. Australasian Paediatric Endocrine Group, and the Australian Diabetes Society. National evidence-based clinical care guidelines for type 1 diabetes in children, adolescents and adults. 2011. Available from: <https://www.nhmrc.gov.au/guidelines-publications/ext4>.

61. Royal Children's Hospital Melbourne. Child psychiatry. 2009:187-201.

62. NHMRC. Clinical practice guidelines: depression in adolescents and young adults. 2011. Available from: <https://www.nhmrc.gov.au/guidelines/publications/ext0007>.

63. National Institute for health and Clinical Excellence (NICE). Depression in children and young people: Identification and management in primary, community and secondary care 2005.

64. Cheung AH, Zuckerbrot RA, Jensen PS, Ghalib K, Laraque D, Stein REK. Guidelines for adolescent depression in primary care (GLAD-PC): II. Treatment and ongoing management. Paediatrics. 2007;120(55):e1313-26.

65. A. ZR, Cheung AH, Jensen PS, Stein REK, Laraque D. Guidelines for adolescent depression in primary care (GLAD-PC): I. Identification, assessment and initial management. Pediatrics. 2007;12(5):1299-312.

66. The Royal Australian and New Zealand College of Psychiatrists (RANZCP), The Royal Australian College of General Practitioners (RACGP) & The Royal Australian College of Physicians (RACP). Clinical guidance on the use of antidepressant medications in children and adolescents. 2005. Available from: <https://www.ranzcp.org/Files/Resources/College_Statements/Practice_Guidelines/Clinical_Guidance_on_the_use_of_Antidepressant_med.aspx>.

67. Royal Children's Hospital M. Eczema management. Secondary Eczema management 2013. 2013. Available from: <http://www.rch.org.au/rchcpg/hospital_clinical_guideline_index/Eczema_management/>.

68. Scottish Intercollegiate Guidelines Network (SIGN). Management of atopic eczema in primary care (CG125). Secondary Management of atopic eczema in primary care (CG125) 2011. Available from: <http://www.sign.ac.uk/guidelines/fulltext/125/index.html>.

69. SA Child Health Clinical Network. Management of fever without focus in children (excluding neonates). Secondary Management of fever without focus in children (excluding neonates) 2013. Available from: <http://www0.health.nsw.gov.au/policies/pd/2010/PD2010_063.html>.

70. National Institute for Health and Clinical Excellence. Feverish illness in children: assessment and initial management in children younger than 5 years 2013.

71. Cincinnati Children's Hospital Medical Center. Evidence-based care guideline for fever of uncertain source in infants 60 days of age or less. 2010. Available from: <http://www.cincinnatichildrens.org/workarea/downloadasset.aspx?id=87913>.

72. The Royal Children's Hospital Melbourne. Febrile child. 2011. Available from: <http://www.rch.org.au/clinicalguide/guideline_index/Febrile_Child/>.

73. NSW Kids and Families. Policy directive: Children and infants with fever - acute management. 2010. Available from: <http://www0.health.nsw.gov.au/policies/pd/2010/PD2010_063.html>.

74. Queensland Government Children's Health Service Royal Children's Hospital. Gastro-oesophageal reflux in children over 4 years. 2011 updated 9 December 2016. Available from: <http://www.health.qld.gov.au/rch/professionals/forms/Gastro_RefluxOver4yrs.pdf>.

75. Queensland Government Children's Health Service Royal Children's Hospital. Gasto-oesophageal reflux - regurgitation in infants 2011 updated 2016. Available from: <http://www.health.qld.gov.au/rch/professionals/forms/Gastro_Reflux.pdf>

76. The Royal Children's Hospital Melbourne. Gastrooesophageal reflux in infants Melbourne2012. Available from: <http://www.rch.org.au/clinicalguide/guideline_index/Gastrooesophageal_Reflux_in_infants/>.

77. Vandenplas Y, Rudolph CD, Di Lorenzo C, Hassall E, Liptak G, Mazur L, et al. Pediatric gastroesophageal reflux clinical practice guidelines: Joint recommendations of the North American Society for Pediatric Gastroenterology, Hepatology, and Nutrition (NASPGHAN) and the European Society for Pediatric Gastroenterology, Hepatology, and Nutrition (ESPGHAN). Journal of Pediatric Gastroenterology and Nutrition. 2009;49(4):498-547.

78. Lightdale JR, Gremse DA, Heitlinger LA, Cabana M, Gilger MA, Gugig R, et al. Gastroesophageal reflux: Management guidance for the pediatrician. Pediatrics. 2013;131(5):e1684-e95.

79. Bhavsar H, Cullen M, Beattie RM. Gastro-oesophageal reflux in infancy. Paediatrics and Child Health. 2011;21(9):394-400.

80. Allen K. Gastro-oesophageal reflux in children. Australian Family Physician. 2012;41(5):268-72.

81. Families NKa. Children and infants - acute management of head injury 2011. Available from: <http://www0.health.nsw.gov.au/policies/pd/2011/pdf/PD2011_024.pdf>.

82. The Royal Children's Hospital Melbourne. Trauma - Head Injury ND. Available from: <http://www.rch.org.au/clinicalguide/guideline_index/Head_Injury_Guideline/>.

83. National Health & Medical Research Council (NHMRC). Clinical practice guidelines for the management of overweight and obesity in adults, adolescents and children in Australia Canberra 2013. Available from: <http://www.nhmrc.gov.au/_files_nhmrc/publications/attachments/n57>.

84. Scottish Intercollegiate Guidelines Network (SIGN). Management of obesity: A national clinical guideline (no. 115) Edinburgh2010. Available from: <http://www.sign.ac.uk/guidelines/fulltext/115/index.html>.

85. Lau DC, Douketis JD, Morrison KM, Hramiak IM, Sharma AM, Ur E. 2006 Canadian clinical practice guidelines on the management and prevention of obesity in adults and children [summary]. CMAJ : Canadian Medical Association journal = journal de l'Association medicale canadienne. 2007;176 (8):S1-13.

86. Ministry of Health - Clinical Trials Research Unit. Clinical Guidelines for Weight Management in New Zealand Children and Young People Wellington2009. Available from: <http://www.health.govt.nz/publication/clinical-guidelines-weight-management-new-zealand-children-and-young-people>.

87. Calonge N, Petitti DB, DeWitt TG, Dietrich A, Gregory KD, Grossman D, et al. Screening for obesity in children and adolescents: US preventive services task force recommendation statement. Pediatrics. 2010;125 (2):361-7.

88. Barlow SE. Expert committee recommendations regarding the prevention, assessment, and treatment of child and adolescent overweight and obesity: summary report. Pediatrics. 2007;120 Suppl 4:S164-92.

89. August GP, Caprio S, Fennoy I, Freemark M, Kaufman FR, Lustig RH, et al. Prevention and treatment of pediatric obesity: An Endocrine Society clinical practice guideline based on expert opinion. Journal of Clinical Endocrinology and Metabolism. 2008;93 (12):4576-99.

90. National Institute for Health and Clinical Excellence (NICE). Obesity: guidance on the prevention, identification, assessment and management of overweight and obesity in adults and children (CG43). London. 2006.

91. Gunasekera H. Otitis media in children. Australian Doctor. 2008(July ):33-40.

92. Families NKa. Children and infants with otitis media - acute management 2005. Available from: <http://www0.health.nsw.gov.au/policies/PD/2005/pdf/PD2005_385.pdf>.

93. British Columbia Medical Association. British Columbia Ministry of Health Services guidelines & protocols advisory committee. Otitis media: acute otitis media (AOM) & otitis media with effusion (OME) 2010.

94. Lieberthal AS, Carroll AE, Chonmaitree T, Ganiats TG, Hoberman A, Jackson MA, et al. The diagnosis and management of acute otitis media. Pediatrics. 2013;131 (3):e964-e99.

95. Scottish Intercollegiate Guidelines Network (SIGN). Diagnosis and management of childhood otitis media in primary care (national guideline 66) Edinburgh2003. Available from: <http://www.sign.ac.uk/pdf/sign66.pdf>.

96. Royal Children's Hospital Brisbane. Secondary Otitis media - acute suppurative otitis media (ASOM): All ages 2010. Available from: <http://www.health.qld.gov.au/rch/professionals/forms/ENT_OtitisMedia.pdf>.

97. Department of Health Western Australia. Otitis Media Model of Care 2013. Available from: <http://www.healthnetworks.health.wa.gov.au/netnews/Otitis-moc.pdf>

98. Royal Australian College of General Practitioners. Guidelines for preventive activities in general practice 8th edition - Preventive activities in children and young people. 2013. Available from: <http://www.racgp.org.au/your-practice/guidelines/redbook/>.

99. NSW Kids and Families. Children and infants with seizures - acute management 2009. Available from: <http://www0.health.nsw.gov.au/policies/pd/2009/PD2009_065.html>.

100. Scottish Intercollegiate Guidelines Network. Diagnosis and management of epilepsies in children and young people 2005. Available from: <http://www.sign.ac.uk/pdf/sign81.pdf>.

101. NSW Kids and Families. Children and infants with sore throats - acute management. 2006. Available from: <http://www0.health.nsw.gov.au/policies/pd/2006/pdf/PD2006_019.pdf>.

102. Scottish Intercollegiate Guidelines Network (SIGN). Management of sore throat and indications for tonsillectomy Edinburgh2010. Available from: <http://www.sign.ac.uk/pdf/sign117.pdf>.

103. TCSoAaNZ. HFN. GUIDE: for sore throat management 2007. Available from: <http://www.world-heart-federation.org/fileadmin/user_upload/documents/RHD-net/AUS_NZ_resources/Guidelines/NZRh_F_Algorithm_4.pdf>.

104. Pelucchi C, Grigoryan L, Galeone C, Esposito S, Huovinen P, Little P, et al. Guideline for the management of acute sore throat: ESCMID Sore Throat Guideline Group C. Pelucchi et al. Guideline for management of acute sore throat. Clinical Microbiology and Infection. 2012;18 (SUPPL.1):1-28.

105. Royal Australasian College of Physicians|Australian Society of Otolaryngology Head and Neck Surgery. Indications for Tonsillectomy and Adenotonsillectomy In Children 2008. Available from: <http://www.mdconsult.com.ezlibproxy.unisa.edu.au/website/view?linkfrom=pg&URL=http%3A%2F%2Fwww.racp.edu.au%2Findex.cfm%3Fobjectid%3DB5637C7B-E823-E407-E65AB8D6F27A07BD>.

106. Urquhart DS, Montague ML. When to remove tonsils and the alternatives. Paediatrics and Child Health. 2012;22 (1):37-41.

107. Baugh RF, Archer SM, Mitchell RB, Rosenfeld RM, Amin R, Burns JJ, et al. Clinical practice guideline: Tonsillectomy in children. Otolaryngology - Head and Neck Surgery. 2011;144 (SUPPL.1):S1-S30.

108. National Institute for Health and Care Excellence (NICE). Respiratory tract infections - antibiotic prescribing: Prescribing of antibiotics for self-limiting respiratory tract infections in adults and children in primary care 2008. Available from: <https://www.google.com.au/#q=Respiratory+tract+infections+-+antibiotic+prescribing:+Prescribing+of+antibiotics+for+self-limiting+respiratory+tract+infections+in+adults+and+children+in+primary+care>.

109. Snellman L AW, Anderson G, Godfrey A, Gravley A, Johnson K, Marshall P, Myers C, Nesse R, Short S. Institute for Clinical Systems Improvement. Diagnosis and Treatment of Respiratory Illness in Children and Adults 2013. Available from: <https://www.icsi.org/_asset/1wp8x2/RespIllness.pdf>.

110. Wong DM, Blumberg DA, Lowe LG. Guidelines for the use of antibiotics in acute upper respiratory tract infections. American Family Physician. 2006;74(6):956-66+69.

111. Royal Children's Hospital Melbourne. Urinary Tract Infection Guideline Melbourne2011. Available from: <http://www.rch.org.au/clinicalguide/guideline_index/Urinary_Tract_Infection_Guideline/>.

112. National Institute for Health and Care Excellence (NICE). Urinary tract infection in under 16s: diagnosis and management 2007. Available from: <https://www.nice.org.uk/guidance/CG54>.

113. Therapeutic Guidelines eTG complete. Urinary tract infections in children Melbourne2014. Available from: <https://tgldcdp.tg.org.au/viewTopic?topicfile=urinary-tract-infections&guidelineName=Antibiotic#toc_d1e751>.
