# Supplementary material for: Clinical indicators for common paediatric conditions: Processes, provenance and products of the CareTrack Kids study
Source: PLoS One. 2019 Jan 9;14(1):e0209637. doi: 10.1371/journal.pone.0209637 (PMC6326465; doi:10.1371/journal.pone.0209637)
Supplement: S2 Table — (DOCX) [file pone.0209637.s002.docx]

**S2 Table. *CareTrack Kids* excluded original recommendations from included clinical practice guidelines.**

Subtypes of conditions ANXI (i.e. panic, obsessive-compulsive, post-traumatic stress, social anxiety disorders) and DEPR (i.e. bipolar disorder, psychotic depression) and their accompanying recommendations were not included.

|  | **Condition** | **Original Recommendation** | **Source** | | | **Rationale for exclusion** |
| --- | --- | --- | --- | --- | --- | --- |
|  |  |  | **Reference** | **Grade** | **Level of evidence** |  |
| 1 | ABDO | Clinicans should consider the age of the child as certain conditions are more common in particular age groups. Age is a key factor in the evaluation of abdominal pain. | Makin 2012(1)    Cheung 2011(2)  Leung 2003(3) |  |  | Strength/certainty of wording (“should consider”) |
| 2 | ABDO | Investigations will depend on the differential diagnosis but may include the following:  - urine MCS  - blood sugar for DKA  - electrolytes +/- LFTs  - Lipase (pancreatitis)  - urine pregnancy test/ quantitative beta hCG  - Imaging  ◦AXR if obstruction suspected. Not helpful in diagnosing constipation.  ◦CXR if pneumonia suspected  ◦Ultrasound (may be requested after discussion with senior staff, is not clinically indicated for testicular torsion) | RCH Melb 2013(4) |  |  | Strength/certainty of wording (“may include”) |
| 3 | ABDO | Transfer or referral to tertiary centre if the child requiring care is beyond the comfort level of the hospital | RCH Melb 2013(4) |  |  | Recommendation is not measurable (“comfort level of the hospital”) |
| 4 | ADHD | ADHD is a description rather than an explanation of a pervasive, persistent, disabling pattern of inattentiveness, overactivity and/or impulsivity. A child/adolescent who meets diagnostic criteria for ADHD may not be always best served by making that diagnosis. For example, their behaviour could be understood as a reaction to specific cognitive difficulties or family/environmental circumstances. | NHMRC 2012(5) |  |  | Description of the condition |
| 5 | ADHD | Where indicated, young children (under 7 years) and school age children diagnosed with ADHD, and their families, may benefit from evidence-based psychological interventions that have demonstrated effectiveness for associated mental health problems. Such interventions can improve outcomes for internalising (emotional) and externalising (behavioural) symptoms. Not all psychological approaches have an evidence base, and only those where this is the case should be implemented. | NHMRC 2012(5)  Kohn M 2008(6) |  |  | Strength/certainty of wording (“may”) |
| 6 | ADHD | In prioritising intervention and management, consideration should be given to the ability of the child/adolescent and their parents/carers to implement strategies. These interventions may be impractical for families due to time demands and cost associated with them or may not be locally available particularly in some rural areas. | NHMRC 2012(5)  Kohn M 2008(6) |  |  | Strength/certainty of wording (“consideration”, “may be”) |
| 7 | ADHD | Use of stimulant medications (methylphenidate and dexamphetamine sulphate) can reduce core ADHD symptoms and improve social skills and peer relations in children and adolescents diagnosed with ADHD in the short term (up to 3 years). | NHMRC 2012(5) |  |  | Strength/certainty of wording (“can”) |
| 8 | ADHD | The optimal dose of stimulant medication can be titrated against clinical benefit and may be prescribed in various forms of the medications, including: immediate release, extended release or both. | NHMRC 2012(5)  AAP 2011(7) | Grade B - Strong (AAP) |  | Strength/certainty of wording (“can”, “may”) |
| 9 | ADHD | Consideration of the changing needs of patients with ADHD as they transition through school and into adult life is imperative to the provision of optimal clinical care. | NHMRC 2012(5) |  |  | Guiding statement (“consideration”) |
| 10 | ADHD | In terms of improving behavioural and academic outcomes, children/adolescents may benefit from individually tailored modifications to the educational setting and curriculum, as informed by the overall case formulation. These modifications could include classroom based approaches that restructure how information is presented to students with ADHD and should form part of an individual education plan for the child (or its equivalent), at their school. | NHMRC 2012(5) |  |  | Guiding statement (“could include”) |
| 11 | ANXI | DSM-IV-TR diagnostic criteria:  - There must be evidence of the capacity for age-appropriate social relationships with familiar people and the anxiety must occur in peer settings, not just in interactions with adults  - The anxiety may be expressed by crying, tantrums, freezing, or shrinking from social situations with  unfamiliar people  - May not recognize that the fear is excessive or unreasonable  - In individuals < 18 years,  - the duration is at least 6 months | CPA 2006(8) |  |  | Guiding statement (included as definition in surveyors’ manual) |
| 12 |  | To determine the level of anxiety the following instruments and questionnaires may be useful:  - Anxiety disorder interview schedule  - Diagnostic interview schedule for children IV  - Revised child manifest anxiety scale  - Childhood depression inventory  - Children's depression rate scale  - Yale brown compulsive scale | RCH paed handbook 2009(9) |  |  | Strength/certainty of wording (“may be useful”) |
| 13 | ANXI | SOC, as defined by the Diagnostic and Statistical Manual Mental Disorders (4th Edition) [DSM-IV],[1] is characterized by the following:  • marked and persistent fear about being subjected to embarrassment or humiliation in social situations such as public speaking, starting or holding conversations, eating in front of others, dating, playing sports, going to parties, or any other circumstance that may involve being scrutinized or evaluated by others;  • when exposed to these situations, children and adolescents experience intense anxiety that interferes with their normal functioning;  • the fears are excessive or unreasonable;  • the child or adolescent avoids feared social or performance situations whenever possible, or endures them with intense distress;  • the duration of illness is at least 6 months; and  • the symptoms cannot be better explained by another condition. | Khalid-Khan 2007(10) |  |  | Generalised anxiety only included (not within audit sample or scope) |
| 14 | ANXI | Social Anxiety Phobia (SAD) – At least 6 months of noticeable and persistent fear of being negatively evaluated for at least one social or performance situation. The child fears appearing anxious or incompetent, resulting in feelings of embarrassment and humiliation and marked social avoidance of both peers and adults (e.g. tantrums, crying, freezing or shrinking from social situations with unfamiliar people). Social phobia is often undiagnosed as the child is seen only as shy with strangers. Developmental loss in learning social competence at this age may result in long term vulnerability to leading a marginal life and depression. | British Columbia 2010(11)  O'Brien 2012(12) |  |  | Generalised anxiety only included (not within audit sample or scope) |
| 15 | ANXI | As part of a comprehensive assessment, assess for causal and maintaining factors for social anxiety disorder in the child or young person's home, school and social environment, in particular:  - parenting behaviours that promote and support anxious behaviours or do not support positive behaviours  - peer victimisation in school or other settings | NICE 2013(13) |  |  | Generalised anxiety only included (not within audit sample or scope) |
| 16 | ANXI | As part of a comprehensive assessment, assess for possible coexisting conditions such as:  - other mental health problems (for example, other anxiety disorders and depression)  - neurodevelopmental conditions such as attention deficit hyperactivity disorder, autism and learning disabilities  - drug and alcohol misuse  - speech and language problems. | NICE 2013(13) |  |  | Generalised anxiety only included (not within audit sample or scope) |
| 17 | ANXI | To aid the assessment of social anxiety disorder and other common mental health problems consider using formal instruments (both the child and parent versions if available and indicated), such as:  - the LSAS – child version or the Social Phobia and Anxiety Inventory for Children (SPAI-C) for children, or the SPIN or the LSAS for young people  - the Multidimensional Anxiety Scale for Children (MASC), the Revised Child Anxiety and Depression Scale (RCADS) for children and young people who may have comorbid depression or other anxiety disorders, the Spence Children's Anxiety Scale (SCAS) or the Screen for Child Anxiety Related Emotional Disorders (SCARED) for children. | NICE 2013(13)  Khalid-Khan 2007(10) |  |  | Generalised anxiety only included (not within audit sample or scope) |
| 18 | ANXI | Use formal assessment instruments to aid the diagnosis of other problems, such as:  - a validated measure of cognitive ability for a child or young person with a suspected learning disability  - the Strengths and Difficulties Questionnaire for all children and young people. | NICE 2013(13) |  |  | Generalised anxiety only included (not within audit sample or scope) |
| 19 | ANXI | All interventions for children and young people with social anxiety disorder should be delivered by competent practitioners. Psychological interventions should be based on the relevant treatment manual(s), which should guide the structure and duration of the intervention. Practitioners should consider using competence frameworks developed from the relevant treatment manual(s) and for all interventions should:  - receive regular high-quality supervision  - use routine sessional outcome measures, for example:  (LSAS – child version or the SPAI-C, and the SPIN or LSAS for young people or the MASC, RCADS, SCAS or SCARED for children)  - engage in monitoring and evaluation of treatment adherence and practitioner competence – for example, by using video and audio tapes, and external audit and scrutiny if appropriate. | NICE 2013(13) |  |  | Structure-level recommendation (not within audit sample or scope) |
| 20 | ANXI | Offer individual or group CBT focused on social anxiety to children and young people with social anxiety disorder. Consider involving parents or carers to ensure the effective delivery of the intervention, particularly in young children. | NICE 2013(13) |  |  | Generalised anxiety only included (not within audit sample or scope) |
| 21 | ANXI | Do not routinely offer pharmacological interventions to treat social anxiety disorder in children and young people. | NICE 2013(13) |  |  | Generalised anxiety only included (not within audit sample or scope) |
| 22 | ANXI | Panic Disorder – At least one panic attack (acute episode of unprovoked, intense fear/distress accompanied by somatic or cognitive symptoms) resulting in worry about having another panic attack and its implications or engaging in significant behavioural avoidance. | British Columbia 2010(11) |  |  | Guiding statement |
| 23 | ANXI | Panic Disorder  • The magnitude of fear of bodily sensations which are perceived as similar to those of panic is often underestimated. Patients benefit from watching family physicians and parents model interoceptive exposure (i.e. simulation exercises) involving rapid breathing, spinning, etc., and then slowly have the child or youth do the same thing along with the clinician. This helps build tolerance.  • Ensure that young patients understand that panic attacks are safe, painless, private and brief. Encourage them to have them, wherever they are. This helps patients view them as increasingly incidental.  • The treatment of choice for agoraphobia is behavioural exposure. The most common problem in its treatment is that exposure is not daily or focuses only on one theme at a time (e.g. going to the mall). Exposure should be robust and creative. Patients will tolerate exposure better if accompanied at first by a trusted other and then if the trusted other arranges to meet them in the target area after the client has accomplished planned exposure alone. | British Columbia 2010(11) |  |  | Guiding statement |
| 24 | ANXI | Generalized Anxiety Disorder (GAD) – At least 6 months of excessive and uncontrollable daily worry about multiple themes (e.g. school, family safety, world issues, natural calamities, friends, personal performance) that results in physical symptoms (e.g. fatigue, irritability, restlessness, poor concentration, muscle tension, sleep disturbance) and functional impairment. | British Columbia 2010(11)  O’Brien 2012 |  |  | Guiding statement |
| 25 | ANXI | Generalized Anxiety Disorder  • Children and youth relate well to the concept of chaining of thoughts, where one worry provokes another, and the need to “break the chain”.  • Focus on 2-3 worry themes in the same week, instead of completing one worry hierarchy before addressing another.  • Help children and youth with GAD make frequent “behavioural experiments” to test their worries (“if that were true, what would you predict will happen tomorrow when…? Let’s see if it is true.”), collect the evidence for or against the prediction and then modify the prediction and belief accordingly (“Well maybe it doesn’t happen very often”).  • Help patients reframe worries into problem solving (“What could you do to help ensure this doesn’t happen?).  • Have patients estimate the possibility and probability of a feared event occurring within a particular time frame, and help them draw the appropriate conclusions when their biases are revealed. | British Columbia 2010(11) |  |  | Guiding statement |
| 26 | ASTH | In children with an intermediate probability of asthma who can perform spirometry and have no evidence of airways obstruction:  - consider testing for atopic status, bronchodilator reversibility and if possible, bronchial  hyper-responsiveness using methacholine, exercise or mannitol  - consider specialist referral. | BTS and SIGN 2012(14) |  |  | Strength/certainty of wording (“consider”) |
| 27 | ASTH | In some children, particularly the under 5s, there is insufficient evidence for a firm diagnosis of asthma but no features to suggest an alternative diagnosis.  Possible approaches (dependent on frequency and severity of symptoms) include:  - watchful waiting with review  - trial of treatment with review  - spirometry and reversibility testing. | BTS and SIGN 2012(14) |  |  | Strength/certainty of wording (“possible approaches include”) |
| 28 | ASTH | If exercise is a specific problem in patients taking inhaled steroids who are otherwise well controlled, consider adding one of the following therapies:  - leukotriene receptor antagonists  - long-acting β2 agonists  - chromones  - oral β2 agonists  - theophyllines. | BTS and SIGN 2012(14) |  |  | Strength/certainty of wording (“consider”) |
| 29 | ASTH | ACUTE ASTHMA - Children and adults with mild and moderate exacerbations of asthma should be treated by pMDI + spacer with doses titrated according to clinical response. | BTS and SIGN 2012(14) |  |  | Guiding statement  Covered in other recommendations |
| 30 | ASTH | STABLE ASTHMA - In children aged 5-12, pMDI + spacer is as effective as any other hand held inhaler. | BTS and SIGN 2012(14) |  |  | Guiding statement  Covered in other recommendations |
| 31 | ASTH | INHALED STEROIDS FOR STABLE ASTHMA - In children aged 5-12 years, pMDI + spacer is as effective as any DPI. | BTS and SIGN 2012(14) |  |  | Guiding statement  Covered in other recommendations |
| 32 | ASTH | INHALER DEVICES in children under 5 - In young (0-5 years) children, little or no evidence is available on which to base recommendations | BTS and SIGN 2012(14) |  |  | Guiding statement  Covered in other recommendations |
| 33 | ASTH | In children aged 0-5 years, pMDI and spacer are the preferred method of delivery of β2 agonists or inhaled steroids. A face mask is required until the child can breathe reproducibly using the spacer mouthpiece. Where this is ineffective a nebuliser may be required. | BTS and SIGN 2012(14) |  |  | Guiding statement  Covered in other recommendations |
| 34 | ASTH | ACUTE SEVERE  SpO2 <92% PEF 33-50%  - Can’t complete sentences in one breath or too breathless to talk or feed  - Pulse >125 (>5 years) or >140 (2 to 5 years)  - Respiration >30 breaths/min (>5 years) or >40 (2 to 5 years) | BTS and SIGN 2012(14) |  |  | Definition (included in surveyors’ manual) |
| 35 | ASTH | LIFE THREATENING  SpO2 <92% PEF<33-50% best or predicted  - Hypotension  - Silent chest  - Exhaustion  - Cyanosis  - Confusion  - Poor respiratory effort  - Coma | BTS and SIGN 2012(14) |  |  | Definition (included in surveyors’ manual) |
| 36 | ASTH | Treat children transported to hospital by ambulance with oxygen and nebulised β2 agonists during the journey. | BTS and SIGN 2012(14) |  |  | Ambulance and paramedic records not assessed in main study audit. |
| 37 | ASTH | Paramedics attending to children with acute asthma should administer nebulised salbutamol driven by oxygen if symptoms are severe whilst transferring the child to the emergency department. | BTS and SIGN 2012(14) |  |  | Ambulance and paramedic records not assessed in main study audit. |
| 38 | ASTH | Consider early addition of a single bolus dose of IV salbutamol (15 mcg/kg over 10 minutes) in severe cases where the patient has not responded to initial inhaled therapy. | BTS and SIGN 2012(14) |  |  | Strength/certainty of wording (“consider”) |
| 39 | ASTH | Consider aminophylline in an HDU or PICU setting for children with severe or life threatening bronchospasm unresponsive to maximal doses of bronchodilators plus steroids. | BTS and SIGN 2012(14)  NAC 2006 |  |  | Strength/certainty of wording (“consider”) |
| 40 | ASTH | The assessment of acute asthma in early childhood can be difficult  Intermittent wheezing attacks are usually due to viral infection and the response to asthma medication is inconsistent.  The differential diagnosis of symptoms includes:  - aspiration pneumonitis  - pneumonia  - bronchiolitis  - tracheomalacia  - complications of underlying conditions such as congenital anomalies and cystic fibrosis.  Prematurity and low birth weight are risk factors for recurrent wheezing | BTS and SIGN 2012(14) |  |  | Guiding statement |
| 41 | ASTH | For mild to moderate acute asthma, a pMDI+spacer is the optimal drug delivery device. | BTS and SIGN 2012(14) |  |  | Guiding statement  Covered in other recommendations |
| 42 | ASTH | Consider steroid tablets in infants early in the management of moderate to severe episodes of acute asthma in the hospital setting. | BTS and SIGN 2012(14) |  |  | Strength/certainty of wording (“consider”) |
| 43 | ASTH | Consider inhaled ipratropium bromide in combination with an inhaled β2 agonist for more severe symptoms. | BTS and SIGN 2012(14)  NAC 2006(15) |  |  | Strength/certainty of wording (“consider”) |
| 44 | ASTH | All people with asthma should have access to primary care services delivered by doctors and nurses with appropriate training in asthma management. | BTS and SIGN 2012(14) |  |  | Structure-level recommendation (not within audit sample or scope) |
| 45 | ASTH | Consider carrying out routine reviews by telephone for people with asthma. | BTS and SIGN 2012(14) |  |  | Structure-level recommendation (not within audit sample or scope)  Guiding statement (“consider”) |
| 46 | ASTH | General practices should maintain a register of people with asthma. Clinical review should be structured and utilise a standard recording system | BTS and SIGN 2012(14) |  |  | Structure-level recommendation (not within audit sample or scope) |
| 47 | ASTH | Feedback of audit data to clinicians should link guidelines recommendations to management of individual patients. | BTS and SIGN 2012(14) |  |  | Structure-level recommendation (not within audit sample or scope) |
| 48 | ASTH | Healthcare professionals who provide asthma care should have heightened awareness of the complex needs of ethnic minorities, socially disadvantaged group, adolescents, the elderly and those with communication difficulties. | BTS and SIGN 2012(14) |  |  | Structure-level recommendation (not within audit sample or scope) |
| 49 | ASTH | Manage hospital inpatients in specialist rather than general units. | BTS and SIGN 2012(14) |  |  | Structure-level recommendation (not within audit sample or scope) |
| 50 | ASTH | Clinicians in primary and secondary care should treat asthma according to recommended guidelines. | BTS and SIGN 2012(14) |  |  | Structure-level recommendation (not within audit sample or scope) |
| 51 | ASTH | An acute consultation offers the opportunity to determine what action the patient has already taken to deal with the exacerbation. Their self-management strategy may be reinforced or refined and the need for consolidation at a routine follow up considered | BTS and SIGN 2012(14) |  |  | Guiding statement (“may be”)  Covered in other recommendations |
| 52 | ASTH | A consultation for an upper respiratory tract infection, or other known trigger, is an opportunity to rehearse self-management in the event of their asthma deteriorating | BTS and SIGN 2012(14) |  |  | Guiding statement  Covered in other recommendations |
| 53 | ASTH | Brief simple education linked to patient goals is most likely to be acceptable to patients. | BTS and SIGN 2012(14) |  |  | Strength/certainty of wording (“may be”)  Covered in other recommendations |
| 54 | ASTH | Computer repeat-prescribing systems provide a useful index of compliance. | BTS and SIGN 2012(14) |  |  | Structure-level recommendation (not within audit sample or scope) |
| 55 | AUTI | Acknowledge initial concerns regarding development and behaviour raised in the community by families, carers or service providers. | RACP 2008(16)  SIGN 2007(17) | Grade D |  | Guiding statement |
| 56 | AUTI | Use clinical judgement regarding the possible presence of an autism spectrum disorder and do not rely solely on screening tools. | RACP 2008(16) |  |  | Guiding statement |
| 57 | AUTI | Reassure parents where development and behaviour are judged to be within normal limits and arrange a review appointment. | RACP 2008(16) |  |  | Guiding statement  Relates to children without a diagnosis of ASD (covered in suite of Preventive Care indicators) |
| 58 | AUTI | Encourage parents to initiate earlier review should they have ongoing concerns. | RACP 2008(16) |  |  | Guiding statement  Relates to children without a diagnosis of ASD (covered in suite of Preventive Care indicators) |
| 59 | AUTI | Multidisciplinary assessment of development/cognition, language, play skills and sensory sensitivities contribute essential information to help with planning appropriate management and early intervention. | Tonge B, Brereton A. 2011(18)  SIGN 2007(17) | Grade D |  | Guiding statement  Covered in other recommendations |
| 60 | AUTI | Actively consult with those involved in the care of the child about the extent and nature of difficulties in development and behaviour including social interaction, communication, interests and play. | RACP 2008(16)  DOHA 2007(19) |  |  | Guiding statement  Covered in other recommendations |
| 61 | AUTI | Provide a diagnosis of one of the autism spectrum disorders fulfilling DSM-IV or ICD-10 classifications at the conclusion of Stage 2. For DSM-IV these are Autistic Disorder 299.00, Asperger’s Disorder 299.80 or Pervasive Developmental Disorder Not Otherwise Specified (including Atypical Autism) 299.80. For ICD-10 these are Childhood autism F84.0, Atypical autism F84.1 or Asperger’s syndrome F84.5. | RACP 2008(16)  DOHA 2007(19) |  |  | Guiding statement  Included as definitions in the surveyors’ manual. |
| 62 | AUTI | Consider and discuss the risk of recurrence of ASD or associated developmental disorders | RACP 2008(16) |  |  | Guiding statement (“consider”) |
| 63 | AUTI | Proceed to Stage 4: Discussion and Intervention Planning, when a clear diagnosis has been made. If the diagnosis remains unclear proceed to Stage 3: Autism Spectrum Disorder specific assessment. | RACP 2008(16) |  |  | Guiding statement  Covered in other recommendations |
| 64 | AUTI | Active review of all information gathered and procedures performed at Stage 2, and updates and supplements as required. | RACP 2008(16) |  |  | Guiding statement  Covered in other recommendations |
| 65 | AUTI | Consider available local resources able to provide this assessment or refer to a diagnosis and assessment specialist team, where there is uncertainty about diagnosis or where for example, educational, adaptive and psychosocial factors combine to complicate the presentation, requiring an autism spectrum disorder specific assessment. | RACP 2008(16) |  |  | Strength/certainty of wording (“consider”) |
| 66 | AUTI | Employ the use of autism specific observation and parent interview tools where necessary. Examples of these include the Autism Diagnostic Observation Schedule (ADOS), Autism Diagnostic Interview – Revised (ADI-R) and Diagnostic Interview for Social and Communication Disorders (DISCO). These tools require specialist training, and experience, and additional time and resources. Naturalistic observations in home, preschool and school settings may also be appropriate. | RACP 2008(16) |  |  | Guiding statement (“where necessary”) |
| 67 | AUTI | Recognise the high support needs and intervention requirements of the child who presents with a complex neurodevelopmental disorder for whom a diagnosis of one of the autism spectrum disorders is not met. | RACP 2008(16) |  |  | Guiding statement  Covered in other recommendations |
| 68 | AUTI | Assist in identifying a case manager or key worker. Most likely this will be the parent or carer of the child. However this is not always the situation and Paediatricians and other agencies also fulfil this role, often in partnership. | RACP 2008(16) |  |  | Guiding statement  Covered in other recommendations |
| 69 | AUTI | Intervention should begin as early as possible in the child’s life, (optimally between 2 and 4 years). | DOHA 2007(19) |  |  | Guiding statement.  Recommendation not measurable (dependent on child and parent presentation). |
| 70 | AUTI | xiii) Transition  There should be systematic connection and integration between the early intervention program and the next stage for the child, whether it is transition to school or to another therapeutic or special educational setting. Parents, teachers and therapists need to collaborate in preparing the child for transition. | DOHA 2007(19) |  |  | Guiding statement.  Recommendation not measurable (covered in other recommendations) |
| 71 | AUTI | Recognising and diagnosing autism before pre-school age has been uncommon until the last few years. But increasingly autism is being identified very early in development. It has been shown that diagnosis can be valid and reliable at 2 years of age, and signs can be recognisable and predictive of autism even from early in the second year of life. In future it is likely that autism will be diagnosed for most children in the toddler age period (18 - 30 months). Very early therapeutic intervention is likely to improve developmental and adaptive outcomes so it will be necessary to develop, implement and evaluate interventions for this age group in Australia. Trials of interventions in the USA and UK are focusing on training parents to work with their very young children in the key areas of social responsiveness, attention skills, early communication skills, and interactive play. | DOHA 2007(19) |  |  | Guiding statement. |
| 72 | AUTI | Management of anxiety and depression includes altering the environment to reduce stress and anxiety, creating the experience of successful achievement at school, psychological treatments such as cognitive behavioural therapy modified to take account of the child’s cognitive abilities, and the use of the selective serotonin reuptake inhibitor (SSRI) fluoxetine in some cases. | Tonge B, Brereton A. 2011(18) |  |  | Guiding statement. |
| 73 | BRON | Supplemental oxygen is indicated if oxyhemoglobin saturation (SpO2) falls persistently below 90% in previously healthy infants. If the SpO2 does persistently fall below 90%, adequate supplemental oxygen should be used to maintain SpO2 at or above 90%. Oxygen may be discontinued if SpO2 is at or above 90% and the infant is feeding well and has minimal respiratory distress. | AAP 2006(20) | AAP - option: D |  | Contradictory to recommendations published across multiple more recent clinical practice guidelines (NSW Health 2012, SIGN 2006, Zetnz 2011). |
| 74 | BRON | A carefully monitored trial of α-adrenergic or β-adrenergic medication is an option. Inhaled bronchodilators should be continued only if there is a documented positive clinical response to the trial using an objective means of evaluation | AAP 2006(20) | AAP - option: B |  | Guiding statement (“an option”) |
| 75 | BRON | If asthma is considered a possibility, in infants aged six to twelve months, order a standard stat dose, (e.g. salbutamol via a nebuliser or via a metered dose aerosol with spacer device), watch it being given, assess and record the effects before deciding whether to order more | NSW Health 2012(21)  SCH 2011(22) |  |  | Guiding statement (“considered an option”).  Recommendation not measurable (“before deciding whether to order more”) |
| 76 | BRON | Immunoprophylactic medications may decrease hospitalisation rates in some infants but evidence is equivocal. It should be considered on an individual basis in infants considered to be at high risk | NSW Health 2012(21) |  |  | Guiding statement |
| 77 | BRON | Clinicians may administer palivizumab prophylaxis to selected infants and children with CLD or a history of prematurity (less than 35 weeks’ gestation) or with congenital heart disease | AAP 2006(20) | AAP - option: A |  | Guiding statement |
| 78 | CROU | Children with croup should be allowed to adopt the position that they find most comfortable.  Do not forcibly change a child's posture - they will adopt the posture that minimises airways obstruction. | HKSE 2007(23)  RCH Melb 2011(24) |  |  | Recommendation not measurable (unlikely to be documented). |
| 79 | CROU | If a General Practitioner is not available, children with moderate croup should be taken to a hospital emergency department. | NSW Health 2010(25) | Grade D |  | Recommendation not measurable (unlikely to be documented). |
| 80 | CROU | Where possible nebulisation should be driven by oxygen. | HKSE 2007(23) | Grade D |  | Guiding statement (“where possible”).  Recommendation not measurable (covered in other recommendations) |
| 81 | DIAB | Clinicians should be aware that the co-occurrence of psychological disorders in type 1 diabetes is common | Aust Diabetes Society 2011(26) | Grade A |  | Guiding statement (covered in other recommendations) |
| 82 | DEPR | Psychological therapies used in the treatment of children and young people should be provided by therapists who are also trained child and adolescent mental healthcare professionals.  Services should agree minimum training criteria for healthcare professionals engaging in psychological therapy.  Healthcare professionals delivering psychological therapies should meet agreed minimum criteria. | NICE 2005(27) |  |  | Structure-level recommendation (not within audit sample or scope) |
| 83 | DEPR | Healthcare professionals in primary care, schools and other relevant community settings should be trained to detect children and young people who may be at risk of depression. Training should include the evaluation of recent and past psychosocial risk factors, such as age, gender, family discord, bullying, physical, sexual or emotional abuse, comorbid disorders, including drug and alcohol use, and a history of parental depression; the natural history of single loss events; the importance of multiple risk factors; ethnic and cultural factors; and factors known to be associated with high a risk of depression and other health problems, such as homelessness, refugee status and living in institutional settings  Services should have training programmes for tier 1 professionals that address:  • detection of depressive symptoms  • assessment of risk factors for depression  • culturally sensitive systems for detecting and supporting children and young people with depression | NICE 2005(27) |  |  | Structure-level recommendation (not within audit sample or scope) |
| 84 | DEPR | Child and Adolescent Mental Health Services (CAMHS) tier 2 or 3 should work with health and social care professionals in primary care, schools and other relevant community settings to provide training and  develop ethnically and culturally sensitive systems for detecting, assessing, supporting and referring children and young people who  are either depressed or at significant risk of becoming depressed | NICE 2005(27) |  |  | Structure-level recommendation (not within audit sample or scope) |
| 85 | DEPR | Training opportunities should be made available to improve the accuracy of CAMHS professionals in diagnosing depressive conditions. The existing interviewer-based instruments (such as Kiddie-Sads [K-SADS] and Child and Adolescent Psychiatric Assessment [CAPA]) could be used for this purpose but will require modification for regular use in busy routine CAMHS settings.  Services should have training programmes for CAMHS professionals across all tiers that address the detection and diagnosis of depression in children and young people. | NICE 2005(27) |  |  | Structure-level recommendation (not within audit sample or scope) |
| 86 | DEPR | If a PC clinician identifies an adolescent with moderate or severe depression or complicating factors/conditions such as coexisting substance abuse or psychosis, consultation with a mental health specialist should be considered (C). | Cheung et al 2007(28) |  | C | Strength/certainty of wording (“should be considered”) |
| 87 | DEPR | Prescription of the selective serotonin reuptake inhibitor (SSRI) fluoxetine should be considered for acute, short-term reduction of depressive symptoms in adolescents with moderate to severe major depressive disorder, where psychological therapy has not been effective, is not available or is refused, or if symptoms are severe. | NHMRC 2011(29) |  | B | Strength/certainty of wording (“should be considered”) |
| 88 | DEPR | Recommendation 3: For patients who achieve only partial improvement after PC diagnostic & therapeutic approaches have been exhausted (including exploration of poor adherence, comorbid disorders and ongoing conflicts or abuse), a mental health consultation should be considered (D) | Cheung et al 2007(28) |  | D | Strength/certainty of wording (“should be considered”) |
| 89 | DEPR | Choose an appropriate diagnostic questionnaire available for download on the internet. If the screen indicates a possible problem then either begin or schedule time to begin a detailed inquiry about anxiety or depressive symptoms, evaluate severity and the potential for self-harm. Consider that there may be more than one psychiatric disorder when screening because anxiety and depressive disorders are highly comorbid in children and adolescents. | British Columbia 2010(11) |  |  | Guiding statement (covered in other recommendations) |
| 90 | DEPR | Individual interviews of the child or adolescent to help understand the nature of the symptoms and their impact as age appropriate. This will also help with building a therapeutic rapport with the child or adolescent. | RCH paed handbook 2009(9) |  |  | Guiding statement (covered in other recommendations) |
| 91 | DEPR | Unless specifically excluded by the child or young person, parent(s) or carer(s) should have the opportunity to be involved in decisions about the child or young person’s care and treatment. | NICE 2005(27) |  |  | Guiding statement (covered in other recommendations) |
| 92 | DEPR | In most cases it is beneficial to involve the young person’s parents/carers in discussions about his or her care. However, the degree of involvement will depend on the young person’s age, stage of development, wishes and circumstances. | NHMRC 2011(29) |  |  | Guiding statement (covered in other recommendations) |
| 93 | DEPR | Family involvement is invaluable for assisting with and monitoring treatments as well as providing assurance and emotional support for the child or youth. Assure the family that the questions and fact-finding is not to assign blame but to better understand the situation. | British Columbia 2010(11) |  |  | Guiding statement (covered in other recommendations) |
| 94 | DEPR | With the young person’s consent, multiple informants should be involved to assist in identifying possible causes of the young person’s distress and providing information about any changes in his or her behaviour or functioning. | NHMRC 2011(29) |  |  | Guiding statement (covered in other recommendations) |
| 95 | DEPR | Structured instruments and questionnaires may be useful, These include:  - Anxiety Disorder interview  - Diagnostic Interview Schedule for Children IV  - Revised Child Manifest Anxiety Scale  - Childhood Depression Inventory  - Children's Depression Rate Scale  - Yale Brown Obsessive Compulsive Scale | RCH paed handbook 2009(9) |  |  | Guiding statement (covered in other recommendations) |
| 96 | DEPR | Children and young people with depression should have the opportunity to make informed decisions about their care and treatment, but this does depend on their age and capacity to make decisions. It is good practice for healthcare professionals to involve the young person’s parent(s) or carer(s) in the decision-making process. Where a child or young person is not old enough or does not have the capacity to make decisions, healthcare professionals should follow the Department of Health guidelines – Reference guide to consent for examination or treatment (2001) (available from www.dh.gov.uk). | NICE 2005(27) |  |  | Guiding statement (covered in other recommendations) |
| 97 | DEPR | Psychosocial interventions of the types investigated to date are not currently recommended for universal prevention of depressive symptoms or major depressive disorder in the adolescent population. More research is needed to identify effective approaches. | NHMRC 2011(29) |  |  | Guiding statement |
| 98 | DEPR | For children who experience a family-related risk factor for depression, family-focused interventions should be considered for the prevention of major depressive disorder in adolescence. | NHMRC 2011(29) |  |  | Strength/certainty of wording (“should be considered”) |
| 99 | DEPR | Cognitive behavioural interventions should be considered for short-term symptom reduction in adolescents with identified depressive symptoms who do not meet diagnostic criteria for major depressive disorder. | NHMRC 2011(29) |  |  | Strength/certainty of wording (“should be considered”) |
| 100 | DEPR | Psychosocial interventions of the types investigated to date are not currently recommended for universal prevention of depressive symptoms or major depressive disorder in the adolescent population. More research is needed to identify effective approaches. | NHMRC 2011(29) |  |  | Guiding statement |
| 101 | DEPR | For children who experience a family-related risk factor for depression, family-focused interventions should be considered for the prevention of major depressive disorder in adolescence. | NHMRC 2011(29) |  |  | Strength/certainty of wording (“should be considered”) |
| 102 | DEPR | Cognitive behavioural interventions should be considered for short-term symptom reduction in adolescents with identified depressive symptoms who do not meet diagnostic criteria for major depressive disorder. | NHMRC 2011(29) |  |  | Strength/certainty of wording (“should be considered”) |
| 103 | DEPR | Given the lack of evidence in young adults, it is strongly recommended that strategies to prevent major depressive disorder in this age group be a focus for continuing research. | NHMRC 2011(29) |  |  | Guiding statement |
| 104 | DEPR | Preventive strategies in young adults should be guided by findings in adolescents until more evidence is available. | NHMRC 2011(29) |  |  | Guiding statement |
| 105 | DEPR | Healthcare professionals in primary care, schools and other relevant community settings should be trained to detect symptoms of depression, and to assess children and young people who may be at risk of depression. Training should include the evaluation of recent and past psychosocial risk factors, such as age, gender, family discord, bullying, physical, sexual or emotional abuse, comorbid disorders, including drug and alcohol use, and a history of parental depression; the natural history of single loss events; the importance of multiple risk factors; ethnic and cultural factors; and factors known to be associated with a high risk of depression and other health problems, such as homelessness, refugee status and living in institutional settings. | NICE 2005(27) |  |  | Structure-level recommendation (not within audit sample or scope) |
| 106 | DEPR | Child and Adolescent Mental Health Services (CAMHS) tier 2 or 3 should work with health and social care professionals in primary care, schools and other relevant community settings to provide training and develop ethnically and culturally sensitive systems for detecting, assessing, supporting and referring children and young people who are either depressed or at significant risk of becoming depressed. | NICE 2005(27) |  |  | Structure-level recommendation (not within audit sample or scope) |
| 107 | DEPR | Note red flags for bipolar depression: family history, psychotic depression, mania with SSRIs, hyper sexuality, risk-taking behaviour and pre-pubertal depression. Consider referral if bipolar depression is suspected. Manage the patient while waiting for referral (see management considerations) and provide follow up. | British Columbia 2010(11) |  |  | Guiding statement  Strength/certainty of wording (“consider referral”) |
| 108 | DEPR | Health professionals involved in the care of young people must take the time to build strong therapeutic relationships, which will form the basis of continuing care. | NHMRC 2011(29) |  |  | Guiding statement (covered in other recommendations) |
| 109 | DEPR | Recognition is important as untreated childhood depression increases the risk for depression in adulthood | RCH paed handbook 2009(9) |  |  | Guiding statement (covered in other recommendations) |
| 110 | DEPR | Additionally, depression in childhood (and particularly in adolescence) increases the risk of suicide and self-harming behaviours. | RCH paed handbook 2009(9) |  |  | Guiding statement (covered in other recommendations) |
| 111 | DEPR | Training opportunities should be made available to improve the accuracy of CAMHS professionals in diagnosing depressive conditions. The existing interviewer-based instruments (such as Kiddie-Sads [K-SADS] and Child and Adolescent Psychiatric Assessment [CAPA]) could be used for this purpose but will require modification for regular use in busy routine CAMHS settings | NICE 2005(27) |  |  | Structure-level recommendation (not within audit sample or scope) |
| 112 | DEPR | Following multidisciplinary review, the following should be  considered:  • an alternative psychological therapy which has not been tried previously  (individual CBT, interpersonal therapy or shorter-term family therapy, of at  least 3 months’ duration), or  • systemic family therapy (at least 15 fortnightly sessions), or  • individual child psychotherapy (approximately 30 weekly sessions). | NICE 2005(27) | B |  | Strength/certainty of wording (“should be considered”) |
| 113 | DEPR | 1.6.4 How to use antidepressants in children and young people  All antidepressant drugs have significant risks when given to children and young people with depression and, with the exception of fluoxetine, there is little evidence that they are effective in this context. Although fluoxetine can cause significant adverse drug reactions, it is safer when combined with psychological therapies. The following guidance outlines how fluoxetine should be used, and suggests possible alternatives in the event that fluoxetine is ineffective or not tolerated because of side effects. | NICE 2005(27) |  |  | Guiding statement (covered in other recommendations) |
| 114 | DEPR | Prescription of the selective serotonin reuptake inhibitor (SSRI) fluoxetine should be considered for acute, short-term reduction of depressive symptoms in adolescents with moderate to severe major depressive disorder, where psychological therapy has not been effective, is not available or is refused, or if symptoms are severe. | NHMRC 2011(29) |  |  | Strength/certainty of wording (“should be considered”) |
| 115 | DEPR | CBT may be added to/continued with SSRI therapy, to reduce the risk of suicidal thinking and improve functioning in adolescents with major depressive disorder. | NHMRC 2011(29) |  |  | Strength/certainty of wording (“may be”) |
| 116 | DEPR | In the absence of more substantial evidence on treating depression in young adults, it is reasonable to extrapolate from the evidence on pharmacological treatment in adolescents (see Recommendations 5, 6 and 7; last 3 items directly above). | NHMRC 2011(29) |  |  | Guiding statement (covered in other recommendations) |
| 117 | DEPR | Health professionals should be aware of the risk that a manic episode may be precipitated following initiation of SSRIs. For young people with depressive episodes and a history of mania or mixed presentations, a mood stabiliser may be required. | NHMRC 2011(29) |  |  | Guiding statement (covered in other recommendations) |
| 118 | DEPR | While there is a small evidence base, current good clinical practice suggests continuing medication therapy for 6 months post-remission. | NHMRC 2011(29) |  |  | Strength/certainty of recommendation (“suggests”) |
| 119 | DEPR | Where SSRI medication is warranted, a combined SSRI plus CBT/IPT approach appears to provide the most effective care. If a moderate to severe depressive disorder fails to respond to combined therapy, specialist advice or a second opinion should be sought. | NHMRC 2011(29) |  |  | Guiding statement (covered in other recommendations) |
| 120 | DEPR | If discontinuation of treatment is planned, consideration needs to be given to factors that may contribute to relapse and recurrence. | NHMRC 2011(29) |  |  | Strength/certainty of wording (“consideration”) |
| 121 | DEPR | Complex presentations may require a longer assessment phase and multiple interventions delivered by different health professionals. Overall case management by one health professional is advisable. | NHMRC 2011(29) |  |  | Strength/certainty of wording (“may”, “advisable”) |
| 122 | DEPR | Electroconvulsive therapy (ECT) may be considered in rare cases, such as treating severe depression with psychotic features where other approaches have not been successful. | NHMRC 2011(29) |  |  | Strength/certainty of wording (“may be considered”) |
| 123 | DEPR | If inpatient care is required, admission should be to an environment designed for young people wherever possible. | NHMRC 2011(29) |  |  | Structure-level recommendation (not within audit sample or scope) |
| 124 | DEPR | Referral to Specialist  Indications for referral to a specialist:  • Depression or anxiety that has not responded to primary treatment  • High suicide risk  • Severe OCD and panic  • Persistent school avoidance  • Possible bipolarity  • Postpartum depression | British Columbia 2010(11) |  |  | Guiding statement (covered in other recommendations) |
| 125 | DEPR | Referral options (also refer to other resources in the Resource List for Physicians)  • Continue to follow the patient until they are seen by the specialist  • For specialist mental health consultation and CBT refer to MCFD - Child and Youth Mental Health (250 387-7027 (Greater Victoria) or toll free 1 877 387-7027); or contact a community or private Psychiatrist  • Community or private psychologist with skills in CBT for children and youth  British Columbia Psychological Association at 604 730-0522 (Lower Mainland) or toll free 1 800 730-0522 or the website http://www.psychologists.bc.ca/referral.html  • For treatment resistant cases (aged 6 to 19), refer to the tertiary care Mood and Anxiety Disorders Clinic at BC Children’s Hospital, 604 875-2010 or the website at http://www.bcchildrens.ca/Services/default.htm | British Columbia 2010(11) |  |  | Guiding statement (covered in other recommendations)  Strength/certainty of wording (“options”) |
| 126 | DEPR | If treatment with fluoxetine is unsuccessful or is not tolerated because of side effects, consideration should be given to the use of another antidepressant. In this case sertraline or citalopram are the  recommended second-line treatments.6 B | NICE 2005(27) |  |  | Strength/certainty of wording (“consideration”) |
| 127 | DEPR | Psychotherapy can be effective for treating depression, particularly in adolescents. If unavailable, medications may be indicated.  • The drug most often used is fluoxetine  • If bipolar vulnerability, start with a shorter-acting SSRI (e.g. sertraline)  • If comorbid anxiety, fluvoxamine or sertraline are possible alternatives  The majority of randomised control trials in depression show no significant benefit of SSRI medications over placebo. Spontaneous remission in community diagnosed adolescent depression is 50% within two months. However, those not remitting in this time period have a high risk of chronicity. Refer to a specialist. | British Columbia 2010(11) |  |  | Strength/certainty of wording (“can be”, “may be”) |
| 128 | DEPR | As with all other medications, consideration should be given to possible drug interactions when prescribing medication for depression in children and young people. This should include possible interactions with complementary and alternative medicines as well as with alcohol and ’recreational’ drugs. GPP | NICE 2005(27) |  |  | Strength/certainty of wording (“consideration”) |
| 129 | DEPR | A child or young person with depression who is taking St John's wort as an over-the-counter preparation should be informed of the risks and advised to discontinue treatment while being monitored for recurrence of depression and assessed for alternative treatments in accordance with this guideline. | NHMRC 2011(29) |  |  | Structure-level recommendation (not within audit sample or scope) |
| 130 | DEPR | In the absence of more substantial evidence on treating depression in young adults, it is reasonable to extrapolate from the evidence on psychological therapy in adolescents (see Recommendation 4, item directly above). | NHMRC 2011(29) |  |  | Guiding statement (covered in other recommendations) |
| 131 | DEPR | While CBT and IPT have high acceptability among young people with depression, consideration should be given to the young person’s suitability to undertake psychological therapy. | NHMRC 2011(29) |  |  | Strength/certainty of wording (“consideration”) |
| 132 | DEPR | CBT and IPT should be provided by professionally trained CBT/IPT therapists who have experience in working with young people. It is important that the therapy is applied in line with evidence-based practice manuals. Continuing maintenance of therapy skills is essential. | NHMRC 2011(29) |  |  | Structure-level recommendation (not within audit sample or scope) |
| 133 | DEPR | This guideline makes recommendations for the identification and treatment of depression in children (5–11 years) and young people (from the age of 12 up to their 18th birthday) in primary, community and secondary care. Depression is a broad and heterogeneous diagnostic grouping, central to which is depressed mood or loss of pleasure in most activities. Depressive symptoms are frequently accompanied by symptoms of anxiety, but may also occur on their own. The ICD-10 Classification of Mental and Behavioural Disorders  (World Health Organization, 1992) uses an agreed list of 10 depressive symptoms, and divides the common form of major depressive episode into four groups: not depressed (fewer than four symptoms), mild depression (four symptoms), moderate depression (five to six symptoms), and severe depression (seven or more symptoms, with or without psychotic symptoms). Symptoms should be present for at least 2 weeks and every symptom should be present for most of the day.  For the purposes of this guideline, the treatment and management of  depression have been divided into the following descriptions as defined by  ICD-10:  • mild depression  • moderate and severe depression  • severe depression with psychotic symptoms. | NICE 2005(27) |  |  | Guiding statement (covered in other recommendations) |
| 134 | DEPR | 1.4 Step 2: Recognition  CAMHS professionals need to improve their ability to recognise depression. | NICE 2005(27) |  |  | Structure-level recommendation (not within audit sample or scope) |
| 135 | DEPR | Children and young people of 11 years or older referred to CAMHS without a diagnosis of depression should be routinely screened with a self-report questionnaire for depression (of which the Mood and Feelings Questionnaire [MFQ] is currently the best) as part of a general assessment procedure. | NICE 2005(27) | B |  | Structure-level recommendation (not within audit sample or scope) |
| 136 | DEPR | Training opportunities should be made available to improve the accuracy of CAMHS professionals in diagnosing depressive conditions. The existing interviewer-based instruments (such as Kiddie-Sads [K-SADS] and Child and Adolescent Psychiatric Assessment [CAPA]) could be used for this purpose but will require modification for regular use in busy routine CAMHS settings. | NICE 2005(27) | C |  | Structure-level recommendation (not within audit sample or scope) |
| 137 | DEPR | However, it is doubtful whether the severity of the depressive illness can realistically be captured in a single symptom count. Clinicians will wish to consider family context and previous history, as well as the degree of associated impairment, in making this assessment | NICE 2005(27) |  |  | Guiding statement (covered in other recommendations) |
| 138 | DEPR | A multidisciplinary team approach is likely to have advantages for individuals with complex presentations. | NHMRC 2011(29) |  |  | Guiding statement (covered in other recommendations) |
| 139 | DEPR | The length of treatment required for effective remission varies. Depressive conditions may require up to 36 weeks of active treatment. | NHMRC 2011(29) |  |  | Guiding statement (covered in other recommendations) |
| 140 | DEPR | Referral to local mental health services for further assessment is generally required. | RCH paed handbook 2009(9) |  |  | Guiding statement (covered in other recommendations) |
| 141 | DEPR | GPs can play an important role in providing ongoing support and counselling to the child and/or family | RCH paed handbook 2009(9) |  |  | Guiding statement (covered in other recommendations) |
| 142 | DEPR | Antidepressants (generally SSRIs) can be used for non-rapid cycling bipolar disorder, psychotic depression with severe symptoms that prevents effective psychotherapy and poor response to adequate psychotherapy | RCH paed handbook 2009(9) |  |  | Strength/certainty of wording (“can be”) |
| 143 | DEPR | Attention should be paid to the possible need for parents’ own psychiatric problems (particularly depression) to be treated in parallel, if the child or young person’s mental health is to improve. If such a need is identified, then a plan for obtaining such treatment should be made, bearing in mind the availability of adult mental health provision and other services | NICE 2005(27) |  |  | Guiding statement (covered in other recommendations) |
| 144 | DEPR | Make a diagnosis if possible, and then begin to treat or refer as appropriate.  • Refer to Treatment Algorithm in this guideline  • Refer to Appendix A: Diagnosis of Anxiety Disorders in Children and Youth and Appendix B: Treatment of Anxiety Disorders and Depression in Children and Youth  • Provide parents with A Guide for Parents (either for Anxiety or Depression) and the Resource List for Families included with this guideline which lists pamphlets, books, web-resources and information | British Columbia 2010(11) |  |  | Guiding statement (covered in other recommendations) |
| 145 | DEPR | 1.1.2 Language and ethnic minorities  Information should be provided in a language and format that a child or young person and their family or carer(s) can properly understand; interpreters should be engaged when needed. Psychological treatments are also best conducted in the child or young person’s first language. Healthcare professionals should be trained to understand the specific needs of depressed children or young people from black and minority ethnic groups. Patients, families and carers, including those from black and minority ethnic groups, should be involved in planning services. | NICE 2005(27) |  |  | Guiding statement (covered in other recommendations) |
| 146 | DEPR | Where possible, all services should provide written information or audiotaped material in the language of the child or young person and their family or carer(s), and professional interpreters should be sought for those whose preferred language is not English. | NICE 2005(27) |  |  | Guiding statement (covered in other recommendations) |
| 147 | DEPR | Consideration should be given to providing psychological therapies and information about medication and local services in the language of the child or young person and their family or carers where the patient’s and/or their family’s or carer’s first language is not English. If this is not possible, an interpreter should be sought. | NICE 2005(27) |  |  | Strength/certainty of wording (“consideration”) |
| 148 | DEPR | Healthcare professionals in primary, secondary and relevant community settings should be trained in cultural competence to aid in the diagnosis and treatment of depression in children and young people from black and minority ethnic groups. This training should\ take into consideration the impact of the patient’s and healthcare professional’s racial identity status on the patient’s depression | NICE 2005(27) |  |  | Structure-level recommendation (not within audit sample or scope) |
| 149 | DEPR | Healthcare professionals working with interpreters should be provided with joint training opportunities with those interpreters, to ensure that both healthcare professionals and interpreters understand the specific requirements of interpretation in the mental health setting. | NICE 2005(27) |  |  | Structure-level recommendation (not within audit sample or scope) |
| 150 | DEPR | The development and evaluation of services for children and young  people with depression should be undertaken in collaboration with  stakeholders involving patients and their families and carers, including  members of black and minority ethnic groups | NICE 2005(27) |  |  | Structure-level recommendation (not within audit sample or scope) |
| 151 | DEPR | When a child or young person has been diagnosed with depression, consideration should be given to the possibility of parental depression, parental substance misuse, or other mental health problems and associated problems of living, as these are often associated with depression in a child or young person and, if untreated, may have a negative impact on the success of treatment offered to the child or young person. | NICE 2005(27) |  |  | Strength/certainty of wording (“consideration”) |
| 152 | DEPR | When the clinical progress of children and young people with depression is being monitored in secondary care, the self-report Mood and Feelings Questionnaire (MFQ) should be considered as an adjunct to clinical judgement. | NICE 2005(27) | C |  | Strength/certainty of wording (“should be considered”) |
| 153 | DEPR | The form of assessment should take account of cultural and ethnic variations in communication, family values and the place of the child or young person within the family. | NICE 2005(27) |  |  | Guiding statement (covered in other recommendations) |
| 154 | DEPR | 1.1.4 The organisation and planning of services  Better links between Child and Adolescent Mental Health Services (CAMHS) and tier 1 and tier 2 are needed to improve detection and availability of treatment (see glossary for explanations of tiers). All healthcare professionals should monitor detection rates and record outcomes for local planning and local, regional and national comparison. | NICE 2005(27) |  |  | Structure-level recommendation (not within audit sample or scope) |
| 155 | DEPR | Healthcare professionals specialising in depression in children and young people should work with local CAMHS to enhance specialist knowledge and skills regarding depression in these existing services.  This work should include providing training and help with guideline implementation. | NICE 2005(27) |  |  | Structure-level recommendation (not within audit sample or scope) |
| 156 | DEPR | CAMHS and primary care trusts (PCTs) should consider introducing a primary mental health worker (or CAMHS link worker) (see glossary) into each secondary school and secondary pupil referral unit as part of tier 2 provision within the locality. | NICE 2005(27) |  |  | Structure-level recommendation (not within audit sample or scope) |
| 157 | DEPR | Primary mental health workers (or CAMHS link workers) should establish clear lines of communication between CAMHS and tier 1 or 2, with named contact people in each tier or service, and develop systems for the collaborative planning of services for young people with depression in tiers 1 and 2. | NICE 2005(27) |  |  | Structure-level recommendation (not within audit sample or scope) |
| 158 | DEPR | CAMHS and PCTs should routinely monitor the rates of detection,  referral and treatment of children and young people, from all ethnic groups, with mental health problems, including those with depression, in local schools and primary care. This information should be used for planning services and made available for local, regional and national comparison. | NICE 2005(27) |  |  | Structure-level recommendation (not within audit sample or scope) |
| 159 | DEPR | Treatment considerations in all settings  Most treatment should be undertaken in outpatient settings or the community.  Before treatment is started the social networks around the child or young person need to be clearly identified. If bullying is a factor, school and healthcare professionals should jointly develop anti-bullying strategies. Psychological treatments should be provided by professionally trained therapists, who should aim to quickly develop an alliance with the child or young person and their family or carer(s). Comorbid conditions will also need to be treated and interventions considered for parents with depression or other significant personal problems. Advice about exercise, sleep and nutrition should also be considered. | NICE 2005(27) |  |  | Structure-level recommendation (not within audit sample or scope) |
| 160 | DEPR | Most children and young people with depression should be treated on an outpatient or community basis. | NICE 2005(27) | C |  | Guiding statement (“most”) |
| 161 | DEPR | When bullying is considered to be a factor in a child or young person’s depression, CAMHS, primary care and educational professionals should work collaboratively to prevent bullying and to develop effective anti-bullying strategies. | NICE 2005(27) | C |  | Structure-level recommendation (not within audit sample or scope) |
| 162 | DEPR | Psychological therapies used in the treatment of children and young people with depression should be provided by therapists who are also trained child and adolescent mental healthcare professionals. | NICE 2005(27) | B |  | Structure-level recommendation (not within audit sample or scope) |
| 163 | DEPR | Attention should be paid to the possible need for parents’ own psychiatric problems (particularly depression) to be treated in parallel, if the child or young person’s mental health is to improve. If such a need is identified, then a plan for obtaining such treatment should be made, bearing in mind the availability of adult mental health provision and other services. | NICE 2005(27) | B |  | Structure-level recommendation (not within audit sample or scope) |
| 164 | DEPR | 1.2 Stepped care  The stepped-care model of depression draws attention to the different needs that depressed children and young people have – depending on the characteristics of their depression and their personal and social circumstances – and the responses that are required from services. It provides a framework in which to organise the provision of services that support both healthcare professionals and patients and their parent(s) or carer(s) in identifying and accessing the most effective interventions (see Table 1).  Table 1 The stepped-care model.  Focus Action Responsibility  Detection Risk profiling Tier 1  Recognition Identification in presenting children or young  people  Tiers 2–4  Mild depression (including  dysthymia)  Watchful waiting  Non-directive supportive therapy/group  cognitive behavioural therapy/guided self-help  Tier 1  Tier 1 or 2  Moderate to severe  depression  Brief psychological therapy  +/– fluoxetine  Tier 2 or 3  Depression unresponsive  to treatment/recurrent  depression/psychotic  depression  Intensive psychological therapy  +/– fluoxetine, sertraline, citalopram,  augmentation with an antipsychotic  Tier 3 or 4  The guidance follows these five steps.  1. Detection and recognition of depression and risk profiling in primary care and community settings.  2. Recognition of depression in children and young people referred to  CAMHS.  3. Managing recognised depression in primary care and community settings – mild depression.  4. Managing recognised depression in tier 2 or 3 CAMHS – moderate to severe depression.  5. Managing recognised depression in tier 3 or 4 CAMHS – unresponsive, recurrent and psychotic depression, including depression needing inpatient care.  Each step introduces additional interventions; the higher steps assume interventions in the previous step. | NICE 2005(27) |  |  | Guiding statement (covered in other recommendations) |
| 165 | DEPR | 1.3 Step 1: Detection, risk profiling and referral  Healthcare professionals working with children or young people in primary care, schools and the community need training to assess the risk of depression, to provide emotional support and know when to refer, especially when a child or young person has experienced an undesirable life event.  CAMHS tier 2 or 3 should work with tier 1 healthcare professionals and help provide training in the recognition of depression. | NICE 2005(27) |  |  | Structure-level recommendation (not within audit sample or scope) |
| 166 | DEPR | Healthcare professionals in primary care, schools and other relevant community settings should be trained to detect symptoms of depression, and to assess children and young people who may be at risk of depression. Training should include the evaluation of recent  and past psychosocial risk factors, such as age, gender, family  discord, bullying, physical, sexual or emotional abuse, comorbid  disorders, including drug and alcohol use, and a history of parental  depression; the natural history of single loss events; the importance of multiple risk factors; ethnic and cultural factors; and factors known to be associated with a high risk of depression and other health problems, such as homelessness, refugee status and living in institutional settings. | NICE 2005(27) | C |  | Structure-level recommendation (not within audit sample or scope) |
| 167 | DEPR | Healthcare professionals in primary care, schools and other relevant community settings should be trained in communications skills such as ‘active listening’ and ‘conversational technique’, so that they can deal confidently with the acute sadness and distress (‘situational dysphoria’) that may be encountered in children and young people following recent undesirable events. | NICE 2005(27) |  |  | Structure-level recommendation (not within audit sample or scope) |
| 168 | DEPR | Healthcare professionals in primary care settings should be familiar with screening for mood disorders. They should have regular access to specialist supervision and consultation. | NICE 2005(27) |  |  | Structure-level recommendation (not within audit sample or scope) |
| 169 | DEPR | Healthcare professionals in primary care, schools and other relevant community settings who are providing support for a child or young person with situational dysphoria should consider ongoing social and environmental factors if the dysphoria becomes more persistent. | NICE 2005(27) |  |  | Structure-level recommendation (not within audit sample or scope) |
| 170 | DEPR | Child and Adolescent Mental Health Services (CAMHS) tier 2 or 3 should work with health and social care professionals in primary care, schools and other relevant community settings to provide training and develop ethnically and culturally sensitive systems for detecting, assessing, supporting and referring children and young people who are either depressed or at significant risk of becoming depressed. | NICE 2005(27) |  |  | Structure-level recommendation (not within audit sample or scope) |
| 171 | DEPR | In the provision of training by CAMHS professionals for healthcare professionals in primary care, schools and relevant community settings, priority should be given to the training of pastoral support staff in schools (particularly secondary schools), community paediatricians and GPs. | NICE 2005(27) |  |  | Structure-level recommendation (not within audit sample or scope) |
| 172 | DEPR | Within tier 3 CAMHS, professionals who specialise in the treatment of depression should have been trained in interviewer-based assessment instruments (such as K-SADS and CAPA) and have skills in non-verbal assessments of mood in younger children. | NICE 2005(27) |  |  | Structure-level recommendation (not within audit sample or scope) |
| 173 | DEPR | 1.6 Steps 4 and 5: Moderate to severe depression  There is little research evidence on the effectiveness of treatments for the younger child (5–11 years) with moderate to severe depression. In particular, there is little evidence for the effectiveness of antidepressant medication in children, which should, therefore, only be used very cautiously in this age group. In other respects, the recommended treatments for children are based upon the evidence for effectiveness in young people (12–18 years). In children and young people psychological therapies are the first-line treatments. | NICE 2005(27) |  |  | Guiding statement (covered in other recommendations) |
| 174 | DEPR | In severe depression, SSRIs such as fluoxetine may be helpful on a case-by-case basis with careful monitoring. | RCH paed handbook 2009(9) |  |  | Strength/certainty of wording (“may be helpful”) |
| 175 | DEPR | Benzodiazepine have no proven role in anxiety or depressive disorders in children, and may produce paradoxical agitation. | RCH paed handbook 2009(9) |  |  | Guiding statement (covered in other recommendations) |
| 176 | DEPR | Prescribing antidepressants for children and young people  At the date of publication (September 2005), there are no antidepressant drugs with a current UK Marketing Authorisation for depression in children and young people (under 18 years).2 However, in 2000, the Royal College of Paediatrics and Child Health issued a policy statement on the use of unlicensed medicines, or the use of licensed medicines for unlicensed applications, in children and young people. This states that such use is necessary in paediatric practice and that doctors are legally allowed to prescribe unlicensed medicines where there are no suitable alternatives and where the use is justified by a responsible body of professional opinion.  In December 2003, following a review by an Expert Working Group of the Committee on Safety of Medicines (CSM), the CSM advised that, despite the lack of a marketing authorisation for fluoxetine in the treatment of major depressive disorder in under 18s at that time, the balance of risks and benefits for this drug was favourable. The CSM also stated that sertraline, citalopram and escitalopram, paroxetine, venlafaxine and fluvoxamine should not be used as new therapy.4 However, its advice was clear that child and adolescent psychiatrists are able to prescribe selective serotonin reuptake inhibitors (SSRIs) other than fluoxetine in certain circumstances; for example, where drug treatment is indicated but a patient is intolerant of fluoxetine. In April 2005 the Committee on Human Medicinal Products (CHMP) of the  European Medicines Evaluation Agency (EMEA) also issued advice on the paediatric use of SSRIs and serotonin noradrenaline reuptake inhibitors (SNRIs). This advice referred to all uses of these drugs in paediatrics, not just the treatment of depression. The CHMP advised that these products should not be used in children and adolescents except within their approved indications – not usually depression – because of the risk of suicide-related behaviour and hostility. However, like the CSM, the CHMP also made it clear that doctors may make decisions based on the individual clinical needs of a child or an adolescent to use these products for the treatment of depression or anxiety. In such circumstances the CHMP recommended that patients be  monitored carefully for the appearance of suicidal behaviour, self-harm or  hostility, particularly at the beginning of treatment. | NICE 2005(27) |  |  | Guiding statement (covered in other recommendations) |
| 177 | DEPR | Pharmacological Management Strategies  In general, pharmacotherapy alone is not recommended for children and adolescents. Its use should ideally be preceded and complemented by psychotherapy and/or behavioural therapy. Employ pharmacological management strategies if non-pharmacological interventions are not achieving therapeutic goals. If required, these are the issues to be considered.  There is very little peer reviewed evidence as to the safety or efficacy of SSRI medications for the treatment of anxiety and/or depression in young children. Approximately two-thirds of randomized placebo controlled pharmacological trials for depressive disorders in children and adolescents consider an age range starting from ages 6-8 through to ages 17 or 18, while the other third of cases consider ages 12-13 through 17-18.  Given the above, no SSRIs are approved for marketing in Canada as appropriate medications for patients under age 18. Refer to the Health Canada statement below.  “It is important to note that Health Canada has not approved these drugs for use in patients under 18 years. The prescribing of drugs is a physician’s responsibility. Although these drugs are not authorized for use in children, doctors rely on their knowledge of patients and the drugs to determine whether to prescribe them at their discretion in a practice called off-label use. Off-label use of these drugs in children is acknowledged to be an important tool for doctors. Doctors are advised to carefully monitor patients of all ages for emotional or behavioural changes that may indicate potential for harm, including suicidal thoughts and the onset or worsening of agitation-type adverse events.”  Adding pharmacotherapy to the non-pharmacological approaches needs to be done with careful monitoring, while informing patient and family about risks and benefit. An emergency safety plan should be made when there is moderate to severe symptoms, whether or not pharmacotherapy is used.  Indications for pharmacotherapy include: persistent depression and/or a comorbid anxiety disorder which have not responded to psychosocial interventions. | British Columbia 2010(11) |  |  | Guiding statement (covered in other recommendations) |
| 178 | DEPR | A number of studies have indicated that SSRIs are prescribed for a variety of childhood problems including anxiety disorders, major depressive episodes, ADHD and other disorders. Prescribing rates are increasing in Australia, USA & Europe. | RCH paed handbook 2009(9) |  |  | Guiding statement (covered in other recommendations) |
| 179 | DEPR | There has been some concern regarding increased risk of suicidality and deliberate self-harm associated with SSRI use in children, although the evidence to date is relatively weak. Risk of deliberate self-harm appears to be highest in the first 2-3 weeks of starting an SSRI. | RCH paed handbook 2009(9) |  |  | Guiding statement (covered in other recommendations) |
| 180 | DEPR | 1.6.6 Inpatient care  Inpatient treatment for children and young people with depression should only be considered when the patient is at significant risk of self-harm and/or needs intensive treatment or supervision not available elsewhere. The following guidance outlines the use of inpatient facilities. | NICE 2005(27) |  |  | Strength/certainty of wording (“considered”) |
| 181 | DEPR | Inpatient treatment should be considered for children and young people who present with a high risk of suicide, high risk of serious self-harm or high risk of self-neglect, and/or when the intensity of treatment (or supervision) needed is not available elsewhere, or when intensive assessment is indicated. | NICE 2005(27) | C |  | Strength/certainty of wording (“considered”) |
| 182 | DEPR | When considering admission for a child or young person with depression, the benefits of inpatient treatment need to be balanced against potential detrimental effects, for example loss of family and community support. | NICE 2005(27) | C |  | Strength/certainty of wording (“considering”, “need to be balanced”) |
| 183 | DEPR | When inpatient treatment is indicated, CAMHS professionals should involve the child or young person and their parent(s) or carer(s) in the admission and treatment process whenever possible. | NICE 2005(27) | B |  | Strength/certainty of wording (“wherever possible”) |
| 184 | DEPR | Commissioners and strategic health authorities should ensure that inpatient treatment is available within reasonable travelling distance to enable the involvement of families and maintain social links. | NICE 2005(27) | B |  | Structure-level recommendation (not within audit sample or scope) |
| 185 | DEPR | Commissioners and strategic health authorities should ensure that inpatient services are able to admit a young person within an appropriate timescale, including immediate admission if necessary. | NICE 2005(27) |  |  | Structure-level recommendation (not within audit sample or scope) |
| 186 | DEPR | Inpatient services should have a range of interventions available including medication, individual and group psychological therapies and family support. | NICE 2005(27) | C |  | Structure-level recommendation (not within audit sample or scope) |
| 187 | DEPR | Inpatient facilities should be age appropriate and culturally enriching, with the capacity to provide appropriate educational and recreational activities. | NICE 2005(27) | C |  | Structure-level recommendation (not within audit sample or scope) |
| 188 | DEPR | Planning for aftercare arrangements should take place before admission or as early as possible after admission and should be based on the Care Programme Approach. | NICE 2005(27) |  |  | Structure-level recommendation (not within audit sample or scope) |
| 189 | DEPR | Tier 4 CAMHS professionals involved in assessing children or young people for possible inpatient admission should be specifically trained in issues of consent and capacity, the use of current mental health legislation and the use of childcare laws, as they apply to this group of patients. | NICE 2005(27) |  |  | Structure-level recommendation (not within audit sample or scope) |
| 190 | DEPR | CAMHS should keep primary care professionals up to date about progress and the need for monitoring of the child or young person in primary care. CAMHS should also inform relevant primary care professionals within 2 weeks of a patient being discharged and should provide advice about whom to contact in the event of a recurrence of depressive symptoms. | NICE 2005(27) |  |  | Structure-level recommendation (not within audit sample or scope) |
| 191 | DIAB | In young people with diabetes, psychological disorders are relatively common compared with prevalence rates for end-organ complications. | Aust Diabetes Society 2011(26) |  |  | Guiding statement (covered in other recommendations) |
| 192 | DIAB | Validated screening tools for psychological disorders in type 1 diabetes are available | Aust Diabetes Society 2011(26) |  |  | Guiding statement (covered in other recommendations) |
| 193 | DIAB | To minimise the impact of diabetes on cognitive function, every effort should be directed toward achieving glycaemic targets. | Aust Diabetes Society 2011(26) | Grade B |  | Guiding statement (covered in other recommendations) |
| 194 | DIAB | Real-time CGM could be considered for use by specialist units, in specific patient populations, such as those with hypoglycaemia unawareness, recurrent severe hypoglycaemia or suspected nocturnal hypoglycaemia. In these situations, use of a hypoglycaemia alarm in a real- time monitoring system may help to treat hypoglycaemia in a timely manner and prevent severe episodes of hypoglycaemia. | Aust Diabetes Society 2011(26) |  |  | Strength/certainty of wording (“could be considered”) |
| 195 | DIAB | When combined with CSII therapy, evidence from sensor-augmented CSII studies supports use of real-time CGM to improve HbA1c when they are used at least 70% of the time. | Aust Diabetes Society 2011(26) |  |  | Guiding statement |
| 196 | DIAB | Real-time CGM systems are not currently reimbursed by the NDSS or health insurance funds. Given current cost constraints, they are most likely to be useful over short periods of time, to aid profile setting and trouble shooting in glycaemic control. | Aust Diabetes Society 2011(26) |  |  | Guiding statement |
| 197 | DIAB | Retrospective CGM systems are not recommended for routine use to improve glycaemic control or reduce severe hypoglycaemia, but may be considered for children and adolescents. | Aust Diabetes Society 2011(26) | Grade C |  | Strength/certainty of wording (“may be considered”) |
| 198 | DIAB | Retrospective CGM systems could be considered for use by specialist units, in specific patient populations such as those with suspected nocturnal hypoglycaemia. | Aust Diabetes Society 2011(26) |  |  | Strength/certainty of wording (“could be considered”) |
| 199 | DIAB | Retrospective CGM systems are not currently reimbursed by the NDSS or health insurance funds. These systems are designed to be used continuously over short periods of time (e.g. up to 6 days continuously), to aid profile setting and trouble shooting in glycaemic control. | Aust Diabetes Society 2011(26) |  |  | Guiding statement |
| 200 | DIAB | Human insulin or insulin analogues may be used as treatment for glycaemic control. | Aust Diabetes Society 2011(26) | Grade C |  | Strength/certainty of wording (“may be used”) |
| 201 | DIAB | Basal and rapid-acting insulin analogues may reduce the risk of hypoglycaemia compared to human insulin | Aust Diabetes Society 2011(26) |  |  | Guiding statement |
| 202 | DIAB | Insulin analogues may be useful in people who have a history of recurrent nocturnal or severe hypoglycaemia. | Aust Diabetes Society 2011(26) |  |  | Guiding statement |
| 203 | DIAB | In some people, basal and rapid-acting insulin analogues may improve an individual’s HbA1c level without increasing hypoglycaemia. | Aust Diabetes Society 2011(26) |  |  | Guiding statement |
| 204 | DIAB | Rapid-acting insulin analogues may be useful in people who match bolus insulin doses to carbohydrate intake. | Aust Diabetes Society 2011(26) |  |  | Guiding statement |
| 205 | DIAB | Personal preference and quality of life should be considered when individualising insulin therapy, including analogue therapy versus human insulin. | Aust Diabetes Society 2011(26) |  |  | Guiding statement |
| 206 | DIAB | Nonsensor-augmented Continuous Subcutaneous Insulin Infusion (CSII) should be considered for use in individuals in whom the expected magnitude of benefit is clinically significant in terms of reducing HbA1c, reducing hypoglycaemia or improving QoL. | Aust Diabetes Society 2011(26) | Grade C |  | Strength/certainty of wording (“should be considered”) |
| 207 | DIAB | Individuals who may be likely to benefit from CSII pump therapy, as part of intensive diabetes management, are:  • some children and adolescents, including infants and young children, and pregnant women (ideally  preconception)  • individuals with microvascular complications of diabetes  • individuals with reduced hypoglycaemia awareness  • individuals (or their supervising adults) with desirable motivational factors; for example, those seeking to  improve blood glucose control and having realistic expectations  • individuals exhibiting desirable CSII treatment-related behavioural factors, including those who:  - are able to perform carbohydrate counting  - are currently undertaking four or more blood glucose tests per day  - have reliable adult supervision (in paediatrics), and a history of good self-management skills (in adults)  - are able to master the technical skills of CSII  - are reliable in follow-up health care. | Aust Diabetes Society 2011(26) |  |  | Guiding statement.  Strength/certainty of wording (“may be likely to benefit”) |
| 208 | DIAB | Metformin may be considered in individuals who have a high insulin requirement (e.g. overweight or obese subjects with total daily insulin dose at or above 2.0 IU/kg body weight), although the evidence demonstrates only a modest overall reduction in insulin requirement. | Aust Diabetes Society 2011(26) |  |  | Strength/certainty of wording (“may be considered”) |
| 209 | DIAB | Since metformin may contribute to lactic acidosis development in metabolically unstable patients, it is relatively contraindicated in people who are at high risk of developing diabetic ketoacidosis or have high alcohol consumption. | Aust Diabetes Society 2011(26) |  |  | Guiding statement. |
| 210 | DIAB | Technological mechanisms to support management can be a component of care for rural and remote patients, but should not replace face-to-face clinical care. | Aust Diabetes Society 2011(26) |  |  | Guiding statement. |
| 211 | DIAB | Consideration should be given to the practicality of using specific psychological screening tools in clinical practice (self versus interviewer or clinician administered; length; complexity), reference to more general tools or screening already undertaken, resourcing issues and labelling (as per mental health in general). | Aust Diabetes Society 2011(26) |  |  | Guiding statement. |
| 212 | DIAB | Diabetes care teams should have appropriate access to mental health professionals to support them in the assessment of psychological functioning in people with type 1 diabetes. | Aust Diabetes Society 2011(26) |  |  | Structure-level recommendation (not within audit sample or scope) |
| 213 | DIAB | In children and adolescents with type 1 diabetes, assessment of developmental progress in all domains (i.e. physical, intellectual, academic, emotional and social development) is advised on a routine basis. | Aust Diabetes Society 2011(26) |  |  | Strength/certainty of wording (“is advised”) |
| 214 | DIAB | Education and psychological support are an essential component of standard diabetes care. Intensified education and psychological support programs should be considered when treatment goals are not being met. | Aust Diabetes Society 2011(26) | Grade B |  | Strength/certainty of wording (“should be considered”)  (covered in other recommendations) |
| 215 | DIAB | The multidisciplinary diabetes health-care team should aim to maintain consistent contact with people with diabetes and their families or carers. | Aust Diabetes Society 2011(26) |  |  | Strength/certainty of wording (“should aim”) |
| 216 | DIAB | It is important for the multidisciplinary diabetes team to provide preventive interventions for patients and families (include training parents in effective behaviour-management skills) at key developmental stages, including after diagnosis and before adolescence. The aim of these interventions is to emphasise appropriate family involvement and support in diabetes management, effective problem-solving and self-management skills, and realistic expectations about glycaemic control (Delamater 2009). | Aust Diabetes Society 2011(26) |  |  | Recommendation not measurable. |
| 217 | DIAB | Diabetes care teams should have appropriate access to mental health professionals to support them in the delivery of psychological support. | Aust Diabetes Society 2011(26) |  |  | Structure-level recommendation (not within audit sample or scope) |
| 218 | DIAB | Flexible intensive insulin therapy programs, such as DAFNE, aim to provide dietary freedom for people with type 1 diabetes. | Aust Diabetes Society 2011(26) |  |  | Strength/certainty of wording (“aim”) |
| 219 | DIAB | Matching of mealtime insulin dose to carbohydrate intake should be considered for patients using multiple daily injection therapy. | Aust Diabetes Society 2011(26) | Grade C |  | Strength/certainty of wording (“should be considered”) |
| 220 | DIAB | An individualised insulin to carbohydrate ratio should be used for individuals using CSII and may be used in those on multiple daily injection therapy as part of a comprehensive education program. | Aust Diabetes Society 2011(26) |  |  | Strength/certainty of wording (“may be used”) |
| 221 | DIAB | Adjusting insulin with carbohydrate quantity has the potential to improve QoL and increase flexibility in food intake in people with type 1 diabetes. However, regularity in meal routines remains important for optimal glycaemic control | Aust Diabetes Society 2011(26) |  |  | Guiding statement (covered in other recommendation) |
| 222 | DIAB | Advice on carbohydrate quantity and distribution should take into account an individual's energy requirements, previous dietary and eating patterns, activity levels and insulin regimen. | Aust Diabetes Society 2011(26) |  |  | Guiding statement (covered in other recommendation) |
| 223 | DIAB | Advice on carbohydrate quantification should be given within the context of a healthy diet to ensure that overall nutritional needs are being met. In contrast, focussing solely on carbohydrate can lead to poor food choices, carbohydrate avoidance and/or insulin omission. | Aust Diabetes Society 2011(26) |  |  | Guiding statement (covered in other recommendation) |
| 224 | DIAB | Day-to-day consistency in carbohydrate intake is important for patients who are on fixed insulin regimens. | Aust Diabetes Society 2011(26) |  |  | Guiding statement (covered in other recommendation) |
| 225 | DIAB | GI should not be used in isolation, but with a method of carbohydrate quantification or regulation. | Aust Diabetes Society 2011(26) |  |  | Guiding statement (covered in other recommendation) |
| 226 | DIAB | High GI food choices combined with low GI food choices can lower the glycaemic impact of a meal. | Aust Diabetes Society 2011(26) |  |  | Guiding statement (covered in other recommendation) |
| 227 | DIAB | Where possible, high GI food choices should be substituted with moderate or low GI choices. | Aust Diabetes Society 2011(26) |  |  | Guiding statement (covered in other recommendation) |
| 228 | DIAB | Food choices should also consider other nutritional aspects in addition to GI, with a focus on lower fat, higher fibre, nutrient-dense foods. | Aust Diabetes Society 2011(26) |  |  | Guiding statement (covered in other recommendation) |
| 229 | DIAB | High-protein diets, particularly those based on animal protein or red meat, may lead to progression of diabetic nephropathy. Reducing protein intake to levels consistent with the recommended dietary intakes or replacing red meat with vegetable or soy protein may help to reduce the progression of nephropathy. | Aust Diabetes Society 2011(26) |  |  | Guiding statement (covered in other recommendation) |
| 230 | DIAB | Restricting carbohydrate intake may affect the nutritional adequacy of the diet and may cause hypoglycaemia if insulin therapy is not adjusted accordingly. | Aust Diabetes Society 2011(26) |  |  | Guiding statement (covered in other recommendation) |
| 231 | DIAB | High-protein, low carbohydrate diets result in ketosis, which may affect blood glucose control and result in dehydration, lethargy and loss of lean body mass. | Aust Diabetes Society 2011(26) |  |  | Guiding statement (covered in other recommendation) |
| 232 | DIAB | Advice on fat intake should include focussing on reducing saturated and trans fat intake, to reduce the risk of cardiovascular disease. | Aust Diabetes Society 2011(26) |  |  | Guiding statement (covered in other recommendation) |
| 233 | DIAB | Substitution of saturated and trans fats with monounsaturated or polyunsaturated fats should be encouraged. | Aust Diabetes Society 2011(26) |  |  | Guiding statement (covered in other recommendation) |
| 234 | DIAB | Education on carbohydrate quantification should discourage consumption of high-fat foods, particularly packaged snacks | Aust Diabetes Society 2011(26) |  |  | Guiding statement (covered in other recommendation) |
| 235 | DIAB | CAM should not be used in type 1 diabetes to target metabolic outcomes. |  | Grade C |  | Guiding statement.  Recommendation not measurable (not sampling CAM health practitioner records) |
| 236 | DIAB | Clinicians should ask people about CAM in a nonjudgmental way, and document their use. | Aust Diabetes Society 2011(26) |  |  | Guiding statement (covered in other recommendation) |
| 237 | DIAB | People with type 1 diabetes should be aware that there is a lack of evidence for the effectiveness of CAM | Aust Diabetes Society 2011(26) |  |  | Guiding statement |
| 238 | DIAB | While there is evidence for a low rate of adverse events due to CAM usage, the possibility of adverse interactions between CAM and conventional medicines should be considered | Aust Diabetes Society 2011(26) |  |  | Guiding statement |
| 239 | DIAB | Minimising severe hypoglycaemia is important, including as a component of intensive diabetes management | Aust Diabetes Society 2011(26) |  |  | Guiding statement |
| 240 | DIAB | Intensive diabetes management may increase the risk of severe hypoglycaemia; therefore, some people who have a high risk of severe hypoglycaemia may not be suitable for the usual tight HbA1c targets. | Aust Diabetes Society 2011(26) |  |  | Guiding statement |
| 241 | DIAB | Specific management strategies should be implemented for people who have a high risk of severe hypoglycaemia, including those with a history of severe hypoglycaemia or a reduced ability to detect early warning symptoms of hypoglycaemia (i.e. hypoglycaemia unawareness). In cases of hypoglycaemia unawareness, strategies to reduce severe hypoglycaemia include more frequent SMBG, and ensuring that any blood glucose below a certain threshold (e.g. <4 mmol/L) is treated as hypoglycaemia, even in the absence of hypoglycaemic symptoms | Aust Diabetes Society 2011(26) |  |  | Guiding statement |
| 242 | DIAB | A medical practitioner should carefully assess whether a person with type 1 diabetes is fit to drive a motor vehicle, this is required, in particular, to help reduce the risk of motor vehicle crashes due to severe hypoglycaemia. The AustRoads Assessing fitness to drive booklet, should be used as a reference. | Aust Diabetes Society 2011(26) |  |  | Outside of scope and sample of medical record audit (i.e. children aged < 16 years) |
| 243 | DIAB | Acute hypoglycaemia (Grade B) and hyperglycaemia (Grade C) should be minimised to maintain optimal cognitive performance. | Aust Diabetes Society 2011(26) | Grade B (hypoglycaemia); Grade C (hyperglycaemia) |  | Guiding statement |
| 244 | DIAB | Mild hypoglycaemia and mild hyperglycaemia are common in type 1 diabetes; however, acute severe dysregulation of blood glucose to either extreme that may cause cognitive effects should be avoidable in most people with type 1 diabetes, if due self-care is taken. | Aust Diabetes Society 2011(26) |  |  | Guiding statement |
| 245 | DIAB | Adverse cognitive effects of acute severe hypoglycaemia and acute severe hyperglycaemia should be avoided during tasks requiring high level cognitive function, such as in school, college or university examinations; or in adolescents and adults during potentially dangerous activities involving occupational health, such as operating heavy machinery or during driving. In some cases, the risk or presence of acute severe changes in blood glucose to very low and possibly very high levels may lead to the need for exemption from or avoidance of the cognitively demanding or high-risk activity. | Aust Diabetes Society 2011(26) |  |  | Guiding statement |
| 246 | DIAB | The blood glucose level at which a person develops cognitive effects from severe hypoglycaemia can vary, related to the degree of chronic glycaemia control and avoidance of severe hypoglycaemia if an episode has occurred during recent weeks to months. In such cases, early warning symptoms of hypoglycaemia that may have been lacking in a person with type 1 diabetes may at least partially return. | Aust Diabetes Society 2011(26) |  |  | Guiding statement |
| 247 | DIAB | Some programs, such as BGAT, can be delivered as individual or group programs. | Aust Diabetes Society 2011(26) |  |  | Guiding statement |
| 248 | DIAB | Where resource constraints apply, structured education should be offered preferentially to individuals at highest risk of and from severe hypoglycaemia, for example, those with a history of recurrent severe hypoglycaemia, and adults who are motor vehicle drivers. | Aust Diabetes Society 2011(26) |  |  | Guiding statement |
| 249 | DIAB | Research into modified programs to prevent severe hypoglycaemia that may require less resource and time input needs to be undertaken. Such research needs documented outcomes, including assessment of optimal time intervals for people to undertake refresher courses. | Aust Diabetes Society 2011(26) |  |  | Guiding statement |
| 250 | DIAB | Blood ketone measurement is preferred in people using CSII. | Aust Diabetes Society 2011(26) |  |  | Guiding statement |
| 251 | DIAB | Blood ketone measurement may be especially useful in very young children or when urine specimens are difficult to obtain | Aust Diabetes Society 2011(26) |  |  | Guiding statement |
| 252 | DIAB | The generalisability of implementing an intensive glycaemic control strategy may be limited by the strict inclusion criteria in the clinical trials undertaken. The potential benefit of a strategy of intensive glycaemic control needs to be individualised as much as is practical. | Aust Diabetes Society 2011(26) |  |  | Guiding statement |
| 253 | DIAB | For patients who are intolerant of ACEI, ARBs can be used as an alternative treatment for the secondary prevention of nephropathy. | Aust Diabetes Society 2011(26) |  |  | Strength/certainty of wording (“can be used”) |
| 254 | DIAB | On the basis of the systematic evidence, including data in adolescents (Cook et al 1990), ACEI in type 1 diabetes can control albuminuria in normotensive microalbuminuria; however, there are currently restrictions from the Therapeutic Goods Administration to be considered in their use in this setting of normotension. | Aust Diabetes Society 2011(26) |  |  | Guiding statement |
| 255 | DIAB | Children and adolescents with DKA should be managed in a unit that has:  Experienced nursing staff trained in the monitoring and management of DKA  A paediatric endocrinologist, paediatrician or paediatric critical care specialist with training and expertise in the management of DKA. Where such expertise is not available on-site, telephone advice should be sought from the appropriate specialists  Access to laboratories for frequent and timely evaluation of biochemical variables | Aust Diabetes Society 2011(26) |  |  | Structure-level recommendation (not within audit sample or scope) |
| 256 | DIAB | Supportive measures to consider if appropriate:  Secure the airway and consider ng tube placement (to avoid aspiration) in the unconscious / severely obtunded patient  Insert a second peripheral IV catheter for convenient and painless repetitive blood sampling  Give oxygen to patients with severe circulatory impairment or shock.  Use a cardiac monitor for continuous electrocardiographic monitoring to assess for signs of hyperkalaemia (peaked T-waves, widened QRS) or hypokalaemia (flattened or inverted T waves, ST depression, wide PR interval).  Consider antibiotics for febrile patients after obtaining appropriate cultures  Catheterise the bladder if the patient is unconscious or unable to void on demand to allow for strict fluid balance (e.g. in infants and very ill young children) | RCH 2013(30) |  |  | Strength/certainty of wording (“to consider if appropriate”) |
| 257 | DIAB | Fluid Requirements  Intravenous or oral fluids that may have been given at another facility should be factored into the assessment and calculation of fluid deficit and replacement needs | RCH 2013(30) |  |  | Recommendation not measurable (out of scope and sample of medical record audit) |
| 258 | DIAB | Patients with DKA rarely require >20 ml/kg in total as a bolus. The potential for harm with over-zealous fluid administration must be remembered. Please discuss the use of additional boluses (> 20ml/kg total) with the endocrinologist on call or local paediatrician | RCH 2013(30) |  |  | Guiding statement |
| 259 | DIAB | The insulin infusion may be run as a sideline with the rehydrating fluid via a three-way tap, provided a syringe pump is used. Ensure that the insulin is clearly labelled. | RCH 2013(30) |  |  | Structure-level recommendation (not within audit sample or scope) |
| 260 | DIAB | Beware the rare entity of hyperglycaemic-hyperosmolar non-ketotic coma. If this is a possibility, insulin should ONLY be used after discussion with local paediatric team and/or paediatric endocrinologist | RCH 2013(30) |  |  | Guiding statement |
| 261 | DIAB | Cerebral Oedema  Some degree of subclinical brain swelling is present during most episodes of diabetic ketoacidosis. Clinical cerebral oedema occurs suddenly, usually between 6 and 12 hours after starting therapy (range 2 - 24 hr). Mortality or severe morbidity is very high without early treatment. | RCH 2013(30) |  |  | Guiding statement |
| 262 | DIAB | Prevention  Slow correction of fluid and biochemical abnormalities. Optimally, the rate of fall of blood glucose and serum osmolality should not exceed 5 mmol/l/hr, but in children there is often a quicker initial fall in glucose. Patients should be nursed head up. | RCH 2013(30) |  |  | Guiding statement |
| 263 | DIAB | Warning signs  Risk factors: first presentation, long history of poor control, young age (< 5 yr)  No sodium rise as glucose falls, hyponatraemia during therapy, initial adjusted hypernatraemia  Headache, irritability, lethargy, depressed consciousness, incontinence, thermal instability.  Very late signs - bradycardia, increased BP and respiratory impairment. | RCH 2013(30) |  |  | Guiding statement |
| 264 | DIAB | Additional tests to consider  For children / adolescents who are overweight or have clinical evidence of acanthosis nigricans:  C-peptide and insulin levels (may help to distinguish Type 2 diabetes, although T1DM still more likely in this scenario)  lipid profile  LFTs | RCH 2013(30) |  |  | Strength/certainty of wording (“to consider”) |
| 265 | DIAB | Management  The decision about the individual insulin regimen will be made by the paediatric diabetes team in discussion with the family and child. The regimens outlined below are a guide only and individual clinicians may recommend an alternative approach. | RCH 2013(30) |  |  | Guiding statement |
| 266 | DIAB | Patients with established T1DM who present with hyperglycaemia and ketosis but normal pH, will need additional s.c. insulin to clear their ketones. | RCH 2013(30) |  |  | Guiding statement |
| 267 | DIAB | Diabetic educators and the endocrinology team are available for help with management. | RCH 2013(30) |  |  | Guiding statement |
| 268 | ECZE | Twice weekly maintenance therapy with a topical corticosteroid should be considered in patients with moderate to severe atopic eczema experiencing frequent relapses. | SIGN 2011(31) |  | Grade A | Strength/certainty of wording (“should be considered”) |
| 269 | ECZE | Topical tacrolimus should be considered, in patients aged two years and older, for short term, intermittent treatment of moderate to severe atopic eczema that has not been controlled by topical corticosteroids or where there is a serious risk of important adverse effects from further topical corticosteroid use, particularly skin atrophy. | SIGN 2011(31) |  | Grade C | Strength/certainty of wording (“should be considered”) |
| 270 | ECZE | There are 2 types of wet dressings that can be applied. Either can be recommended and cost the same amount.  - Tubifast. This is quicker and easier to apply  - Disposable towel and crepe bandage. This stays wetter longer. | RCH Melbourne 2013(32) |  |  | Guiding statement |
| 271 | ECZE | Wet dressings are usually ordered to be applied 2-3 times per day | The Children’s Hospital at Westmead 2012(32) |  |  | Guiding statement. |
| 272 | ECZE | The use of distraction techniques during the dressing help to make the process more relaxed. Playing children’s music, singing songs, playing games or watching TV while doing the dressing may make it a more pleasant time for everyone. | The Children’s Hospital at Westmead 2012(32) |  |  | Guiding statement. |
| 273 | ECZE | Children may need distraction in between dressings if they become itchy. Distractions include those mentioned above plus playing, formal playgroup, walks and television. | The Children’s Hospital at Westmead 2012(32) |  |  | Guiding statement. |
| 274 | ECZE | Secondary bacterial infection of eczema is a common complication, it should be suspected if there is crusting, weeping, erythema, cracks, frank pus or multiple excoriations and increased itch suggest bacterial infection. The usual organism is Staphylococcus aureus. | RCH Melbourne 2013(32) |  |  | Guiding statement. |
| 275 | ECZE | Secondary herpes simplex 1 infection is characterised by a sudden onset of grouped, small white or clear fluid filled vesicles, satellite or "punch out" lesions, pustules, and erosions. It is often tender, painful and itchy. | RCH Melbourne 2013(32) |  |  | Guiding statement. |
| 276 | ECZE | The principles of managing infected eczema are:  - Removing the crusts (cool compressing or soaking in the bath) | RCH Melbourne 2013(32) |  |  | Guiding statement. |
| 277 | ECZE | The principles of managing infected eczema are:  Cortisone can be applied over open skin and presence of infection, however rove the crusts and weeping FIRSTLY. | RCH Melbourne 2013(32) |  |  | Guiding statement. |
| 278 | ECZE | Bacterial or viral swabs may be taken if the diagnosis needs to be verified. | RCH Melbourne 2013(32) |  |  | Strength/certainty of wording (“may be taken”) |
| 279 | FEVE | If infectious disease is suspected it is essential that infection control measures are implemented to prevent cross contamination and spread | NSW Health 2010(33) |  |  | Structure-level recommendation (not within audit sample or scope) |
| 280 | FEVE | Young infants usually present with non-specific symptoms and signs of illness, and localising signs of disease are often lacking. General aspects of the child's behaviour and appearance provide the best indication of whether a serious infection is likely | SA Child Health Clinical Network 2013(34) |  |  | Guiding statement |
| 281 | FEVE | Because bacteraemia can occur with focal infections, it is recommended that when a source of infection is identified on physical examination, further evaluation be considered if the doctor judges that focal findings are insufficient to explain the degree of the child's fever and illness. If the source of the fever is found, then appropriate management should be instituted | SA Child Health Clinical Network 2013(34) |  |  | Guiding statement |
| 282 | FEVE | Children at higher risk of SBI should usually have appropriate investigations performed according to their age and risk group - Children at higher risk of serious bacterial infection include:  - infants under 3 months of age with temperature ≥38°C;  - infants aged 3-6 months with temperature >39°C; and  - children aged 6-36 months who are not fully immunised or appear unwell  These groups should usually have investigations performed. | SA Child Health Clinical Network 2013(34)  NICE 2013(35) |  |  | Strength/certainty of wording (“usually”) |
| 283 | FEVE | Well appearing children 6-36 months with temperature > 39 degrees C may have a less invasive approach to management | SA Child Health Clinical Network 2013(34) |  |  | Guiding statement |
| 284 | FEVE | Referral for immediate investigation at an appropriate facility should be strongly considered in all infants less than 1 month of age with fever seen by any health care provider | SA Child Health Clinical Network 2013(34) |  |  | Strength/certainty of wording (“considered”) |
| 285 | FEVE | Antipyretics may be administered to a distressed child with fever - paracetamol 15mg/kg per dose given up to 4 hourly up to a maximum dose of four doses in 24 hours. Ibuprofen (not recommended for children less than 6 months of age) 10mg/kg per dose, given up to 6 hourly up to a maximum of four doses each 24 hours | NSW Health 2010(33) |  |  | Strength/certainty of wording (“may be”) |
| 286 | FEVE | It is advisable to speak with child’s usual paediatrician or specialist when they present with febrile illness. If usual doctor is unavailable, advised to speak with relevant specialist or on call paediatrician at WCH | SA Child Health Clinical Network 2013(34) |  |  | Guiding statement |
| 287 | GORD | Further investigations such as barium radiology, pH study, milk scan, impedance, oesophageal manometry, oesphagoscopy may be indicated where:  - there is a need to confirm the diagnosis  - faltering growth  - excessive vomiting  - features suggestive of oesophagitis  - abnormal electrolytes/acidosis  - unexplained or difficult to control respiratory disease | Bhavsar 2011(36) |  |  | Strength/certainty of wording (“may be indicated”) |
| 288 | GORD | Consider a pH study for acid reflux for children in whom:  - diagnosis is uncertain  - poor response to medical treatment  - surgery is considered  - doing the test will lead to a change in management  - symptoms suggesting occult reflux  - unexplained or difficult to control respiratory disease | Vandenplas 2009(37)  Bhavsar 2011(36) |  |  | Strength/certainty of wording (“consider”) |
| 289 | GORD | Oesophageal impedance is superior to pH monitoring in order to evaluate the temporal relationship between symptoms and GORD. | Vandenplas 2009(37)  Bhavsar 2011(36) |  |  | Guiding statement |
| 290 | GORD | In children with severe symptomatic reflux or suspected oesophagitis an upper gastrointestinal endoscopy should be considered | Bhavsar 2011(36) |  |  | Strength/certainty of wording (“considered”) |
| 291 | GORD | Infants with uncomplicated recurrent regurgitation who present with warning signs or the regurgitation is unresolved at 12-18mths of age should be considered for a oesophagogastroduodenoscopy and biopsy | Vandenplas 2009(37) |  |  | Strength/certainty of wording (“considered”) |
| 292 | GORD | Regurgitation in infants "happy spitter" treatment includes:  - if formula fed consider milk thickener (Soya formulae is NOT recommended for infants <6mths) | Qld Health 2011  RCH paed handbook 2009(9)  Vandenplas 2009(37)  Bhavsar 2011(36) |  |  | Strength/certainty of wording (“consider”) |
| 293 | GORD | Surgery may be indicated for children in whom:  - there is a failure of optimal medical therapy  - dependence on long term medical therapy  - extra oesophageal manifestations (asthma, cough, chest pain, recurrent pulmonary aspiration or refluxate)  - complications of GORD (Barrett’s oesophagus, peptic stricture) | Bhavsar 2011(36) |  |  | Strength/certainty of wording (“may be indicated”) |
| 294 | GORD | Anti-reflux surgery should be considered only in children with GERD and failure of optimized medical therapy, or long-term dependence on medical therapy where compliance or patient preference preclude ongoing use, or life-threatening complications. | Vandenplas 2009(37)  Allen 2012(38) |  |  | Strength/certainty of wording (“considered”) |
| 295 | HEAD | Severe Head Injury:  The initial aim of management of a child with a serious head injury is prevention of secondary brain damage. The key aims are to maintain oxygenation, ventilation and circulation, and to avoid rises in intracranial pressure (ICP).  Urgent CT of head and c-spine. Ensure early neurosurgical and ICU intervention.  Cervical spine immobilisation should be maintained even if cervical spine imaging is normal.  Intubation and ventilation:  Child unresponsive or not responding purposefully to pain  GCS persistently <8  Loss of protective laryngeal reflexes  Respiratory irregularity  In consultation with the neurosurgeon consider measures to decrease intracranial pressure:  Nurse 20-30 degrees head up (after correction of shock) and head in midline position to help venous drainage.  Ventilate to a pCO2 35mmHg 4-4.5 kPa (consider arterial catheter).  Ensure adequate blood pressure with crystalloid infusion or inotropes (e.g. noradrenaline) if necessary.  Consider mannitol (0.5-1 g/kg over 20-30 min i.v.) or hypertonic saline (NaCl 3% 3 ml/kg over 10-20 min i.v.).  Consider phenytoin loading dose (20 mg/kg over 20 min i.v.). | RCH Melb(39) |  |  | Guiding statement (covered in other recommendation) |
| 296 | HEAD | Refer  Any infant or young child with a suspicious head injury may have been physically assaulted and therefore should be referred immediately to a tertiary centre. Consultation with a tertiary centre is advisable in all cases where there is the slightest suspicion that an infant has been abused. | NSW Health 2010 |  |  | Guiding statement (covered in other recommendation) |
| 297 | HEAD | It takes mandatory reporters through a series of questions to assist practitioners to assess whether or not a case involves suspected risk of significant harm and if it does, a report must be made to the  Child Protection Helpline. In all other cases of suspected inflicted head injury, the Mandatory Reporter Guide will help inform the practitioner’s next steps, which might include making a report to a NSW Health Child Wellbeing Unit on 1300 480 420. In all suspected cases, if there are young siblings in the house they will need to be examined. | NSW Health 2010 |  |  | Guiding statement (covered in other recommendation) |
| 298 | HEAD | Become suspicious of inflicted head injury in situations where infants have: 1. Symptoms and signs of head injury  (a) vomiting, (b) decreased level of consciousness, (c) irritability, (d) lethargy, (e) apnoea, (f) seizures, (g) hypothermia, (h) bradycardia and,  (i) a bulging fontanelle. Symptoms which may suggest that a child has had a previous brain injury are (a) irritability and vomiting, (b) feeding difficulties, (c) lethargy and drowsiness, (d) a rising head circumference. | NSW Health 2010 |  |  | Guiding statement (covered in other recommendation) |
| 299 | HEAD | Relative indications for neuroimaging:  Loss of consciousness lasting more than 5 minutes (witnessed)  Amnesia (antegrade or retrograde) lasting more than 5 minutes  Persistent vomiting  Clinical suspicion of non-accidental injury  Post-traumatic seizures (except a brief (<2 min) convulsion occurring at time of the impact)  GCS persistently less than 14, or for a baby under 1 year GCS (paediatric) persistently less than 15, on assessment in the emergency department  If under 1 year, presence of bruise, swelling or laceration of more than 5 cm on the head  Dangerous mechanism of injury (high-speed road traffic accident either as pedestrian, cyclist or vehicle occupant, fall from a height).  Known bleeding tendency | RCH Melb(39) |  |  | Guiding statement (covered in other recommendation) |
| 300 | HEAD | CT scanning should be considered within eight hours if any of the following features are present (excluding indications for an immediate scan):  - presence of any bruise/swelling/laceration >5 cm on the head  - post-traumatic seizure, but no history of epilepsy nor history suggestive of  reflex anoxic seizure  - amnesia (anterograde or retrograde) lasting >5 minutes  - clinical suspicion of non-accidental head injury  - a significant fall  - age under one year: GCS<15 in emergency department assessed by  personnel experienced in paediatric GCS monitoring  - three or more discrete episodes of vomiting  - abnormal drowsiness (slowness to respond). | SIGN 2009 | Grade C |  | Strength/certainty of wording (“considered”) |
| 301 | HEAD | Consideration may be given to offering families access to social work or pastoral care support, particularly in case of severe injury (consistent with local policies and resources). | NSW Health 2011(40) |  |  | Strength/certainty of wording (“consideration”) |
| 302 | HEAD | Ongoing neuromuscular blockade after initial intubation may mask signs of deterioration such as fitting and should be used judiciously. | NSW Health 2011(40) |  |  | Guiding statement (covered in other recommendation) |
| 303 | HEAD | Cervical spine CT scans  Cervical spine injuries are less common in children than adults but consideration should be given to imaging the c-spine at  the same time as head CT in the following circumstances:  - Midline cervical spine tenderness  - Focal neurological signs or symptoms  - GCS less than or equal to 8  - Abnormal or suspicious plain films  - Strong suspicion of injury despite normal or inadequate plain films. | NSW Health 2011(40) |  |  | Guiding statement (covered in other recommendation) |
| 304 | HEAD | Diagnostic tests  CT scans in children  The most accurate and rapid means of detecting intracranial injury is with a CT scan. A CT scan is relatively safe, although some children (particularly between six months and four years of age) will require sedation or general anaesthesia for the procedure. Access to monitoring and resuscitation is essential during transfer and scanning. CT involves a larger dose of radiation than conventional x-ray therefore exposure should be minimised by utilising this imaging modality only when clinically indicated. A CT scan is used to identify intra-cerebral bleeding, cerebral oedema or diffuse brain injury but may be normal if performed early. Subdural haematoma classically manifests clinically sometime after the initial injury, often with subtle signs or symptoms: a repeat CT scan should be considered for persistent symptoms or evolving signs. Young children are particularly difficult to evaluate. Where there are ongoing clinical concerns, a repeat CT scan or consultation with a paediatric expert is essential. Given the issue of distance and dislocation for families if the child requires transfer to a larger centre the benefits of a scan have to be weighed against the risks of delay in diagnosis resulting from an "observation only" policy. All high risk patients who are unable have immediate CT scanning should be discussed with an expert regarding indication for transfer or observation in hospital for at least 24 hours. | NSW Health 2011(40) |  |  | Guiding statement (covered in other recommendation) |
| 305 | HEAD | Early referral to NETS (or a local retrieval service) is encouraged for clinical support or advice or to activate a medical retrieval. Any change in the condition of a patient should result in further consultation with the retrieval service, even after retrieval has been arranged, to discuss modification in management strategy if required. | NSW Health 2011(40) |  |  | Guiding statement (covered in other recommendation) |
| 306 | HEAD | Childhood injury can be devastating. Most head injuries are mild and have a good prognosis, but moderate to severe head injuries are associated with poorer outcomes. Parents should be provided with timely information on their child’s condition and be involved in the decision making process. Health professionals should make arrangements for the parent to be with the child at all times with the exception of urgent interventions. The child, especially if conscious, will be more co-operative and interpretation of clinical symptoms and signs will be more reliable in the presence of a parent | NSW Health 2011(40) |  |  | Guiding statement (covered in other recommendation) |
| 307 | HEAD | Role of skull X-ray  Skull X-rays have no role in the identification of intracranial injury as intracranial contents are not seen on plain films. Although the radiological presence of a skull fracture significantly increases the risk of intracranial injury paediatric skull X-rays are difficult to interpret. Skull X-rays may be interpreted as abnormal in children who do not have intracranial injury (e.g. due to suture lines) or may be normal where intracranial injury is present | NSW Health 2011(40) |  |  | Guiding statement (covered in other recommendation) |
| 308 | HEAD | When there is no local CT scanning facility available, there may be a limited role for skull X-ray to complement clinical assessment and observation in deciding which children with intermediate risk head injury should be transferred for CT. If used, findings should be interpreted with caution and considered in conjunction with clinical observation. All abnormal skull X-rays should be discussed with a paediatric expert. | NSW Health 2011(40) |  |  | Guiding statement (covered in other recommendation) |
| 309 | HEAD | Administration of hyperosmolar therapy may be considered only after discussion with paediatric referral centre. Options include mannitol 0.5g/kg IV (2.5mls/kg of 20% mannitol) over 20 minutes(12) or hypertonic saline 3%, 3mL/kg over 30 to 60 minutes or as directed by the referral centre. If hyperosmolar therapy is commenced an IDC must be inserted to accurately monitor urine output. | NSW Health 2011(40) |  |  | Strength/certainty of wording (“may be considered”) |
| 310 | HEAD | Children aged less than 1 year  Special care must be taken with infants under 1 year of age who require greater vigilance due to difficulty in clinical assessment and a greater risk of inflicted head injury. A high index of suspicion for intracranial injury must exist for these patients. If all risk factors except age are in the low risk category the child may be managed in the low risk group after consultation with paediatric experts. | NSW Health 2011(40) |  |  | Guiding statement (covered in other recommendation) |
| 311 | HEAD | Anti-emetics  Vomiting increases intra-cranial pressure and places children in cervical spine protection at risk of aspiration. Ondansetron 0.15mg/kg may be given IV or orally to children who are nauseous, vomiting or immobilised. It should be noted that episodes of vomiting separated by periods of recovery are more concerning than where emesis occurs shortly after injury. | NSW Health 2011(40) |  |  | Guiding statement (covered in other recommendation) |
| 312 | HEAD | Other adjuncts  - Oro-gastric tubes and urinary catheters are unpleasant and stressful in the conscious child. They should be inserted in patients who are intubated or considered in the presence of other usual clinical indicators. | NSW Health 2011(40) |  |  | Guiding statement (covered in other recommendation) |
| 313 | HEAD | Analgesia and sedation  These medications may be useful in maintaining the airway, during procedures and in the reduction of pain and stress which are associated with increases in cerebral metabolic rate. Many head injured patients will have concomitant injuries, and effective analgesia is essential. Paracetamol may be used as a simple analgesic. Narcotics are not contraindicated in head injury and should be carefully titrated to pain score using a validated pain assessment tool. More severe pain from other injuries may require a bolus of IV morphine (50-100microgram/kg) and may be followed by a morphine infusion  (20-40microgram/kg/hr) once adequate analgesia is achieved. Sedation may be considered as an adjunct for the treatment of painful and noxious stimuli and elevated ICP in the management of significant head injury. | NSW Health 2011(40) |  |  | Guiding statement (covered in other recommendation) |
| 314 | HEAD | When to consider transfer to a tertiary centre:  All severe head injuries  Deteriorating conscious level (especially motor response changes)  Focal neurological signs  Seizure without full recovery  Definite or suspected penetrating injury  Cerebrospinal fluid leak  Child requiring care beyond the comfort level of the hospital. | NSW Health 2011(40) |  |  | Strength/certainty of wording (“consider”) |
| 315 | HEAD | When to consider consultation with local paediatric or paediatric neurosurgical team:  Moderate head injury with  Ongoing drowsiness or vomiting  Unexplained confusion lasting for more than 4 hours | NSW Health 2011(40) |  |  | Strength/certainty of wording (“consider”) |
| 316 | HEAD | CT scanning should be considered within eight hours if any of the following features are present (excluding indications for an immediate scan):  - presence of any bruise/swelling/laceration >5 cm on the head  - post-traumatic seizure, but no history of epilepsy nor history suggestive of reflex anoxic seizure  - amnesia (anterograde or retrograde) lasting >5 minutes  - clinical suspicion of non-accidental head injury  - a significant fall  - age under one year: GCS<15 in emergency department assessed by  personnel experienced in paediatric GCS monitoring  - three or more discrete episodes of vomiting  - abnormal drowsiness (slowness to respond). | SIGN 2009 | Grade C |  | Strength/certainty of wording (“should be considered”) |
| 317 | OBES | For overweight or obese children and adolescents, plan weight management programs that involve frequent contact with health professionals. | NHMRC 2013(41) | NHMRC - B |  | Guiding statement. |
| 318 | OBES | Routine laboratory endocrinology tests are not recommended unless influenced by puberty stage and family history | August, G et al 2008(42) |  |  | Guiding statement |
| 319 | OBES | Regular monitoring of BMI (ideally 3 monthly or more frequently) may be an appropriate component of approaches to weight management. | NHMRC 2013(41)  Alexander 2011  Lau, D et al 2006(43) |  |  | Strength/certainty of wording (“may be”) |
| 320 | OBES | Assist children and adolescents to get help for disordered eating, poor body image, depression and anxiety and weight-related bullying where these are present. | NHMRC 2013(41) |  |  | Guiding statement |
| 321 | OBES | Inform parents of children who have inexorably gained weight from early infancy and have risen above the 97th percentile for weight by 3yrs of age be informed of MC4R genetic testing (treatment is however not altered) | August, G et al 2008(42) |  |  | Guiding statement (treatment not altered). |
| 322 | OBES | More frequent contact with a health professional is generally more successful in the short term. In the longer term, the frequency of contact needs to be balanced against sustainability, cost and resources and the individual’s needs. | NHMRC 2013(41) |  |  | Guiding statement |
| 323 | OTIT | Review inflammation in 48hrs. If the inflammation has not resolved, consider these possible explanations:  - wrong diagnosis  - failure to take the medication (antibiotics not given or vomited)  - inappropriate antibiotics prescribed  - antibiotic reaction  - suppurative complication has developed (mastoiditis, facial paralysis, labyrinthitis, intracranial infection | RCH paed handbook 2009(9)  RCH Melb 2012 |  |  | Strength/certainty of wording (“consider”) |
| 324 | OTIT | Antibiotics should be a consideration only as acute symptoms resolve within 24hrs in most cases without antibiotics | RCH paed handbook 2009(9) |  |  | Strength/certainty of wording (“consideration”) |
| 325 | OTIT | Consider insertion of tympanostomy tubes only if:  - middle ear effusion present for at least 3months and appears likely to persist long term  - significant symptoms are present (recurrent AOM or functionally significant hearing loss which is resulting in speech delay, behavioural disturbance or poor school performance) | RCH paed handbook 2009(9) |  |  | Strength/certainty of wording (“consider”) |
| 326 | PREV | Identify family strengths, elicit concerns and promote parental confidence, competence and mental health | RACGP 2013(44) | Grade C |  | Guiding statement. |
| 327 | SEIZ | The first step in the management of the patient who is having a seizure is to assess and support airway, breathing and circulation. This will ensure that the seizure does not compromise supply of oxygenated blood to the brain and is not secondary to hypoxia and/or ischaemia. | NSW Health 2009(45)  SIGN 2005(46) |  |  | Guiding statement |
| 328 | SEIZ | Prolonged seizures and/or repeated doses of anti-epileptic medications may lead to compromise of breathing requiring ongoing support including intubation. Help from senior clinicians should be obtained for intubation. | NSW Health 2009(45) |  |  | Guiding statement |
| 329 | SEIZ | Assess neurological function.  - The AVPU (Alert, Voice, Pain, Unresponsive) score cannot be measured meaningfully during a seizure as a generalised seizure depresses the level of consciousness. | NSW Health 2009(45) |  |  | Guiding statement |
| 330 | TONS | Watchful waiting for recurrent throat infection prior to tonsillectomy:  - mild sore throats (i.e. six months)  - <7 episodes in previous year  - <5 episodes per year in past 2 years  - <3 episodes per year in past 3 years | SIGN 2010(47)  Baugh 2011(48) | SIGN - A  Baugh - recommendation |  | Guiding statement |
| 331 | URIN | Background to condition:  UTI cannot be diagnosed on symptoms alone.  There is no indication for culture of urine from a bag specimen.  In children that can void on request an MSU is an adequate sample. In younger children a clean catch is often adequate. In septic infants an SPA or catheter urine may be required.  Urinary dipstick testing is only a screening test for UTI. It has poor sensitivity and specificity.  Finding a UTI in a sick child does not exclude another site of serious infection (e.g. meningitis). Remember that 2% of young children will have asymptomatic bacteriuria and this may not be the cause of this acute presentation. Organisms may spread from urinary tract to elsewhere including meninges. Do not omit an LP if you are considering meningitis just because you have found a UTI. However, LP does not have to be performed in all children with UTI.  Prior antibiotic therapy may lead to negative urine culture in patients with UTI. The laboratory will test for antibacterial activity in the urine. | RCH Melb 2011(49) |  |  | Guiding statement (covered in other recommendations) |
| 332 | URIN | When to consider transfer to tertiary centre:  Child requiring care beyond the comfort level of the hospital | RCH Melb 2011(49) |  |  | Guiding statement  Strength/certainty of wording (“Strength/certainty”) |
| 333 | URTI | Parents of children should be given advice about the usual natural history of the illness, including the average total length of the illness:  acute otitis media: 4 days  acute sore throat/acute pharyngitis/acute tonsillitis: 1 week  common cold: 1½ weeks  acute rhinosinusitis: 2½ weeks  acute cough/acute bronchitis: 3 weeks | NICE 2008(50) |  |  | Unlikely to be documented. |
| 334 | URTI | Current evidence does not support the effectiveness of commonly used measures such as Vitamin C, Zinc, Echinacea or research based recommendations for symptom relief of viral URTI in children (i.e. antihistamines or decongestants) | Hart 2008(51) |  |  | Guiding statement.  Process not within audit sample or scope (i.e. not prescribed by healthcare practitioners) |

1. Makin E, Davenport M. Evaluation of the acute abdomen. Paediatrics and Child Health (United Kingdom). 2012;22 (6):217-23.

2. Cheung C. Approach to pediatric abdominal pain 2011. Available from: <http://learnpediatrics.sites.olt.ubc.ca/files/2011/11/abdominal-pain.pdf>.

3. Leung AKC, Sigalet DL. Acute abdominal pain in children. American Family Physician. 2003;67 (11):2321-6.

4. The Royal Children's Hospital Melbourne. Abdominal Pain Melbourne2013. Available from: <http://www.rch.org.au/clinicalguide/guideline_index/Abdominal_pain/>.

5. National Health and Medical Research Council. Clinical practice points on the diagnosis, assessment and management of Attention Deficit Hyperactivity Disorder in children and adolescents. Secondary Clinical practice points on the diagnosis, assessment and management of Attention Deficit Hyperactivity Disorder in children and adolescents 2012. Available from: <https://www.nhmrc.gov.au/guidelines/publications/mh26>.

6. Kohn M. Child and adolescent ADHD. Australian Doctor. 2008(November).

7. American Academy of Pediatrics; Subcommittee on Attention-Deficit/Hyperactivity Disorder - Steering committee on quality improvement and management. ADHD: Clinical practice guideline for the diagnosis, evaluation, and treatment of Attention-Deficit.Hyperactivity Disorder in children and adolescents. . Pediatrics. 2011;128.

8. Canadian Psychiatric Association (CPA). Clinical practice guidelines: management of anxiety disorders 2006. Available from: <https://ww1.cpa-apc.org/Publications/CJP/supplements/july2006/anxiety_guidelines_2006.pdf>.

9. Royal Childrens Hospital Melbourne. Paediatric Handbook - Eighth edition. Melbourne, Australia: Wiley-Blackwell; 2009.

10. Khalid-Khan S, Santibanez M-P, McMicken C, Rynn MA. Social Anxiety Disorder in Children and Adolescents: Epidemiology, Diagnosis, and Treatment. Pediatric Drugs. 2007;9(4):227-37.

11. British Columbia Medical Association. Anxiety and Depression in Children and Youth – Diagnosis and Treatment 2010. Available from: <http://www.bcguidelines.ca/pdf/depressyouth.pdf>.

12. O'Brien PG, Fleming L. Recognizing anxiety disorders. Nurse Practitioner. 2012;37(10):35-42.

13. National Institute for Health and Clinical Excellence (NICE). Social anxiety disorder: recognition, assessment and treatment (CG159) London2013. Available from: <http://www.nice.org.uk/nicemedia/live/14168/63868/63868.pdf>.

14. British Thoracic Society, & Scottish Intercollegiate Guidelines Network. British guideline on the management of asthma 2016. Available from: <https://www.brit-thoracic.org.uk/standards-of-care/guidelines/btssign-british-guideline-on-the-management-of-asthma/>.

15. National Asthma Council Australia. Asthma Management Handbook 2006. National Asthma Council; 2006.

16. Royal Australasian College of Physicians. A consensus approach for the paediatrician's role in the diagnosis and assessment of Autism Spectrum Disorders in Australia 2008. Available from: <https://www.racp.edu.au/index.cfm?objectid=B55D4FBA-BFD6-A9E7-152508A3109AC225>.

17. Scottish Intercollegiate Guidelines Network (SIGN). Assessment, diagnosis and clinical interventions for children and young people with autism spectrum disorders 2007. Available from: <http://www.sign.ac.uk/guidelines/fulltext/98/index.html>.

18. Tonge B, & Brereton A. Autism spectrum disorders. Australian Family Physician. 2011;40(9):672-77.

19. Australian Government Department of Health and Ageing. Early intervention for children with autism spectrum disorders: guidelines for best practice 2007. Available from: [www.health.gov.au/internet/publications/publishing.nsf/Content/mental-child-autbro-toc~mental-child-autbro-best](http://www.health.gov.au/internet/publications/publishing.nsf/Content/mental-child-autbro-toc~mental-child-autbro-best) - no longer available online.

20. American Academy of paediatrics (AAP): subcommittee on diagnosis and management of bronchiolitis. Diagnosis and management of bronchiolitis. Pediatrics. 2006;118 (4):1774-93.

21. NSW Health. Infants and Children: Acute management of bronchiolitis PD2012_004 Sydney2012. Available from: <http://www0.health.nsw.gov.au/policies/pd/2012/pdf/PD2012_004.pdf>.

22. Sydney Children's Hospital. Viral Bronchiolitis Inpatient Clinical Guidelines Sydney2011. Available from: <http://www.sch.edu.au/health/professionals/cpg/viral_bronchiolitis_inpatient_clinical_guidelines.pdf>.

23. Health for Kids in the South East SH. Evidence-based practice guideline for the management of croup in children 2007. Available from: No longer available online.

24. The Royal Children's Hospital Melbourne. Clinical practice guidelines: Croup (Laryngotacheobronchitis). 2011. Available from: <http://www.rch.org.au/clinicalguide/guideline_index/Croup_Laryngotracheobronchitis/>.

25. NSW Health. Children and infants - Acute management of croup 2010. Available from: <http://www0.health.nsw.gov.au/policies/pd/2010/PD2010_053.html>.

26. Australasian Paediatric Endocrine Group, and the Australian Diabetes Society. National evidence-based clinical care guidelines for type 1 diabetes in children, adolescents and adults. 2011. Available from: <https://www.nhmrc.gov.au/guidelines-publications/ext4>.

27. National Institute for health and Clinical Excellence (NICE). Depression in children and young people: Identification and management in primary, community and secondary care 2005.

28. Cheung AH, Zuckerbrot RA, Jensen PS, Ghalib K, Laraque D, Stein REK. Guidelines for adolescent depression in primary care (GLAD-PC): II. Treatment and ongoing management. Paediatrics. 2007;120(55):e1313-26.

29. NHMRC. Clinical practice guidelines: depression in adolescents and young adults. 2011. Available from: <https://www.nhmrc.gov.au/guidelines/publications/ext0007>.

30. The Royal Children's Hospital Melbourne. Diabetes Mellitus. Secondary Diabetes Mellitus 2013. Available from: <http://www.rch.org.au/clinicalguide/guideline_index/Diabetes_Mellitus/>.

31. Scottish Intercollegiate Guidelines Network (SIGN). Management of atopic eczema in primary care (CG125). Secondary Management of atopic eczema in primary care (CG125) 2011. Available from: <http://www.sign.ac.uk/guidelines/fulltext/125/index.html>.

32. Royal Children's Hospital M. Eczema management. Secondary Eczema management 2013. 2013. Available from: <http://www.rch.org.au/rchcpg/hospital_clinical_guideline_index/Eczema_management/>.

33. NSW Kids and Families. Policy directive: Children and infants with fever - acute management. 2010. Available from: <http://www0.health.nsw.gov.au/policies/pd/2010/PD2010_063.html>.

34. SA Child Health Clinical Network. Management of fever without focus in children (excluding neonates). Secondary Management of fever without focus in children (excluding neonates) 2013. Available from: <http://www0.health.nsw.gov.au/policies/pd/2010/PD2010_063.html>.

35. National Institute for Health and Clinical Excellence (NICE). Feverish illness in children: assessment and initial management in children younger than 5 years 2013. Available from: <https://www.nice.org.uk/guidance/cg160>.

36. Bhavsar H, Cullen M, Beattie RM. Gastro-oesophageal reflux in infancy. Paediatrics and Child Health. 2011;21(9):394-400.

37. Vandenplas Y, Rudolph CD, Di Lorenzo C, Hassall E, Liptak G, Mazur L, et al. Pediatric gastroesophageal reflux clinical practice guidelines: Joint recommendations of the North American Society for Pediatric Gastroenterology, Hepatology, and Nutrition (NASPGHAN) and the European Society for Pediatric Gastroenterology, Hepatology, and Nutrition (ESPGHAN). Journal of Pediatric Gastroenterology and Nutrition. 2009;49(4):498-547.

38. Allen K. Gastro-oesophageal reflux in children. Australian Family Physician. 2012;41(5):268-72.

39. The Royal Children's Hospital Melbourne. Trauma - Head Injury ND. Available from: <http://www.rch.org.au/clinicalguide/guideline_index/Head_Injury_Guideline/>.

40. Families NKa. Children and infants - acute management of head injury 2011. Available from: <http://www0.health.nsw.gov.au/policies/pd/2011/pdf/PD2011_024.pdf>.

41. National Health & Medical Research Council (NHMRC). Clinical practice guidelines for the management of overweight and obesity in adults, adolescents and children in Australia Canberra 2013. Available from: <http://www.nhmrc.gov.au/_files_nhmrc/publications/attachments/n57>.

42. August GP, Caprio S, Fennoy I, Freemark M, Kaufman FR, Lustig RH, et al. Prevention and treatment of pediatric obesity: An Endocrine Society clinical practice guideline based on expert opinion. Journal of Clinical Endocrinology and Metabolism. 2008;93 (12):4576-99.

43. Lau DC, Douketis JD, Morrison KM, Hramiak IM, Sharma AM, Ur E. 2006 Canadian clinical practice guidelines on the management and prevention of obesity in adults and children [summary]. CMAJ : Canadian Medical Association journal = journal de l'Association medicale canadienne. 2007;176 (8):S1-13.

44. Royal Australian College of General Practitioners. Guidelines for preventive activities in general practice 8th edition - Preventive activities in children and young people. 2013. Available from: <http://www.racgp.org.au/your-practice/guidelines/redbook/>.

45. NSW Kids and Families. Children and infants with seizures - acute management 2009. Available from: <http://www0.health.nsw.gov.au/policies/pd/2009/PD2009_065.html>.

46. Scottish Intercollegiate Guidelines Network. Diagnosis and management of epilepsies in children and young people 2005. Available from: <http://www.sign.ac.uk/pdf/sign81.pdf>.

47. Scottish Intercollegiate Guidelines Network (SIGN). Management of sore throat and indications for tonsillectomy Edinburgh2010. Available from: <http://www.sign.ac.uk/pdf/sign117.pdf>.

48. Baugh RF, Archer SM, Mitchell RB, Rosenfeld RM, Amin R, Burns JJ, et al. Clinical practice guideline: Tonsillectomy in children. Otolaryngology - Head and Neck Surgery. 2011;144 (SUPPL.1):S1-S30.

49. Royal Children's Hospital Melbourne. Urinary Tract Infection Guideline Melbourne2011. Available from: <http://www.rch.org.au/clinicalguide/guideline_index/Urinary_Tract_Infection_Guideline/>.

50. National Institute for Health and Care Excellence (NICE). Respiratory tract infections - antibiotic prescribing: Prescribing of antibiotics for self-limiting respiratory tract infections in adults and children in primary care 2008. Available from: <https://www.google.com.au/#q=Respiratory+tract+infections+-+antibiotic+prescribing:+Prescribing+of+antibiotics+for+self-limiting+respiratory+tract+infections+in+adults+and+children+in+primary+care>.

51. Hart A, Patti A, Noggle B, Haller-Stevenson E, Hines L. Acute Respiratory Infections and Antimicrobial Resistance. American Journal of Nursing. 2008;108(6):56-65.
