# Supplementary material for: Clinical indicators for common paediatric conditions: Processes, provenance and products of the CareTrack Kids study
Source: PLoS One. 2019 Jan 9;14(1):e0209637. doi: 10.1371/journal.pone.0209637 (PMC6326465; doi:10.1371/journal.pone.0209637)
Supplement: S3 Table — (DOCX) [file pone.0209637.s003.docx]

**S3 Table. *CareTrack Kids* list of excluded clinical indicators.**

The rejected clinical indicators are presented by condition, with their source, level of evidence, phase of care, whether they were measured for under- or over-use, and stage (of the development process) at which and reasons why they were excluded.

| **Condition** | | **Rejected indicator** | **Source** | **Level of evidence** | **Phase of care** | | | | **Stage of development process excluded** | | | | | | **Reason(s) for exclusion** | | | | | **Comment(s)** |
| --- | --- | --- | --- | --- | --- | --- | --- | --- | --- | --- | --- | --- | --- | --- | --- | --- | --- | --- | --- | --- |
|  |  |  |  |  | **Diagnosis** | **Treatment** | **Ongoing management** | **Screening** | **Internal review**  **Round 1** | **Internal review**  **Round 2** | **Internal review**  **Round 3** | **External review (wiki)**  **Round 1** | **External review (wiki)**  **Round 2** | **Post-wiki** | **A** | **F** | **I** | **Covered in other indicator(s)** | **Low ‘appropriateness’ score (<7)** |  |
| ABDO | | Children presenting with acute abdominal pain do NOT routinely require rectal or vaginal examination | Holland 2009(1)  Makin 2012(2)  RCH Melb 2013(3) | consensus | ✔ |  |  |  |  | ✔ |  |  |  |  | ✔ |  |  |  |  | Completing and documenting this assessment in every patient is not appropriate |
| ABDO | | Children presenting with acute abdominal pain who meet ALL of the following criteria do NOT receive routine investigations as the first line of treatment:  - normally well AND - unlikely to have renal or hepatic disease | Holland 2009(1) | consensus | ✔ |  |  |  |  | ✔ |  |  |  |  |  | ✔ |  |  |  | Difficult to define “normally well” and “unlike to have renal or hepatic disease”. Difficult to make this indicator work for a surveyor, stick with investigations that are targeted based on history and examination findings. |
| ABDO | | Children presenting with acute abdominal pain and any of the following do NOT receive a contrast enema:  - peritonitis, OR  - significant dehydration OR - an established bowel obstruction | RCH paed handbook 2009(4) | consensus | ✔ |  |  |  |  | ✔ |  |  |  |  |  |  | ✔ |  |  | Contrast enemas infrequently done and would present a rare deviation from current clinical best practice. Unlikely relevant to GPs.  Low impact and prevalence. |
| ABDO | | Children presenting with acute abdominal pain who are severely unwell have IV access inserted at presentation. | RCH Melb 2013(5) | consensus |  | ✔ |  |  | ✔ |  |  |  |  |  |  | ✔ | ✔ |  |  | Should not have to be stated.  How to define “severely unwell” and “at presentation” (i.e. at triage in ED, or any time in ED, etc). Covered in other indicators that refer to fluid resuscitation. |
| ABDO | | Children presenting with acute abdominal pain where a surgical intervention is suspected are managed as follows:  - fasted from food and fluids AND  - reviewed by a surgeon AND  - administered IV antibiotics as prescribed | RCH Melb 2013(5) | consensus |  | ✔ |  |  |  | ✔ |  |  |  |  |  | ✔ | ✔ |  |  | Lack of agreement about when to administer antibiotics  Difficulty in defining “where a surgical intervention is suspected/expected”.  We recommend EXCLUSION of this indicator for the following reasons:  (a) it will be difficult for surveyors to determine if “a surgical intervention is suspected” therefore, it would require the following rewording “Children presenting with acute abdominal pain where a surgical review is planned”…  In which case, the indicator is essentially assessing whether:  (b) children are fasted in the interim for possible surgery, AND  (c) that children are followed up and reviewed by a surgeon AND  (d) receive their prescribed antibiotics (and presumably other medications)  None of these compliance actions are specific to acute abdominal pain, they seem to reflect standard operating procedures rather than “essential care”, and are unlikely to be high impact.  Therefore, we are suggesting this indicator should be excluded for the following reasons:  (a) importance (this is what would be routinely expected for any child/adult where surgery may be indicated)  (b) difficulty in defining “where a surgical intervention is suspected/expected”  (c) lack of consensus re: when to administer antibiotics (as per Reviewer 2)  (d) use of the term “consider” in the original recommendation |
| ABDO | | Children presenting with acute abdominal pain and any of the following are referred to the surgical team for consultation:  - Severe or increasing abdominal pain with progressive signs of deterioration OR  - Bile-stained or feculent vomitus OR  - Involuntary abdominal guarding/rigidity OR  - Rebound abdominal tenderness OR  - Marked abdominal distension with diffuse tympany OR  - Signs of acute fluid or blood loss into the abdomen OR  - Significant abdominal trauma OR  - Abnormal testicular examination (abnormal lie, high riding testis) OR  - Suspected surgical cause for the pain OR  - Abdominal pain without an obvious etiology | Leung 2003(6)  RCH Melb 2013(5) | consensus |  | ✔ |  |  |  | ✔ |  |  |  |  | ✔ |  |  |  |  | Large proportions of children with these signs and symptoms have non-specific pain/cause and do not require surgical consultation.  Who is surgical team? And what is referred? (i.e. Consultant vs on-call Reg, phone call, transfer, review, etc). |
| ABDO | | Children presenting with acute abdominal pain classified as moderate were administered oxycodone along with paracetamol or ibuprofen. | Leung 2003(6)  Makin 2012(2)  NSW Health 2005(7) | A |  | ✔ |  |  |  |  |  |  |  | ✔ |  |  |  |  | ✔ | Low score < 7 |
| ABDO | | Children with non-specific abdominal pain can be discharged home after the following has occurred:  - history and examination performed and documented and no concerning features found AND  - urinalysis and/or MCS performed and unremarkable AND  - a clear follow up plan for review is arranged AND  - information given to parents of when to seek medical attention. | RCH Melb 2013(5) | consensus |  |  | ✔ |  |  | ✔ |  |  |  |  |  |  |  | ✔ |  | History and examination is as outlined at the beginning of this document and it does not differ based on whether the pain is ‘nonspecific’ or ‘acute’  I don’t think it really fits to have a new focus on non-specific abdominal pain after the previous focus on acute abdominal pain. It is also hard to define “non-specific abdominal pain” except as a diagnosis of exclusion. |
| AGE | | Children presenting with gastroenteritis should NOT be prescribed Ondansetron if aged <6months or weigh <8kg | RCH Melb 2013(5) | consensus |  | ✔ |  |  | ✔ |  |  |  |  |  |  |  | ✔ | ✔ |  | Covered in another indicator (Ondansetron is not recommended in GP under age 2 years)  Too rare |
| AGE | | Children presenting with gastroenteritis may be treated with probiotics if used in conjunction with an Oral Rehydrating Solution (ORS) | Churgay 2012(8) | consensus |  | ✔ |  |  | ✔ |  |  |  |  |  | ✔ |  |  |  |  | “may be” wording  A Cochrane review of 63 studies concluded that probiotics reduce the duration of diarrhoea by approx. one day when used in conjunction with an ORS.  Evidence unclear. |
| AGE | | Children with gastro who have no/mild dehydration presenting to the emergency department should be discharged home following:  - a trial of oral fluids (10-20ml over 1 hour), which has been retained  - clinical signs of dehydration have improved  - has passed urine  - parents understand the importance of oral rehydration and are able to continue this at home  - appropriate follow-up arranged e.g. review with GP next day  - parents advised that a few small vomits does not mean the trial has failed | RCH Melb 2013(5) | consensus |  | ✔ |  |  |  | ✔ |  |  |  |  | ✔ | ✔ |  |  |  | Too complex  Trial of fluids not mandatory if no or minimal dehydration |
| AGE | | Children with gastroenteritis who are being managed with IV fluids should continue (as tolerated) their age appropriate diet and fluids. | Cincinnati 2011(9) | consensus |  | ✔ |  |  |  | ✔ |  |  |  |  |  | ✔ |  |  |  | No Australian primary source.  Unlikely to be documented. |
| AGE | | Children presenting to the emergency department with gastroenteritis and moderate dehydration should be rehydrated in order of preference with progression the next stage if indicated:  1. “Aggressive” and diligent oral rehydration (breastfeeding, ORS), reassess after 2hrs and consider the next step if ongoing losses continue  2a. RAPID NG ORS rehydration 25ml/kg/hr for 4 hrs (max 300ml) eg Gastrolyte®, Hydralyte®, Pedialyte®  - if vomiting continues once NG rehydration has commenced consider administration of ondansetron and slow NG fluids temporarily  - Ondansetron wafer dose: 8-15kg = 2mg, 15-30kg =4mg, >30kg =8mg  2b. SLOWER NG (SEE DEFINITIONS) rehydration should occur if: infants <6mths, presence of comorbidities, significant abdominal pain and consists or replacing the deficit over 6hrs (10ml/kg/hr) and then daily maintenance over the next 18hrs (dependent on weight).  Progress to IV fluids (severe dehydration management) if :  - vomiting continues on NG rehydration (despite halving the rate)  - profuse ongoing diarrhoea | NSW Health 2010(10) | consensus |  |  |  |  |  | ✔ |  |  |  |  |  | ✔ |  |  |  |  |
| AGE | | Children presenting with gastroenteritis and no/mild clinical signs of dehydration should receive parental advice about planned follow-up and review if there is a failure to improve, deterioration or development of new signs | NSW Health 2010(10) | consensus |  |  | ✔ |  | ✔ |  |  |  |  |  |  | ✔ | ✔ |  |  | Unlikely to be documented. Low impact. |
| AGE | | Infants should be immunised against rotavirus | Cincinnati 2011(9) | Level I |  |  |  | ✔ | ✔ |  |  |  |  |  |  |  | ✔ |  |  | No Australian primary source  Low impact on management of acute illness |
| AGE | | Children presenting with gastroenteritis and the following clinical signs or symptoms - reduced urine output, thirst and no physical signs, are diagnosed as not dehydrated | NSW Health 2010(10) | consensus |  |  |  |  | ✔ |  |  |  |  |  |  |  |  |  |  |  |
| AGE | | Children presenting with gastroenteritis and the following clinical signs or symptoms - reduced urine output, thirst, dry mucous membranes, and mild tachycardia are diagnosed with mild dehydration (3% of body weight). | NSW Health 2010(10) | consensus | ✔ |  |  |  | ✔ |  |  |  |  |  |  |  |  |  |  |  |
| AGE | | Children presenting with gastroenteritis and mild signs plus the following signs - tachycardia, abnormal respiratory pattern, lethargy, reduced skin turgor, sunken eyes are diagnosed with moderate dehydration (5% of body weight). | NSW Health 2010(10) | consensus | ✔ |  |  |  | ✔ |  |  |  |  |  |  |  |  |  |  |  |
| AGE | | Children presenting with gastroenteritis and moderate signs plus the following signs - poor perfusion (skin mottled, cool limbs, slow capillary refill, altered consciousness), shock (thready peripheral pulses with marked tachycardia and other signs of poor perfusion) are diagnosed with severe dehydration (10% of body weight) | NSW Health 2010(10) | consensus | ✔ |  |  |  | ✔ |  |  |  |  |  |  |  |  |  |  |  |
| AGE | | Children presenting to the GP with gastroenteritis should be referred to ED/hospital if: - if the diagnosis is in doubt - infants/children have moderate or severe dehydration - there are electrolyte abnormalities - they are at high risk of dehydration on the basis of age (<6mths) with a high frequency of diarrhoea (8 in 24hrs) and vomiting (>4 in 24hrs). They should be observed for 4-6hrs to ensure adequate maintenance of hydration -they are high risk infants/children (ileostomy, short gut, cyanotic heart disease, chronic renal disease, metabolic disorders and malnutrition) - infants/children whose parents and carers are thought to be unable to manage the child’s condition at home - IV fluids are required for more than 24hrs | Cincinnati 2011(9)  Heinz 2008(11)  RCH Melb 2013(5)  RCH paed handbook 2009(4) | consensus |  | ✔ |  |  | ✔ |  |  |  |  |  |  |  |  |  |  |  |
| AGE | | Children admitted to hospital with gastroenteritis and severe clinical signs of dehydration should receive: - oxygen until signs of shock are reversed. - IV access (use the intraosseous route if required) - blood tests: EUC, BGL (if possible)  - IV bolus of 20mL/kg 0.9% NaCl or Hartmanns stat. - regular assessments for signs of shock  - a repeat IV fluid bolus if necessary until signs of shock are reversed (organ perfusion restored), if >40ml/kg boluses required involve senior staff and ICU - ORS once initial resus complete (infants =30ml/hr, toddlers =60ml/hr, older children =90ml/hr + 10ml/kg for every loose stool or vomit) - their hydration status assessed. Based on this assessment administer IV fluid replacement over 24 hours: 0.9% NaCl + 2.5% Glucose or 0.45% NaCl + 2.5% Glucose  - frequent assessment of fluid balance and clinical assessment  - continuous monitoring | Churgay 2012(8)  Kelly 2007(12)  NICE 2009(13)  Noone 2012(14)  NSW Health 2010(10)  RCH Melb 2013(15)  RCH paed handbook 2009(4)  WCH 2010(16) | consensus |  | ✔ |  |  | ✔ |  |  |  |  |  |  |  |  |  |  |  |
| AGE | | Children presenting with gastroenteritis and no/mild clinical signs of dehydration should receive parental advice about planned follow-up and review if there is a failure to improve, deterioration or development of new signs | NSW Health 2010(10) | consensus |  |  | ✔ |  | ✔ |  |  |  |  |  |  |  |  |  |  |  |
| BRON | | Infants (aged <12 months) with acute bronchiolitis with any of the following additional risk factors are assessed in the hospital setting:  - full term infants up to 3mths of age OR  - premature or of low-birth weight for gestational age OR  - chronic lung disease (bronchopulmonary dysplasia [BPD], congenital lung abnormalities, cystic fibrosis) OR  - congenital heart disease OR  - immunodeficient  - structural lung disease | AAP 2006(17)  NSW Health 2012(18)  SCH 2011(19)  Zentz 2011(20) | Level B  Level C | ✔ |  |  |  |  | ✔ |  |  |  |  |  | ✔ |  |  |  | Admission should be considered but not all cases will require inpatient management. Difficult to capture in a medical record audit. |
| BRON | | Children diagnosed with bronchiolitis are prescribed oxygen if they have any of the following:  - SpO2 consistently falls below 95% OR  - hypoxia (restlessness, agitation, drowsiness) OR  - increased WOB (chest wall retractions, nasal flaring) OR  - increasing fatigue or tiring with feeds OR  - cyanosis | NSW Health 2012(18)  Zentz 2011(20) | Level B |  | ✔ |  |  |  | ✔ |  |  |  |  |  |  |  | ✔ |  | Content covered in previous indicators around severity of bronchiolitis and management. Some recommendations are contradictory (i.e. AAP says 90%, NSW guideline states less than 92% and consider if 93-95% and signs present. |
| BRON | | Infants (aged <12months) with bronchiolitis should have their fluids replaced based on the degree of tachypnoea, fatigue, distress, risk of pulmonary aspiration and amount of tenacity of secretions with one of the following:  - oral: small frequent breast/bottle (if usually 4hrly consider 2-3hrly) OR  - NG: 2hrly bolus feeds of 75% required intake (first choice is breast milk). Observed for increase in WOB post insertion of tube OR  - IV: severe bronchiolitis not tolerating oral/NG, restrict fluids to 75%, check ELU prior to commencement, offer small comfort feeds (10-20ml) | RCH MELB 2012(21)  SCH 2011(19) | consensus |  | ✔ |  |  |  | ✔ |  |  |  |  |  | ✔ |  | ✔ |  | Suggest EXCLUSION of this indicator for two reasons  1. due to feasibility as it will be difficult for the surveyor to objectively access the degree of tachypnoea, fatigue, distress etc without strict criteria.  2. Already covered in previous indicators and wording is inconsistent ie IV fluids (severe) and small frequent feeds and NG considered (moderate). |
| ANXI | | Children with anxiety should have ALL of the following assessed at diagnosis:  - behavioural  - cognitive  - family  - school  - developmental | RCH paed handbook 2009(4) | consensus |  |  |  |  | ✔ |  |  |  |  |  |  |  |  | ✔ |  | Combined with ANXI01. |
| ANXI | | Children with anxiety receive Cognitive Behavioural Therapy which includes:  - corrective education about anxiety and feared situations;  - somatic management skills such as progressive muscle relaxation, diaphragmatic breathing, and self-monitoring;  - cognitive restructuring to identify maladaptive thoughts and teach coping-focused thinking;  - exposure techniques involving gradual, systematic, and controlled desensitization to feared situations;  - relapse prevention plans to consolidate and generalize skills that have been learned | British Columbia 2010(22)  Madden 2007(23)  Sakolsky 2008(24) | consensus |  | ✔ |  |  | ✔ |  |  |  |  |  |  | ✔ |  | ✔ |  | CBT included in another indicator.  This level of detail will not be document therefore not feasible for surveyors to gather these data. |
| ANXI | | Children with anxiety receive 14-18 CBT sessions over 3-4 months. | CPA  2006(25)  Sakolsky 2008(24) | consensus |  |  | ✔ |  |  |  |  |  |  |  | ✔ |  |  |  |  | Not appropriate within the Australian health setting.  Almost all children and adolescents access their care under the Medicare Better Access program which allows for 10 sessions over a calendar year. They can get more (if they pay) but most don’t go for even that many because they can’t afford to and most tend to do well with 6-10 session - especially these days when phone app CBT has been shown to be as good as face to face CBT (at least in adults - not sure about evidence in adolescents).  Under Medicare, 6 or 12 sessions are covered with a clinical psychologist, usually weekly or 2-weekly. |
| ANXI | | Children with anxiety who are prescribed pharmacotherapy are monitored - weekly for the first 4 weeks, AND  - every 2 weeks for weeks 4-8, AND  - at 3-months, AND  - at 6 months | British Columbia 2010(22)  Sakolsky 2008(24) | consensus |  |  |  |  |  | ✔ |  |  |  |  | ✔ |  |  | ✔ |  | EXCLUDED for the following reasons:  (1) No consensus among reviewers re: timeframes  (2) Timeframes not included in the original recommendation  (3) Children with depression are often prescribed similar pharmacotherapy and there are no recommendations that provide strict criteria for the frequency of monitoring. A comparable indicator DEPR08 states children are monitored (which is already covered in ANXI06) |
| ANXI | | Children with moderate or severe anxiety have an emergency safety plan. | British Columbia 2010(22) | consensus |  | ✔ |  |  | ✔ |  |  |  |  |  |  |  |  | ✔ |  |  |
| ANXI | | Children with anxiety who receive SSRIs are monitored for the following side-effects:  - headache,  - gastric distress,  - insomnia, AND  - increased motor activity | Sakolsky 2008(24) | consensus |  | ✔ |  |  | ✔ |  |  |  |  |  |  | ✔ |  |  |  |  |
| ANXI | | Children with anxiety receiving SSRIs are monitored for:  - irritability,  - sleep problems,  - increased agitation,  - akathisia,  - suicidal thoughts and  - behaviour  using:  - SSRI Monitoring Form, and  - Measurement of Functional Change, and  - Family/caregiver diaries | British Columbia 2010(22)  Sakolsky 2008(24) | consensus |  |  | ✔ |  | ✔ |  |  |  |  |  |  |  |  | ✔ |  |  |
| ANXI | | Children with severe generalised anxiety disorder and/or specific fears are referred to a mental health specialist. | British Columbia 2010(22)  RCH paed handbook 2009(4) | consensus |  | ✔ |  |  | ✔ |  |  |  |  |  |  | ✔ |  |  |  | EXCLUSION was based on feasibility, in that from the medical record review, it will not be possible for surveyors to determine the reasons for (or against) referring patients to mental health specialists, such as:  - whether referral to a mental health specialist was appropriate or not  - whether referral was indicated but just not done  - whether non-compliance (i.e. not referring) was due to a lack of mental health specialist services (especially in rural settings). |
| ANXI | | Children (diagnosed or newly diagnosed) with anxiety are NOT prescribed pharmacotherapy as first line management. | AACAP 2007(26)  CPA  2006(25)  Madden 2007(23) | Level I |  | ✔ |  |  |  |  |  |  |  | ✔ |  |  |  |  | ✔ | Low score |
| ANXI | | Children with anxiety are commenced on a SSRI at 1/4 or 1/2 of adult dose. | AACAP 2007(26)  CPA  2006(25)    Sakolsky 2008(24)  WA TAG 2008(27) | consensus |  |  |  |  |  |  |  |  |  | ✔ |  |  |  |  | ✔ | Low score |
| ANXI | Children with anxiety receive SSRIs for at least six months. | | British Columbia 2010(22)  Sakolsky 2008(24)  Madden 2007(23) | consensus |  |  |  |  |  |  |  |  |  | ✔ |  |  |  |  | ✔ | Low score |
| ASTH | Children presenting with symptoms suggestive of a high probability of having asthma are:  - commenced on a trial of treatment AND  - reviewed to assess response | | BTS and SIGN 2012(28) | consensus |  | ✔ |  |  |  |  |  |  |  |  |  |  |  | ✔ |  | Merged with another indicator |
| ASTH | Children presenting with symptoms suggestive of a low probability of having asthma have:  - further detailed investigations AND  - are referred to a specialist | | BTS and SIGN 2012(28) | consensus |  | ✔ |  |  | ✔ |  |  |  |  |  |  | ✔ |  |  |  | Unable to determine from medical record review |
| ASTH | | Children aged >5 years presenting with symptoms suggestive of an intermediate probability of asthma are:  - commenced on a bronchodilator  - reviewed in 4-6 weeks (using spirometry) for a change in FEV1 or PEF. | BTS and SIGN 2012(28)  NAC 2006 | consensus |  | ✔ |  |  |  | ✔ |  |  |  |  |  | ✔ |  |  |  | Too difficult for surveyors to determine “intermediate probability”, very subjective and unable to define. |
| ASTH | | Children with asthma being treated with inhaled steroids are considered for a dose reduction every 3 months | BTS and SIGN 2012(28) | consensus |  |  | ✔ |  |  | ✔ |  |  |  |  |  | ✔ |  |  |  | It will be difficult for the surveyor to assess compliance with this indicator ie where there is no change to the dose we will have to score it as “NO” as it doesn’t indicate if there has been a consideration or not. |
| ASTH | | Parents of children with asthma are advised to administer inhaled short-acting β2 agonists immediately prior to exercise | BTS and SIGN 2012(28) | consensus |  |  |  |  | ✔ |  |  |  |  |  | ✔ |  |  |  |  | Only pertinent for those with exercise induced asthma – therefore exclude. Not a routine recommendation. |
| ASTH | | Children with asthma aged < 5 years already prescribed inhaled steroids 200-400mcg/day that require additional add on therapy are considered for addition of leukotriene receptor antagonist. | BTS and SIGN 2012(28) | consensus |  |  | ✔ |  | ✔ |  |  |  |  |  | ✔ |  |  |  |  | Use of “considered”. |
| ASTH | | Children with asthma aged < 5 years that require additional add-on therapy that are prescribed leukotrene receptor antagonist alone are considered for addition of an inhaled steroid 200-400 mcg/day | BTS and SIGN 2012(28) | consensus |  |  | ✔ |  | ✔ |  |  |  |  |  | ✔ |  |  |  |  | Use of “considered”. |
| ASTH | | Children aged < 5 years with persistent poorly controlled asthma are referred to a respiratory paediatrician | BTS and SIGN 2012(28)  NAC 2006(29) | consensus |  | ✔ |  |  | ✔ |  |  |  |  |  |  |  |  | ✔ |  | Combined with other indicators 12 and 13. |
| ASTH | | Children with asthma aged > 2 years presenting with an acute exacerbation who require short acting β2 agonists more often than four-hourly have long-acting β2 agonists discontinued. | BTS and SIGN 2012(28) | consensus |  | ✔ |  |  | ✔ |  |  |  |  |  |  | ✔ | ✔ |  |  | Difficult to determine from the medical record.  Likely to be few cases. |
| ASTH | | Children aged > 2 years with asthma who present with an acute exacerbation that are prescribed oral prednisolone receive it for at least 3 days | BTS and SIGN 2012(28)  NAC 2006(29) | consensus |  | ✔ |  |  | ✔ |  |  |  |  |  | ✔ |  |  |  |  | Lack of consensus regarding “at least” (i.e. dosage) and recommendations vary across guidelines. |
| ASTH | | Children discharged from hospital after an acute asthma episode are reviewed by their GP or OPD within 30 days of discharge | BTS and SIGN 2012(28)  NAC 2006(29) | consensus |  |  | ✔ |  |  | ✔ |  |  |  |  |  | ✔ |  |  |  | I am suggesting we EXCLUDE this indicator as we will only be able to measure compliance if we:  (a) have the HOSP medical record and the child returns to OPD or  b) look through the GP record for evidence the child has been to hospital and then seen their GP within 30days post discharge (this will be a small number). |
| ASTH | | Children with asthma discharged after an acute episode receive a written action plan | BTS and SIGN 2012(28) | consensus |  | ✔ |  |  | ✔ |  |  |  |  |  |  |  |  | ✔ |  | Combined with indicator 33 |
| ASTH | | Parents of children with asthma receive verbal and written instructions and information on drug treatment | BTS and SIGN 2012(28) | consensus |  | ✔ |  |  |  |  |  |  |  |  |  | ✔ |  |  |  | Not likely to be documented. |
| ASTH | | Children aged less than 5years with asthma and a severe exacerbation are prescribed oral prednisolone (initially 2mg/kg, followed by 1mg/kg daily for 3 days). | BTS and SIGN 2012(28) | consensus |  | ✔ |  |  |  |  |  | ✔ |  |  | ✔ |  |  |  |  | Lack of consensus around age group, use of steroids and prescription. |
| ASTH | | Children aged > 2 years with asthma that present to an ED or hospital with an acute exacerbation that have a SpO2 < 92% on air after initial bronchodilator treatment are admitted for intensive inpatient care | BTS and SIGN 2012(28) | consensus |  | ✔ |  |  |  |  |  |  |  |  |  |  |  |  |  |  |
| ADHD | | Children aged under 7 years diagnosed with ADHD (hyperactive, impulsive and inattentive behaviour) have evidence in the medical record that they were: - observed by a specialist clinician over several months AND  - reviewed as they commenced school prior to having a diagnosis confirmed | NHMRC 2012(30) | consensus | ✔ |  |  |  |  | ✔ |  |  |  |  |  | ✔ |  |  |  | A diverse range of views from the reviewers. Two reviewers excluded and questioned feasibility. The indicator is likely to apply to only a small number of children, and therefore prevalence in the medical record audit may be an issue. |
| ADHD | | Children diagnosed with ADHD (hyperactive, impulsive and inattentive behaviour) have evidence in the medical record that the following occurred at diagnosis :  - complete physical examination AND  - holistic assessment of the individual’s needs, family, social and educational circumstances AND - assessed for co-existing illnesses. | AAP 2011(31)  NHMRC 2012(30) | Level B | ✔ |  |  |  |  | ✔ |  |  |  |  |  |  |  | ✔ |  | Merged with indicator 2 |
| ADHD | | Children with ADHD have evidence in the medical record that an individualised management plan was developed with consideration of the following:  – the best available explanation for the child/adolescent’s presentation and specific interventions for issues that might underpin the presentation AND – the severity of ADHD symptoms and the level of subsequent impairment across multiple settings AND  – the child/adolescent’s overall health and associated problems AND – comorbid disorders that may require specific treatment strategies AND – the family’s resources and their capacity to adhere to the plan. | Kohn, M 2008(32)  NHMRC 2012(30) | consensus |  | ✔ |  |  |  | ✔ |  |  |  |  |  | ✔ |  | ✔ |  | Those thoughts will lie behind the clinicians management plan but are too lengthy to be documented each time  Covered in earlier indicators. |
| ADHD | | Children with ADHD have had their management plan reviewed/documented by their GP | NHMRC 2012(30) | consensus |  |  | ✔ |  |  | ✔ |  |  |  |  | ✔ | ✔ |  |  |  | May be reviewed/documented by other healthcare professionals (e.g. paediatrician). No consensus on timeframes for regularity of review. |
| ADHD | | Adolescents being managed with ADHD that display internalising symptoms are treated with cognitive behavioural therapy | Kohn, M 2008(32)  NHMRC 2012(30) | consensus |  | ✔ |  |  | ✔ |  |  |  |  |  |  |  |  | ✔ |  | This would be done privately by psychologists and already covered in other indicators re: holistic action plan  IDEAL, BUT NUMBERS ENGAGED IN ONGOING CARE (AND COST ISSUES) MAY MAKE THIS LESS FEASIBLE.  GP MAY BE INVOLVED IN PREPARING MANAGEMENT PLAN FOR REFERRAL TO AH |
| ADHD | | Children being managed for ADHD who are receiving psychological therapy have had the acceptability and effectiveness of treatment monitored | NHMRC 2012(30) | consensus |  |  | ✔ |  |  | ✔ |  |  |  |  |  | ✔ |  |  |  | Difficult to determine reliably from the medical record. |
| ADHD | | Children with ADHD that have been prescribed stimulant medication (methylphenidate and dexamphetamine sulphate) have documented in the medical record that a conversation on the benefits and harms of taking medication were discussed with the child and the family prior to commencing medication. | NHMRC 2012(30) | consensus |  |  |  |  | ✔ |  |  |  |  |  |  |  |  | ✔ |  | Covered in another indicator |
| ADHD | | Children with ADHD that have been prescribed stimulant medication (methylphenidate and dexamphetamine sulphate) have had a comprehensive assessment undertaken by a paediatric neurologist or child/adolescent psychiatrist prior to commencing medication | NHMRC 2012(30) | consensus |  | ✔ |  |  | ✔ |  |  |  |  |  |  |  |  | ✔ |  | Covered in another indicator |
| ADHD | | Children with ADHD that have been prescribed stimulant medication (methylphenidate and dexamphetamine sulphate) have evidence in the medical record of a discussion on the risks, benefits and concerns of treatment | NHMRC 2012(30) | consensus |  | ✔ |  |  | ✔ |  |  |  |  |  |  |  |  | ✔ |  | Covered in another indicator |
| ADHD | | Children with ADHD that have been prescribed stimulant medication (methylphenidate and dexamphetamine sulphate) and then have it stopped have regular assessments after the medication has been stopped to determine the response | NHMRC 2012(30) | consensus |  |  | ✔ |  | ✔ |  |  |  |  |  |  | ✔ |  |  |  | 2 reviewers indicated feasibility issues and 1 excluded. We believe surveyor inter-rater reliability would be relatively poor as it will be difficult for the surveyor to navigate. EXCLUDE |
| ADHD | | Children aged less than 7 years, being managed with ADHD have evidence that environmental and family interventions were trialled and were not effective prior to prescribing stimulant medication (methylphenidate and dexamphetamine sulphate). | AAP 2011(31)  Kohn, M 2008(32)  NHMRC 2012(30) | Level A |  |  | ✔ |  | ✔ |  |  |  |  |  |  |  |  |  |  |  |
| ADHD | | Children with ADHD who have been prescribed stimulant medication (methylphenidate and dexamphetamine sulphate) have their medication ceased if:  - there are unacceptable side effects AND  - there is no demonstrated benefit. | NHMRC 2012(30) | consensus |  |  | ✔ |  |  |  | ✔ |  |  |  |  |  |  | ✔ |  |  |
| ADHD | | Children with ADHD that have been prescribed stimulant medication (methylphenidate and dexamphetamine sulphate) that no longer require medication are trialled off the medication for several weeks | NHMRC 2012(30) | consensus |  |  | ✔ |  | ✔ |  |  |  |  |  |  | ✔ |  | ✔ |  | Covered in another indicator.  Additionally, this is quite a difficult indicator to do, as the inclusion criteria are complicated and cover a reasonably long time period. So a surveyor will need to assess over time that the child previously trialled the environmental and family interventions and that they were not effective, before the meds were prescribed. |
| ADHD | | Children with ADHD have evidence of active involvement from at least two clinicians and/or service providers (teachers, parents, allied health etc). | Kohn, M 2008(32)  NHMRC 2012(30) | consensus |  | ✔ |  |  |  |  |  |  |  | ✔ |  |  |  |  | ✔ | Low score |
| AUTI | | Children being investigated for ASD have the following performed and recorded in the medical record:  - a comprehensive medical and family history AND  - a full physical examination | RACP 2008(33)  SIGN 2007(34) | consensus | ✔ |  |  |  | ✔ |  |  |  |  |  |  |  |  | ✔ |  | Merged with indicator 1 |
| AUTI | | Children suspected as having ASD are referred to appropriate clinicians for: - comprehensive assessment and management AND  - commenced treatment if diagnostic assessment is delayed | RACP 2008(33) | consensus |  | ✔ |  |  | ✔ |  |  |  |  |  |  |  |  | ✔ |  | Merged with indicator 3 |
| AUTI | | Children suspected of having ASD are screened using at least one of the following instruments:  - the modified checklist for autism in toddlers (M-CHAT) OR - a parent- completed checklist screening for autism for children aged 16–30 months OR  - the developmental behaviour checklist (DBC) OR - a parent-completed questionnaire of emotional and behavioural problems that includes an autism screening algorithm for children aged 4–18 years and also younger children aged 18–48 months. | RACP 2008(33) | consensus | ✔ |  |  |  | ✔ |  |  |  |  |  |  |  |  | ✔ |  | Covered in another indicator. |
| AUTI | | Children that test positive for ASD are referred for further assessment to any of the following:  - a paediatrician OR  - a child psychiatrist OR  - an autism assessment team | Tonge B, Brereton A 2011(35)  SIGN 2007(34) | consensus | ✔ |  |  |  |  | ✔ |  |  |  |  |  |  |  | ✔ |  | MERGED WITH PREVIOUS INDICATOR. |
| AUTI | | Children being assessed for ASD have evidence in the medical record that they had the following assessments:  - their behaviour, play and communication directly observed in a natural setting AND/OR  - had a video recording in a natural setting (school, childcare, family, peers) | Tonge B, Brereton A 2011(35)  SIGN 2007(34) | consensus | ✔ |  |  |  |  | ✔ |  |  |  |  |  |  |  | ✔ |  | Merged with previous indicator |
| AUTI | | Children of school age being assessed for ASD are referred to a psychologist for comprehensive psychometric and adaptive skills assessment | DOHA 2007(36)  RACP 2008(33)  SIGN 2007(34) | consensus | ✔ |  |  |  |  | ✔ |  |  |  |  |  |  |  | ✔ |  | Covered in another indicator. |
| AUTI | | Children being assessed for ASD have:  - a full general physical examination AND  - neurological examination AND  - hearing and vision assessment AND  - appropriate pathological investigations AND - genetic investigations (karotype and DNA for fragile X) | RACP 2008(33)  SIGN 2007(34) | consensus | ✔ |  |  |  |  | ✔ |  |  |  |  |  |  |  | ✔ |  | Merged with another indicator |
| AUTI | | Parents of children diagnosed with ASD are provided with:  - recommendations for intervention AND - referral to other agencies as required AND - a comprehensive summary of the assessment results and observations AND  - information about interventions, AND  - the opportunity to be included in goal setting, AND - the opportunity to discuss CAM | RACP 2008(33)  SIGN 2007(34) | consensus |  | ✔ |  |  |  | ✔ |  |  |  |  |  | ✔ |  |  |  | Unable to be determined form the medical record (unlikely to be documented) |
| AUTI | | Children diagnosed with ASD have an individual education intervention plan recorded in the medical record | DOHA 2007(36) | consensus |  | ✔ |  |  | ✔ |  |  |  |  |  |  | ✔ |  |  |  | Unlikely to be documented in the medical record (i.e. an agreement between parents and school). |
| AUTI | | Children diagnosed with ASD receive intervention therapy for at least 20 hours per week over an extended period of at least two years and then supported through school years | DOHA 2007(36) | consensus |  | ✔ |  |  | ✔ |  |  |  |  |  |  | ✔ |  |  |  | Not able to measure during medical record audit. |
| AUTI | | Children diagnosed with ASD with no other comorbid conditions are NOT prescribed medication to treat ASD | Tonge B, Brereton A 2011(35) | consensus |  | ✔ |  |  | ✔ |  |  |  |  |  | ✔ | ✔ |  |  |  | Too generalised (individual cases may be warranted), insufficient evidence. |
| AUTI | | Children diagnosed with ASD that have disruptive, aggressive and self-injurious behaviour that are prescribed resperidone have their weight monitored regularly | SIGN 2007(34) | Level B |  | ✔ |  |  | ✔ |  |  |  |  |  |  |  |  | ✔ |  | Covered in a previous indicator |
| AUTI | | Children diagnosed with ASD that have severe symptoms of inattention, impulsiveness and hyperactvity may be prescribed stimulant medication (methylphenidate) following a test dose to assess effect and close monitoring for side effects | Tonge B, Brereton A 2011(35)  SIGN 2007(34) | Level B |  |  | ✔ |  | ✔ |  |  |  |  |  | ✔ |  |  |  |  | Strength of original recommendation “may be” |
| AUTI | | Children diagnosed with ASD that have difficulty sleeping may be prescribed a sedative antihistamine or melatonin | Tonge B, Brereton A 2011(35)  SIGN 2007(34) | consensus |  |  | ✔ |  | ✔ |  |  |  |  |  | ✔ |  |  |  |  | Strength of original recommendation “may be” |
| AUTI | | Children diagnosed with ASD that are prescribed a sedative antihistamine or melatonin for sleeping difficulties have regular review to monitor side effects and treatment response | Tonge B, Brereton A 2011(35)  SIGN 2007(34) | consensus |  |  | ✔ |  | ✔ |  |  |  |  |  | ✔ |  |  |  |  | Lack of consensus re: timeframe |
| AUTI | | Children being assessed for ASD are referred to a speech pathologist for a language / communication assessment. | DOHA 2007(36)  RACP 2008(33)  SIGN 2007(34) | consensus |  | ✔ |  |  |  |  |  |  |  | ✔ |  |  |  | ✔ |  | Overlap with other indicators |
| AUTI | | Children being assessed for ASD with identifiable motor, play, sensory or self-care difficulties are referred to an Occupational Therapist. | RACP 2008(33)  SIGN 2007(34) | consensus |  | ✔ |  |  |  |  |  |  |  | ✔ |  |  |  | ✔ |  | Overlap with other indicators |
| AUTI | | Children diagnosed with ASD who had disruptive, aggressive and self-injurious behaviour were only prescribed risperidone by a paediatrician or a child psychiatrist. | Tonge B, Brereton A 2011(35)  SIGN 2007(34) | Level B |  | ✔ |  |  |  |  |  |  |  | ✔ |  |  |  |  | ✔ | Low score |
| CROU | | Children presenting with recurrent episodes of croup and any of the following:  - residual symptoms of stridor OR  - exercise limitation between episodes  are referred to a paediatric respiratory physician | Harrison J, Massie J. 2009(37) | consensus | ✔ |  |  |  | ✔ |  |  |  |  |  |  | ✔ |  |  |  | Think we would have trouble identifying this group as they are chronic rather than acute |
| CROU | | Children diagnosed with mild-moderate severity croup who were administered steroids and managed in the community received the following care:  -observed for 2-4 hours following administration of steroids AND - were discharged once stridor free at rest | RCH 2011(38) | consensus |  |  |  |  |  | ✔ |  |  |  |  | ✔ | ✔ |  |  |  | Feasibility/acceptability – GPs do not have the capacity to observe children for 2-4 hours. |
| CROU | | Children diagnosed with mild or moderate croup, presenting to the ED are assessed for all of the following:  - mental state at least hourly AND  - stridor at least hourly AND  - accessory muscle use, tracheal tug or chest wall retraction at least hourly AND  - respiratory rate at least hourly AND  - heart rate every 4 hours | HKSE 2007(39)  JOONDALUP 2006(40) | Level D |  |  |  |  |  | ✔ |  |  |  |  |  |  |  | ✔ |  | Covered in another indicator |
| CROU | | Children diagnosed with croup who are aged < 12 months of age are referred for a paediatric assessment. | HKSE 2007(39) | Level D |  |  |  |  |  | ✔ |  |  |  |  | ✔ | ✔ |  |  |  | Low evidence base, not feasible in some settings |
| CROU | | Children with croup who are administered nebulised adrenaline have observations performed as followed:  - initial observations - maximum of 5 minutes apart AND  - frequency decreasing as symptoms improve and clinical status stabilises. | HKSE 2007(39) | consensus |  |  |  |  |  | ✔ |  |  |  |  |  | ✔ |  | ✔ |  | Covered in another indicator; difficult to determine from medical record. |
| CROU | | Children with symptoms of severe or life threatening croup or with moderate or severe croup not responding to treatment have the following assessments performed and recorded every 15 minutes:  - mental state AND  - stridor AND  - accessory muscle use, tracheal tug or chest wall retraction AND  - respiratory rate AND - oxygen saturations | Harrison J, Massie J. 2009(37) | consensus |  |  |  |  |  | ✔ |  |  |  |  |  |  |  | ✔ |  | Lack of consensus re: timeframes / regularity; covered in another indicator |
| CROU | | Carers of children with croup are advised to call for an emergency ambulance if the child:  - becomes cyanosed OR  - is unusually sleepy OR  - is struggling to breathe. | NICE 2012(41) | consensus |  |  |  |  |  | ✔ |  |  |  |  |  | ✔ |  |  |  | Unlikely to be documented |
| CROU | | Carers of children with croup are advised to administer medication as follows to control fever and pain:  - paracetamol (15mg/kg 4 hourly up to 4 times per day, not exceeding 4 doses in 24 hours) OR  - ibubrofen (>6 months, 10mg/kg 3-4 times per day) | NICE 2012(41)  JOONDALUP 2006(40) | consensus |  |  |  |  | ✔ |  |  |  |  |  |  | ✔ |  |  |  | Unlikely to be documented |
| CROU | | Children presenting with croup should NOT routinely have their throat examined. | Alberta Medical Association 2008(42)  RCH 2011(38) | consensus |  |  |  |  |  |  |  | ✔ |  |  | ✔ |  |  |  |  | Low scores, relates to differential diagnosis for epiglottitis. |
| CROU | | Children presenting with mild croup are managed by a GP unless they have any of the following:  a significant relevant comorbidity or chronic illness OR  history of severe obstruction, or previous severe croup, or known structural upper airways abnormalities (e.g. laryngomalacia, tracheomalacia, vascular ring, Down's syndrome); OR  Age less than 6 months OR  Has inadequate fluid intake, or is refusing liquids OR  Re presentation to GP with the same illness (within 24 hours) OR  Looks toxic OR  Appears dehydrated, cyanosed, or exhausted OR  Has an uncertain diagnosis. | HKSE 2007(39) | Level D |  |  |  |  |  |  |  |  |  | ✔ |  | ✔ |  |  |  | Feasibility |
| CROU | | Children with croup who are administered nebulised adrenaline and show no improvement are managed as follows:  - administered a repeat dose of adrenaline AND  - if after 4 hours still have the following symptoms (significant respiratory compromise (after steroids) or stridor at rest) are admitted to hospital. | RCH 2011(38)  NSW CPG 2004(43)  Harrison J, Massie J. 2009(37) | consensus |  |  |  |  |  |  |  |  |  | ✔ |  | ✔ |  |  |  | Feasibility |
| DEPR | | Children are diagnosed with major depression if symptoms are consistent with the accepted diagnostic criteria of:  - DSM-IV-TR, OR  - ICD-10 | NHMRC 2011(44) | consensus |  |  |  |  |  | ✔ |  |  |  |  |  | ✔ |  |  |  | This has been excluded due to the original recommendation (good practice point) highlighting that diagnosis is based on clinical judgement (not feasible to measure/quantify via medical record audit). |
| DEPR | | Adolescents with depression, who are referred to mental health specialists from primary care, receive coordinated care that has been communicated between the mental health specialist and primary care practitioner. | Cheung et al 2007(45) | D/B/D |  |  |  |  |  | ✔ |  |  |  |  |  | ✔ |  |  |  | Feasibility – surveyors will not be able to obtain “full picture” for one patient. |
| DEPR | | Children and adolescents with moderate to severe depression are prescribed antidepressant medication only when:  - receiving concurrent psychological therapy, OR  - psychological therapy is not available or has been declined. | NICE 2005(46) | consensus |  |  |  |  |  |  |  |  |  | ✔ |  |  |  |  | ✔ | Low score |
| DEPR | | Adolescents with severe depression do NOT receive tricyclic antidepressants. | NHMRC 2011(44) | B |  |  |  |  |  |  |  |  |  | ✔ |  |  |  |  | ✔ | Low score |
| DIAB | | Children and adolescents diagnosed with type 1 diabetes are assessed by the diabetes team at each review for family functioning and diabetes related functioning including:  - communication AND - parental involvement and support AND - roles and responsibilities for self-care behaviours | Aust Diabetes Society 2011(47) | consensus |  |  |  |  |  |  |  |  |  |  |  | ✔ |  |  |  | Covered in other indicators; feasibility (unlikely to be documented) |
| DIAB | | Children and adolescents diagnosed with type 1 diabetes and history of any of the following have their school performance monitored: - developed diabetes before age 5-7 years OR - history of significant hypoglycaemic episodes OR - chronic poor blood glucose control | Aust Diabetes Society 2011(47) | consensus |  |  |  |  | ✔ |  |  |  |  |  |  | ✔ |  |  |  | Unlikely to be documented |
| DIAB | | Children and adolescents diagnosed with type 1 diabetes that are reported as experiencing significant learning difficulties are referred for: - psycho-educational examination OR - neuropsychological examination | Aust Diabetes Society 2011(47) | consensus |  |  |  |  | ✔ |  |  |  |  |  |  |  | ✔ |  |  | Low prevalence |
| DIAB | | Children and adolescents diagnosed with type 1 diabetes do NOT have real time continuous glucose monitoring routinely prescribed. | Aust Diabetes Society 2011(47) | Grade C |  |  |  |  | ✔ |  |  |  |  |  |  | ✔ |  |  |  | Unable to define exclusions |
| DIAB | | Children and adolescents with type 1 diabetes that are recommended the use of real time monitoring systems are provided with education in the correct use and interpretation of results | Aust Diabetes Society 2011(47) |  |  |  |  |  | ✔ |  |  |  |  |  |  | ✔ | ✔ |  |  | Low impact, feasibility |
| DIAB | | Children and adolescents with type 1 diabetes are NOT routinely prescribed metformin | Aust Diabetes Society 2011(47) | Grade C |  |  |  |  | ✔ |  |  |  |  |  |  |  | ✔ |  |  | Low prevalence (not routinely prescribed or TGA approved) |
| DIAB | | Children and adolescents with type 1 diabetes that are prescribed metformin have regular monitoring of Vitamin B12 levels | Aust Diabetes Society 2011(47) | consensus |  |  |  |  | ✔ |  |  |  |  |  |  | ✔ |  |  |  | Feasibility, definitional |
| DIAB | | Children and adolescents newly diagnosed with type 1 diabetes are:  - screened for coeliac disease | Aust Diabetes Society 2011(47) | Grade B | ✔ |  |  |  |  | ✔ |  |  |  |  |  |  |  | ✔ |  | Merged with another indicator |
| DIAB | | Children and adolescents newly diagnosed with type 1 diabetes are managed in an appropriate ambulatory or inpatient setting | Aust Diabetes Society 2011(47) | Grade B |  |  |  |  | ✔ |  |  |  |  |  |  | ✔ |  |  |  | Feasibility – difficult to determine |
| DIAB | | Children and adolescents newly diagnosed with type 1 diabetes that have any of the following are managed in an inpatient setting at the time of diagnosis:  - individuals with diabetic ketoacidosis OR - significant comorbidities OR - inadequate social support or mental health issues OR  - children under 2 years of age OR - those in geographically remote areas OR - non-English speakers | Aust Diabetes Society 2011(47) | consensus |  |  |  |  | ✔ |  |  |  |  |  |  | ✔ |  |  |  | Suspect there is good evidence for this and this is generally what happens  But I don’t think is feasible to assess and I suspect low impact. How do we find the ones that don’t make it to inpatient units? |
| DIAB | | Children and adolescents with type 1 diabetes that have a psychological assessment undertaken for a suspected mental health disorder are referred to a mental health specialist | Aust Diabetes Society 2011(47) | consensus |  |  |  |  | ✔ |  |  |  |  |  |  | ✔ |  |  |  | Difficult to assess |
| DIAB | | Children and adolescents with type 1 diabetes have routine assessment of their physical, intellectual, academic, emotional and social development recorded (timeframe) | Aust Diabetes Society 2011(47) | consensus |  |  |  |  | ✔ |  |  |  |  |  |  | ✔ |  |  |  | Too difficult to collect data |
| DIAB | | Children and adolescents with type 1 diabetes are not prescribed: - high protein/low carbohydrate diets OR - diets high in monounsaturated fats | Aust Diabetes Society 2011(47) | consensus |  |  |  |  | ✔ |  |  |  |  |  |  |  |  | ✔ |  | Covered in another indicator |
| DIAB | | Children and adolescents with type 1 diabetes who are:  - overweight or obese OR - at risk of overweight/obesity are advised to consume a diet lower in energy and fat | Aust Diabetes Society 2011(47) | consensus |  |  |  |  | ✔ |  |  |  |  |  |  | ✔ |  |  |  | Too hard to measure |
| DIAB | | Children and adolescents with diabetes have a comprehensive sick day management plan in their medical record that is regularly reviewed by the patient and diabetes health care team/provider | Aust Diabetes Society 2011(47) | consensus |  |  |  |  | ✔ |  |  |  |  |  |  | ✔ |  | ✔ |  | Not feasible, too hard to measure. Difficult to define “regular”  Combined with / covered in another indicator. |
| DIAB | | Children and adolescents with type 1 diabetes presenting with DKA are commenced initial rehydration therapy as follows: - rehydration with normal saline and potassium AND  - have fluid type adjusted according to ongoing Na, K and Glucose levels AND  - have fluid replacement continued for at least 6 hours AND  - are placed on normal saline with 5% dextrose and potassium if the blood glucose falls rapidly within the first few hours or if the BGL reaches 12-15 -mmol/l | RCH 2013(48) | consensus |  |  |  |  | ✔ |  |  |  |  |  |  |  |  | ✔ |  | Merged with another indicator |
| DIAB | | Children and adolescents with type 1 diabetes presenting with DKA are: - kept nil by mouth (except ice to suck) until alert and stable OR - have a nasogastric tube if the patient is comatose or has recurrent vomiting; leave on free drainage | RCH 2013(48) | consensus |  |  |  |  | ✔ |  |  |  |  |  |  |  | ✔ |  |  | Low impact |
| DIAB | | Children and adolescents with type 1 diabetes presenting with DKA and on fluid replacement are not given fluids with a tonicity of < 0.45% saline | RCH 2013(48) | consensus |  |  |  |  | ✔ |  |  |  |  |  | ✔ |  |  |  |  | Too specific |
| DIAB | | Children and adolescents with type 1 diabetes presenting with DKA that have been on fluid replacement for more than 6 hours are re commenced on 0.45% saline with 5% dextrose if the BGL is 12-15mmol/l provided none of the following are present: - hyponatremia OR - corrected serum sodium fails to stabilise or rise as the BGL decreases OR  - hyperosmolar and concern of rapid shifts in osmolarity | RCH 2013(48) | consensus |  |  |  |  | ✔ |  |  |  |  |  |  | ✔ |  |  |  | Difficult to measure |
| DIAB | | Children and adolescents with type 1 diabetes presenting with DKA have their BGL maintained between 5-12 mmol/l | RCH 2013(48) | consensus |  |  |  |  | ✔ |  |  |  |  |  |  | ✔ |  |  |  | Difficult to measure |
| DIAB | | Children and adolescents with type 1 diabetes presenting with DKA that require rehydration may have it completed orally after the first 24-36 hours if they are metabolically stable (coincides with insulin infusion changing to s/c) | RCH 2013(48) | consensus |  |  |  |  | ✔ |  |  |  |  |  |  | ✔ | ✔ |  |  | Low impact, feasibility |
| DIAB | | Children and adolescents with type 1 diabetes presenting with DKA are commenced on insulin infusion as per the following rates: - 0.1 units/kg/hr in newly diagnosed children, and those with established diabetes who have glucose levels > 15 mmol/l OR  - 0.05 units/kg/hour for children with established diabetes who have had their usual insulin and whose blood glucose level is < 15 mmol/l. | RCH 2013(48) | consensus |  |  |  |  | ✔ |  |  |  |  |  |  | ✔ |  |  |  | Difficult to measure |
| DIAB | | Children and adolescents with type 1 diabetes presenting with DKA have insulin infusion discontinued when they are alert and metabolically stable (pH . 7.30 and HCO3 >15) | RCH 2013(48) | consensus |  |  |  |  | ✔ |  |  |  |  |  |  | ✔ |  |  |  | Difficult to measure |
| DIAB | | Children and adolescents with type 1 diabetes presenting with DKA have the following observations performed and recorded in the medical record (more frequent if unstable): - pulse, BP and respiratory rate at least hourly AND - level of consciousness (GCS) at least hourly AND - neurological status (pupillary responses, assess for change e.g. restlessness, irritability, headache) at lea44st hourly AND - glucose and ketones measurements while on insulin infusion hourly AND - venous blood gas and lab glucose at least 2 hourly for initial 6 hours; then 2 - 4 hourly thereafter AND - Serum urea and electrolytes monitored 2 - 4 hourly for the initial 12 - 24 hours (and as clinically indicated thereafter) AND - temperature measured every 2-4 hours AND - nursed with their head elevated | RCH 2013(48) | consensus |  |  |  |  | ✔ |  |  |  |  |  |  | ✔ |  |  |  | Difficult to measure |
| DIAB | | Children and adolescents with type 1 diabetes presenting with DKA are only administered HCO3 if they meet the following criteria: - extremely sick and rare circumstances (e.g if pH < 7.0 +/-HCO3 < 5mmol/l and they require adrenaline for BP support or have marked hyperkalaemia) AND  - only after consultation with consultant endocrinologist | RCH 2013(48) | consensus |  |  |  |  | ✔ |  |  |  |  |  |  | ✔ |  |  |  | Difficult to measure |
| DIAB | | Children and adolescents with type 1 diabetes presenting with DKA whose blood glucose falls below 4.0mmol/l that are still acidotic are given:  - 10% dextrose IV bolus (2-5ml/kg) AND - have 10% dextrose as ongoing fluids AND  - the insulin infusion continued | RCH 2013(48) | consensus |  |  |  |  | ✔ |  |  |  |  |  |  | ✔ |  |  |  | Difficult to measure |
| DIAB | | Children and adolescents with type 1 diabetes presenting with DKA who have hypoglycaemia despite use of 10% dextrose in preceding 2 hours are managed as follows:  - has the rate of insulin infusion decreased to 0.05U/kg/hr as long as ketosis and acidosis are clearing AND  - continues 10% dextrose concentration in IV fluids until BGL stable | RCH 2013(48) | consensus |  |  |  |  | ✔ |  |  |  |  |  |  |  | ✔ |  |  | Low prevalence |
| DIAB | | Children and adolescents with type 1 diabetes presenting with DKA and suspected cerebral oedema have: - mannitol 20% 0.5g/kg (range 0.25 - 1.0g/kg) IV over 20 minutes as soon as the clinical diagnosis is suspected AND  - repeat dose of mannitol 20% 0.5g/kg (range 0.25 - 1.0g/kg) IV over 20 minutes if there is no change after 30 minutes  - 2 hours AND - reduced fluid input by one third AND  - nursed with head elevated AND - transferred to ICU level care | RCH 2013(48) | consensus |  |  |  |  | ✔ |  |  |  |  |  |  | ✔ |  |  |  | Difficult to measure |
| DIAB | | Children and adolescents with type 1 diabetes presenting with DKA have their case discussed with an endocrinologist at earliest opportunity | RCH 2013(48) | consensus |  |  |  |  | ✔ |  |  |  |  |  |  | ✔ |  |  |  | Difficult to measure |
| DIAB | | Children and adolescents that present with a new diagnosis of type 1 diabetes that are:  - mildly ill (<3% dehydration, no acidosis and not vomiting) AND  - have a BGL > 11.1mmol/l have their blood ketones checked. | RCH 2013(48) | consensus |  |  |  |  | ✔ |  |  |  |  |  |  |  |  | ✔ |  | Covered in another indicator |
| DIAB | | Children and adolescents newly diagnosed with type 1 diabetes are administered 0.25 units/kg (half dose if <= 4 years old or ketotic) of quick acting insulin stat (if within 2 hr prior to meal delay and give mealtime dose) | RCH 2013(48) | consensus |  |  |  |  | ✔ |  |  |  |  |  | ✔ |  |  |  |  | Too specific – affected by local protocols |
| DIAB | | Children and adolescents newly diagnosed with type 1 diabetes are commenced on one of the two following standard insulin regimes: - Twice daily injections of a mixture of short and intermediate-acting insulins OR  - Multiple daily injections (MDI) of insulin | RCH 2013(48) | consensus |  |  |  |  | ✔ |  |  |  |  |  |  | ✔ |  |  |  | Difficult to measure |
| DIAB | | Children and adolescents newly diagnosed with type 1 diabetes are admitted for:  - commencement of insulin AND - diabetic education | RCH 2013(48) | consensus |  |  |  |  | ✔ |  |  |  |  |  |  | ✔ |  |  |  | Difficult to measure |
| DIAB | | Children and adolescents with type 1 diabetes have the following individual risk factors for hypoglycaemia documented:  - low HbA1c less than 7.5%, AND  - seizures. | Aust Diabetes Society 2011(47) | Grade B |  |  |  |  |  |  |  | ✔ |  |  | ✔ |  |  |  |  | Low scores |
| DIAB | | Children and adolescents with type 1 diabetes receive structured education at diagnosis, (self-study material, video programs and blood glucose awareness training) targeting the specific prevention of severe hypoglycaemia. | Aust Diabetes Society 2011(47) | consensus |  |  |  |  |  |  |  | ✔ |  |  |  | ✔ |  |  |  |  |
| DIAB | | Children and adolescents with type 1 diabetes presenting with signs of DKA, who test positive for blood ketones (greater than 0.6mmol/l) are assessed for acidosis by HCO3 measurement. | RCH 2013(48) | consensus |  |  |  |  |  |  |  | ✔ |  |  |  |  |  | ✔ |  | Covered in another indicator |
| DIAB  *SUGGESTED INDICATOR* | | "Children and adolescents with Type 1 diabetes receive immunofluorescence testing at diagnosis for:  - islet-antigen 2 (IA2) autoantibodies AND"  - islet cells autoantibodies (ICA). | External reviewer did not provide reference | External reviewer did not provide reference |  |  |  |  | ✔ |  |  |  |  |  | ✔ |  |  |  |  | Not essential care |
| DIAB | | Adolescents with type 1 diabetes are assessed at least annually for:  - alcohol intake AND  - smoking AND  - recreational drug use. | Aust Diabetes Society 2011(47) | consensus |  |  |  |  |  |  |  |  |  | ✔ |  |  |  |  | ✔ | Low score |
| ECZE | | Children with atopic eczema that present with a flare (acute deterioration) have nasal swabs taken and if positive for staph aureus are prescribed intranasal bactroban | RCH - Melbourne 2013(49) | consensus |  |  |  |  | ✔ |  |  |  |  |  | ✔ |  |  |  |  | Poor evidence base |
| ECZE | | Children with atopic eczema that present with a flare (acute deterioration) that have areas that are oozing have:  - the location of oozing documented AND  - a swab of the area taken | The Childrens Hospital at Westmead - 2012 | consensus |  |  |  |  | ✔ |  |  |  |  |  |  |  |  | ✔ |  | Covered in another indicator |
| ECZE | | Children with atopic eczema that are admitted to hospital have a 2-3 minute daily bath with an additive (QV oil) as ordered by the medical officer | The Childrens Hospital at Westmead - 2012 | consensus |  |  |  |  | ✔ |  |  |  |  |  |  | ✔ |  |  |  | Unlikely to be documented |
| ECZE | | Children with atopic eczema that are admitted to hospital with impetigo are nursed in isolation until the blisters/lesions have dried | The Childrens Hospital at Westmead - 2012 | consensus |  |  |  |  | ✔ |  |  |  |  |  |  | ✔ |  |  |  | Unlikely to be documented |
| ECZE | | Children with atopic eczema with a herpes simplex virus 1 infection:  - are commenced on oral aciclivor within 48 hours of symptom onset, AND  - if unwell, febrile and have threatened eye involvement are referred to ophthalmology, AND - have a swab taken in viral medium to confirm infection AND - have urgent referral to dermatologist or paediatrician if widespread | RCH - Melbourne 2013(49) | consensus |  |  |  |  | ✔ |  |  |  |  |  |  | ✔ | ✔ |  |  | Definitional, small sample size |
| ECZE | | Children with atopic eczema and any of the following are referred for allergy skin prick or RAST testing: - history of flushing, itch, urticaria or general flare of the eczema after ingestion of food OR  - Itchy child (<12 months) with moderate to severe eczema and not improving with treatment OR  - Child compliant with adequate treatment regime for greater than 6 weeks with no improvement in eczema OR  - Eczema lesions are in the periorbital and exposed areas such as arms and legs, may indicate environmental allergy (pollen, dust mite) | RCH - Melbourne 2013(49) | GPP | ✔ |  |  |  |  | ✔ |  |  |  |  |  | ✔ |  |  |  | Difficult to be objective and easily determine this information from the medical record |
| ECZE | | Children who present with atopic eczema and have food allergies confirmed following skin prick testing are referred to a dietician | RCH Melbourne 2013(49)  SIGN 2011(50) | consensus |  |  |  |  |  | ✔ |  |  |  |  | ✔ |  | ✔ |  |  | Low evidence, low prevalence |
| ECZE | | Children with atopic eczema that were admitted to hospital with a flare (acute deterioration) received follow up care as follows: - **inpatient in hospital** or attended eczema workshop in hospital seen in outpatients in 2 - 4 weeks OR - **mild eczema** are advised to receive follow up care with their GP OR - **moderate eczema** are reviewed in outpatients in two to four weeks and if improved to mild advised to receive future follow up with their GP OR  - **severe eczema** are reviewed in outpatients in one to two weeks | RCH - Melbourne 2013(49) | consensus |  |  |  |  | ✔ |  |  |  |  |  |  | ✔ |  |  |  | Too complex |
| ECZE | | Parents of children with atopic eczema are provided with the following information: - Avoid environmental aggravators – over-heating, rough prickly materials, regular and ongoing use of emollients - Follow and institute the treatment plan for excellent management and eczema improvement - Begin flaring treatment as soon as the flare begins and cease flaring treatment until needed again  - Wet dressings are essential in controlling a flare and promoting sleep and should be applied if the other treatments have not cleared the eczema within 24- 48 hours | RCH - Melbourne 2013(49) | consensus |  |  |  |  |  | ✔ |  |  |  |  |  | ✔ |  |  |  | Unlikely to be documented |
| ECZE | | Children diagnosed with atopic eczema have their allergy history documented. | The Childrens Hospital at Westmead - 2012 | consensus |  |  |  |  |  |  |  |  |  | ✔ |  |  |  |  | ✔ | Low score |
| ECZE | | Children who presented with atopic eczema and were prescribed topical steroids were advised to use emollient cream. |  |  |  |  |  |  |  |  |  |  |  | ✔ |  |  |  |  | ✔ | Low score |
| FEVE | | Children aged 0-5 years presenting with fever have their temperature measured and documented via the axilla or infrared tympanic thermometer | NICE 2014(51)  NSW Health 2013 | Level IV  Level I |  |  |  |  | ✔ |  |  |  |  |  |  | ✔ |  |  |  | Unlikely to be documented |
| FEVE | | All children presenting with fever are assessed for ABCD signs of toxicity:  - A for arousal, alertness or activity decreased AND  - B for breathing difficulties (tachypnoea, increased work of breathing) AND  - C for poor colour (pale or mottled), poor circulation (cold peripheries, increased capillary refill time) or cry (weak, high pitched) AND  - D for decreased fluid intake (less than half normal) and/or decreased urine output (fewer than four wet nappies a day) | NSW Health 2010(52) | level II |  |  |  |  | ✔ |  |  |  |  |  |  |  |  | ✔ |  | Covered in other indicators |
| FEVE | | Children presenting with fever are NOT given tepid sponges or other physical interventions to reduce fever. | NSW Health 2010(52) | consensus |  |  |  |  | ✔ |  |  |  |  |  |  | ✔ | ✔ |  |  | Low impact, unlikely to be documented |
| FEVE | | Children with chronic medical conditions presenting with fever have their regular specialist team or paediatrician contacted | SA Child Health Clinical Network 2013(53) | consensus |  |  |  |  | ✔ |  |  |  |  |  | ✔ |  | ✔ |  |  | Low prevalence, may not be standard care |
| FEVE | | Children aged 3 months to 3 years with a temperature > 38 degrees who have a clear source of infection and appear well are treated as clinically indicated | RCH 2011(54) | consensus |  | ✔ |  |  | ✔ |  |  |  |  |  |  | ✔ |  |  |  | Treatment will depend on the source of infection – will be too difficult to determine this information form the medical record (objectively and reliably) |
| FEVE | | Children aged 3 months to 3 years with a temperature > 38 degrees that have a clear source of infection that are unwell are: - reviewed by senior registrar/consultant AND  - investigated for appropriate clinical focus AND  - admitted for treatment | RCH 2011(54) | consensus |  |  |  |  | ✔ |  |  |  |  |  |  | ✔ |  |  |  | Too difficult to measure , definitional |
| FEVE | | Infants aged 1-3 months presenting with fever that are shocked, unrousable or showing signs of meningococcal disease are managed as follows:  -urgently reviewed by paediatrician AND  - referred to paediatric ICU after commencement of appropriate antibiotics | SA Child Health Clinical Network 2013(53) | consensus |  |  |  |  |  | ✔ |  |  |  |  |  |  |  | ✔ |  | Merged with other indicators |
| FEVE | | Children aged 3 months to 3 years presenting with fever (over 38oC) who are toxic are managed as follows:  - admitted to hospital OR  - transferred to an appropriate facility | SA Child Health Clinical Network 2013(53) | consensus |  |  |  |  |  | ✔ |  |  |  |  | ✔ | ✔ |  |  |  | May not be a discriminator of appropriate practice, feasibility issues (i.e. define “appropriate” facility) |
| FEVE | | All children presenting with fever and any of the following symptoms are assessed and investigated and treated for meningococcal disease:  - pre-existing coryzal illness OR  - gastrointestinal symptoms with no rash OR  - blanching non purpuric rash OR  - leg pain cold extremities and abnormal skin colour OR - serial signs of toxicity | NSW Health 2010(52) | consensus |  |  |  |  | ✔ |  |  |  |  |  | ✔ |  |  |  |  | Coryzal and GI symptoms are common and not, on their own, suggestive of meningococcal disease. |
| FEVE | | All children presenting with any of the following symptoms are assessed and investigated for kawasaki disease:  - high fever > 5 days OR  - conjunctival infection OR  - polymorphous rash OR  - changes in mucous membranes OR - changes in the extremities and OR cervical lymphadenopathy OR - neutrophilia with toxic changes OR  - thrombocytosis OR  - raised acute phase reactants OR  - elevated transaminases OR  - low serum albumin | NSW Health 2010(52) | consensus |  |  |  |  |  | ✔ |  |  |  |  |  | ✔ | ✔ |  |  | Low prevalence, too complicated |
| FEVE | | All febrile children that are not distressed are NOT routinely administered paracetamol | SA Child Health Clinical Network 2013(53) | consensus |  |  |  |  | ✔ |  |  |  |  |  |  | ✔ |  |  |  | Too hard to measure |
| FEVE | | Children aged 3 months to 3 years presenting with fever (over 38oC) are not administered paracetamol and ibuprofen simultaneously | SA Child Health Clinical Network 2013(53) | consensus |  |  |  |  |  | ✔ |  |  |  |  |  |  | ✔ |  |  | Low impact |
| FEVE | | All Infants aged 1-3 months presenting with fever greater than 38^o^C have the following investigations performed:  - CBE AND  - Blood culture AND  - Urine culture (SPA/Catheter) AND - CXR (if respiratory symptoms and signs) AND - +/- lumbar puncture | RCH 2011(54) | consensus |  |  |  |  | ✔ |  |  |  |  |  |  |  |  | ✔ |  | Merged with another indicator (covered) |
| FEVE | | Children aged 3 months to 3 years with a temperature >38oC and no clear focus of infection that  look unwell,  are younger than 6 months  have immunocompromised  OR are not fully immunised have:  - Full sepsis workup: FBE, blood culture, urine culture ± CXR (if respiratory symptoms or signs) ± LP AND - Admitted to hospital for observation off antibiotics OR - considered for discharge with antibiotics | RCH 2011(54) | consensus |  |  |  |  | ✔ |  |  |  |  |  |  | ✔ |  |  |  |  |
| FEVE | | Children presenting without a focus of infection have a urinalysis performed and are managed as follows: - If positive - commenced on antibiotics OR - If negative - reviewed the next day if still febrile | NSW Health 2010(52) | consensus |  |  |  |  | ✔ |  |  |  |  |  | ✔ |  |  |  |  | Lack of consensus re: age cutoffs |
| FEVE | | Children aged 3 months to 3 years with a temperature >38^o^C, no clear focus of infection that look miserable but are still relatively alert, interactive and responsive are managed as follows:  - If < 12 months boys or <2 yrs girls -urine, can do SPA up to 12 months of age OR  - If > 12 months - Consider Urine m,c,s AND  - Discuss with registrar or consultant prior to any investigations | RCH 2011(54) | consensus |  |  |  |  | ✔ |  |  |  |  |  |  |  |  | ✔ |  | Covered in another indicator |
| FEVE | | All children presenting with fever who have the following chest symptoms or signs of respiratory illness - cough OR  - tachypnoea OR  - dullness or crackles  Have a chest x-ray | NSW Health 2010(52) | consensus |  |  |  |  | ✔ |  |  |  |  |  | ✔ |  |  |  |  | Chest XR not indicated in all cases (acceptability) |
| FEVE | | All children presenting with fever who do not have respiratory signs have other investigations performed before having a chest x-ray. | NSW Health 2010(52) | consensus |  |  |  |  | ✔ |  |  |  |  |  |  |  |  | ✔ |  | Covered in other indicators |
| FEVE | | Infants aged > 4 months (i.e. aged 4 months or more) presenting with fever and where meningitis has been excluded are commenced on antibiotics (pending blood culture results ie up to 48hours) as follows:  -cefotaxime 25mg/kg (up to 1 g) IV 6 hourly OR  - ceftriaxone 25 mg/kg (up to 1 g) IV daily AND flucloxacillin 50mg/kg (up to 2g) IV 6 hourly | Cincinatti Childrens Hospital 2010(55) | consensus |  |  |  |  |  | ✔ |  |  |  |  |  | ✔ |  |  |  | Too complicated |
| FEVE | | Infants aged < 6 months presenting with fever where meningitis has NOT been excluded are commenced on antibiotics (pending blood culture results) as follows:  - amoxicillin OR ampicillin 50mg/kg IV 6 hourly AND  - cefotaxime 50 mg/kg IV 6 hourly AND  - vancomycin 30 mg/kg IV 12 hourly (if pneumococcal meningitis is likely) | Cincinatti Childrens Hospital 2010(55) | consensus |  |  |  |  |  | ✔ |  |  |  |  |  | ✔ |  |  |  | Difficult to determine from the notes (i.e. where meningitis has NOT been excluded) |
| FEVE | | Infants aged > 6 months (i.e. aged 6 months or more) presenting with fever where meningitis has NOT been excluded are commenced on antibiotics (pending blood culture results) as follows:  - cefotaxime 50mg/kg (up to 2g) IV 6 hourly, OR  - ceftriaxone 100 100mg/kg (up to 4g) IV daily OR  - ceftriaxone 50mg/kg (up to 2g) IV 6 hourly  AND  - flucloxacillin 50mg/kg (up to 2g) IV 6 hourly  (if pneumococcal meningitis is likely, also add: vancomycin 30mg/kg up to 1.5g IV 12 hourly) | Cincinatti Childrens Hospital 2010(55) | consensus |  |  |  |  |  | ✔ |  |  |  |  |  |  |  |  |  | Difficult to determine from the notes (i.e. where meningitis has NOT been excluded) |
| FEVE | | Infants aged < 1 month who present with fever in the community that have difficult IV access have a single intramuscular dose of ampicillin and cefotaxime as follows: - after blood cultures obtained AND  - prior to transfer | SA Child Health Clinical Network 2013(53) | consensus |  |  |  |  |  | ✔ |  |  |  |  | ✔ | ✔ |  |  |  | Acceptability.  Expectation of blood culture and parenteral antibiotic probably not reasonable.  If hospital is close and/or baby looks well, transfer without treatment is OK.  GPs will not attempt IV access. Prompt referral is more important. Blood cultures will usually not be obtained when IV access is difficult. |
| FEVE | | Infants aged 1 -3 months presenting with fever are treated with antibiotics as follows:  - **IF CSF negative** - Ampicillin 25mg/kg/dose IV 6 hourly PLUS Gentamicin 8mg/kg IV daily (Gent levels monitored according to local guidelines). (*A 3rd generation cephalosporin is recommended instead of gentamicin in some literature and may be considered in some circumstances). NB add flucloxacillin 25-50 mg/kg/dose IV 6 hourly if staphyloccocal infection is suspected - e.g. broken skin OR - **If CSF positive or unknown** (bloody tap, child too sick for LP etc) - Ampicillin 25mg/kg/dose IV 6 hourly PLUS Cefotaxime 50mg/kg IV 6 hourly. | SA Child Health Clinical Network 2013(53) | consensus |  |  |  |  |  | ✔ |  |  |  |  |  |  |  | ✔ |  | Merged with another indicator |
| FEVE | | Children aged 3-36 months presenting with fever >38^o^C that are toxic are commenced on antibiotics as follows: - **If CSF negative** - Ceftriaxone 50mg/kg IV daily PLUS Flucloxacillin* 25-50 mg/kg/dose IV 6 hourly (*consider vancomycin if high prevalance/risk MRSA) OR - **If CSF Positive or unknown** (bloody tap, child too sick for LP etc) Ceftriaxone 100mg/kg IV daily PLUS Flucloxacillin 25-50 mg/kg/dose 6 hourly. Consider adding vancomycin 30mg/kg IV 12 hourly if meningitis suspected (to cover pneumococcus) | SA Child Health Clinical Network 2013(53) | consensus |  |  |  |  |  | ✔ |  |  |  |  |  |  |  | ✔ |  | Merged with another indicator |
| FEVE | | Children who present with fever and are discharged but return a positive blood culture have their family contacted immediately to arrange clinical review | RCH 2011(54) | consensus |  |  |  |  |  | ✔ |  |  |  |  | ✔ |  | ✔ |  |  | Adequate sample unlikely  This is management of positive blood culture/possible sepsis rather than fever. |
| FEVE | | Children that presented with fever that were discharged but return a blood culture positive for strep pneumoniae are managed as follows:  - if well, afebrile and already on antibiotics then complete a 7 day course of antibiotics OR  - If they have not received antibiotics and they are well they are not administered antibiotics OR  - If clinically deteriorated or unwell and febrile - complete sepsis workup and admitted for IV antibiotics. | RCH 2011(54) | consensus |  |  |  |  |  | ✔ |  |  |  |  | ✔ |  |  |  |  | Beyond scope of fever  This is a focussed treatment and not just about fever?? |
| FEVE | | Children that presented with fever that were discharged but return a blood culture positive for any other organism (regardless of clinical condition)  - Discuss with ED Consultant (or General Paediatric Consultant on call if patient is admitted) even if isolate is thought to be a contaminant AND  - Sepsis workup and admission for IV antibiotics to be discussed and considered | RCH 2011(54) | consensus |  |  |  |  |  | ✔ |  |  |  |  |  |  |  | ✔ |  | Covered in another indicator |
| FEVE | | All children presenting with fever are reviewed by the senior medical officer (especially if referred by GP or representing with febrile illness) prior to discharge | NSW Health 2010(52) | consensus |  |  |  |  |  | ✔ |  |  |  |  |  | ✔ |  |  |  | The definition of “senior” will vary according to resources. This indicator is in no way specific to fever.  Does this mean consultant?  Registrar review reasonable and therefore may not be the most senior MO. |
| FEVE | | Infants aged 1-3 months presenting with fever may be discharged provided the following criteria are met: - considered low risk (appears well, WCC 5000 - 15000) AND - all investigations are normal AND  - reviewed by senior doctor AND  - Follow up in 12 hours arranged AND  - parents advised to return if child deteriorates AND  - parents provided with fever information brochure | SA Child Health Clinical Network 2013(53)  RCH 2011(54) | consensus |  |  |  |  |  | ✔ |  |  |  |  |  |  |  | ✔ |  | Covered in another indicator |
| FEVE | | Infants aged less than 4 months presenting with fever (greater than 38 degrees Celsius) where meningitis has been excluded are commenced on antibiotics (pending blood culture results) as follows: amoxicillin OR ampicillin AND gentamicin. | SA Child Health Clinical Network 2013(53) | consensus |  |  |  |  |  |  |  | ✔ |  |  | ✔ |  |  |  |  | Antibiotics at 1-3 months are not mandatory |
| GORD | | Children presenting with dysphagia or odynophagia are NOT prescribed acid suppression therapy without having diagnostic evaluation. | Vandenplas (NASPGHAN) 2009(56) | consensus |  |  |  |  |  |  |  |  |  | ✔ |  |  |  |  | ✔ | Low score |
| GORD | | Infants (aged less than 12 months) with recurrent regurgitation and poor weight gain despite adequate energy intake are provided with a 2 to 6 week trial of extensively hydrolyzed protein formula, thickened formula, increased caloric density formula or NG/NJ feeding. | Vandenplas (NASPGHAN) 2009(56)  Bhavsar 2011(57) | B (extensively hydrolyzed protein formula)  A (thickened formula) |  |  |  |  |  |  |  |  |  | ✔ |  |  |  |  | ✔ | Low score |
| GORD | | Children with GORD were prescribed the following medication:  - Infant Gaviscon (dual sachet = 2 doses, added to formula or spoon after feed) less than 4.5kg one dose, greater than 4.5kg 2 doses, max. six times per day OR  - Mylanta 0.5-1ml/kg 3-4 times per day OR  - Ranitidine 2-3mg/kg 2-3 times per day before meals OR  - Omeperazole trial for 2 to 4 weeks less than 10kg (5mg once daily), 10-20kg (10mg once daily, max. 20mg/day), greater than 20kg (20mg once daily, max. 40mg/day). | RCH Melb 2012(58)  RCH paed handbook 2009(4)  Bhavsar 2011(57)  QLD Health 2011 | consensus |  |  |  |  |  |  |  |  |  | ✔ |  |  |  |  | ✔ | Low score |
| GORD | | Infants / Children (aged less than 18 months) with reflux oesophagitis and any of the following are referred to a paediatric gastroenterologist:  - receiving PPI therapy for 3 months and have persistent / recurrent symptoms OR  - are unable to have their medication ceased at each review (PPI dependency). | Vandenplas (NASPGHAN) 2009(56) | A  D (continue PPI and referral to GI specialist) |  |  |  |  |  |  |  |  |  | ✔ |  |  | ✔ |  |  | > 3 inclusion criteria |
| HEAD | | Children presenting with minor/mild head injury are observed in the ED hourly until discharge which includes:  - conscious state, AND  - pulse rate, AND  - respiratory rate, AND  - blood pressure, AND  - pupil assessment, AND  - limb strength | RCH Melb(59) | concensus |  |  |  |  |  | ✔ |  |  |  |  | ✔ |  |  |  |  | Contradicts previous indicator |
| HEAD | | Children presenting with a head injury have oxygen saturations recorded and maintained at ≥ 95% at all times. | NSW Health 2010(60) | consensus |  |  |  |  |  |  |  |  |  |  |  |  |  |  |  |  |
| HEAD | | Children presenting with a head injury who have signs of shock and are not intubated receive 10 litres of oxygen via a non-rebreather mask regardless of oxygen saturation readings. | NSW Health 2010(60) | consensus |  |  |  |  |  | ✔ |  |  |  |  |  |  | ✔ |  |  | Low prevalence |
| HEAD | | Children presenting with a head injury who have focal neurological signs or basal skull fracture (signs or suspicion of) receive a:  - Head CT AND  - review by a neurosurgeon | RCH Melb(59) | consensus |  |  |  |  |  | ✔ |  |  |  |  |  | ✔ |  |  |  | Difficult to determine from the medical record |
| HEAD | | Children presenting with a head injury and show signs of shock are prescribed Normal Saline 20mL/kg (IV or IO) | NSW Health 2010(60) | consensus |  |  |  |  |  | ✔ |  |  |  |  | ✔ |  |  |  |  | Not specific for Head injury (shock) |
| HEAD | | Infants presenting with a head injury and are nil by mouth receive 4 hourly blood glucose monitoring. | NSW Health 2010(60) | consensus |  |  |  |  |  | ✔ |  |  |  |  |  |  | ✔ |  |  | Low prevalence |
| HEAD | | Children presenting with head injury receive blood tests for all of the following:  - full blood count  - electrolytes  - basic clotting studies  - liver enzymes  - amylase | NSW Health 2010(60) | consensus |  |  |  |  |  | ✔ |  |  |  |  | ✔ |  |  |  |  | Acceptability  Exclude. This is not a general trauma indicator. These tests are unnecessary in the vast majority of children with head injuries. |
| OBES | | Children with a BMI ≥ 85th percentile have their weight, height, BMI calculated and documented every three months | NHMRC 2013(61) | NHMRC Practice point |  |  |  |  |  | ✔ |  |  |  |  | ✔ |  |  |  |  | Original recommendation (“may”) Strength of recommendations |
| OBES | | Children with a BMI ≥ 85th percentile are screened for the following and provided with help to deal with the issue if any exist : - disordered eating AND  -poor body image AND  -depression and anxiety AND  - weight related bullying | NHMRC 2013(61) | NHMRC Practice point |  |  |  |  | ✔ |  |  |  |  |  |  | ✔ |  |  |  | Difficult to assess in audit |
| OBES | | Children aged < 12 years with a BMI ≥ 85th percentile, who do NOT have a severe life-threatening comorbidity (sleep apnoea, raised ICP) or insulin resistance are NOT prescribed drug therapy | NICE 2006(62) | consensus |  |  |  |  |  | ✔ |  |  |  |  |  | ✔ | ✔ |  |  | Low impact, too difficult to assess. |
| OBES | | Children with a BMI ≥ 85th percentile who are prescribed Orlistat are managed as follows:  - Orlistat trialled for 6-12 months AND  - placed on low fat diet AND  - monitored for effectiveness, adherence and adverse effects. | NICE 2006(62)  Hearnshaw 2010(63) | consensus |  |  |  |  | ✔ |  |  |  |  |  | ✔ | ✔ |  |  |  | Not routine care, unlikely in GP setting.  Feasibility / acceptability |
| OBES | | Adolescents who underwent bariatric surgery AND also had a binge eating disorder received psychological support post-surgery | Baur 2010(64) | consensus |  |  |  |  | ✔ |  |  |  |  |  |  |  | ✔ |  |  | Low prevalence |
| OBES | | Children who are pre-pubescent should NOT have bariatric surgery | Endocrine 2008(65) | Endocrine 1/++00 |  |  |  |  | ✔ |  |  |  |  |  |  |  | ✔ |  |  | Low prevalence |
| OTIT | | Children who do not have a middle ear effusion are NOT diagnosed with AOM | Lieberthal 2013(66) | Lieberthal B/Recommendation |  |  |  |  | ✔ |  |  |  |  |  |  |  | ✔ |  |  | Low impact |
| OTIT | | Children with AOM referred to an ENT specialist may be considered for a myringotomy and insertion of tympanostomy tubes if: - ≥3 episodes of AOM in 6mths or ≥4 episodes in 12months - retracted tympanic membrane - significant symptoms are present (recurrent AOM or functionally significant hearing loss which is resulting in speech delay, behavioural disturbance or poor school performance) | NSW Health 2005(67)  RCH paed handbook 2009(4) | consensus |  |  |  |  | ✔ |  |  |  |  |  |  |  |  | ✔ |  | Merged with another indicator (overlap) |
| OTIT | | Parents of children diagnosed with AOM are provided with the following advice: - presence of fluid in the middle ear may exist for a few weeks and clear up by itself over a couple of months - the child may have some trouble hearing during this time - any increase in irritability or hearing loss seek medical advice - symptoms persist for >3months seek medical advice | NSW Health 2005(67) | consensus |  |  |  |  | ✔ |  |  |  |  |  |  | ✔ |  |  |  | Unlikely to be documented |
| OTIT | | Children presenting with signs and symptoms of AOM that have any of the following are diagnosed with Otitis media with effusion:  - afebrile OR  - tympanic membrane not red OR  - tympanic membrane not bulging OR  - asymptomatic | Gunasekera 2008(68) | consensus |  |  |  |  | ✔ |  |  |  |  |  | ✔ |  |  |  |  | Low acceptability |
| OTIT | | Children presenting with the following signs and symptoms are diagnosed with Acute Otitis Media (AOM):  red tympanic membrane OR bulging tympanic membrane OR reduced tympanic membrane motility AND  new onset of otorrhea not due to acute otitis externa AND  fever AND  ear pain (irritability, holding, tugging, or rubbing of ear) | RCH paed handbook 2009(4)    RCH Melb 2012(69)  Gunasekera 2008(68) | consensus |  |  |  |  |  |  |  |  |  | ✔ |  | ✔ |  |  |  | Feasibility – all records will be pulled by diagnostic code therefore not applicable |
| PREV | | At the time of birth, parents of neonates received advice and counselling on injury prevention including: - accidental and non-accidental injury AND  - passive smoking AND  - SIDS AND  - sun exposure AND  - settling AND  - maternal health | RACGP 2013(70) | Level III; Grade B  GPP |  |  |  |  | ✔ |  |  |  |  |  |  | ✔ |  |  |  | Unlikely to be documented |
| PREV | | Infants aged 2,4, and 6 months whose parents present with concerns have an assessment of:  - parental mental health AND  - family functioning | RACGP 2013(70) | Grade C |  |  |  |  | ✔ |  |  |  |  |  |  | ✔ | ✔ |  |  | Feasibility, impact  Definitional |
| PREV | | Infants aged 2, 4 and 6 months have evidence in the medical record that parents received advice on the following: - physical activity AND  - injury prevention (accidental and non-accidental injury; passive smoking; SIDS; sun exposure; water and home environment) AND - settling AND  - maternal health AND  - teething AND  - play | RACGP 2013(70) | Grade B  Level III  GPP |  |  |  | ✔ | ✔ |  |  |  |  |  |  | ✔ |  |  |  | Will not be documented |
| PREV | | Infants aged 12 and 18 months are assessed for all of the following:  - nutrition AND  - physical activity AND  - risk of iron depletion and Vitamin D deficiency AND  - family functioning, dysfunction and social environment | RACGP 2013(70) | Grade B  Grade C |  |  |  |  |  | ✔ |  |  |  |  |  | ✔ |  |  |  | Unlikely to be documented |
| PREV | | Infants aged 12 and 18 months have evidence in the medical record that their parents received advice and counselling on:  - social and emotional wellbeing AND  - toilet training AND  - behaviour management techniques | RACGP 2013(70) | GPP |  |  |  | ✔ | ✔ |  |  |  |  |  |  | ✔ |  |  |  | Unlikely to be documented |
| PREV | | Children aged 2 years have evidence in the medical record of an assessment of the following:  - their nutrition AND  - physical activity and screen time  AND  - behaviour and emotional problems AND  - family functioning | RACGP 2013(70) | Grade B |  |  |  |  | ✔ |  |  |  |  |  |  | ✔ |  |  |  | Unlikely to be documented |
| PREV | | Children aged 2 years have evidence in the medical record that their parents received advice and counselling on all of the following:  - injury prevention AND  - sun protection AND  - social and emotional well being | RACGP 2013(70) | Level III  Grade B GPP |  |  |  |  | ✔ |  |  |  |  |  |  | ✔ |  |  |  | Unlikely to be documented |
| PREV | | Children aged 4 years have an assessment of the following recorded in the medical record: - nutrition AND  - physical activity AND  - development and emotional progress AND  - family functioning AND  - emotional and behavioural problems | RACGP 2013(70) | Grade B |  |  |  |  | ✔ |  |  |  |  |  |  | ✔ |  |  |  | Unlikely to be documented |
| PREV | | Children aged 4 years have evidence in the medical record that parents received the following information: - "Get set 4 Life" publication AND  - injury prevention AND  - sun protection AND  - social and emotional wellbeing | Get set 4 Life | Level III  Grade B  GPP  Grade C |  |  |  |  | ✔ |  |  |  |  |  | ✔ | ✔ |  |  |  | Poor acceptability & feasibility |
| PREV | | Children aged between 6 and 13 years have assessment of the following at least every year:  - height and weight and BMI AND  - progress at school AND  - "lift the lip" dental check AND  - nutrition and physical activity AND  - family functioning AND  - behaviour and emotional well being | RACGP 2013(70) | Grade B  Grade C |  |  |  |  |  | ✔ |  |  |  |  |  | ✔ |  |  |  | Feasibility (kids don’t attend annually), unlikely to be documented |
| PREV | | Children aged 6-13 years have evidence in the medical record that their parents received advice and counselling on all of the following each year:  - injury prevention AND  - sun protection AND  - social and emotional well being | RACGP 2013(70) | Grade B  Grade C |  |  |  |  | ✔ |  |  |  |  |  |  | ✔ |  |  |  | Unlikely to be documented |
| PREV | | Adolescents aged between 14 and 16 years have evidence in the medical record that the following was assessed and documented at least every year:  - received immunisations according to DOHA schedule AND - height, weight and BMI assessed AND - nutrition assessed AND  - physical activity level assessed AND  - screened for chlamydia if sexually active AND  - screened for major depressive disorder | RACGP 2013(70) | Grade B  Grade C |  |  |  |  |  | ✔ |  |  |  |  |  | ✔ |  |  |  | Unlikely to be documented  Opportunistic screening |
| PREV | | Adolescents aged between 14 and 16 years receive advice and counselling on:  - injury prevention AND  - harm minimisation AND  - sun protection AND  - social and emotional wellbeing AND  - oral health | RACGP 2013(70) | Level II; Grade C  Grade B |  |  |  |  | ✔ |  |  |  |  |  |  | ✔ |  |  |  | Feasibility  Not recorded |
| PREV | | Children aged 3 years had their vision checked. | RACGP 2013(70) | consensus |  |  |  |  |  |  |  | ✔ |  |  | ✔ |  |  |  |  | Lack of evidence |
| PREV | | Children aged 3 years have a healthy kids check that includes assessment of all of the following:  - height and weight (plot and interpret growth curve/calculate BMI) AND  - eyesight AND  - hearing AND  - oral health (teeth and gums) 'Lift the Lip' dental check AND  - toileting AND  - allergies. | RACGP 2013(70) | consensus |  |  |  |  |  |  |  | ✔ |  |  | ✔ |  |  |  |  | Not routinely assessed |
| PREV | | At the time of birth neonates had the following documented:  Weight AND  Length AND  Head circumference AND  Eyes: observation, appearance and red reflexes AND  Neurological and developmental status, including responsiveness and tone AND  Cardiovascular status AND  Umbilicus AND  Femoral pulse (for radio-femoral delay) AND  Hips (Barlow and Ortolani), limbs, joints, hands (palmar creases), feet (for talipes) AND  Genitalia, testes, anal region. | RACGP 2013(70) | Grade C |  |  |  |  |  |  |  |  |  | ✔ |  | ✔ |  |  |  | Not sampling neonates |
| PREV | | At the time of birth neonates received the following:  vitamin K administered AND  a metabolic screen AND  a nutrition assessment AND  had breastfeeding promoted AND had a universal hearing assessment AND immunisation. | RACGP - Red Book 8th edition 2013  RACGP 2013(70) | Level IV, Grade B  Grade A  Grade C |  |  |  |  |  |  |  |  |  | ✔ |  |  |  |  |  | Not sampling neonates |
| SEIZ | | Children presenting with seizure have the adequacy of their breathing assessed, with evidence of the following documented in the medical record: - Effort of breathing - recession, respiratory rate and grunting – this may be caused by  the convulsion and not be a sign of  respiratory distress in this instance AND  - Efficacy of breathing - breath sounds and chest expansion/abdominal excursion AND  - Effects of breathing - heart rate and skin colour. | NSW Health 2009(71) | consensus |  |  |  |  |  | ✔ |  |  |  |  |  | ✔ |  |  |  | Feasibility, unlikely to be documented |
| SEIZ | | Children presenting with seizure and signs of shock are given 20ml/kg rapid bolus of normal saline | NSW Health 2009(71) | consensus |  |  |  |  | ✔ |  |  |  |  |  |  |  | ✔ |  |  | Low prevalence |
| SEIZ | | Children presenting with seizure are consulted by a specialist if they have any of the following:  - airway compromise requiring intubation OR breathing compromise e.g. persistent hypoventilation, aspiration OR  - circulatory compromise e.g. requiring more than 20 mL/kg fluid bolus OR  -neurological compromise e.g. localizing signs – focal fit, asymmetry of movement, asymmetry of reflexes; prolonged depression of level of consciousness  - prolonged seizures OR - seizures continuing after two doses of  a benzodiazepine OR  - suspected serious underlying cause of  seizures e.g. meningitis, metabolic  abnormality, head injury. | NSW Health 2009(71) | consensus |  |  |  |  | ✔ |  |  |  |  |  |  | ✔ |  |  |  | Feasibility – too difficult to determine from medical record review |
| SEIZ | | Children presenting with status epilepticus or seizures (after 5 minutes) receive intravenous or Intraosseous access. | NSW Health 2009(71) | consensus |  |  |  |  |  |  |  | ✔ |  |  |  |  |  | ✔ |  | Covered in other indicators; lack of agreement scores |
| TONS | | Children aged 3-14 years who present with a sore throat and are considered HIGH risk of GABHS (≥3 Centor prediction score) are managed as follows:  - a throat swab taken AND  - commenced on empiric antibiotics. | National heart foundation NZ and the cardiac society of AU and NZ 2007(72) | Pelucchi A/1 |  |  |  |  |  | ✔ |  |  |  |  | ✔ | ✔ |  |  |  | Lack Of evidence, feasibility |
| URTI | | Children presenting with the following are diagnosed with acute bacterial sinusitis:  - viral URTI symptoms which have not improved after 10 days or that worsen after 5-7 days AND  - purulent nasal discharge AND  - daytime cough which has not improved after 10 days AND  - fever AND  - local maxillary sinus pain | NSW Health 2006 | consensus |  |  |  |  | ✔ |  |  |  |  |  | ✔ |  |  |  |  | Acceptability |
| URTI | | Children diagnosed with moderate to severe acute bacterial sinusitis are prescribed antibiotics as follows:  - first line therapy: Amoxicillin 80-90mg/kg/day for 10 days  - alternative therapy: ceftriaxone, cefuroxime or bactrim/clindamycin if allergy to penicillin | NSW Health 2006  Wong 2006(73) | consensus |  |  |  |  |  |  |  |  |  |  |  | ✔ |  |  |  | Out of scope (sinusitis) |
| URTI | | Children with URTI who:  - are systemically unwell AND  - have a moist cough which has not improved after 10 days are assessed for an alternative diagnosis. | Wong 2006(73)  Hart 2008(74) | consensus |  |  |  |  |  | ✔ |  |  |  |  |  | ✔ |  |  |  | Feasibility |
| URTI | | Children diagnosed with an URTI are given instructions to be managed as follows:  rest AND fluids AND analgesia: paracetamol or Ibuprofen. | NSW Health 2006 | consensus |  |  |  |  |  |  |  |  |  | ✔ |  |  |  |  | ✔ | Low score |
| URIN | | All children presenting with suspected UTI are assessed for the following risk factors for UTI and serious underlying pathology and have them recorded in the medical record:  History including:  – poor urine flow  – history suggesting previous UTI or confirmed previous UTI  – recurrent fever of uncertain origin  – antenatally-diagnosed renal abnormality  – family history of vesicoureteric reflux (VUR) or renal disease  – constipation  – dysfunctional voiding  Examination including:  – enlarged bladder  – abdominal mass  – evidence of spinal lesion (hair tuft, lipoma, sinus or skin discolouration over the lower spine, abnormal lower limb tone, power or reflexes)  – poor growth  – high blood pressure | NICE 2007(75) | consensus |  |  |  |  |  | ✔ |  |  |  |  |  | ✔ |  |  |  | Feasibility, unlikely to be documented |
| URIN | | All children with a proven UTI have follow up with the local paediatric team. | RCH melb 2011(76) | consensus |  |  |  |  | ✔ |  |  |  |  |  | ✔ |  |  |  |  | Acceptability  No consensus on timeframe. |
| URIN | | Children aged between 6 months and 3 years treated for UTI have radiological imaging as follows:  - renal ultrasound during acute infection if atypical UTI OR  - renal ultrasound within 6 weeks of presentation if recurrent UTI  AND  - VCUG should be considered if abnormal renal ultrasound, or poor urine flow ,or non E- coli infection, or family history of reflux | RCH melb 2011(76) | consensus |  |  |  |  |  |  |  |  |  |  |  |  |  | ✔ |  | Doubles up with indicator 11. I think 11 is more clear. |

**Rejected references**

| **Condition** | **Reference** | **Source of suggested reference** | **Reason for rejection** |
| --- | --- | --- | --- |
| ABDO | Proposed new reference: Prospective evaluation of a clinical pathway for suspected appendicitis, Pediatrics. 2014 Jan;133(1):e88-95. Epub 2013 Dec 30 | Indicator reviewer, External review (wiki) Round 1 | Thank you for the suggested reference, however this was published electronically in December 2013, which falls outside the scope of the CareTrack Kids study. NOTE: these indicators will be used in a retrospective audit of medical records from 2012-2013, and must therefore be based on references that are relevant to and fall within these parameters (allowing for a delay in healthcare professional being able to adopt newly published recommendations into their clinical practice). |

# References

1. Holland A. Acute abdominal pain in children. Australian Doctor. 2009:25-32.

2. Makin E, Davenport M. Evaluation of the acute abdomen. Paediatrics and Child Health (United Kingdom). 2012;22 (6):217-23.

3. The Royal Children's Hospital Melbourne. Abdominal Pain Melbourne2013. Available from: <http://www.rch.org.au/clinicalguide/guideline_index/Abdominal_pain/>.

4. Royal Childrens Hospital Melbourne. Paediatric Handbook - Eighth edition. Melbourne, Australia: Wiley-Blackwell; 2009.

5. The Royal Children's Hospital M. Abdominal pain 2013. Available from: <http://www.rch.org.au/clinicalguide/guideline_index/Abdominal_pain/>.

6. Leung AKC, Sigalet DL. Acute abdominal pain in children. American Family Physician. 2003;67 (11):2321-6.

7. NSW Health. Children and Infants with Acute Abdominal Pain - Acute Management (PD2005_384) Sydney2005. Available from: <http://www0.health.nsw.gov.au/policies/PD/2005/pdf/PD2005_384.pdf>.

8. Churgay CA, Aftab Z. Gastroenteritis in children: Part II. prevention and management. American Family Physician. 2012;85(11):1066-70.

9. Acute Gastroenteritis Guideline Team Cincinnati Children's hospital medical center. Evidence based care guideline -Prevention and management of Acute Gastroenteritis (AGE) in children aged 2 months to 18 years Cincinnati, Ohio2011. Available from: <http://www.cincinnatichildrens.org/search/default/?q=prevention%20and%20management%20of%20acute%20gastroenteritis&start=0&site=>.

10. NSW Health. Children and infants with gastroenteritis - acute management Syndey2010. Available from: <http://www0.health.nsw.gov.au/archive/policies/pd/2009/pdf/PD2009_064.pdf>.

11. Heinz P. Management of acute gastroenteritis in children. Paediatrics and Child Health. 2008;18(10):453-7.

12. Kelly A, Cheong, E. Paediatric gastroenteritis Sydney2007. Available from: <http://www.australiandoctor.com.au/search?q=paediatric%20gastoenteritis>.

13. National Institute for Health and Clinical Excellence (NICE). Diarrhoea and vomiting in children Diarrhoea and vomiting caused by gastroenteritis: diagnosis, assessment and management in children younger than 5 years London2009. Available from: <http://www.nice.org.uk/nicemedia/live/11846/47350/47350.pdf>.

14. Noone M. Management of acute gastroenteritis in children. Paediatrics and Child Health (United Kingdom). 2012;22(10):426-31.

15. The Royal Children's Hospital Melbourne. Gastroenteritis Melbourne2013. Available from: <http://www.rch.org.au/clinicalguide/guideline_index/Gastroenteritis/>.

16. Women's and Children's Health Network. Gastroenteritis Adelaide2010. Available from: <http://www.cyh.com/HealthTopics/HealthTopicDetails.aspx?p=114&np=303&id=1845>.

17. American Academy of paediatrics (AAP): subcommittee on diagnosis and management of bronchiolitis. Diagnosis and management of bronchiolitis. Pediatrics. 2006;118 (4):1774-93.

18. NSW Health. Infants and Children: Acute management of bronchiolitis PD2012_004 Sydney2012. Available from: <http://www0.health.nsw.gov.au/policies/pd/2012/pdf/PD2012_004.pdf>.

19. Sydney Children's Hospital. Viral Bronchiolitis Inpatient Clinical Guidelines Sydney2011. Available from: <http://www.sch.edu.au/health/professionals/cpg/viral_bronchiolitis_inpatient_clinical_guidelines.pdf>.

20. Zentz SE. Care of Infants and Children With Bronchiolitis: A Systematic Review. Journal of Pediatric Nursing. 2011;26(6):519-29.

21. The Royal Children's Hospital Melbourne. Bronchiolitis Guideline. Secondary Bronchiolitis Guideline 2012 2012. Available from: <http://www.rch.org.au/clinicalguide/guideline_index/Bronchiolitis_Guideline/>.

22. British Columbia Medical Association. Anxiety and Depression in Children and Youth – Diagnosis and Treatment 2010. Available from: <http://www.bcguidelines.ca/pdf/depressyouth.pdf>.

23. Madden S. How to treat anxiety disorder in children and adolescents. Australian Doctor. 2007(October 5):31-8.

24. Sakolsky D, Birmaher B. Pediatric anxiety disorders: management in primary care. Current Opinion in Pediatrics. 2008;20(5):538-43.

25. Canadian Psychiatric Association (CPA). Clinical practice guidelines: management of anxiety disorders 2006. Available from: <https://ww1.cpa-apc.org/Publications/CJP/supplements/july2006/anxiety_guidelines_2006.pdf>.

26. American Academy of Child and Adolescent Psychiatry. Practice parameter for the assessment and treatment of children and adolescents with anxiety disorders. Journal of the American Academy of Child and Adolescent Psychiatry. 2007;46 (2):267-83.

27. Western Australian Therapeutic Advisory Group (WA TAG). "Antidepressant combination and augmentation" and "Antipsychotic Combinations". 2008. Available from: <http://www.watag.org.au/wapdc/guidelines.cfm>.

28. British Thoracic Society, Scottish Intercollegiate Guidelines Network. British guideline on the management of asthma 2012 2012. Available from: <http://sign.ac.uk/guidelines/fulltext/101/>.

29. National Asthma Council Australia. Asthma Management Handbook 2006. National Asthma Council; 2006.

30. National Health and Medical Research Council. Clinical practice points on the diagnosis, assessment and management of Attention Deficit Hyperactivity Disorder in children and adolescents. Secondary Clinical practice points on the diagnosis, assessment and management of Attention Deficit Hyperactivity Disorder in children and adolescents 2012. Available from: <https://www.nhmrc.gov.au/guidelines/publications/mh26>.

31. American Academy of Pediatrics; Subcommittee on Attention-Deficit/Hyperactivity Disorder - Steering committee on quality improvement and management. ADHD: Clinical practice guideline for the diagnosis, evaluation, and treatment of Attention-Deficit.Hyperactivity Disorder in children and adolescents. . Pediatrics. 2011;128.

32. Kohn M. Child and adolescent ADHD. Australian Doctor. 2008(November).

33. Royal Australasian College of Physicians. A consensus approach for the paediatrician's role in the diagnosis and assessment of Autism Spectrum Disorders in Australia 2008. Available from: <https://www.racp.edu.au/index.cfm?objectid=B55D4FBA-BFD6-A9E7-152508A3109AC225>.

34. Scottish Intercollegiate Guidelines Network (SIGN). Assessment, diagnosis and clinical interventions for children and young people with autism spectrum disorders 2007. Available from: <http://www.sign.ac.uk/guidelines/fulltext/98/index.html>.

35. Tonge B, & Brereton A. Autism spectrum disorders. Australian Family Physician. 2011;40(9):672-77.

36. Australian Government Department of Health and Ageing. Early intervention for children with autism spectrum disorders: guidelines for best practice 2007. Available from: [www.health.gov.au/internet/publications/publishing.nsf/Content/mental-child-autbro-toc~mental-child-autbro-best](http://www.health.gov.au/internet/publications/publishing.nsf/Content/mental-child-autbro-toc~mental-child-autbro-best) - no longer available online.

37. Harrison J, J. M. Acute respiratory infections in children. Australian Doctor. 2009(May):27-34.

38. The Royal Children's Hospital Melbourne. Clinical practice guidelines: Croup (Laryngotacheobronchitis). 2011. Available from: <http://www.rch.org.au/clinicalguide/guideline_index/Croup_Laryngotracheobronchitis/>.

39. Health for Kids in the South East SH. Evidence-based practice guideline for the management of croup in children 2007. Available from: No longer available online.

40. Campus JH. Nurse Practitioner - emergency services: Clinical practice guideline management of croup. 2006. Available from: <http://www.nursing.health.wa.gov.au/docs/career/np/joondalup/CPG_Croup.pdf>.

41. National Institute for Health and Care Excellence (NICE). Croup 2012.

42. Association. AM. Guideline for the diagnosis and management of croup. 2009. Available from: <http://www.topalbertadoctors.org/download/252/croup_guideline.pdf>.

43. Sydney West Area Health Service. Nurse Practitioner clinical practice guidelines for the management of croup 2004. Available from: No longer available online.

44. NHMRC. Clinical practice guidelines: depression in adolescents and young adults. 2011. Available from: <https://www.nhmrc.gov.au/guidelines/publications/ext0007>.

45. Cheung AH, Zuckerbrot RA, Jensen PS, Ghalib K, Laraque D, Stein REK. Guidelines for adolescent depression in primary care (GLAD-PC): II. Treatment and ongoing management. Paediatrics. 2007;120(55):e1313-26.

46. National Institute for health and Clinical Excellence (NICE). Depression in children and young people: Identification and management in primary, community and secondary care 2005.

47. Australasian Paediatric Endocrine Group, and the Australian Diabetes Society. National evidence-based clinical care guidelines for type 1 diabetes in children, adolescents and adults. 2011. Available from: <https://www.nhmrc.gov.au/guidelines-publications/ext4>.

48. The Royal Children's Hospital Melbourne. Diabetes Mellitus. Secondary Diabetes Mellitus 2013. Available from: <http://www.rch.org.au/clinicalguide/guideline_index/Diabetes_Mellitus/>.

49. Royal Children's Hospital M. Eczema management. Secondary Eczema management 2013. 2013. Available from: <http://www.rch.org.au/rchcpg/hospital_clinical_guideline_index/Eczema_management/>.

50. Scottish Intercollegiate Guidelines Network (SIGN). Management of atopic eczema in primary care (CG125). Secondary Management of atopic eczema in primary care (CG125) 2011. Available from: <http://www.sign.ac.uk/guidelines/fulltext/125/index.html>.

51. National Institute for Health and Clinical Excellence (NICE). Feverish illness in children: assessment and initial management in children younger than 5 years 2013. Available from: <https://www.nice.org.uk/guidance/cg160>.

52. NSW Kids and Families. Policy directive: Children and infants with fever - acute management. 2010. Available from: <http://www0.health.nsw.gov.au/policies/pd/2010/PD2010_063.html>.

53. SA Child Health Clinical Network. Management of fever without focus in children (excluding neonates). Secondary Management of fever without focus in children (excluding neonates) 2013. Available from: <http://www0.health.nsw.gov.au/policies/pd/2010/PD2010_063.html>.

54. The Royal Children's Hospital Melbourne. Febrile child. 2011. Available from: <http://www.rch.org.au/clinicalguide/guideline_index/Febrile_Child/>.

55. Cincinnati Children's Hospital Medical Center. Evidence-based care guideline for fever of uncertain source in infants 60 days of age or less. 2010. Available from: <http://www.cincinnatichildrens.org/workarea/downloadasset.aspx?id=87913>.

56. Vandenplas Y, Rudolph CD, Di Lorenzo C, Hassall E, Liptak G, Mazur L, et al. Pediatric gastroesophageal reflux clinical practice guidelines: Joint recommendations of the North American Society for Pediatric Gastroenterology, Hepatology, and Nutrition (NASPGHAN) and the European Society for Pediatric Gastroenterology, Hepatology, and Nutrition (ESPGHAN). Journal of Pediatric Gastroenterology and Nutrition. 2009;49(4):498-547.

57. Bhavsar H, Cullen M, Beattie RM. Gastro-oesophageal reflux in infancy. Paediatrics and Child Health. 2011;21(9):394-400.

58. The Royal Children's Hospital Melbourne. Gastrooesophageal reflux in infants Melbourne2012. Available from: <http://www.rch.org.au/clinicalguide/guideline_index/Gastrooesophageal_Reflux_in_infants/>.

59. The Royal Children's Hospital Melbourne. Trauma - Head Injury ND. Available from: <http://www.rch.org.au/clinicalguide/guideline_index/Head_Injury_Guideline/>.

60. Families NKa. Children and infants - acute management of head injury 2011. Available from: <http://www0.health.nsw.gov.au/policies/pd/2011/pdf/PD2011_024.pdf>.

61. National Health & Medical Research Council (NHMRC). Clinical practice guidelines for the management of overweight and obesity in adults, adolescents and children in Australia Canberra 2013. Available from: <http://www.nhmrc.gov.au/_files_nhmrc/publications/attachments/n57>.

62. National Institute for Health and Clinical Excellence (NICE). Obesity: guidance on the prevention, identification, assessment and management of overweight and obesity in adults and children (CG43). London. 2006.

63. Hearnshaw C, Matyka K. Managing childhood obesity: When lifestyle change is not enough. Diabetes, Obesity and Metabolism. 2010;12 (11):947-57.

64. Baur LA, Fitzgerald DA. Recommendations for bariatric surgery in adolescents in Australia and New Zealand. Journal of Paediatrics and Child Health. 2010;46 (12):704-7.

65. August GP, Caprio S, Fennoy I, Freemark M, Kaufman FR, Lustig RH, et al. Prevention and treatment of pediatric obesity: An Endocrine Society clinical practice guideline based on expert opinion. Journal of Clinical Endocrinology and Metabolism. 2008;93 (12):4576-99.

66. Lieberthal AS, Carroll AE, Chonmaitree T, Ganiats TG, Hoberman A, Jackson MA, et al. The diagnosis and management of acute otitis media. Pediatrics. 2013;131 (3):e964-e99.

67. NSW Health. Children and infants with otitis media - acute management Sydney2005. Available from: <http://www0.health.nsw.gov.au/policies/PD/2005/pdf/PD2005_385.pdf>.

68. Gunasekera H. Otitis media in children. Australian Doctor. 2008(July ):33-40.

69. The Royal Children's Hospital Melbourne. Acute otitis media Melbourne2012. Available from: <http://www.rch.org.au/clinicalguide/guideline_index/Acute_Otitis_Media/>.

70. Royal Australian College of General Practitioners. Guidelines for preventive activities in general practice 8th edition - Preventive activities in children and young people. 2013. Available from: <http://www.racgp.org.au/your-practice/guidelines/redbook/>.

71. NSW Kids and Families. Children and infants with seizures - acute management 2009. Available from: <http://www0.health.nsw.gov.au/policies/pd/2009/PD2009_065.html>.

72. TCSoAaNZ. HFN. GUIDE: for sore throat management 2007. Available from: <http://www.world-heart-federation.org/fileadmin/user_upload/documents/RHD-net/AUS_NZ_resources/Guidelines/NZRh_F_Algorithm_4.pdf>.

73. Wong DM, Blumberg DA, Lowe LG. Guidelines for the use of antibiotics in acute upper respiratory tract infections. American Family Physician. 2006;74(6):956-66+69.

74. Hart A, Patti A, Noggle B, Haller-Stevenson E, Hines L. Acute Respiratory Infections and Antimicrobial Resistance. American Journal of Nursing. 2008;108(6):56-65.

75. National Institute for Health and Care Excellence (NICE). Urinary tract infection in under 16s: diagnosis and management 2007. Available from: <https://www.nice.org.uk/guidance/CG54>.

76. Royal Children's Hospital Melbourne. Urinary Tract Infection Guideline Melbourne2011. Available from: <http://www.rch.org.au/clinicalguide/guideline_index/Urinary_Tract_Infection_Guideline/>.
