# Supplementary material for: Clinical indicators for common paediatric conditions: Processes, provenance and products of the CareTrack Kids study
Source: PLoS One. 2019 Jan 9;14(1):e0209637. doi: 10.1371/journal.pone.0209637 (PMC6326465; doi:10.1371/journal.pone.0209637)
Supplement: S4 Table — (DOCX) [file pone.0209637.s004.docx]

**S4 Table**. ***CareTrack Kids*** ‘new’ indicators by way of splitting existing indicators mapped to their corresponding final medical record indicator items.

* ‘new’ indicators are presented (i.e. content, format) as they were first introduced in the development process and may have undergone further refinement in subsequent review rounds to become their corresponding final medical record indicator item.

| **Indicator item** | **‘New’ indicators*** | **Source** | **Level of evidence** | **Classification** | **U / O** | **Original indicator** | **Reason for splitting** |
| --- | --- | --- | --- | --- | --- | --- | --- |
| ABDO16 | Children presenting with acute abdominal pain classified as severe are administered IV morphine or intranasal fentanyl. | Makin E, Davenport M. 2012(1)  Leung AKC, Sigalet DL. 2003(2)  NSW Kids and Families. 2005(3) | Grade A | Treatment | U | Children presenting with acute abdominal pain are administered pain relief as follows:  - severe pain - IV morphine or intranasal fentanyl OR  - less severe pain- paracetamol (PO, IV or PR), oxycodone for more moderate pain | This indicator was split into three separate indicators to reflect the different eligibility (i.e. severity of pain) and compliance criteria. |
| *REJECTED*  *(low ‘appropriateness’ score)* | Children presenting with acute abdominal pain classified as moderate pain are administered oxycodone | NSW Health 2005(3)  Leung AKC, Sigalet DL. 2003(2)  Makin E, Davenport M. 2012(1) | Grade A (Leung) | Treatment | U |  |  |
| ABDO17 | Children presenting with acute abdominal pain classified with less severe pain are administered paracetamol | Makin E, Davenport M. 2012(1)  Leung AKC, Sigalet DL. 2003(2)  NSW Kids and Families. 2005(3) | Grade A | Treatment | U |  |  |
| ABDO18 | Children presenting with acute abdominal pain who are dehydrated are managed as follows:  - electrolytes measured AND  - blood sugar measured AND  - fluid resuscitation (initial bolus 10ml/kg normal saline) | The RCH Melbourne. 2013(4) | Consensus-based recommendation | Treatment | U | Children presenting with acute abdominal pain that are dehydrated or shocked are managed as follows:  - electrolytes measured AND  - blood sugar measured AND  - Maintenance fluids commenced AND  - Replacement fluids commenced  - fluid resuscitation in cases of shock (initial bolus 20ml/kg normal saline) | This indicator was split into two separate indicators to reflect the different eligibility (i.e. dehydration or shocked status) and compliance criteria. |
| ABDO19-ABDO21 | Children presenting with acute abdominal pain who are shocked are managed as follows:  - electrolytes measured AND  - blood sugar measured AND  - maintenance fluids commenced AND  - replacement fluids commenced, AND  - fluid resuscitation (initial bolus 20ml/kg normal saline) | The RCH Melbourne. 2013(4) | Consensus-based recommendation | Treatment | U |  |  |
| BRON01 – BRON06 | Infants (aged <12months) presenting with acute bronchiolitis have the following history recorded:  - duration and progression of symptoms AND  - presence of apnoea AND  - feeding AND  - previous episodes of bronchiolitis, AND  - family history of atopy or apnoea, AND  - pre-existing conditions | SA Child Health Clinical Network. 2013(5)  NSW Kids and Families. 2012(6)  The Royal Children's Hospital Melbourne. 2012(7)  The Royal Children's Hospital Melbourne. 2013(8)  Sydney Children's Hospital. 2011(9)  Zentz SE. 2011(10)  American Adacemy of Pediatrics. 2006(11)  Scottish Intercollegiate Guidelines Network. 2006(12) | Grade B | Diagnosis | U | Children presenting with symptoms of acute bronchiolitis have evidence in the medical record that the following was performed to inform the diagnosis: - physical examination performed AND  - age recorded AND  - history recorded AND  - duration of and progression of symptoms recorded. | This indicator was divided into two separate indicators to reflect different criteria for HISTORY and EXAMINATION. |
| BRON07 – BRON10 | Infants (aged <12months) presenting with acute bronchiolitis have the following examined:  - General appearance and basic observations (Temp, RR, HR, SaO2, colour)  - Hydration (review of skin turgor, capillary refill, peripheral refill, mucous membranes, fontanelle)  - Respiratory examination (work of breathing, recession, auscultation)  - Feeding (duration and volume, saturations whilst feeding) | SA Child Health Clinical Network. 2013(5)  NSW Kids and Families. 2012(6)  The Royal Children's Hospital Melbourne. 2012(7)  The Royal Children's Hospital Melbourne. 2013(8)  Sydney Children's Hospital. 2011(9)  Zentz SE. 2011(10)  American Adacemy of Pediatrics. 2006(11).  Scottish Intercollegiate Guidelines Network. 2006(12) | Grade D | Diagnosis | U |  |  |
| BRON36 | Infants (aged <12 months) presenting to the GP with acute bronchiolitis and two of the following:  - poor feeding (<50% of usual fluid intake in preceding 24 hours) OR  - lethargy OR  - history of apnoea OR  - respiratory rate >60/min OR <30/min  - nasal flaring and/or grunting) OR  - severe chest wall recession or tracheal tug OR  - cyanosis OR  - oxygen saturation <95% on air OR  - uncertainty regarding diagnosis  are referred to hospital. | Scottish Intercollegiate Guidelines Network. 2006(12) | Consensus-based recommendation | Ongoing management | U | Children presenting to the GP or hospital with acute bronchiolitis or suspected acute bronchiolitis and any of the following:  - poor feeding (<50% of usual fluid intake in preceding 24 hours) OR  - lethargy OR  - history of apnoea OR  - respiratory rate >70/min OR  - presence of nasal flaring and/or grunting OR  - severe chest wall recession OR  - cyanosis OR  - oxygen saturation <96% OR  - uncertainty regarding diagnosis  are referred for urgent review at hospital | This indicator was split into three separate indicators to reflect:  (a) two different compliance criteria (i.e. referred to hospital, and urgent review)  (b) the differing severity of bronchiolitis (i.e. moderate vs severe) and hence the immediacy of review required. |
| BRON30 | Infants (aged <12 months) presenting to the ED with acute moderate bronchiolitis and any of the following:  - lethargy OR  - presence of nasal flaring and/or grunting OR  - oxygen saturation <95% on air OR  - uncertainty regarding diagnosis  are reviewed within 30 minutes. | Scottish Intercollegiate Guidelines Network. 2006(12) | Consensus-based recommendation | Treatment | U |  |  |
| BRON31 | Infants (aged <12 months) presenting to the ED with acute severe bronchiolitis and any of the following:  - poor feeding (<50% of usual fluid intake in preceding 24 hours) OR  - respiratory rate >60/min OR  - presence of nasal flaring and/or grunting OR  - severe chest wall recession OR  - cyanosis OR  are reviewed immediately. | Scottish Intercollegiate Guidelines Network. 2006(12) | Consensus-based recommendation | Treatment | U |  |  |
| CROU01 – CROU05 | Children diagnosed with croup have the following assessed to determine the severity:  heart rate AND  mental state AND  work of breathing AND  stridor AND  SpO_2_ / oxygen requirement. | The Royal Children's Hospital Melbourne. 2011(13)  NSW Health. 2010(14)  Rajapaksa S, Starr M. 2010(15)  Health for Kids in the South East, Southern Health. 2007(16)  Sydney West Area Health Service. 2004(17)  Harrison J, Massie J.2009(18) | Grade D | Diagnosis | U | Children diagnosed with croup have the following assessed to determine the severity and have it recorded as mild/moderate/severe:  - heart rate AND  - mental state AND  - work of breathing AND  - stridor AND  - SpO2/oxygen requirement | This indicator was split into two to capture the separate compliance criteria (i.e. assessment items, and the recording of severity) |
| CROU06 | Children diagnosed with croup have the severity recorded as mild / moderate / severe. | The Royal Children's Hospital Melbourne. 2011(13)  NSW Health. 2010(14)  Rajapaksa S, Starr M. 2010(15)  Health for Kids in the South East, Southern Health. 2007(16)  Sydney West Area Health Service. 2004(17)  Harrison J, Massie J.2009(18) | Grade D | Diagnosis | U |  |  |
| FEVE30 – FEVE32 | Infants aged < 3 months presenting to ED with fever (over 38^o^C) who are shocked OR unrousable OR showing signs of meningococcal disease receive:  - immediate antibiotics AND  - immediate fluid resuscitation AND  - referral to retrieval service or paediatric PICU | NSW Kids and Families. 2010(19) | Consensus-based recommendation | Treatment | U | Infants aged < 1 month presenting with fever that are: - shocked OR  - unrousable OR  - showing signs of meningococcal disease  are urgently reviewed by an experienced paediatrician and referred to paediatric ICU after commencing antibiotics. | This indicator was split into two separate indicators to reflect distinct compliance actions.  Please note: the age range was amended in accordance with reviewer feedback. |
| *REJECTED*  *(covered in another indicator)* | Infants aged < 3 months presenting to a GP with fever (over 38^o^C) who are shocked OR unrousable OR showing signs of meningococcal disease receive:  - immediate antibiotics AND  - transfer to hospital | SA Child Health Clinical Network. 2013(20) | Consensus-based recommendation | Treatment | U |  |  |
| *REJECTED*  *(acceptability)* | Infants aged < 4 months presenting with fever (over 38^o^C) where meningitis has been excluded are commenced on antibiotics (pending blood culture results i.e. up to 48 hours) as follows:  - amoxicillin OR ampicillin 50mg/kg IV 6 hourly AND gentamicin 7.5 mg/kg IV for 1 dose | Cincinnati Childrens Hospital 2010(21)  SA Child Health Clinical Network. 2013(20) | Consensus-based recommendation | Treatment | U | Infants aged < 1 month presenting with fever are commenced on antibiotics (pending blood culture results) as follows: - **If CSF negative** - Ampilcillin week 1 of life: 25mg/kg/dose IV 12 hourly; week 2-4 of life: 25 mg/kg/dose IV 6 hourly PLUS Gentamicin OR  - **If CSF positive or unknown** (bloody tap, child too sick for LP etc) administer Ampilicillin - week 1 of life: 100mg/kg/dose IV 12 hourly; week 2-4 of life 100mg/kg/dose IV 6 hourly PLUS cefotaxime. | Separate indicators were created to reflect the management/decision-making by (a) children’s age (i.e. aged < or > 4 or 6 months) and (b) whether meningitis has been excluded. The KEY here is identifying how clinicians would/should/could “exclude meningitis” without ALL children receiving lumbar punctures (which is not practical/feasible/likely). |
| *REJECTED*  *(feasibility)* | Infants aged > 4 months (i.e. aged 4 months or more) presenting with fever and where meningitis has been excluded are commenced on antibiotics (pending blood culture results i.e. up to 48hours) as follows:  -cefotaxime 25mg/kg (up to 1 g) IV 6 hourly OR  - ceftriaxone 25 mg/kg (up to 1 g) IV daily AND flucloxacillin 50mg/kg (up to 2g) IV 6 hourly | Cincinnati Childrens Hospital 2010(21)  SA Child Health Clinical Network. 2013(20) | Consensus-based recommendation | Treatment | U |  |  |
| *REJECTED*  *(feasibility)* | Infants aged < 6 months presenting with fever where meningitis has NOT been excluded are commenced on antibiotics (pending blood culture results) as follows:  - amoxicillin OR ampicillin 50mg/kg IV 6 hourly AND  - cefotaxime 50 mg/kg IV 6 hourly AND  - vancomycin 30 mg/kg IV 12 hourly (if pneumococcal meningitis is likely) | Cincinnati Childrens Hospital 2010(21)  SA Child Health Clinical Network. 2013(20) | Consensus-based recommendation | Treatment | U |  |  |
| *REJECTED*  *(feasibility)* | Infants aged > 6 months (i.e. aged 6 months or more) presenting with fever where meningitis has NOT been excluded are commenced on antibiotics (pending blood culture results) as follows:  - cefotaxime 50mg/kg (up to 2g) IV 6 hourly, OR  - ceftriaxone 100 100mg/kg (up to 4g) IV daily OR  - ceftriaxone 50mg/kg (up to 2g) IV 6 hourly  AND  - flucloxacillin 50mg/kg (up to 2g) IV 6 hourly  (if pneumococcal meningitis is likely, also add: vancomycin 30mg/kg up to 1.5g IV 12 hourly) | Cincinnati Childrens Hospital 2010(21)  SA Child Health Clinical Network. 2013(20) | Consensus-based recommendation | Treatment | U |  |  |
| GORD01 – GORD03 | Infants/Children presenting with regurgitation have the following documented:  - weight and height (growth chart) AND  - allergies (skin rash/urticaria/ eczema/diarrhoea/perineal/perianal excoriation), food and milk intolerances (cows’ milk) AND  - history of sleep disturbance AND  - history of food refusal, regurgitation and abdominal pain (children aged 1-6 years), regurgitation/vomiting, cough, epigastric pain/heartburn (children aged 6-17 years) | Queensland Government Children's Health Service Royal Children's Hospital. 2011.  The Royal Children's Hospital Melbourne. 2012(22)  Vandenplas et al 2009(23)  Lightdale et al 2013(24) | Consensus-based recommendation | Diagnosis | U | Infants/Children presenting with regurgitation have the following assessments performed and documented:  - weight and height recorded (growth chart) AND  - Urine MC&S AND  - history of allergies (skin rash/urticaria/ eczema/diarrhoea/perineal excoriation), food and milk intolerances (cow’s milk) AND  - associated signs and symptoms NASPHGAN | This indicator was split into two separate indicators to address screening of infants/children, especially with respect to differentiating between GORD and regurgitation; and the investigations that should be undertaken on diagnosis of GORD using NASPGHAN criteria and signs and symptoms. |
| GORD04-GORD05 | Infants/children presenting with:  - history of food refusal OR regurgitation/vomiting OR abdominal/epigastric pain AND  - loss of weight or failure to thrive, AND  - history of allergies  have the following investigations:  - weight and height (growth chart) AND  - urine MC&S | Queensland Government Children's Health Service Royal Children's Hospital. 2011.  The Royal Children's Hospital Melbourne. 2012(22).  Vandenplas et al 2009(23)  Lightdale et al 2013(24) | Consensus-based recommendation | Diagnosis | U |  |  |
| GORD22 – GORD23 | Older children/adolescents presenting with heartburn are managed as follows:  - assessed for lifestyle factors (diet, alcohol, weight, sleeping position, smoking) AND  - prescribed a PPI for 4 weeks | Vandenplas et al 2009(23) | Grade A | Treatment | U | Older children/adolescents presenting with heartburn are managed as follows: - assessed for lifestyle factors (diet, alcohol, weight, sleeping position, smoking) AND - prescribed a PPI for 4 weeks AND  - reassessed after 4 weeks of PPI therapy AND - continued on PPI for 3 months if symptoms resolved OR  - PPI ceased if symptoms do not improve | This indicator was split into two separate indicators to reflect the initial and subsequent (i.e. after a PPI has been prescribed) management recommended. |
| GORD24 – GORD25 | Older children/adolescents presenting with heartburn who have been prescribed and used a PPI for 4 weeks:  - are reviewed by their GP AND  - if symptoms resolved/improved, continue PPI for 3 months OR  - if recurrent/persistent symptoms, PPI therapy is ceased and they are referred to a gastroenterologist | Vandenplas et al 2009(23) | Grade D | Ongoing management | U |  |  |
| GORD26 – GORD27 | Infants/Children (aged < 18 months) with reflux oesophagitis are managed as follows:  - lifestyle factors assessed (diet, alcohol, weight, sleeping position, smoking) AND  - prescribed a PPI for 3 months (if persistent or recurrent symptoms) AND  - have symptoms recorded at each review AND  - have medication ceased at each review | Vandenplas et al 2009(23) | Grade A | Ongoing management | U | Infants/Children who are diagnosed with reflux oesophagitis are managed as follows: - lifestyle factors assessed (diet, alcohol, weight, sleeping position, smoking) AND - prescribed a PPI for 3 months AND  - have symptoms reassessed regularly AND - have medication tapered/ceased at each review | This indicator was split into two separate indicators to reflect the initial and subsequent (i.e. after a PPI has been prescribed) management recommended. |
| *REJECTED*  *(multiple eligibility criteria therefore low prevalence)* | Infants/Children (aged <18 months) with reflux oesophagitis who are:  - receiving PPI therapy for 3 months and have persistent/recurrent symptoms OR  - are unable to have their medication ceased at each review (PPI dependency)  are referred to a paediatric gastroenterologist. | Vandenplas et al 2009(23) | Grade D | Ongoing management | U |  |  |
| GORD15 | Children aged greater than 18 months who present with dysphagia or odynophagia are referred to a paediatric gastroenterologist. | Vandenplas et al 2009(23) | Consensus-based recommendation | Treatment | U | Children aged > 18 months who present with dysphagia or odynophagia receive:  - a barium swallow and  - a referral to a paediatric gastroenterologist | This indicator was split into two separate indicators to accommodate the fact that each of these compliance actions may be undertaken by different healthcare providers (i.e. GP to refer to paediatric gastroenterologist who orders the barium swallow). |
| GORD16 | Children aged greater than 18 months who present with dysphagia or odynophagia receive a barium swallow. | Vandenplas et al 2009(23) | Grade D | Treatment | U |  |  |
| *REJECTED*  *(covered in other indicators)* | Children presenting with status epilepticus or seizure (after 5 minutes) receive intravenous or intraosseous access | NSW Kids and Families. 2009(25) | Consensus-based recommendation | Treatment | U | Children presenting with seizure are managed as follows at the time of presentation: - intravenous access obtained or intraosseous access if IV access not obtained or signs of shock AND  - pupillary size, reaction and symmetry assessed and recorded AND - assessed for focal neurological signs during or after the seizure AND - has their posture recorded (decorticate or decerebrate) AND - assessed for neck stiffness and full fontanelle in an infant AND - assessed for rashes or bruising which may indicate sepsis or injury AND - have their BP recorded as soon as the seizure has ended | This indicator was split into two separate indicators to reflect the different types of compliance actions (i.e. establishing access, and assessments) |
| SEIZ09 – SEIZ13 | Children presenting with a seizure has evidence in the medical record of the following assessments at the time of presentation:  - pupillary size, reaction and symmetry AND  - focal neurological signs during or after the seizure AND  - neck stiffness and full fontanelle in an infant AND  rashes or bruising which may indicate sepsis or injury AND  - BP as soon as the seizure has ended | NSW Kids and Families. 2009(25)  Scottish Intercollegiate Guidelines Network. 2005(26) | Consensus-based recommendation | Diagnosis | U |  |  |
| URIN19 – URIN21 | Children with a confirmed UTI who are aged ≥ 12 months who appear septic are managed as follows:  - admitted for IV antibiotics AND  - have blood cultures and electrolytes performed AND  - given IV fluid resuscitation if shocked | Royal Children's Hospital Melbourne. 2011(27)  National Institute of Health and Clinical Excellence. 2007(28) | Consensus-based recommendation | Treatment | U | Children with a confirmed UTI that are aged < 3 months OR are unwell are managed as follows:  - admitted for IV antibiotics – gentamicin (7.5 mg/kg if 1month-10 year, 6mg/kg if older than 10 years, 4mg/kg/dose if < 1 month- need to adjust frequency based on gestational age) and ampicillin (25-50 mg/kg qid)(if they have had previous UTIS the antibiotics should be prescribed according to the sensitivities)  - if a penicillin allergy is present cefotaxime (50 mg/kg/dose qid) or ceftriaxone (50 mg/kg/dose daily) can be substituted for the ampicillin  - have gentamicin levels performed pre third dose if continued for > 3 doses - have blood cultures and electrolytes performed - given IV fluid resuscitation if shocked  - if IV access not able to be obtained gentamicin and ceftriaxone should be given intramuscularly | This indicator was split into two separate indicators to reflect the different eligibility criteria (i.e. age groups and septic status). |
| URIN16 – URIN18 | Infants aged < 12 months with a confirmed UTI are managed as follows:  - admitted for IV antibiotics AND  - have blood cultures and electrolytes performed AND  - given IV fluid resuscitation if shocked | Royal Children's Hospital Melbourne. 2011(27)  National Institute of Health and Clinical Excellence. 2007(28) | Consensus-based recommendation | Treatment | U |  |  |
| URIN27 | Children who are treated for an atypical UTI have a renal ultrasound during the acute infection phase | National Institute of Health and Clinical Excellence. 2007(28) | Consensus-based recommendation | Treatment | U | Children aged < 6 months of age treated for UTI have radiological imaging as follows:  - renal ultrasound during acute infection if atypical UTI (ie organisms other than E.coli, bladder or abdominal mass, poor response to 48 hours of appropriate antibiotic treatment), renal impairment) or recurrent UTI  OR  - renal ultrasound within 6 weeks if they responded well to treatment within 48 hours AND  - VCUG if abnormal ultrasound, atypical UTI or recurrent UTI | This indicator was divided into three separate indicators to simplify and capture different eligibility criteria and/or compliance actions. |
| URIN28 | Children with a UTI who have responded well to treatment within 48 hours have a renal ultrasound within 6 weeks. | National Institute of Health and Clinical Excellence. 2007(28) | Consensus-based recommendation | Treatment | U |  |  |
| URIN29 | Infants (aged < 6 months) with a UTI and who have an abnormal ultrasound, atypical UTI or recurrent UTI receive a voiding cystourethrogram (VCUG). | National Institute of Health and Clinical Excellence. 2007(28) | Consensus-based recommendation | Treatment | U |  |  |
| URTI08 – URTI09 | Clinicians should reassure parents that antibiotics are not needed immediately for URTI because of the following:  - they are likely to make little difference to symptoms AND  - may have side effects (diarrhoea, vomiting and rash) | National Institute for Health and Care Excellence. 2008(29) | Consensus-based recommendation | Treatment | U | Clinicians should reassure parents that antibiotics are not needed immediately for URTI because of the following:  - they are likely to make little difference to symptoms AND  - may have side effects (diarrhoea, vomiting and rash) AND  - they should be advised to return if the condition worsens or becomes prolonged. | This indicator was split into two separate indicators to reflect the different compliance criteria (i.e. reassurance that antibiotics are not needed immediately, advice to return if the condition worsens or becomes prolonged). |
| URTI14 | Parents of children with an URTI should be advised to return if the condition worsens or becomes prolonged. | National Institute for Health and Care Excellence. 2008(29) | Consensus-based recommendation | Ongoing management | U |  |  |

1. Makin E, Davenport M. Evaluation of the acute abdomen. Paediatrics and Child Health (United Kingdom). 2012;22 (6):217-23.

2. Leung A K C, Sigalet D L. Acute abdominal pain in children. American Family Physician. 2003;67 (11):2321-6.

3. NSW Kids and Families. Infants and children: acute management of abdominal pain 2005. Available from: <http://www0.health.nsw.gov.au/policies/PD/2005/pdf/PD2005_384.pdf>.

4. The Royal Children's Hospital Melbourne. Abdominal Pain Melbourne2013. Available from: <http://www.rch.org.au/clinicalguide/guideline_index/Abdominal_pain/>.

5. SA Child Health Clinical Network. Clinical Guideline: Management of Bronchiolitis in children 2013. Available from: <http://www.sahealth.sa.gov.au/wps/wcm/connect/0a3fd50040d03f4d96fbbe40b897efc8/Bronchiolitis+in+Children_Aug2013.pdf?MOD=AJPERES&CACHEID=0a3fd50040d03f4d96fbbe40b897efc8>.

6. NSW Kids and Families. Infant and Children - Acute Management of Bronchiolitis 2012. Available from: <http://www0.health.nsw.gov.au/policies/pd/2012/pdf/PD2012_004.pdf>.

7. The Royal Children's Hospital Melbourne. Bronchiolitis Guideline. Secondary Bronchiolitis Guideline 2012 2012. Available from: <http://www.rch.org.au/clinicalguide/guideline_index/Bronchiolitis_Guideline/>.

8. The Royal Children's Hospital Melbourne. Bronchiolitis - Ongoing management. Secondary Bronchiolitis - Ongoing management 2013. Available from: <http://www.rch.org.au/clinicalguide/guideline_index/Bronchiolitis_Guideline/>.

9. Sydney Children's Hospital. Viral Bronchiolitis Inpatient Clinical Guidelines Sydney2011. Available from: <http://www.sch.edu.au/health/professionals/cpg/viral_bronchiolitis_inpatient_clinical_guidelines.pdf>.

10. Zentz SE. Care of Infants and Children With Bronchiolitis: A Systematic Review. Journal of Pediatric Nursing. 2011;26(6):519-29.

11. American Academy of paediatrics (AAP): subcommittee on diagnosis and management of bronchiolitis. Diagnosis and management of bronchiolitis. Pediatrics. 2006;118 (4):1774-93.

12. Scottish Intercollegiate Guidelines Network (SIGN). Bronchiolitis in children - a national clinical guideline no.91 Edinburgh2006. Available from: <http://www.sign.ac.uk/pdf/sign91.pdf>.

13. The Royal Children's Hospital Melbourne. Clinical practice guidelines: Croup (Laryngotacheobronchitis). 2011. Available from: <http://www.rch.org.au/clinicalguide/guideline_index/Croup_Laryngotracheobronchitis/>.

14. NSW Health. Children and infants - Acute management of croup 2010. Available from: <http://www0.health.nsw.gov.au/policies/pd/2010/PD2010_053.html>.

15. Rajapaksa S, Starr M. Croup: Assessment and management. Australian Family Physician 2010;39(5):280-82.

16. Health for Kids in the South East SH. Evidence-based practice guideline for the management of croup in children 2007. Available from: No longer available online.

17. Sydney West Area Health Service. Nurse Practitioner clinical practice guidelines for the management of croup 2004. Available from: No longer available online.

18. Harrison J, Massie J. Acute respiratory infections in children. Australian Doctor. 2009(May):27-34.

19. NSW Kids and Families. Policy directive: Children and infants with fever - acute management. 2010. Available from: <http://www0.health.nsw.gov.au/policies/pd/2010/PD2010_063.html>.

20. SA Child Health Clinical Network. Management of fever without focus in children (excluding neonates). Secondary Management of fever without focus in children (excluding neonates) 2013. Available from: <http://www0.health.nsw.gov.au/policies/pd/2010/PD2010_063.html>.

21. Cincinnati Children's Hospital Medical Center. Evidence-based care guideline for fever of uncertain source in infants 60 days of age or less. 2010. Available from: <http://www.cincinnatichildrens.org/workarea/downloadasset.aspx?id=87913>.

22. The Royal Children's Hospital Melbourne. Gastrooesophageal reflux in infants Melbourne2012. Available from: <http://www.rch.org.au/clinicalguide/guideline_index/Gastrooesophageal_Reflux_in_infants/>.

23. Vandenplas Y, Rudolph CD, Di Lorenzo C, Hassall E, Liptak G, Mazur L, et al. Pediatric gastroesophageal reflux clinical practice guidelines: Joint recommendations of the North American Society for Pediatric Gastroenterology, Hepatology, and Nutrition (NASPGHAN) and the European Society for Pediatric Gastroenterology, Hepatology, and Nutrition (ESPGHAN). Journal of Pediatric Gastroenterology and Nutrition. 2009;49(4):498-547.

24. Lightdale JR, Gremse DA, Heitlinger LA, Cabana M, Gilger MA, Gugig R, et al. Gastroesophageal reflux: Management guidance for the pediatrician. Pediatrics. 2013;131(5):e1684-e95.

25. NSW Kids and Families. Children and infants with seizures - acute management 2009. Available from: <http://www0.health.nsw.gov.au/policies/pd/2009/PD2009_065.html>.

26. Scottish Intercollegiate Guidelines Network. Diagnosis and management of epilepsies in children and young people 2005. Available from: <http://www.sign.ac.uk/pdf/sign81.pdf>.

27. Royal Children's Hospital Melbourne. Urinary Tract Infection Guideline Melbourne2011. Available from: <http://www.rch.org.au/clinicalguide/guideline_index/Urinary_Tract_Infection_Guideline/>.

28. National Institute for Health and Care Excellence (NICE). Urinary tract infection in under 16s: diagnosis and management 2007. Available from: <https://www.nice.org.uk/guidance/CG54>.

29. National institute for Health and Clinical Excellence (NICE). Respiratory tract infections (CG69) 2008. Available from: <http://guidance.nice.org.uk/CG69/NICEGuidance>.
