# Supplementary material for: Clinical indicators for common paediatric conditions: Processes, provenance and products of the CareTrack Kids study
Source: PLoS One. 2019 Jan 9;14(1):e0209637. doi: 10.1371/journal.pone.0209637 (PMC6326465; doi:10.1371/journal.pone.0209637)
Supplement: S5 Table — (DOCX) [file pone.0209637.s005.docx]

**S5 Table: Evolution of numbers of indicators over the development process, from original recommendations to the final indicators and medical record audit indicator items, per condition**

| **Conditions** | **Original**  **recommendations**  **extracted** | **Original**  **recommendations**  **included** | **Internal review**  **(Round 1)** | **Internal review**  **(Round 2)** | **Internal review**  **(Round 3)** | **External review - wiki**  **(Round 1)** | **External review - wiki**  **(Round 2)** | **Post-external review** | **FINAL indicators** | **FINAL indicator items** |
| --- | --- | --- | --- | --- | --- | --- | --- | --- | --- | --- |
| ABDO | 30 | 27 | 12 | 11 | 8 | 8 | 8 | 8 | 7 | 21 |
| ADHD | 43 | 36 | 26 | 17 | 14 | 13 | 13 | 13 | 12 | 34 |
| AGE | 31 | 31 | 26 | 15 | 12 | 12 | 12 | 12 | 12 | 35 |
| ANXI | 88 | 73 | 18 | 11 | 10 | 10 | 10 | 10 | 7 | 13 |
| ASTH | 109 | 80 | 36 | 25 | 22 | 22 | 21 | 21 | 21 | 39 |
| AUTI | 76 | 58 | 27 | 17 | 13 | 13 | 13 | 13 | 10 | 17 |
| BRON | 56 | 51 | 16 | 16 | 16 | 16 | 16 | 16 | 16 | 40 |
| CROU | 53 | 49 | 22 | 20 | 15 | 15 | 14 | 14 | 12 | 26 |
| DEPR | 147 | 38 | 12 | 12 | 10 | 10 | 10 | 10 | 8 | 15 |
| DIAB | 233 | 156 | 52 | 21 | 19 | 19 | 17 | 17 | 15 | 35 |
| ECZE | 65 | 54 | 21 | 15 | 11 | 11 | 11 | 11 | 9 | 9 |
| FEVE | 87 | 79 | 39 | 25 | 14 | 14 | 13 | 13 | 13 | 47 |
| GORD | 44 | 36 | 15 | 15 | 18 | 19 | 19 | 19 | 15 | 32 |
| HEAD | 105 | 83 | 25 | 24 | 17 | 17 | 17 | 17 | 17 | 54 |
| OBES | 36 | 30 | 12 | 8 | 6 | 5 | 5 | 5 | 5 | 18 |
| OTIT | 54 | 51 | 18 | 14 | 13 | 12 | 12 | 12 | 11 | 37 |
| PREV | 71 | 70 | 24 | 14 | 11 | 11 | 9 | 9 | 7 | 43 |
| SEIZ | 38 | 35 | 17 | 15 | 15 | 15 | 14 | 14 | 14 | 33 |
| TONS | 25 | 24 | 13 | 8 | 7 | 7 | 7 | 7 | 7 | 11 |
| URIN | 21 | 19 | 13 | 11 | 12 | 12 | 12 | 12 | 12 | 24 |
| URTI | 20 | 18 | 7 | 5 | 5 | 5 | 5 | 5 | 4 | 14 |
| *Total* | 1432 | 1098 | 451 | 319 | 268 | 266 | 258 | 258 | 234 | 597 |
| *Mean*  *(SD)* | 68.2 (50.1) | 52.3 (31.4) | 21.5 (10.7) | 15.2 (5.5) | 12.8 (4.3) | 12.7 (4.5) | 12.3 (4.3) | 12.3 (4.3) | 11.1 (4.3) | 28.4  (12.8) |
| *Median* | 54 | 49 | 18 | 15 | 13 | 12 | 12 | 12 | 12 | 32 |
