# Supplementary material for: Clinical indicators for common paediatric conditions: Processes, provenance and products of the CareTrack Kids study
Source: PLoS One. 2019 Jan 9;14(1):e0209637. doi: 10.1371/journal.pone.0209637 (PMC6326465; doi:10.1371/journal.pone.0209637)
Supplement: S6 Table — (DOCX) [file pone.0209637.s006.docx]

**S6 Table: Characteristics of included medical record audit indicator items**

| **Condition** | **Medical record audit indicator items (n)** | **Phase of care** | | | | **Healthcare practice facility** | | | | **Type** | | **Grade of recommendation*** | | | | **Level of evidence*** | | | | |
| --- | --- | --- | --- | --- | --- | --- | --- | --- | --- | --- | --- | --- | --- | --- | --- | --- | --- | --- | --- | --- |
|  |  | **Screening** | **Diagnosis** | **Treatment** | **Ongoing management** | **Emergency Department** | **General Practice** | **Hospital** | **Specialist** | **Overuse** | **Underuse** | **A** | **B** | **C** | **D** | **I** | **II** | **III** | **IV** | **Consensus recommendations** |
| ABDO | 21 | 0 | 15 | 6 | 0 | 21 | 19 | 21 | 19 | 3 | 18 | 2 | 0 | 0 | 0 | 0 | 0 | 0 | 0 | 19 |
| ADHD | 34 | 0 | 15 | 9 | 10 | 0 | 29 | 0 | 31 | 0 | 34 | 1 | 3 | 0 | 0 | 0 | 0 | 0 | 0 | 30 |
| AGE | 35 | 0 | 10 | 13 | 12 | 34 | 18 | 26 | 0 | 4 | 31 | 0 | 0 | 0 | 0 | 0 | 0 | 0 | 0 | 35 |
| ANXI | 13 | 0 | 4 | 9 | 0 | 7 | 13 | 7 | 11 | 2 | 11 | 0 | 0 | 0 | 0 | 0 | 0 | 0 | 0 | 13 |
| ASTH | 39 | 0 | 4 | 20 | 15 | 36 | 38 | 37 | 36 | 6 | 33 | 0 | 0 | 0 | 0 | 0 | 1 | 0 | 0 | 38 |
| AUTI | 17 | 0 | 8 | 6 | 3 | 0 | 17 | 0 | 17 | 0 | 17 | 0 | 0 | 4 | 8 | 0 | 0 | 0 | 0 | 5 |
| BRON | 40 | 0 | 13 | 19 | 8 | 39 | 23 | 37 | 0 | 8 | 32 | 1 | 8 | 5 | 8 | 3 | 0 | 1 | 0 | 20 |
| CROU | 26 | 0 | 13 | 7 | 6 | 25 | 23 | 25 | 0 | 8 | 18 | 2 | 1 | 1 | 14 | 1 | 4 | 0 | 1 | 6 |
| DEPR | 15 | 0 | 7 | 7 | 0 | 14 | 15 | 14 | 15 | 1 | 14 | 1 | 4 | 3 | 2 | 0 | 0 | 0 | 0 | 5 |
| DIAB | 35 | 0 | 2 | 25 | 8 | 32 | 13 | 33 | 14 | 0 | 35 | 0 | 6 | 0 | 0 | 0 | 0 | 0 | 0 | 29 |
| ECZE | 9 | 0 | 2 | 5 | 2 | 8 | 8 | 9 | 8 | 1 | 8 | 0 | 2 | 1 | 0 | 0 | 0 | 0 | 0 | 6 |
| FEVE | 47 | 0 | 21 | 25 | 1 | 43 | 38 | 43 | 34 | 1 | 46 | 0 | 0 | 0 | 0 | 0 | 3 | 0 | 0 | 44 |
| GORD | 32 | 0 | 11 | 11 | 10 | 30 | 32 | 30 | 30 | 4 | 28 | 5 | 0 | 2 | 9 | 0 | 0 | 0 | 0 | 16 |
| HEAD | 54 | 0 | 4 | 49 | 1 | 54 | 21 | 52 | 0 | 2 | 52 | 0 | 6 | 0 | 0 | 0 | 0 | 0 | 0 | 48 |
| OBES | 18 | 0 | 10 | 7 | 1 | 0 | 18 | 0 | 18 | 0 | 18 | 0 | 5 | 2 | 0 | 0 | 0 | 0 | 0 | 11 |
| OTIT | 37 | 0 | 2 | 14 | 21 | 37 | 37 | 37 | 37 | 4 | 33 | 6 | 7 | 1 | 2 | 0 | 0 | 0 | 0 | 23 |
| PREV | 43 | 43 | 0 | 3 | 0 | 0 | 43 | 0 | 0 | 0 | 43 | 0 | 3 | 3 | 0 | 0 | 0 | 0 | 0 | 37 |
| SEIZ | 33 | 0 | 14 | 16 | 3 | 33 | 0 | 30 | 0 | 0 | 33 | 0 | 1 | 0 | 8 | 0 | 0 | 0 | 0 | 24 |
| TONS | 11 | 0 | 1 | 4 | 6 | 6 | 6 | 11 | 6 | 2 | 9 | 4 | 0 | 1 | 2 | 0 | 0 | 0 | 0 | 4 |
| URIN | 24 | 0 | 9 | 12 | 3 | 24 | 9 | 24 | 0 | 0 | 24 | 0 | 0 | 0 | 0 | 0 | 0 | 0 | 0 | 24 |
| URTI | 14 | 0 | 7 | 6 | 1 | 14 | 14 | 14 | 0 | 0 | 14 | 0 | 0 | 0 | 0 | 0 | 0 | 0 | 0 | 14 |

| Total | 597 | 0 | 172 | 273 | 111 | 457 | 434 | 450 | 276 | 46 | 551 | 22 | 46 | 23 | 53 | 4 | 8 | 1 | 1 | 451 |
| --- | --- | --- | --- | --- | --- | --- | --- | --- | --- | --- | --- | --- | --- | --- | --- | --- | --- | --- | --- | --- |

* some medical record audit indicator items were based on more than one grade of recommendation or level of evidence

BRON14 (C, I); BRON16 (C, I); BRON17 (A, III); BRON19 (C, D); BR0N23 (C, D)

CROU18 (I, II, IV); CROU21 (A, II); CROU22 (A, II, II)

OTIT16 (B, B, D)
