# Supplementary material for: Clinical indicators for common paediatric conditions: Processes, provenance and products of the CareTrack Kids study
Source: PLoS One. 2019 Jan 9;14(1):e0209637. doi: 10.1371/journal.pone.0209637 (PMC6326465; doi:10.1371/journal.pone.0209637)
Supplement: S7 Table — (DOCX) [file pone.0209637.s007.docx]

**S7 Table: Mapping of the *CareTrack Kids* approach to practical guidance for using and reporting Delphi procedures (17)**

| **Methodological consideration** | ***CareTrack Kids* approach and reporting** |
| --- | --- |
| Initial Delphi round | - Define study objectives - Describe method for selecting indicators - Report numbers of indicators in first Delphi round (Figure 2, Appendix 5) - Develop and include scoring criteria for indicators (Box 3) - Establish definition for consensus |
| Experts^ | - Describe recruitment methods - Provide composition and characteristics of expert panel - Report response rates for each round |
| Mode of delivery | - Describe vehicle for disseminating Delphi survey (Figure 1) |
| Subsequent Delphi rounds | - Provide flow of indicators eliminated and added at each round   (Figure 2)   - Report method for providing feedback to Delphi participants   (Figure 1, Box 4) |
| Final Delphi round | - Report duration of Delphi procedure - Include list of selected indicators and scores (Appendix 1) |

^ in accordance with study objectives, experts were limited to Australian healthcare professionals (i.e. did not include informal caregivers or patients, or experts from different countries)
